# A comprehensive investigation discovered the novel methyltransferase METTL24 as one presumably prognostic gene for kidney renal clear cell carcinoma potentially modulating tumor immune microenvironment

**DOI:** 10.3389/fimmu.2022.926461

**Published:** 2022-10-14

**Authors:** Zhongji Jiang, Wei Zhang, Zhipeng Zeng, Donge Tang, Chujiao Li, Wanxia Cai, Yumei Chen, Ya Li, Qiu Jin, Xinzhou Zhang, Lianghong Yin, Xueyan Liu, Yong Xu, Yong Dai

**Affiliations:** ^1^ Clinical Medical Research Center, The Second Clinical Medical College of Jinan University, Shenzhen People’s Hospital, Shenzhen, China; ^2^ Department of Laboratory Medicine, Shenzhen Institute of Translational Medicine, The First Affiliated Hospital of Shenzhen University, Shenzhen Second People’s Hospital, Shenzhen, China; ^3^ Key Renal Laboratory of Shenzhen, Department of Nephrology, Shenzhen People’s Hospital, The Second Clinical Medical College of Jinan University, Shenzhen, China; ^4^ Department of Nephrology, Institute of Nephrology and Blood Purification, The First Affiliated Hospital of Jinan University, Jinan University, Guangzhou, China; ^5^ Department of Intensive Care Unit, Shenzhen Key Laboratory of Prevention and Treatment of Severe Infections, The Second Clinical Medical College of Jinan University (Shenzhen People’s Hospital), Shenzhen, China

**Keywords:** METTL24, methyltransferase, immune microenvironment, prognostic biomarker, kidney cancer

## Abstract

**Background:**

Recently, an increasing number of studies have uncovered the aberrant expression of methyltransferase-like family (METTL) plays an important role in tumorigenesis, such as METTL3 (an m6A writer). In our recent work, we discovered METTL24 expression was highly associated with the hazard ratio (HR) of kidney renal clear cell carcinoma (KIRC) compared to other tumors, implying a special function of METTL24 in KIRC carcinogenesis. Until now, the functions and mechanisms of METTL24 in KIRC have remained mostly unknown.

**Methods:**

The mRNA expression of METTL24 in KIRC was analyzed using the TIMER 2.0, GEPIA, and UALCAN databases. The immunohistochemical assay was performed to validate METTL24 expression in our self-built Chinese cohort (n _tumor_ = 88, n _normal_ = 85). The gene set enrichment analysis (GSEA) was used to investigate the biological processes in which METTL24 might be engaged. The Spearman analysis was used to evaluate the expression correlations between METTL24 and a range of immunological variables, and the effects of METTL24 on the infiltration levels of multiple immune cells were explored using TCGA data. The upstream transcription factors of METTL24 were screened through a multi-omics analysis.

**Results:**

METTL24 expression in KIRC tissues was significantly decreased compared to normal adjacent kidney tissues, which was associated with the lower survival rate of KIRC patients. METTL24 potentially participated in the immune-relevant biological processes such as cytokine binding, NF-kappa B binding, MHC protein complex, and interleukin-12 action. Besides, METTL24 expression was linked to a number of immune checkpoints, cytokines, chemokines, and chemokine receptors, and also correlated with the infiltration levels of 10 types of immune cells in KIRC. Meanwhile, METTL24 expression differently affected the overall survival rates (OS) of KIRC patients with high or low levels of immune infiltration. Finally, CTCF and EP300 were discovered to be the probable transcription factors of METTL24 in KIRC.

**Conclusion:**

This study revealed that METTL24 might serve as a prognostic marker in KIRC and as one immune-relevant target for clinical treatment.

## Introduction

After prostate and bladder cancer, kidney cancer is the third most frequent urological malignancy, with 431,288 new cases and 179,368 deaths reported by the Global Cancer Observatory in 2020 (1). Kidney renal clear cell carcinoma (KIRC) is the most frequent subtype of kidney cancer, accounting for over 70% of all cases each year (2). Due to the lack of typical manifestations and screening indicators, KIRC is usually diagnosed at an advanced stage, which makes therapy challenging and increases the risk of recurrence (3). Patients with metastatic KIRC have a 5-year survival rate of only 10% (4).

Anti-tumor therapy has been incredibly challenging for a long time since the focus on tumor treatment has been limited to the tumor itself. The tumor microenvironment (TME), consisting of immune cells, endothelial cells, fibroblasts, and various biological molecules, is critical for carcinogenesis and therapeutic responses (5–7). Immune checkpoint blockade (ICB) therapy has achieved success in multiple cancers (8), but the evidence for its effectiveness in kidney cancer is inconclusive, and only some patients may benefit from it (9, 10). Therefore, more research into potential treatment targets for kidney cancer is required.

Methyltransferase-like (METTL) genes encode proteins with seven β-chain with S-adenosylmethionine binding domains that typically act as methyltransferases, writing methylations on DNAs, RNAs, or proteins (11–13). Recent studies have demonstrated that the aberrant expression of METTL family genes, such as METTL3 (one m6A writer), plays a key role in tumorigenesis (11, 14). In our previous study, we found that the METTL family was more closely associated with the risk of kidney cancer than other malignancies (Wei Zhang, et al), indicating that these proteins might be crucial for the initiation and progression of kidney cancer. Also, we identified METTL24 as one protecting factor for renal cancer (**Supplementary Figure S1**) . There are currently few reports about METTL24, and only one article has proclaimed that METTL24 expression is likely connected with the prognosis of rectal cancer patients (15). METTL24’s role and mechanisms in tumorigenesis are still barely known.

In this study, we analyzed METTL24 expression in KIRC tissues and normal adjacent tissues using the data sets from the Cancer Genome Atlas (TCGA) and Genotype-Tissue Expression (GTEx) databases (n _tumor_ = 523, n _normal_ = 72) and evaluated its prognostic value for KIRC patients. Next, we clarified the potential biological processes and pathways that METTL24 was involved in. Subsequently, we explored the role of METTL24 in the immune microenvironment, including analyzing the correlation between METTL24 expression and the infiltration ratios of immune cells and investigating the expression correlation between METTL24 and several immune families. We also looked at the impact of METTL24 on the survival rates of KIRC patients with high or low immune infiltration. Ultimately, we explored the potential transcription factors of METTL24 in KIRC.

## Methods

### Patients

The Shanghai Outdo Biotech Company contributed 90 tumorous samples and paired normal nearby samples from KIRC patients. Patient exclusion criteria: Older than 85 years old or younger than 18 years old, with severe organ failure, as well as a history of chemotherapy or radiotherapy. All participants gave their informed consent, and the Ethics Committee of Shanghai Outdo Biotech Company approved this study (No.YB-M-05-02), which followed the World Medical Association’s Code of Ethics.

### Immunohistochemistry assay

The tissue sections were incubated at 63°C for 60 minutes before being immersed in xylene for 15 minutes. Then, the soaking step was repeated once more. The chips were dewaxed twice in 100% ethanol for 7 minutes, then in 90%, 80%, and 70% ethanol for 5 minutes each on LEICAST5020 fully automated stainers (Leica, Biosystems), with antigen retrieval using the Automatic DAKO PT-Link. After that, the chips were placed in distilled water at room temperature to allow natural cooling for 10 minutes. The chip was then washed three times with the PBS solution, each time for 5 minutes. Following that, the chip was incubated at 4°C for 12 hours with METTL24 antibody incubation solution (Invitrogen, PA557952, 1:1000). The rewarming process was carried out at room temperature for 45 minutes. The chip was then washed three times with the PBS solution, each time for 5 minutes. A biotin blocking kit was used to saturate the endogenous biotin (Maixin, BLK-0002), and the chip was then incubated at 37°C for one hour with the secondary antibody incubation solution (Boster, SA1055). The chip was cleaned three times with PBS solution for 5 minutes each time before being colored with diaminobenzidine (DABs). After that, the chip was stained with hematoxylin for 1 minute, submerged in 0.25% hydrochloric acid alcohol for around 10 seconds, then washed for 5 minutes with running water. The chip was dehydrated and sealed after being cleaned with running water for 10 minutes. The stained chip was scanned with the Aperio Scanner (LEICA, Aperio XT), and two pathologists evaluated the staining intensity and proportion of stained cells. Under low magnification, the staining intensity was graded as follows: The score of light yellow was 1, brownish yellow was 2, and tan was 3. In terms of the proportion, three different staining intensity fields were chosen to measure their positive rate, and then the average value was calculated. Finally, the staining score was calculated by multiplying the staining intensity and proportion.

### Tumor immune estimation resource online database 2.0 (TIMER 2.0)

TIMER 2.0 (16) is a comprehensive database that displays the amount of various immune cells within cancer as computed using the immune deconvolution approach and provides gene expression in tumorous tissues vs normal neighboring tissues. The “Exploration-Gene DE” module of TIMER 2.0 was used to assess METTL24 expression in more than 30 tumors and nearby normal tissues, and the link between METTL24 expression and immune cell infiltration ratios was also explored using the TIMER 2.0 database.

### Kaplan-Meier plotter

Kaplan-Meier plotter (17) is a database collecting the published microarray and RNA-sequencing data sets from GEO, European Genome-Phenome Archive (EGA), and TCGA databases, with an emphasis on the discovery and verification of survival relevant genes across all malignancies. In our research, the “kidney cancer RNA-seq” module was utilized to assess the effect of METTL24 mRNA expression on the overall survival rate (OS) of KIRC patients. In **Figure 4A**, according to the best cut-off value, the patients were divided into METTL24-high-expression and METTL24-low-expression groups; in **Figure 4B**, “median value” was used to divide patients into two groups. Using the Kaplan-Meier plotter, the effect of METTL24 expression on the OS of patients with high or low levels of immune infiltration was also investigated.

### Human transcription factor targets (hTFtarget)

The hTFtarget database (18) is a transcription factor database that includes a significant number of human chromatin immunoprecipitation (ChIP) sequencing data sets (7,190 experimental samples) and 659 identified transcription factors. The hTFtarget database was used to find the seven possible upstream transcription factors of METTL24 in kidney tissue.

### Linkedomics

Linkedomics (19) is a comprehensive database that combines the multi-omics data sets from the Clinical Proteomic Tumor Analysis Consortium (CPTAC) and TCGA databases. The METTL24 co-expressed genes in KIRC tissues were retrieved from the Linkedomics database, and gene set enrichment analysis (GSEA) of METTL24 was performed using the Linkedomics’ module “LinkInterpreter”.

### Gene expression profiling interactive analysis (GEPIA)

GEPIA (20) is an online database containing data sets of 9736 tumor samples and 8587 normal samples from TCGA and The Genotype-Tissue Expression (GTEx) projects. Here, METTL24 expression between tumorous tissues of KIRC, kidney chromophobe (KICH), and kidney renal papillary cell carcinoma (KIRP) and normal surrounding tissues was analyzed using the “Expression DIY” module of GEPIA. The Spearman method was used for the expression correlation between AR, CTCF, EP300, and METTL24.

### UALCAN

UALCAN (21) is an interactive network database that gathers multi-platform-based data sets from other publicly available databases like TCGA, MET500, and CPTCA, and lets users to analyze them. In this study, the UALCAN database was utilized to study METTL24 expression in different patient groups. Meanwhile, the UALCAN database was used to look into protein expression and phosphoprotein levels (AR, CTCF, and EP300).

### SangerBox

SangerBox is an integrated bioinformatic analysis tool that can perform a wide range of bioinformatic studies and visualizations. SangerBox was used to compare METTL24 expression in tumor tissues from 27 cancers to normal tissues in this study.

### Assistant for clinical bioinformatics (ACLBI)

ACLBI is a web-based interactive application that allows users to analyze data from TCGA, the Cancer Cell Line Encyclopedia (CCLE), and the International Cancer Genome Consortium (ICGC) databases. ACLBI was utilized in this study to execute univariate and multivariate Cox regression analysis to identify the risk factors and independent prognostic genes in KIRC. Also, the nomogram was drawn based on the results of multivariate Cox regression analysis, which provides a graphical representation of multiple factors that might be used to assess the risk of recurrence. The ACLBI was also used to study the impact of METTL24 expression on OS and progression-free survival rate (PFS). Furthermore, the correlation of METTL24 mRNA expression and immune-relevant scores and immune cell infiltration levels were calculated using the algorithms including MCPCOUNTER, QUANTISEQ, and TIMER in ACLBI.

### Statistical analyses

The log-rank method was utilized to determine the significance of METTL24 expression on patient survival. Fisher’s exact test was used to analyze the significance of enrichment results. And the Spearman method was used for the correlation analysis. A p-value of less than 0.05 was regarded as significant. ***p < 0.001, **p < 0.01, and *p < 0.05.

## Results

### METTL24 is decreasingly expressed in tumor tissues versus normal tissues in most cancers

**Figure 1:**
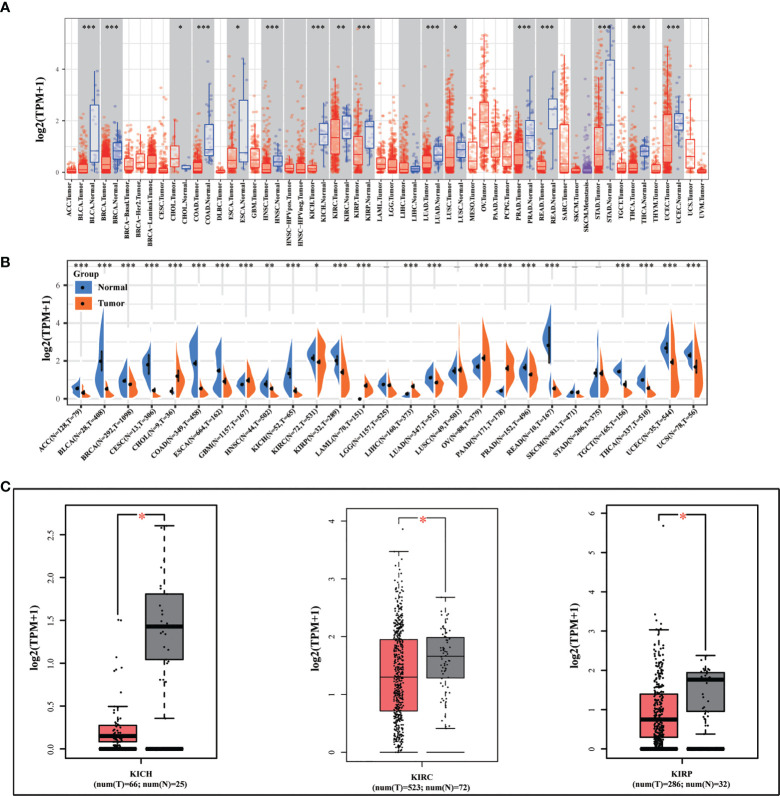
METTL24 was decreasingly expressed in KIRC tissues versus normal kidney tissues. **(A)** METTL24 expression in various cancers and normal tissues as analyzed using the TIMER database. **(B)** METTL24 expression in various cancers and normal tissues as analyzed using the data sets from TCGA and GTEx. **(C)** METTL24 expression in KIHC, KIRC, and KIRP tissues and adjacent normal tissues as analyzed using the GEPIA database. ***p < 0.001, **p < 0.01 and *p < 0.05.

We initially looked at the expression of METTL24 in various cancers to see if it had a role in carcinogenesis. We discovered that METTL24 was considerably lower expressed in tumor tissues compared to normal tissues in 15 types of tumors as analyzed using data sets from TCGA (**Figure 1A**). Besides, we revealed that METTL24 was decreasingly expressed in cancer tissues of 17 tumors compared to normal tissues using data sets from TCGA and GTEx (**Figure 1B**). As we were focusing on kidney cancer, we confirmed METTL24 expression in KICH, KIRC, KIPR tissues and normal neighboring kidney tissues using the GEPIA database (**Figure 1C**).

### The validation of METTL24 expression based on a self-built Chinese cohort

**Figure 2:**
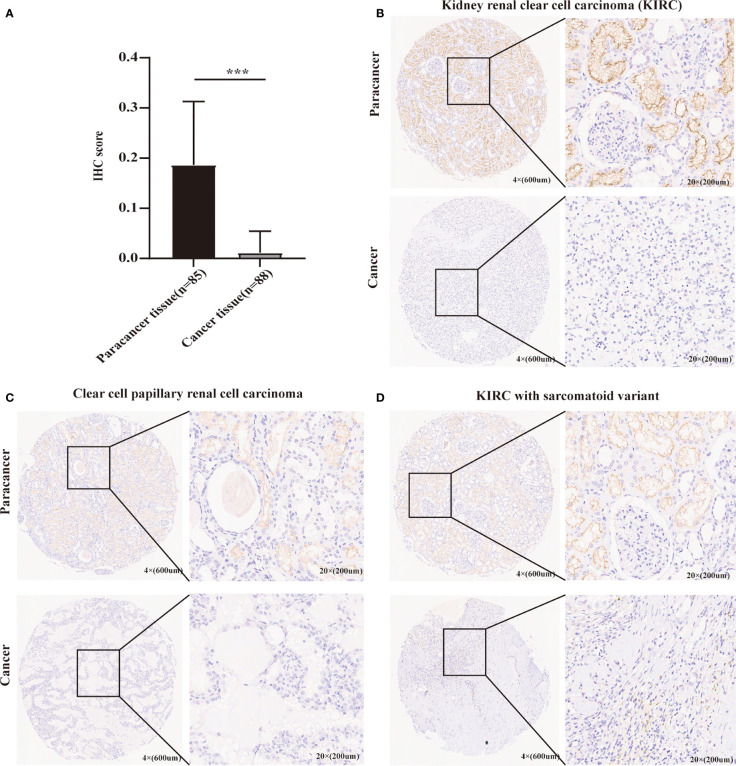
The verification of METTL24 expression in KIRC tissues and normal adjacent tissues using our self-built Chinese cohort. **(A)** The protein level of METTL24 was significantly decreased in KIRC tissues compared with normal adjacent tissues (n _tumor_ = 88, n _normal_ = 85); T-test was used for the statistical analysis. **(B-D)** Representative images of METTL24 expression in KIRC tissues and paracancerous tissues. The immunohistochemical assay was performed for detecting METTL24 expression. ***p < 0.001.

As mentioned above, METTL24 was significantly downregulated in three renal cancer subtypes, and since KIRC accounts for more than 70% of renal malignancies (2), this study selected KIRC as a representative model of renal cancer for follow-up studies. To verify METTL24 expression in KIRC, we used IHC to detect METTL24 protein expression in 88 cancer samples and 85 para-cancer samples (**Table 1**). The result indicated that the protein level of METTL24 in KIRC tissues was appreciably lower than normal adjacent tissues (p<0.0001) (**Figure 2A**). **Figure 2B** depicts METTL24 protein expression in KIRC tissue and para-cancer, whereas **Figures 2C, D** depict exemplary microscopic illustrations of clear cell papillary renal cell carcinoma (CCPRCC) and KIRC with sarcomatoid variation, respectively. These findings showed that METTL24 expression was lower in tumorous tissues compared to normal surrounding tissues in Chinese KIRC patients, which was in line with the above computer-analyzing-based results.

**Table 1:** The characteristics of 90 kidney cancer patients of self-built Chinese cohort.

	Type	Patients
**Age**	≤65	60 (66.7%)
	>65	30 (33.3%)
**Sex**	Female	59 (65.6%)
	Male	31 (34.4%)
**Subtypes**	Kidney clear cell renal cell carcinoma (KIRC)	87 (96.7%)
	KIRC with partial papillary renal cell carcinoma	2 (2.2%)
	KIRC with partial sarcomatoid variant	1 (1.1%)
**Staging**	1	55 (61.1%)
	2	24 (26.7%)
	3	6 (6.7%)
	4	2 (2.2%)
	–	3 (3.3%)
**T-stage**	T1	58 (64.4%)
	T2	25 (27.8%)
	T3	7 (7.8%)
	–	0 (0.0%)
**N-stage**	N0	85 (94.5%)
	N1	2 (2.2%)
	N2	0 (0.0%)
	N3	0 (0.0%)
	–	3 (3.3%)
**M-stage**	M0	88 (97.8%)
	M1	2 (2.2%)
	–	0 (0.0%)

**“–”:** The staging and T-stage of patients with distal or lymphatic metastases was not identified.

### METTL24 expression in different KIRC groups

**Figure 3:**
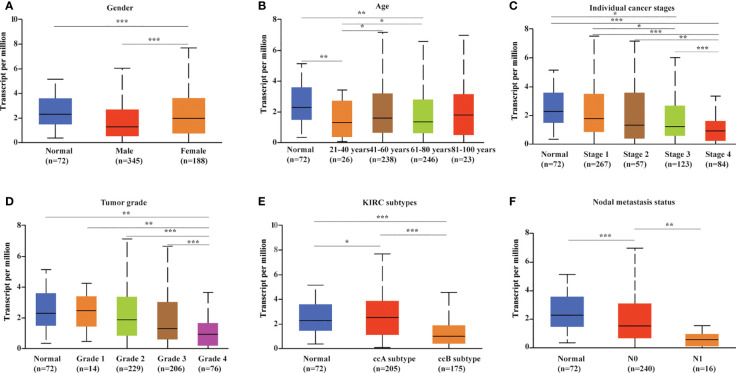
METTL24 expression in different patient groups. The box plots showed the relationship between METTL24 expression and parameters including **(A)** sex, **(B)** age, **(C)** cancer stage, **(D)** tumor grade, **(E)** KIRC subtype, **(F)** nodal metastasis status was analyzed using the UALCAN database. The T-test was used to estimate the statistical significance of expression differences between distinct groups. ***p < 0.001,**p < 0.01 and *p < 0.05.

We evaluated METTL24 expression in different clinical subgroups using data sets from the UALCAN database to see if there was a link between METTL24 expression and other KIRC clinical characteristics. In male KIRC patients, METTL24 expression was downregulated in tumorous tissues compared to normal surrounding tissues, but not in females (**Figure 3A**). When it came to age, METTL24 expression dropped drastically in KIRC tissues compared to normal tissues in patients of various ages (21-40 years, 61-80 years), but there was no difference between the age groups (**Figure 3B**). In terms of tumor stage, METTL24 expression revealed a substantial downregulation trend as tumor grade increased, with grade 4 having the lowest expression (**Figure 3C**). In addition, as tumor stages progressed, the level of METTL24 in KIRC tissues fell considerably (**Figure 3D**). In terms of tumor subtypes, METTL24 expression was significantly lower in CCB tumorous tissues than in normal tissues (**Figure 3E**), but it was much greater in CCA carcinogenic tissues than in normal samples. Furthermore, as the N stage increased, METTL24 expression showed a strong downregulation trend (**Figure 3F**). The results regarding grade, stage, and N stage elucidated that METTL24 was potentially related to the advancement of KIRC.

### METTL24 as an independent prognostic gene for KIRC

**Figure 4:**
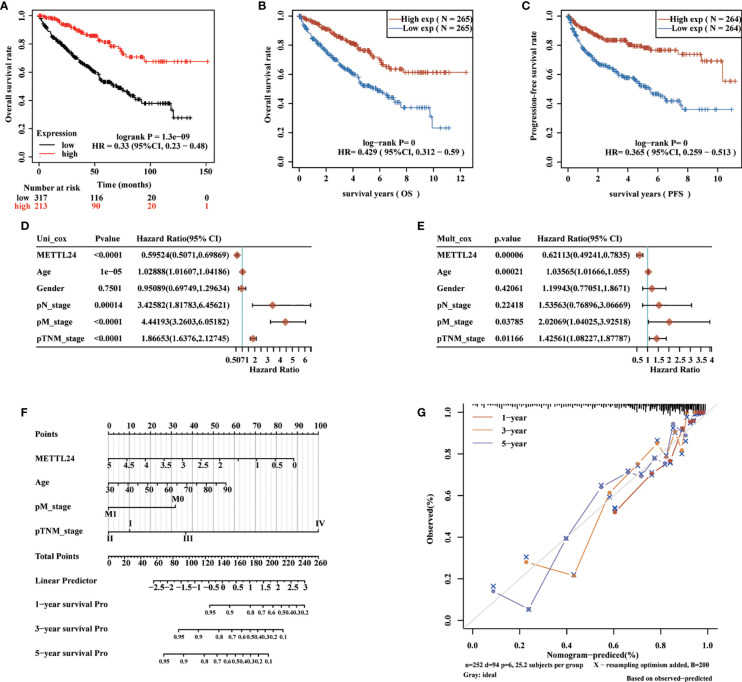
METTL24 as one potential prognostic gene for KIRC. **(A, B)** The Kaplan-Meier curve showed the impacts of METTL24 expression on the OS **(C)** and PFS of KIRC patients. **(D)** The forest image showed METTL24 as a potential risk factor for KIRC as analyzed using the univariate Cox regression. **(E)** The forest image showed METTL24 as one potential prognostic factor for KIRC as analyzed using the multivariate Cox regression. **(F)** A predictive nomogram based on the METTL24 risk score and other clinicopathological variables predicted the 1-, 3-, and 5-year survival rates of KIRC patients. **(G)** The Calibration curves indicated the agreement between anticipated and actual survival rates after 1, 3, and 5 years.

To preliminarily explore the function of METTL24 in KIRC, we plotted the survival curve of METTL24 using TCGA data sets. As a result, reduced METTL24 expression in KIRC was linked to a shorter OS, PFS and DSS, but not RFS or DFS (**Figures 4A–C**, **Supplementary Figures S2**). Following that, we conducted the univariate Cox regression analysis using OS data, which revealed that low METTL24 expression was a risk factor for KIRC (HR= 0.59, 95% confidence interval (CI) = 0.50-0.70, p <0.0001) (**Figure 4D**). Furthermore, METTL24 was discovered as an independent predictive predictor for KIRC in a multivariate Cox regression analysis based on the OS (**Figure 4E**). Similarly, we used disease-specific survival (DSS) and PFS to conduct univariate and multivariate Cox regression analysis and came to similar conclusions (**Supplementary Figures S3**). Moreover, we created a nomogram that included METTL24 expression and TNM stages to predict the OS, DSS, and PFS of KIRC patients. The forecast model’s anticipated values were highly compatible with the actual 1-, 3-, and 5-year OS, DSS, and PFS, according to the findings. The OS, DSS, and PFS nomograms had concordance indexes of 0.778, 0.85, and 0.842, respectively (**Figures 4D–G**, **Supplementary Figures S3**). As a result, this nomogram may aid clinicians in identifying high-risk and low-risk patients and providing tailored treatment.

### METTL24 may play a role in KIRC’s immune microenvironment modulation

**Figure 5:**
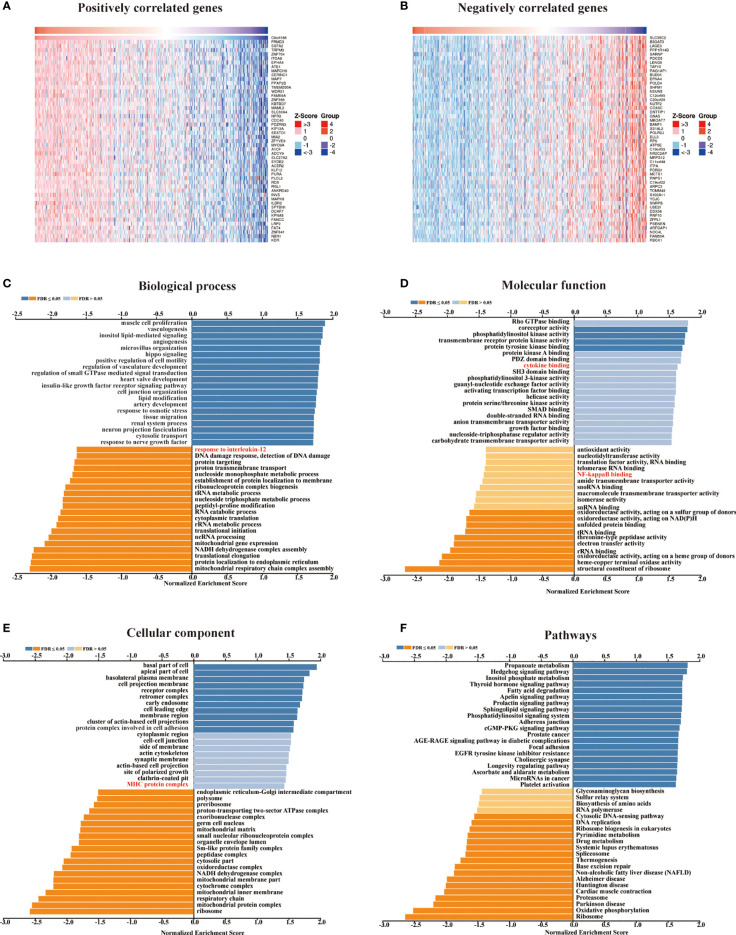
In KICR, METTL24 most likely served an immune function. **(A, B)** The heat maps showed the top 50 positively and negatively expression-correlated genes of METTL24. The METTL24 relevant **(C)** biological processes (BP), **(D)** molecular functions (MF), **(E)** cellular compartments (CC), **(F)** and pathways in KIRC.

We retrieved 1,3977 co-expressed mRNAs of METTL24 in KIRC patients from the Linkedomics database to investigate the mechanism of METTL24 in KIRC (**Supplementary Table S1**). **Figures 5A,B** showed the 50 METT24 genes that were most positively and adversely associated. Then, using an enrichment analysis of these co-expressed genes, we looked into the biological processes that METTL24 might be involved in. As a consequence, METTL24 was presumably connected with a range of immunological functions, including cytokine binding, NF-kappa B binding, MHC protein complex, and interleukin-12 activity (**Figures 5C–F**), suggesting METTL24 might have a role in the control of immune milieu of KIRC.

**Figure 6:**
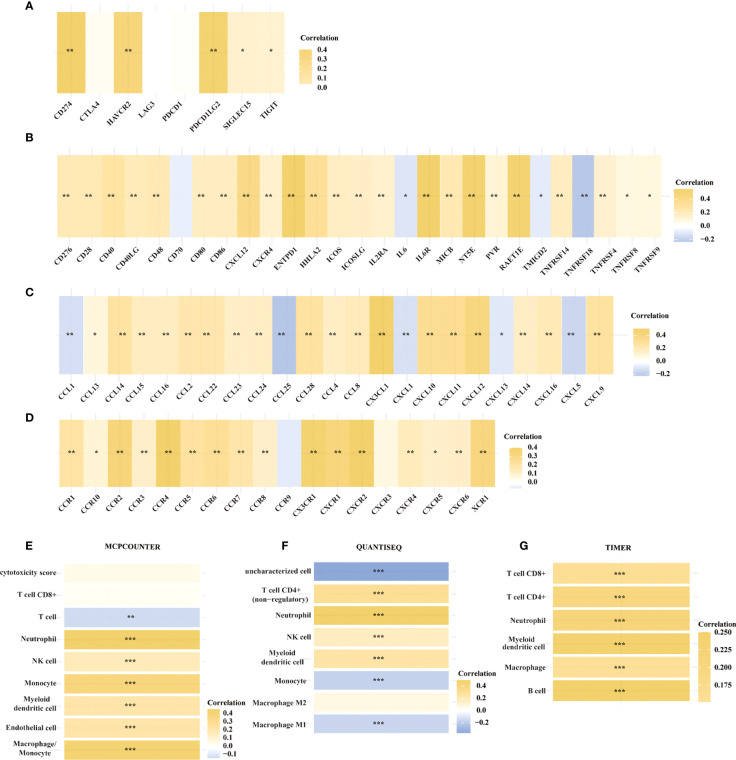
The expression of METTL24 was potentially correlated with various immune genes and the infiltration levels of multiple immune cells in KIRC. The expression correlation between METTL24 and **(A)** typical immune checkpoints, **(B)** cytokines, **(C)** chemokines, and **(D)** chemokine receptors; The correlation between METTL24 expression and the infiltration degrees of distinct immune cells as analyzed using **(E)** MCPCOUNTER, **(F)** QUANTISEQ, and **(G)** TIMER algorithms. ***p < 0.001, **p < 0.01 and *p < 0.05.

We then looked at the relationship between the expression of METTL24 and numerous immune genes in KIRC to confirm the immunological functions of METTL24. As demonstrated in **Figure 6A**, METTL24 and the traditional immunological checkpoints CD274, HAVCR2, PDCD1LG2, SIGLEC15, and TIGIT have a significant positive expression connection. METTL24 was also discovered to be commonly co-expressed with a variety of cytokines, including CD276, CD28, CD40, CD40LG, CD48, CD80, CD86, CXCL12, CXCR4, ENTPD1, HHLA2, ICOS, ICOSLG, IL2RA, IL6R, MICB, NT5E, PVR, RAET1E, TNFRSF14, TNFRSF4 (**Figure 6B**). CCL13, CCL14, CCL15, CCL16, CCL2, CCL22, CCL23, CCL24, CCL28, CCL4 CCL8, CX3CL1, CXCL10, CXCL11, CXCL12, CXCL14, CXCL16, CXCL9, CCL1, CCL25, CXCL1, CXCL13, and CXCL15 were also substantially linked with METTL24 expression. METTL24 expression was also tied to the expression of a number of chemokine receptors, including CCR1, CCR10, CCR2, CCR3, CCR4, CCR5, CCR6, CCR7, CCR8, CX3CR1, CXCR1, CXCR2, CXCR4, CXCR5, CXCR6, CXCR9, and CXCR3 (**Figures 6C, D**). Notably, the expression of METTL24 was positively connected with the majority of immune genes, with only a few immune genes being adversely correlated. The foregoing finding also suggested that METTL24 might play a function in immunological modulation in KIRC patients.

Following that, we used various algorithms, such as MCPCOUNTER, QUANTISEQ, and TIMER, to investigate the relationship between METTL24 expression and the infiltration ratios of various immune cells (**Figures 6E–G**). As a result, the infiltration levels of 10 types of immune cells in KIRC, including CD4+ T cells, CD8+ T cells, neutrophils, natural killer (NK) cells, myeloid dendritic cells, macrophages, and B cells, were correlated with METTL24 expression. CD4+ T cells, CD8+ T cells, neutrophils, natural killer (NK) cells, and myeloid dendritic cells were all positively linked with METTL24 expression among these immune cells. Infiltrating CD4+ T cells and CD8+ T cells within malignancies are known to limit tumor activity, whereas Tregs are more likely to promote tumor growth (22, 23). Our findings revealed that METTL24 was more likely to be a tumor suppressor gene in KIRC tumorigenesis, and the findings suggested that METTL24’s effects on CD4+ and CD8+ T cell infiltration might be involved in its functions in tumors.

**Figure 7:**
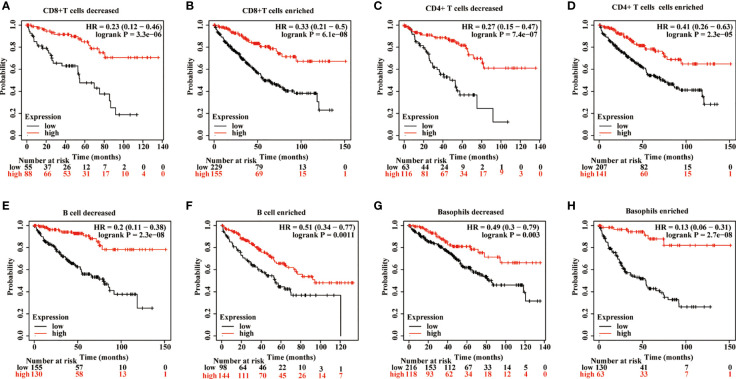
The influence of METTL24 expression on the OS of KIRC patients with high or low infiltration degrees of immune cells. The impact of METTL24 expression on the OS of KIRC patients with enriched or decreased infiltration ratios of **(A, B)** CD8+ T cells, **(C, D)** CD4+ memory T cells, **(E, F)** B cells, **(G, H)** basophils. The survival analysis was performed on Kaplan-Meier plotter database.

The effects of METTL24 expression on the OS of KIRC patients with high or low immune cell infiltration were next explored using data sets from the Kaplan-Meier Plotter database to see if it altered the progression of KIRC *via* controlling the immune microenvironment. The results revealed a difference in METTL24 expression between patients with high immune infiltration and those with low immune infiltration (**Figure 7**, **Supplementary Figure S4**). For example, METTL24 expression had no influence on the OS of patients with enriched B cells (p = 0.0011), but had a substantial effect on patients with depleted B cells (p = 2.3e-08). Similar results were seen in basophils and NKT cells. As a result of the previous findings, it appears that METTL24 regulates carcinogenesis by influencing the immune system.

### Multi-omics-based analyses identified CTCF and EP300 as the potential transcription factors of METTL24 in KIRC

**Figure 8:**
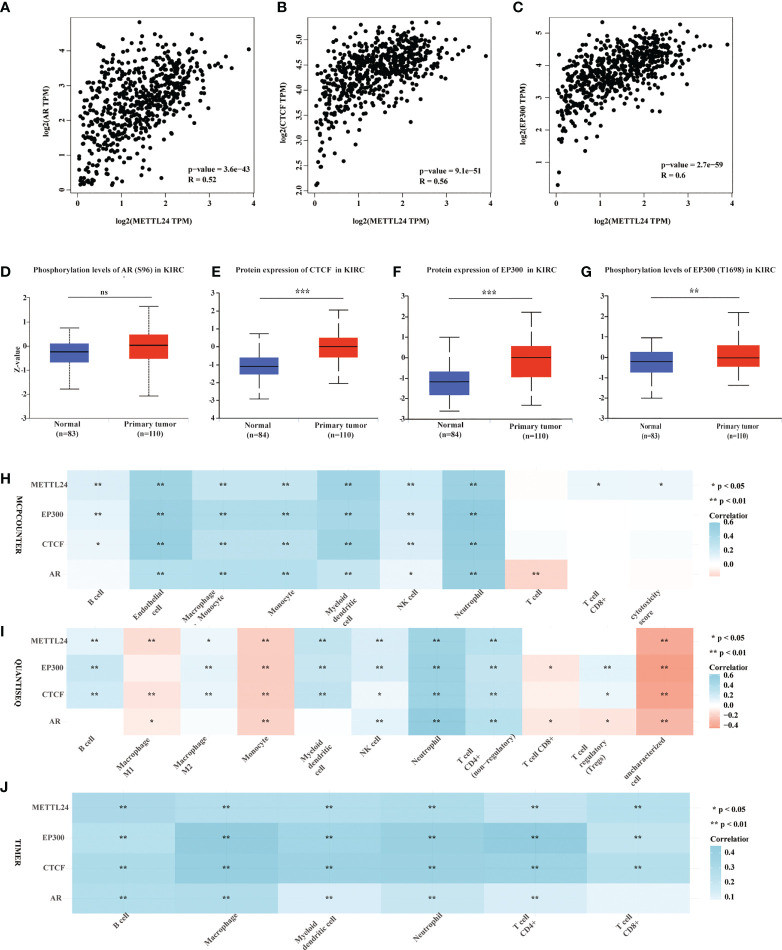
Protein levels and phosphorylation content of EP300, CTCF and AR, the possible METTL24 transcription factors, were altered in KIRC tissues compared to normal kidney tissues. The Spearman correlation between METTL24 expression and **(A)** AR, **(B)** CTCF, and **(C)** EP300 expression in KIRC. The phosphorylation levels of **(D)** AR and **(E)** EP300 in KIRC and normal kidney tissues. The protein expression levels of **(F)** CTCF and **(G)** EP300 in KIRC versus normal kidney tissues. The correlation between METTL24, EP300, CTCF, AR expression and the infiltration degrees of distinct immune cells in KIRC as analyzed using **(H)** MCPCOUNTER, **(I)** QUANTISEQ, and **(J)** TIMER algorithms. ***p < 0.001,**p < 0.01 and *p < 0.05; ns, not significant.

Gene expression is known to be influenced in part by transcription factors. To better understand the reasons for METTL24’s downregulation in KIRC, we performed a multi-omics investigation to look for putative METTL24 upstream transcription factors. To begin, we used the hTFtarget database identifying seven probable transcription factors of METTL24 found in kidney tissue by ChIP sequencing, including AR, BRD2, CTCF, EP300, MYC, SPI1, and ZNF76. Then, the co-expression relationship between METTL24 and these genes was investigated, and we discovered that AR, CTCF, and EP300 were all positively linked with METTL24 (**Supplementary Table S2**). Furthermore, Spearman correlation analysis revealed that AR, CTCF, and EP300 gene expression were substantially linked with METTL24 expression, with correlation coefficients of 0.52, 0.56, and 0.60, respectively (**Figures 8A–C**). The protein expression and phosphorylation levels of AR, CTCF, and EP300 in KIRC tissues were next studied, and it was observed that the protein contents of EP300 and CTCF were significantly up-regulated in KIRC tissues compared to normal kidney tissues, as were the phosphorylation levels of AR at Ser96 and EP300 at Thr1698 (**Figures 8D–G**). Since METTL24 potentially regulated the immune microenvironment, we hypothesized that transcription factors upstream of METTL24 should also be involved in immune regulation. Therefore, we investigated the correlation between CTCF and EP300 expression and the infiltration ratios of various immune cells in KIRC using distinct algorithms. The result showed that the expression of CTCF and EP300 were significantly correlated with the infiltration levels of multiple immune cells, and METTL24’s trend was also present in the link between CTCF and EP300 and the majority of immune cells (**Figures 8H-J**). This result suggested that CTCF and EP300 might also play a role in immunomodulation of KIRC tumorigenesis as METTL24.

## Discussion

KIRC is the most frequent kind of kidney cancer, accounting for more than 70% of all occurrences (24). In the clinic, approximately 15% of KIRC patients have metastatic tumors at the time of diagnosis, and only a few patients benefit from targeted therapy, cytokine therapy, and ICB (25–27). Prognostic biomarkers will aid in the identification of high-risk and low-risk individuals in order to develop a tailored treatment plan. There have been no clinically employed molecular prognostic biomarkers till recently. This study revealed that METTL24 expression was significantly decreased in KIRC tissues compared to normal adjacent tissues and identified METTL24 as one potential independent prognostic biomarker for KIRC. As a result, METTL24 might be employed as a prognostic factor in renal carcinoma clinical diagnosis and treatment.

The immune microenvironment, which consists of tumor cells, immune cells, endothelial cells, cytokines, and other factors, has a significant impact on tumor initiation and progression (28, 29). Because renal carcinoma is not inherently susceptible to radiotherapy or chemotherapy; Cytokines, such as interleukin and interferon, were mostly utilized for treatment in the 1990s, but the objective response rate is only 5% to 27%, and the median PFS is only 3-5 months (30–32). Although the median survival of kidney cancer patients has increased significantly as a result of ICB treatment, most advanced renal carcinoma patients still do not benefit from the treatment in the long run (33, 34). In this study, we elucidated that METTL24 expression correlated with the infiltration levels of various immune cells, and the expression of immune checkpoints, cytokines, chemokines, and chemokine receptors in KIRC, suggesting that METTL24 might be used as an immune target combined with the typical targets in KIRC.

In eukaryotic organisms, the METTL family usually encodes functional proteins that operate as methyltransferases. Previous research has identified METTL3 and METTL14 are m6A methylation writers on mRNAs, which regulate the growth and metastasis of renal cancer, colorectal cancer, pancreatic cancer, and other cancers (11, 14, 35, 36). Despite this, there are few publications on the role of METTL24 in carcinogenesis. According to Guoying’s research, elevated METTL24 expression is associated with a worse prognosis in individuals with rectal cancer (HR = 2.1) (15). In our study, we discovered that the expression of METTL24 and numerous immune genes were highly correlated (**Figure 6**). Meanwhile, we found that METTL24 was potentially associated with NF-κB pathway, according to the GSEA analysis (**Figure 5D**). As is well-known, NF-κB signaling pathway is one of indispensable pathways for immune microenvironment regulation (37–39). For example, NF-κB pathway has been reported to maintain the activity of tumor-associated macrophages in tumor progression. Therefore, we speculated that the correlation between METTL24 and the immune genes might be mediated by NF-κB pathway. We discovered that METTL24 expression affects the survival rate of KIRC patients and that it might play a function in controlling the immune microenvironment in this study. The immunomodulatory role of METTL24 has been discovered for the first time, to our knowledge.

In conclusion, this work found lower levels of METTL24 expression in KIRC tissues compared to normal tissues, discovered METTL24’s prognostic relevance in KIRC, and established METTL24’s possible activities in the immune microenvironment. As a result, METTL24 might be used as a prognostic marker in KIRC as well as an immune target in the clinic.

## Data availability statement

The original contributions presented in the study are included in the article/**Supplementary Material**. Further inquiries can be directed to the corresponding authors.

## Ethics statement 

The studies involving human participants were reviewed and approved by the Ethics Committee of Shanghai Xinchao Biotechnology Co., Ltd (NO. YB-M-05-02). Written informed consent for participation was not required for this study in accordance with the national legislation and the institutional requirements.

## Author contributions

Study concept and design: WZ, ZJ, YX, and YD. Experiment and data acquisition: WZ, ZZ, DT, CL, WC, and YC. Analysis and interpretation of data: ZJ, WZ, YL, and QJ. Statistical analysis: ZJ and WZ. Drafting of the manuscript: ZJ, WZ, and CL. Funding: WZ, LY, and YD. Study supervision: XZ, XL, YX, and YD. All authors contributed to the article and approved the submitted version.

## Funding

This study was supported by the National Natural Science Foundation of China (Grant No. 82003172), Shenzhen Fund for Guangdong Provincial High-level Clinical Key Specialties (No. SZGSP001), the Postdoctoral Science Foundation of China (No. 2020M673065) and Guangzhou Development Zone entrepreneurship leading talent project (No: 2017-L153).

## Acknowledgments

Thanks to Shanghai Outdo Biotech Company for donating commercial tissue chip.

## Conflict of interest

The authors declare that the research was conducted in the absence of any commercial or financial relationships that could be construed as a potential conflict of interest.

## Publisher’s note

All claims expressed in this article are solely those of the authors and do not necessarily represent those of their affiliated organizations, or those of the publisher, the editors and the reviewers. Any product that may be evaluated in this article, or claim that may be made by its manufacturer, is not guaranteed or endorsed by the publisher.
