# Supplementary figures and images for: A comprehensive investigation discovered the novel methyltransferase METTL24 as one presumably prognostic gene for kidney renal clear cell carcinoma potentially modulating tumor immune microenvironment
Source: Front Immunol. 2022 Oct 14;13:926461. doi: 10.3389/fimmu.2022.926461 (PMC9613963; doi:10.3389/fimmu.2022.926461)

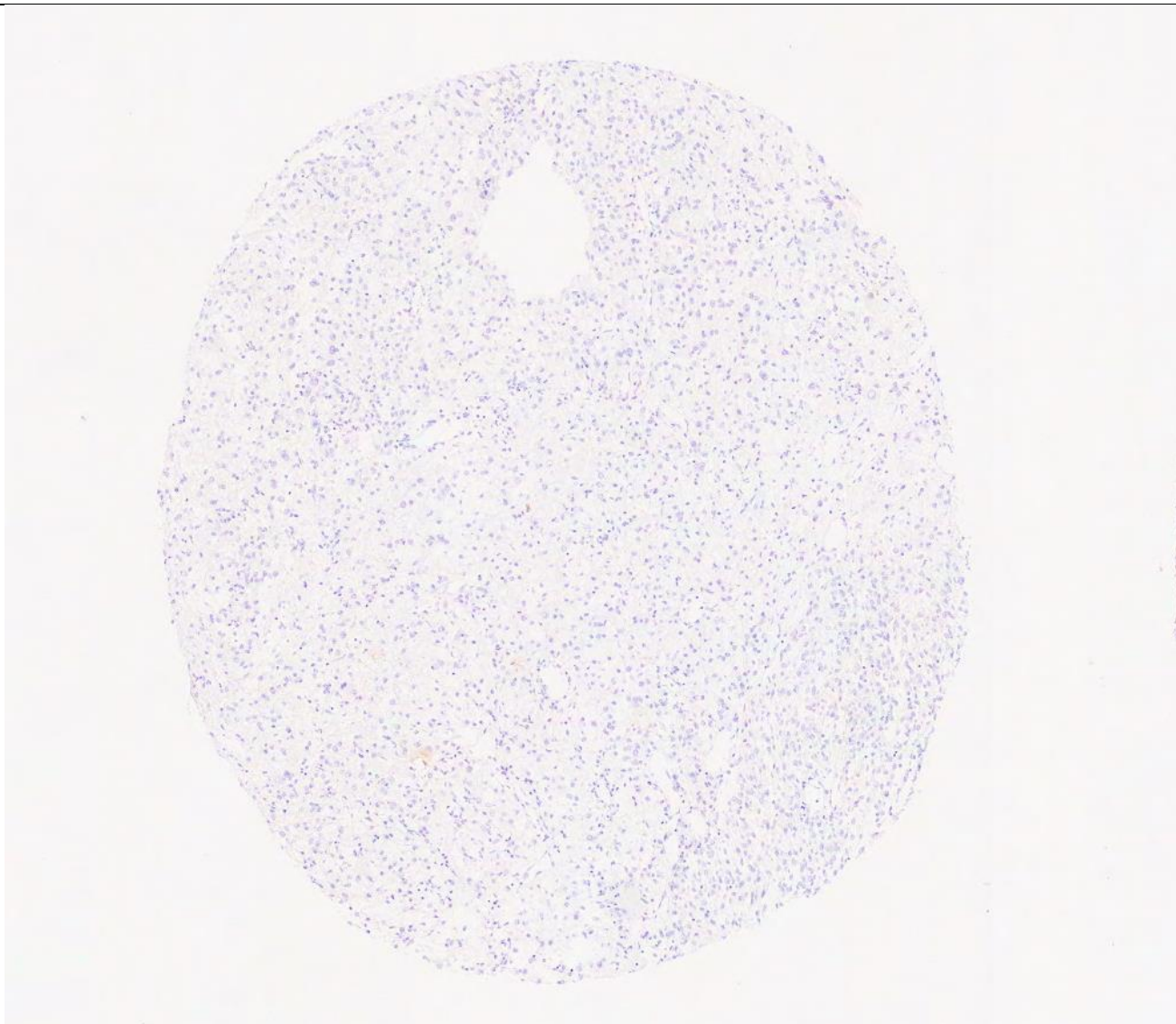

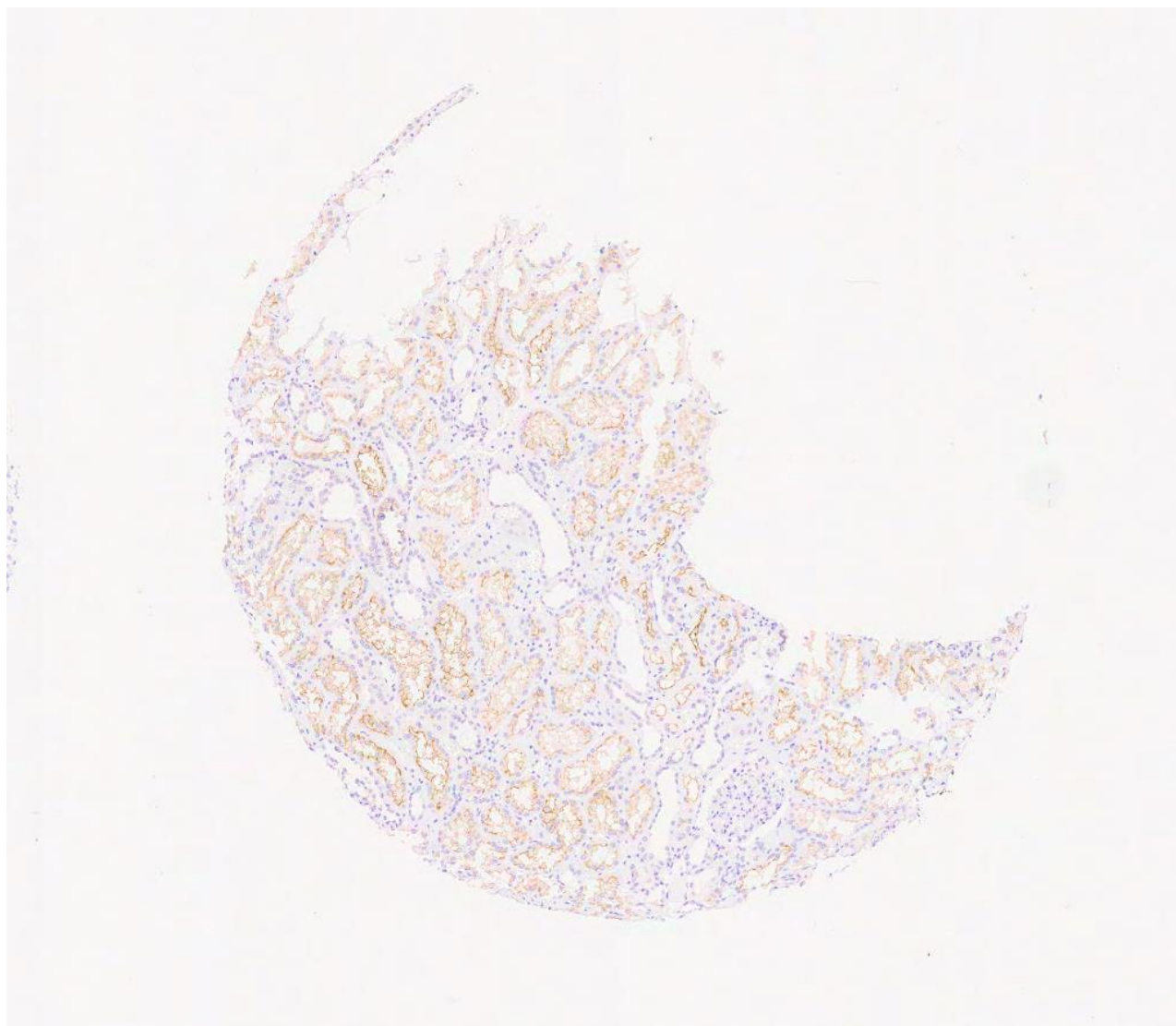

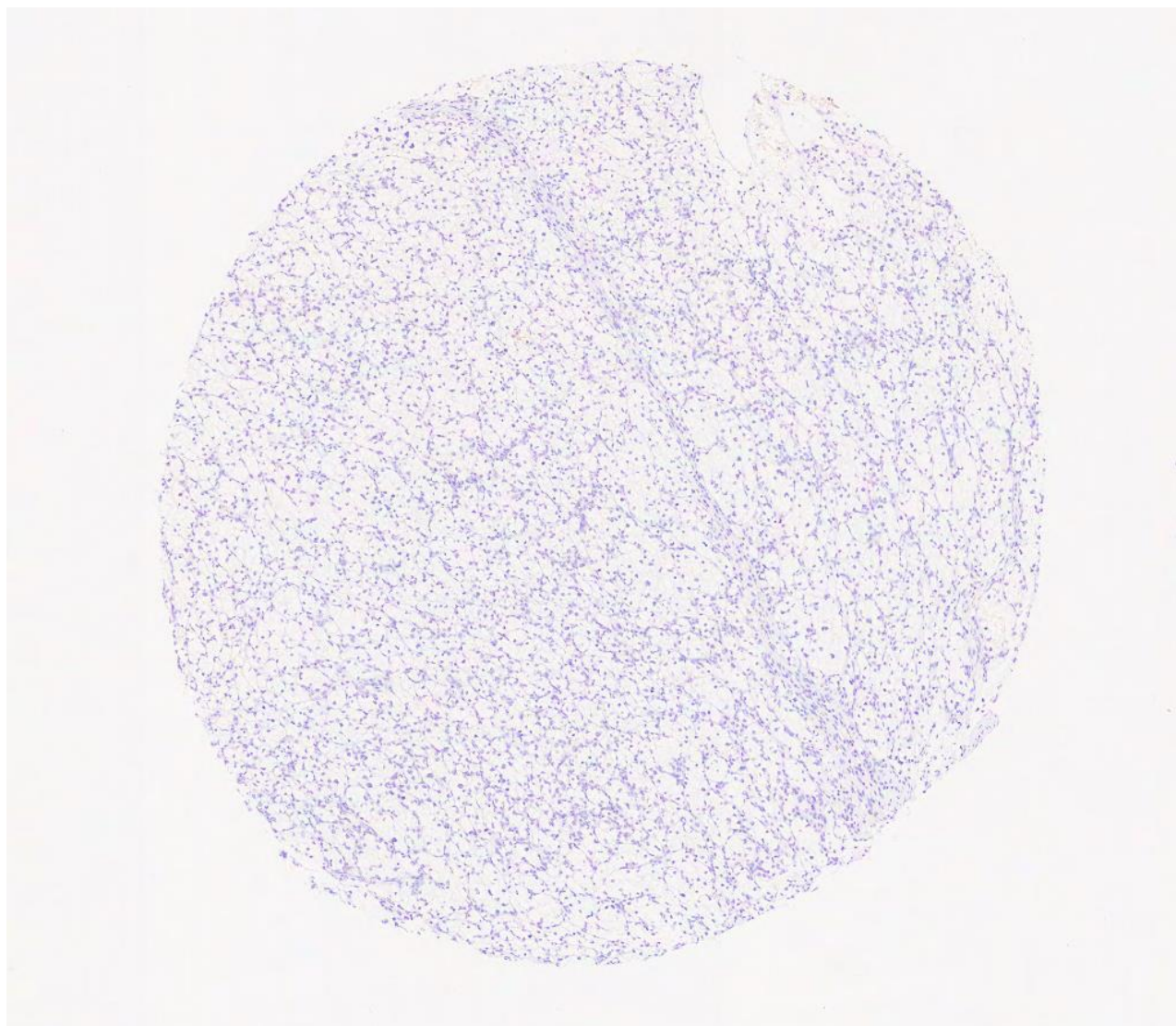

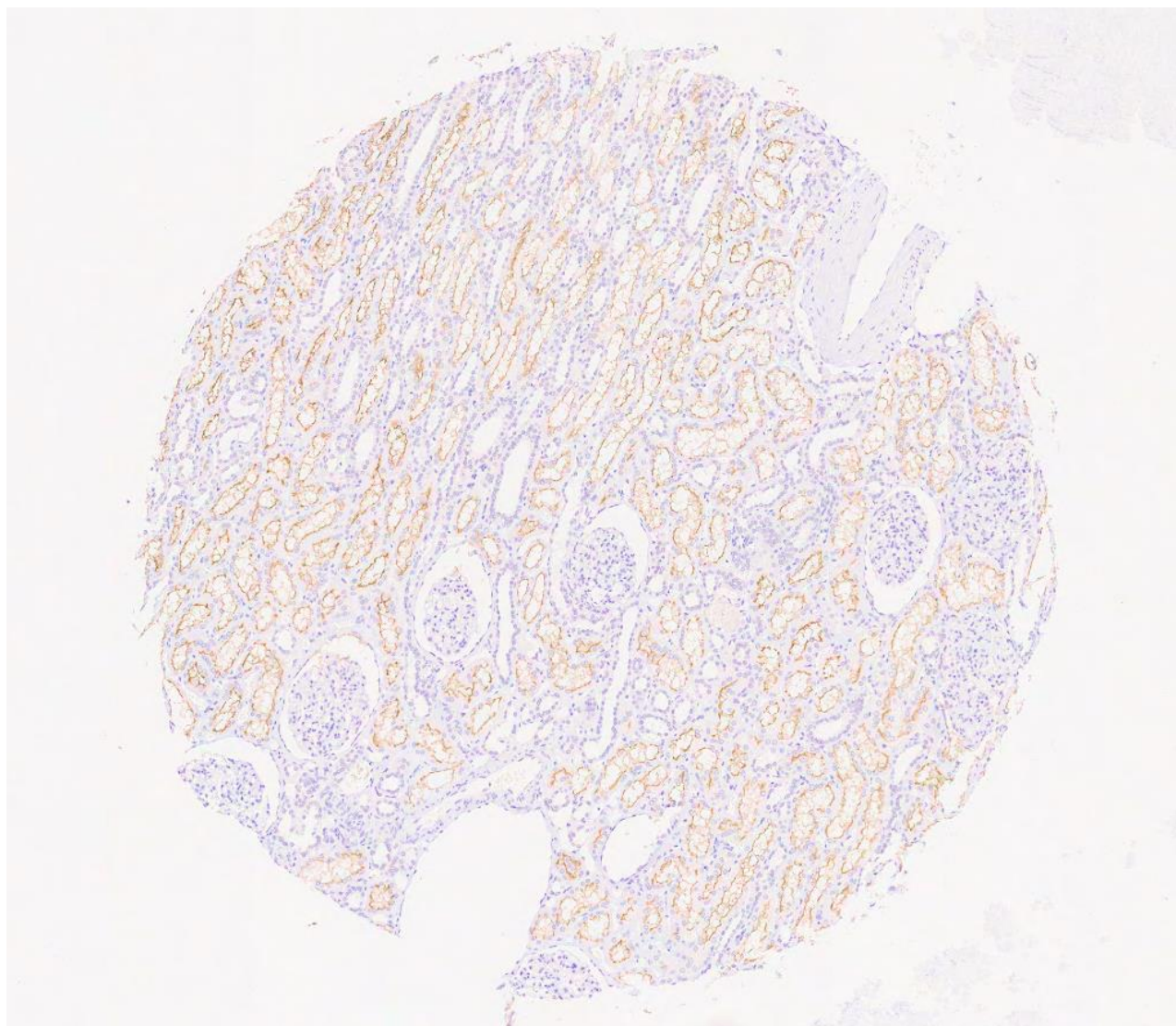

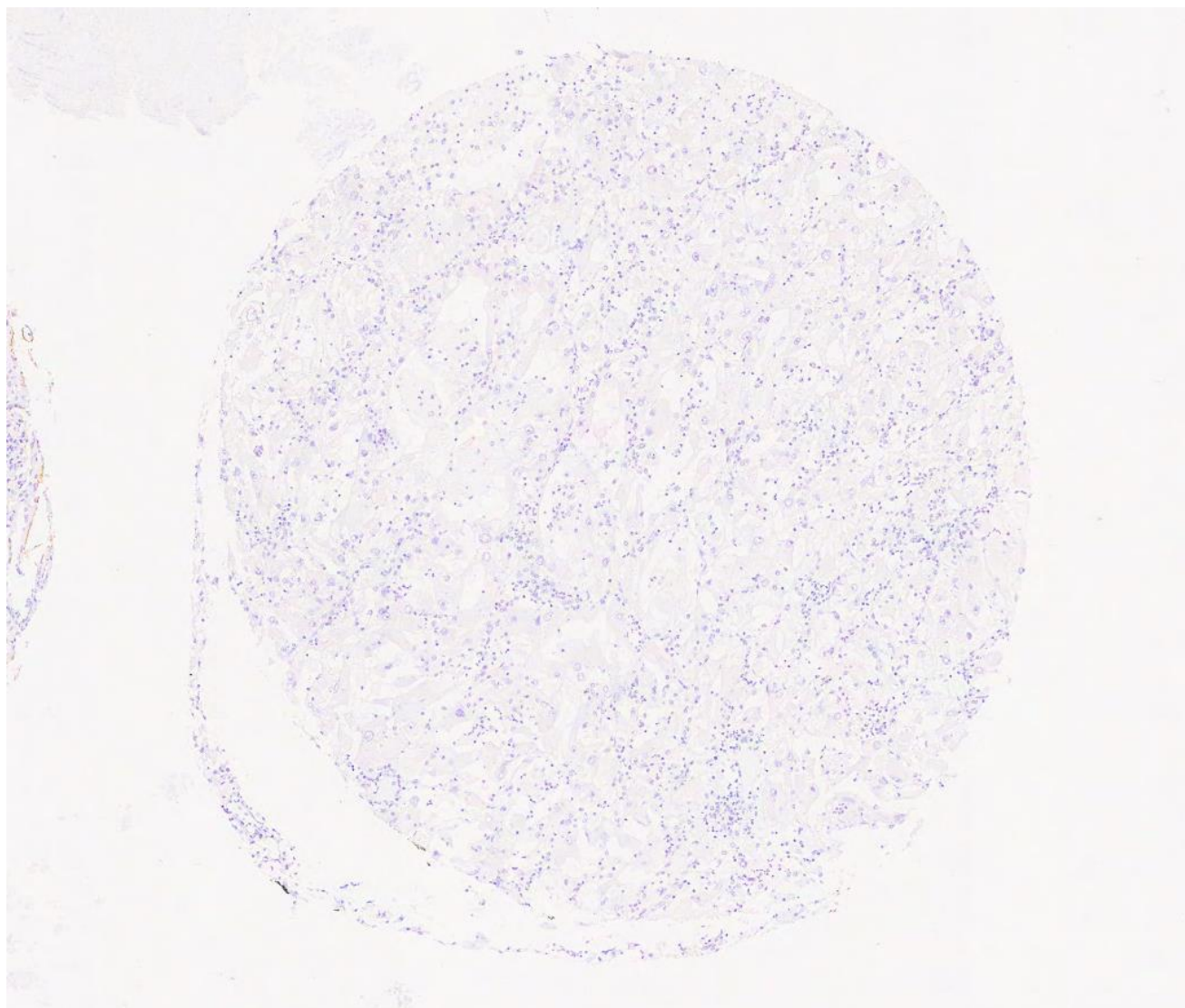

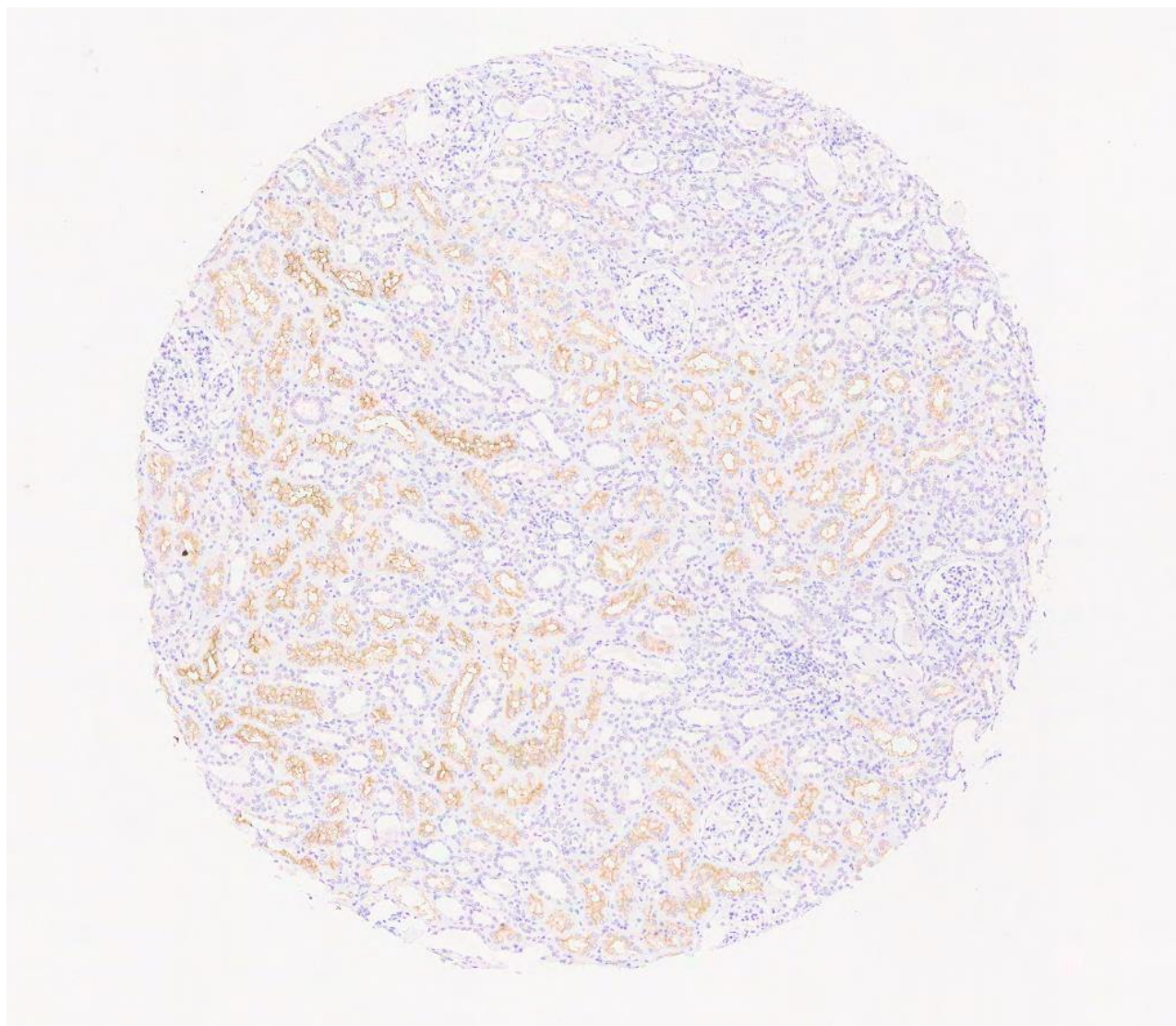

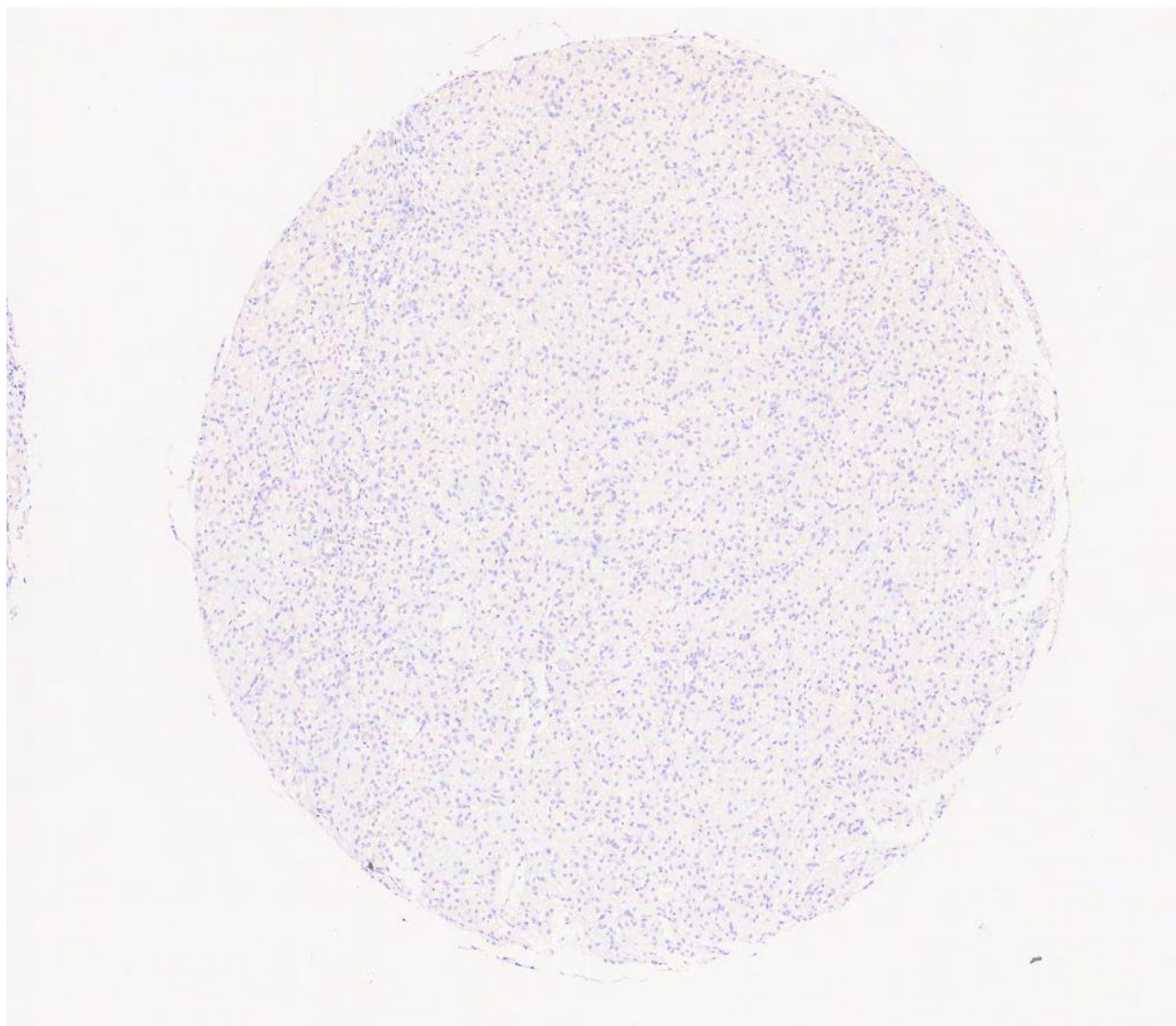

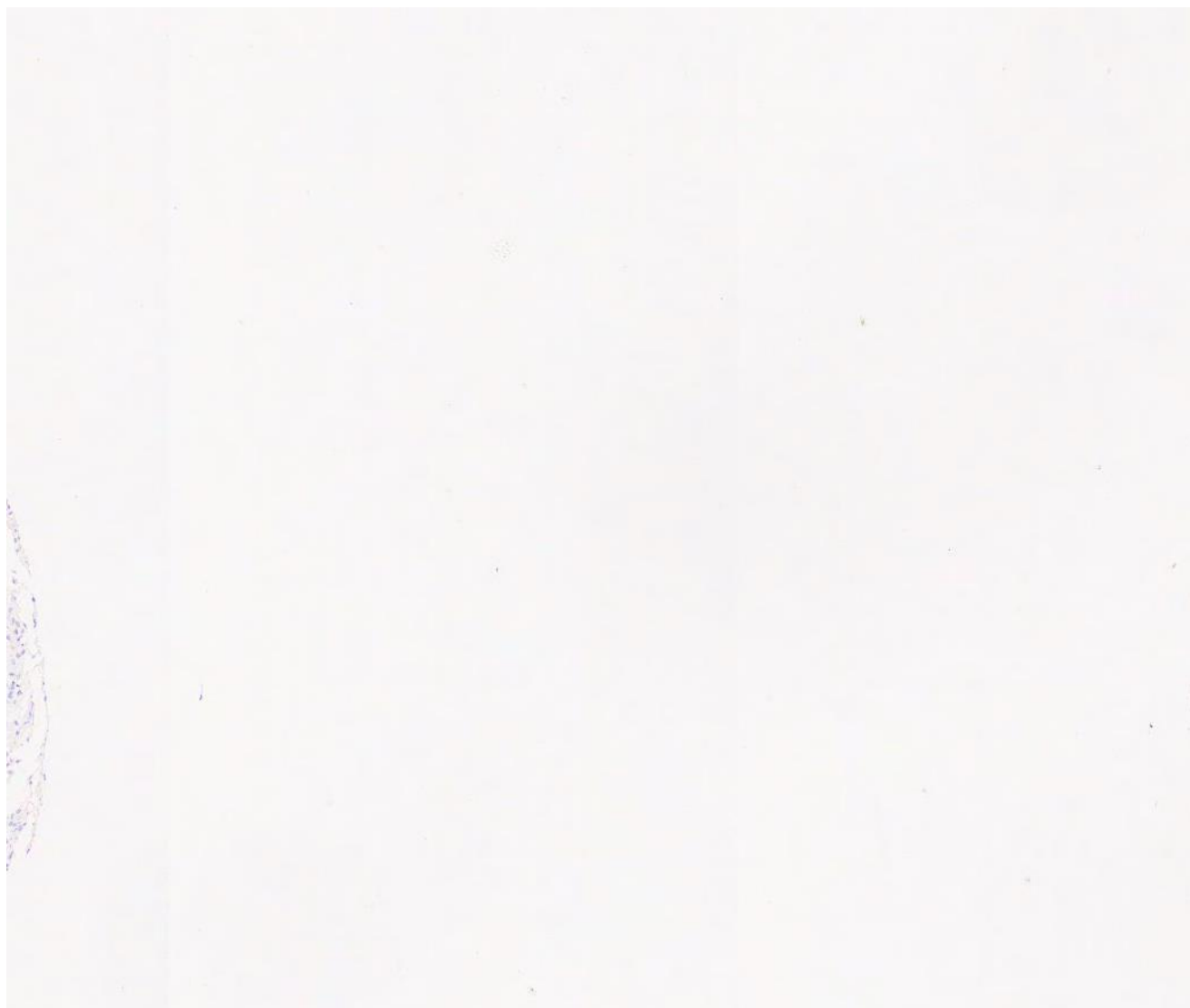

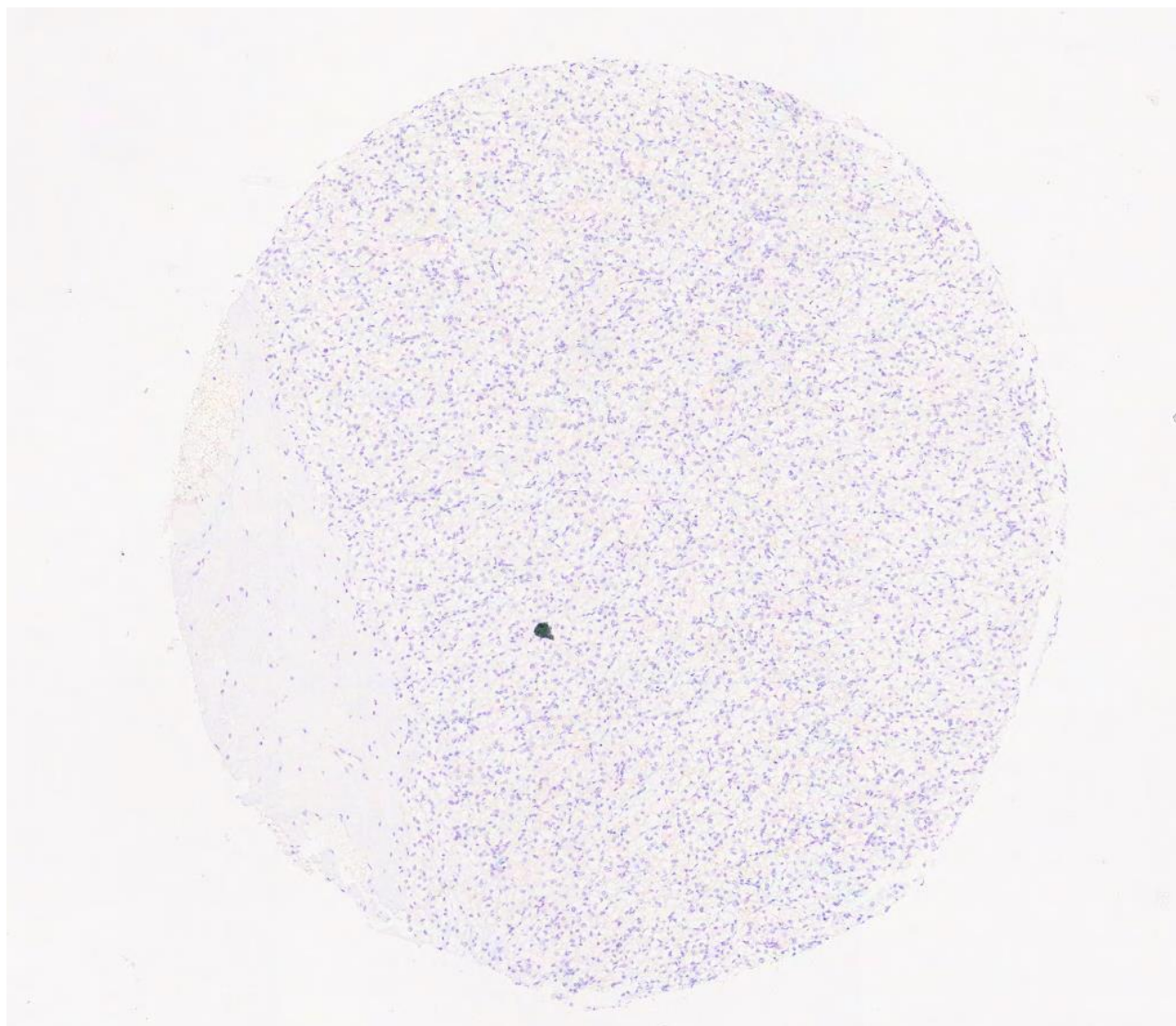

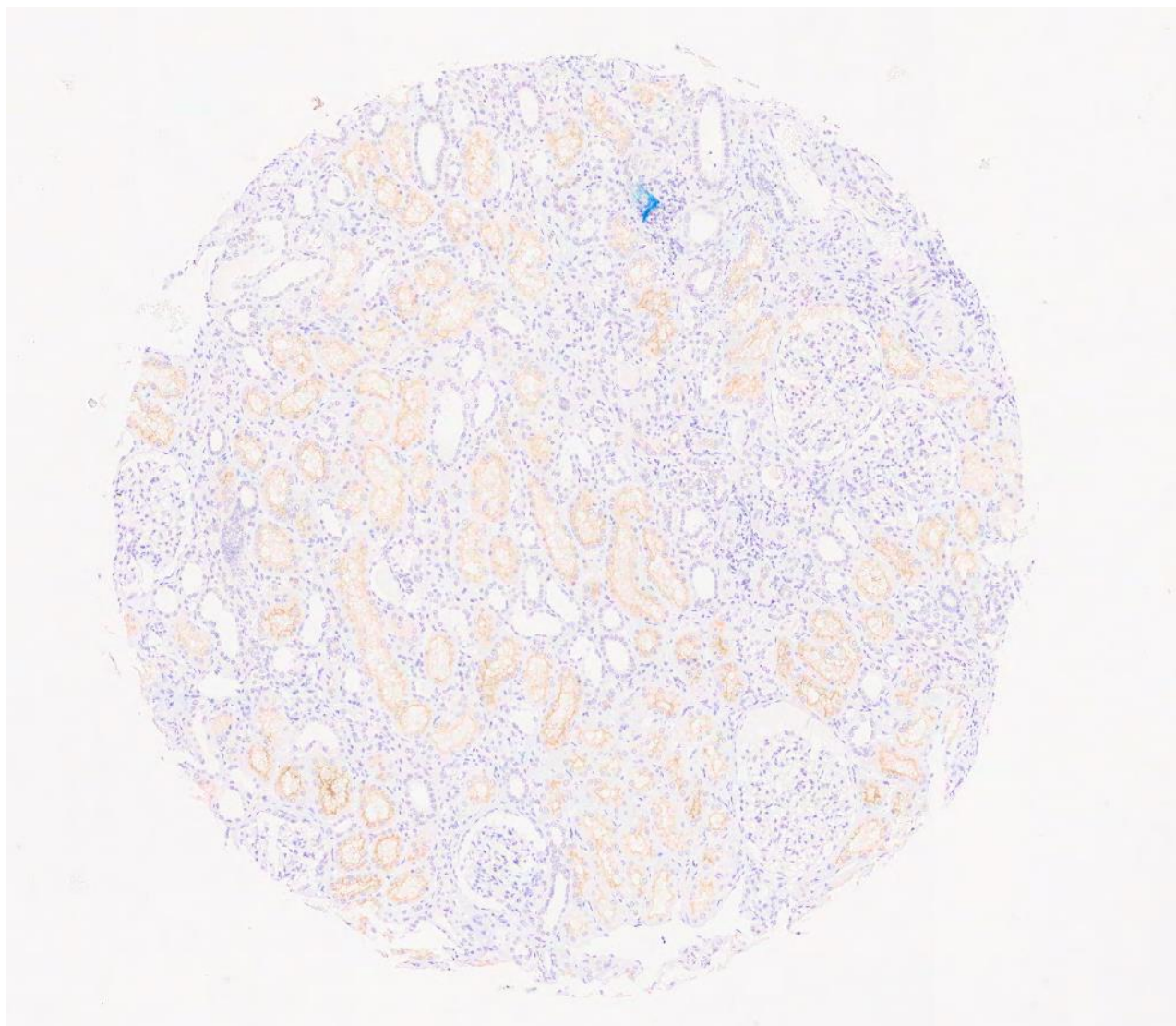

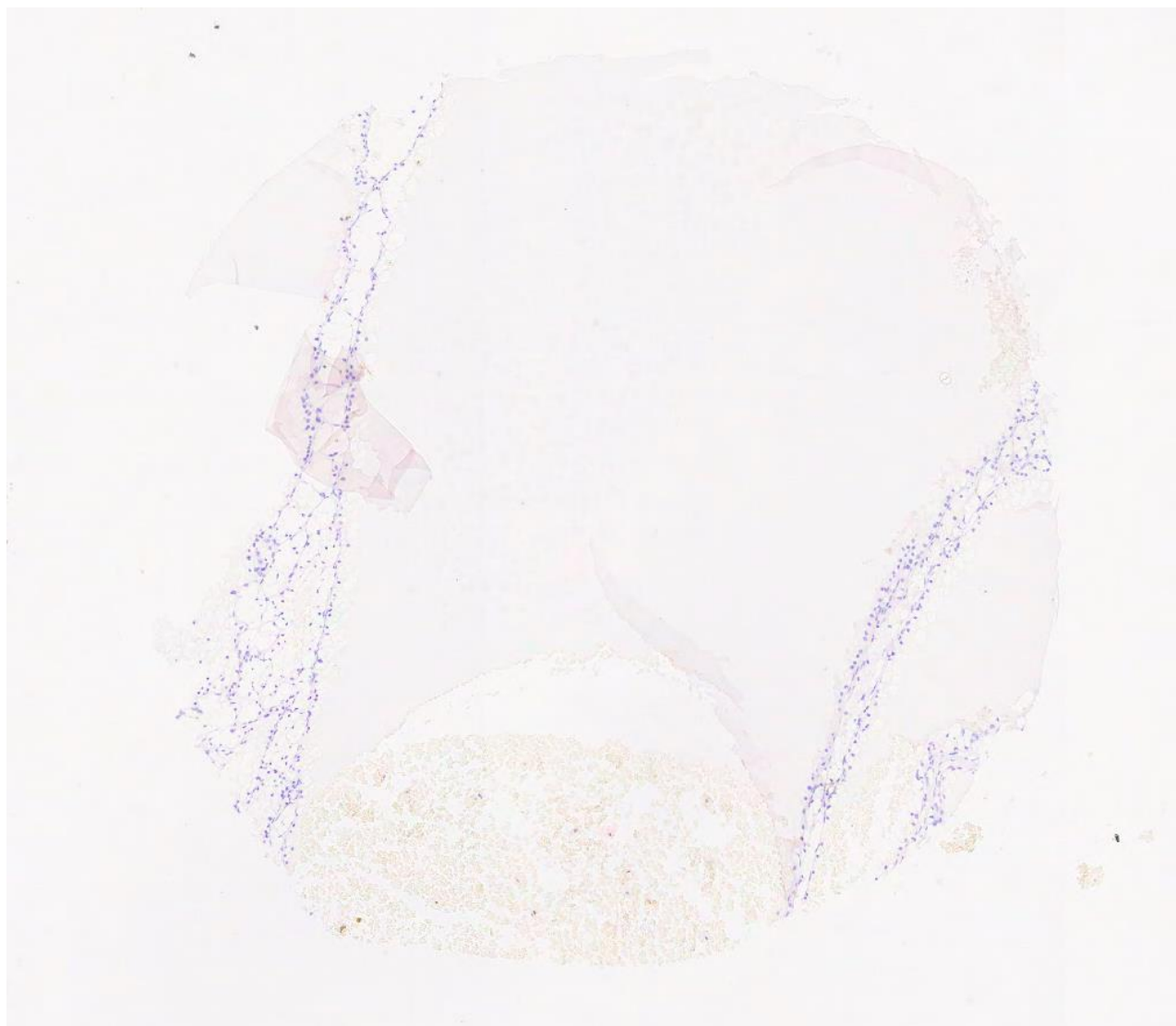

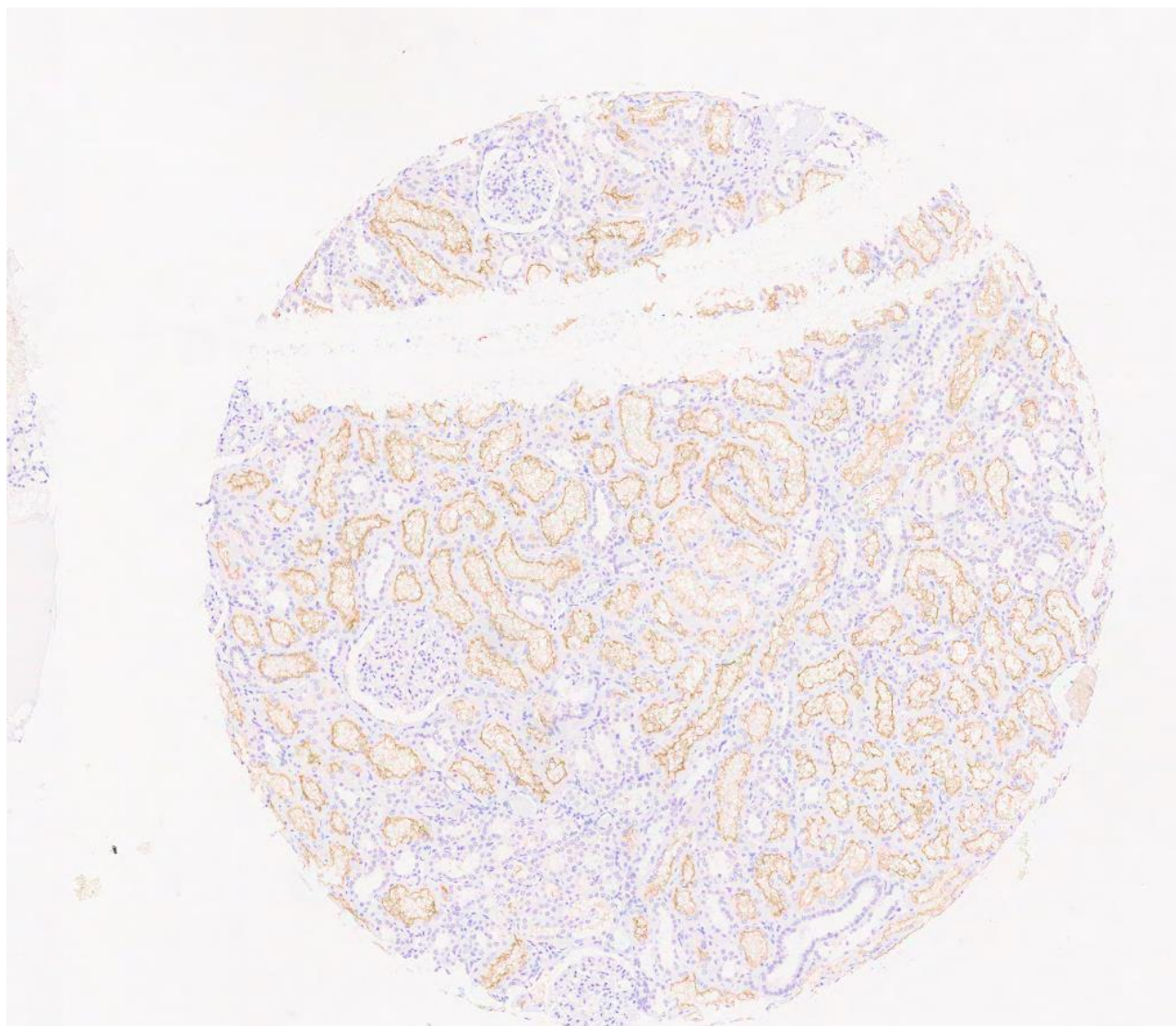

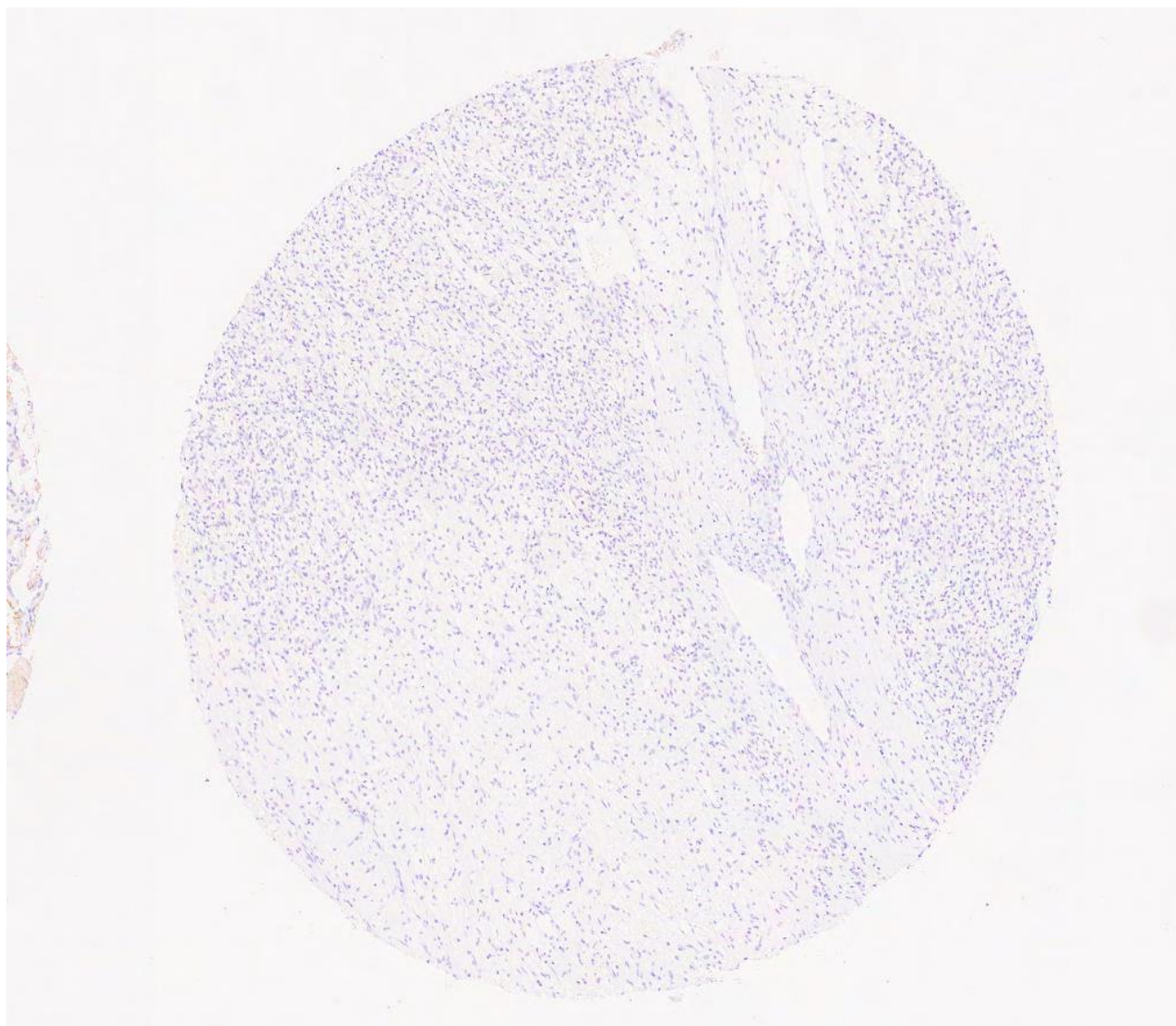

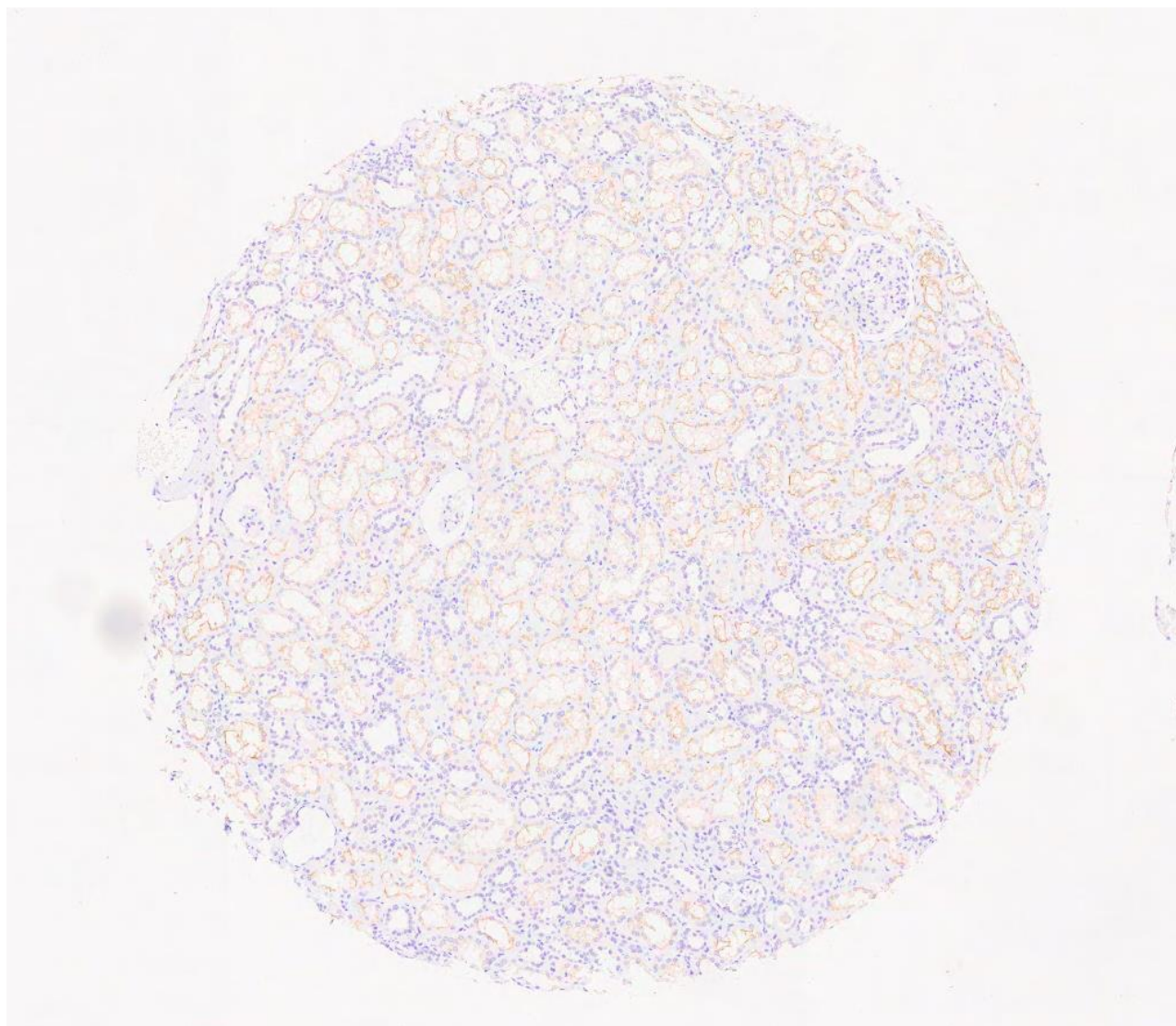

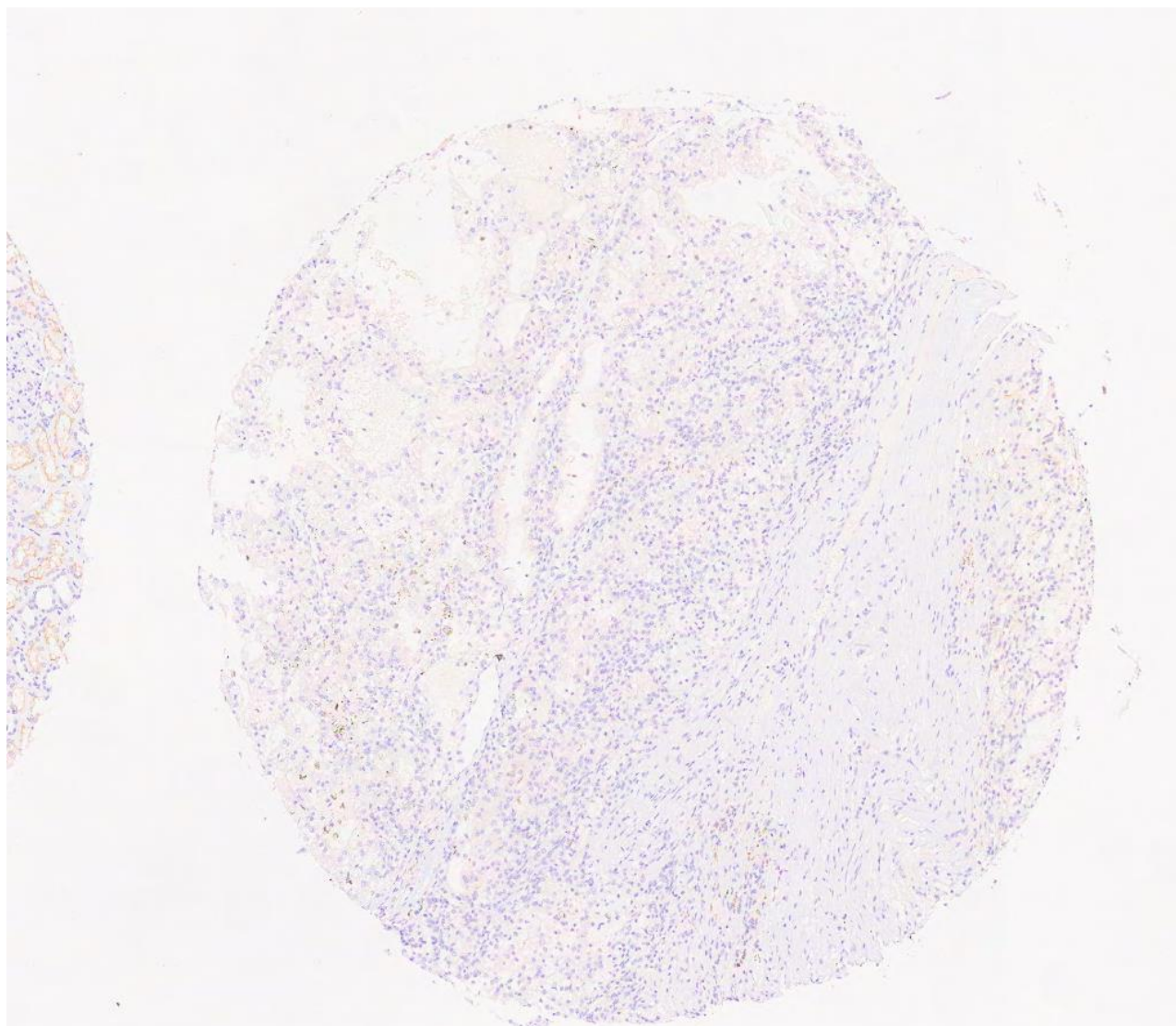

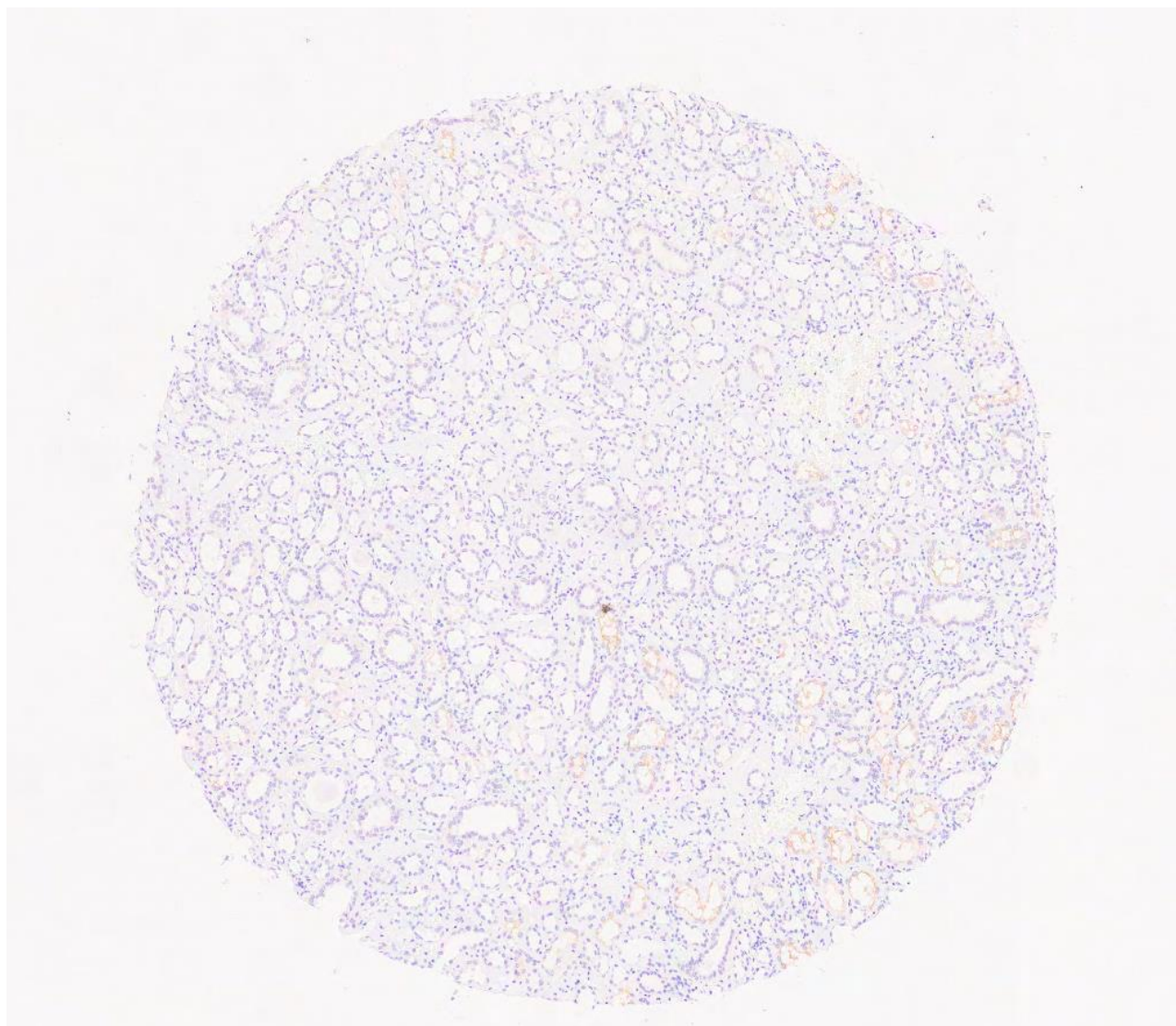

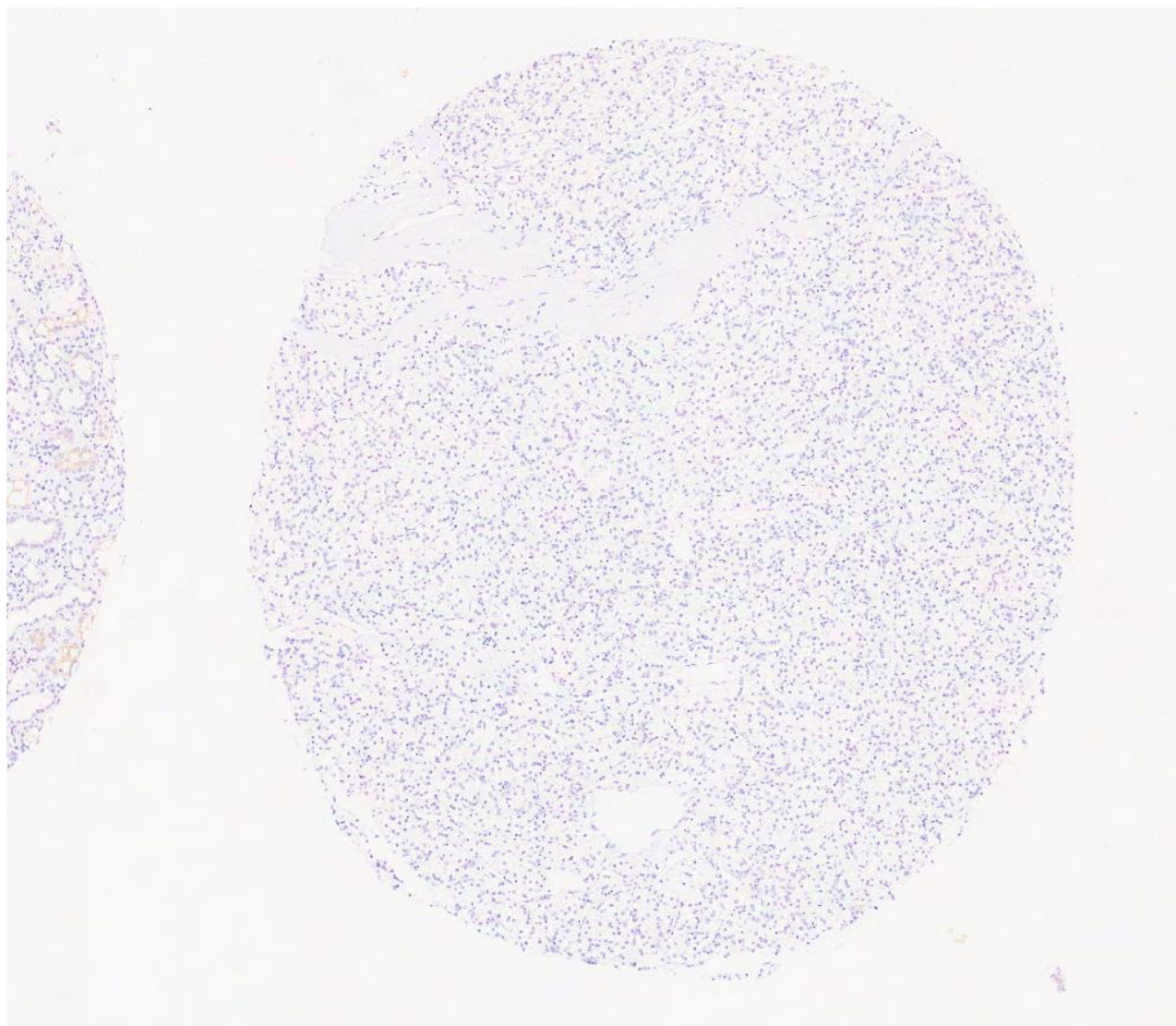

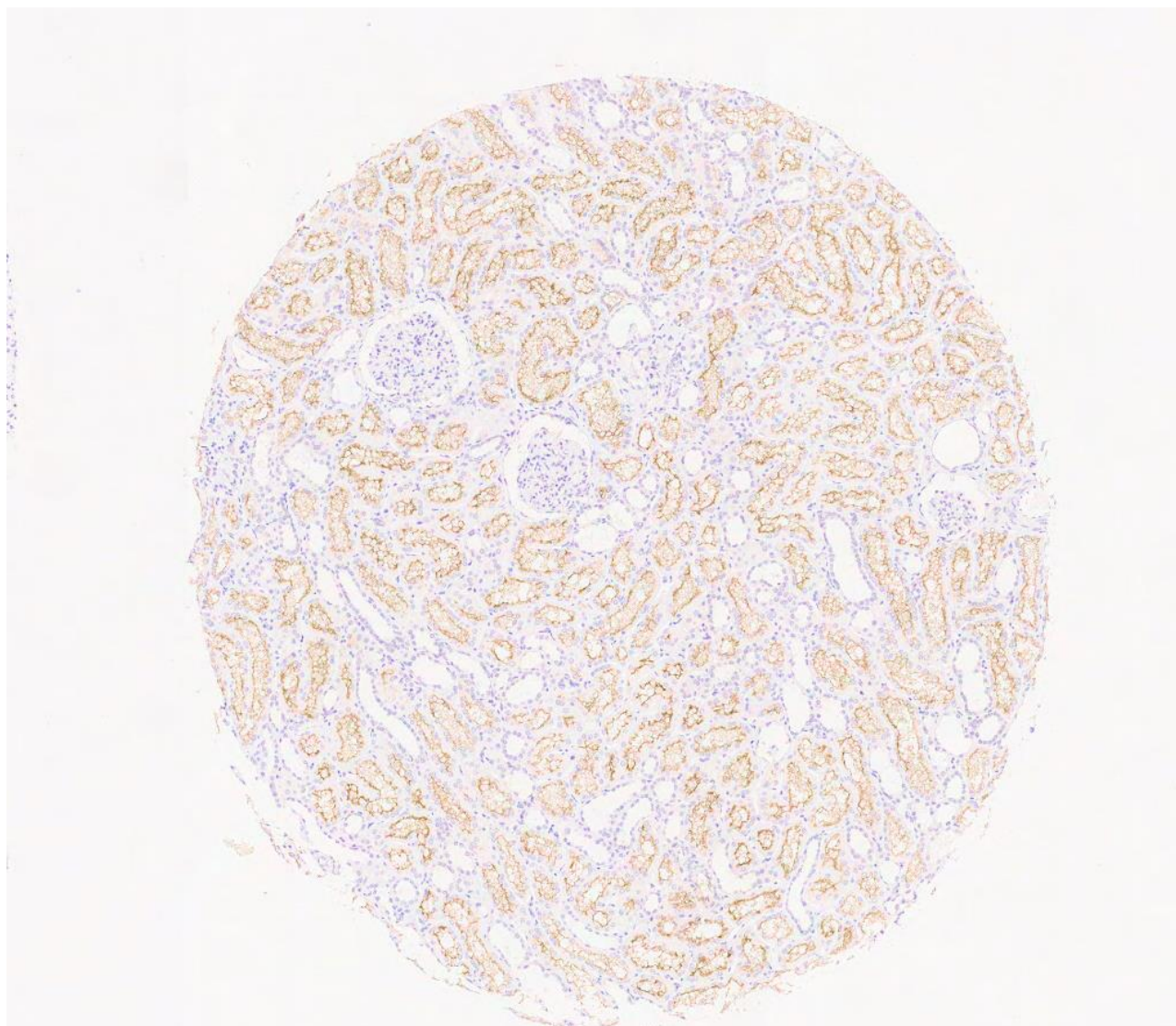

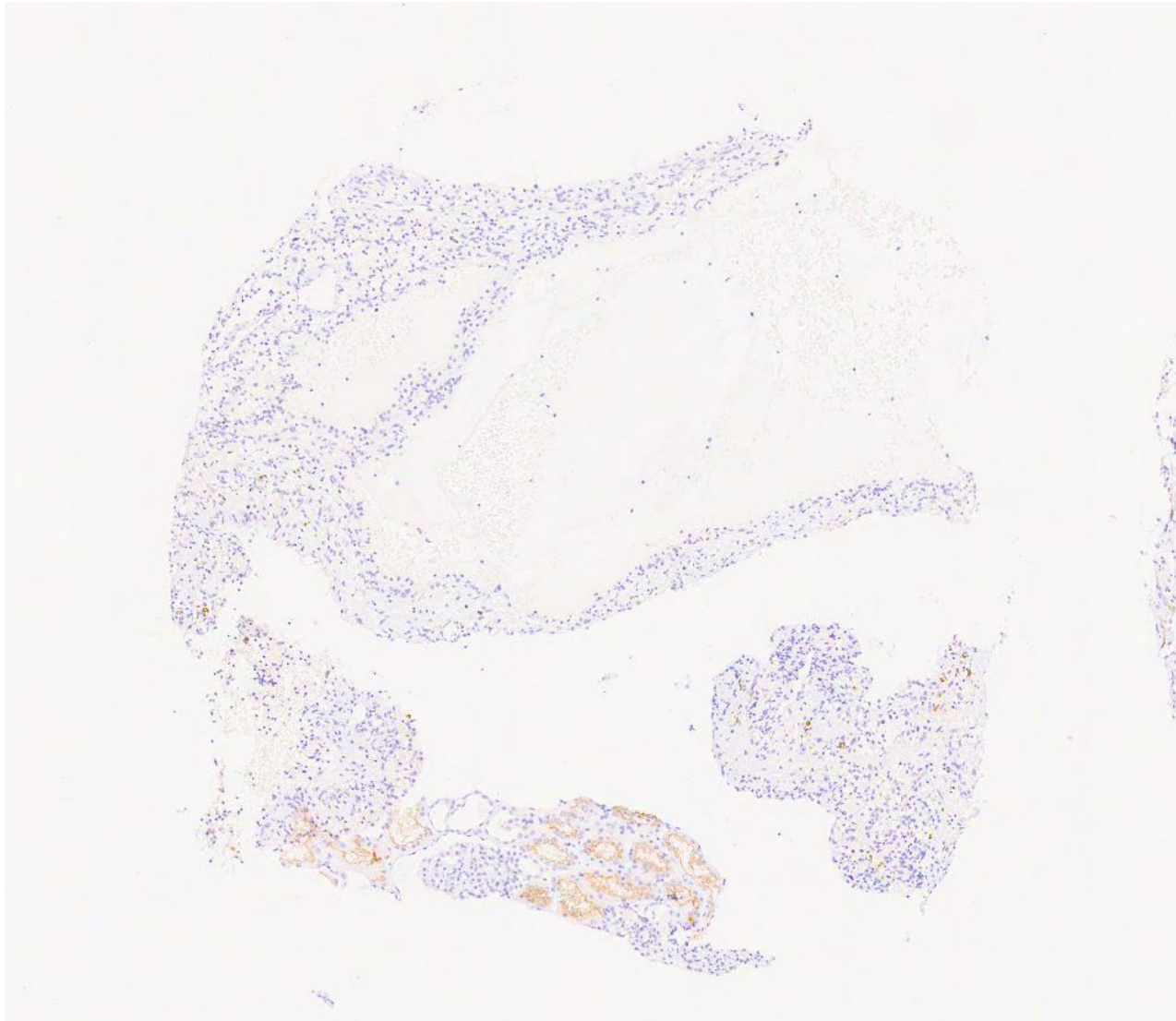

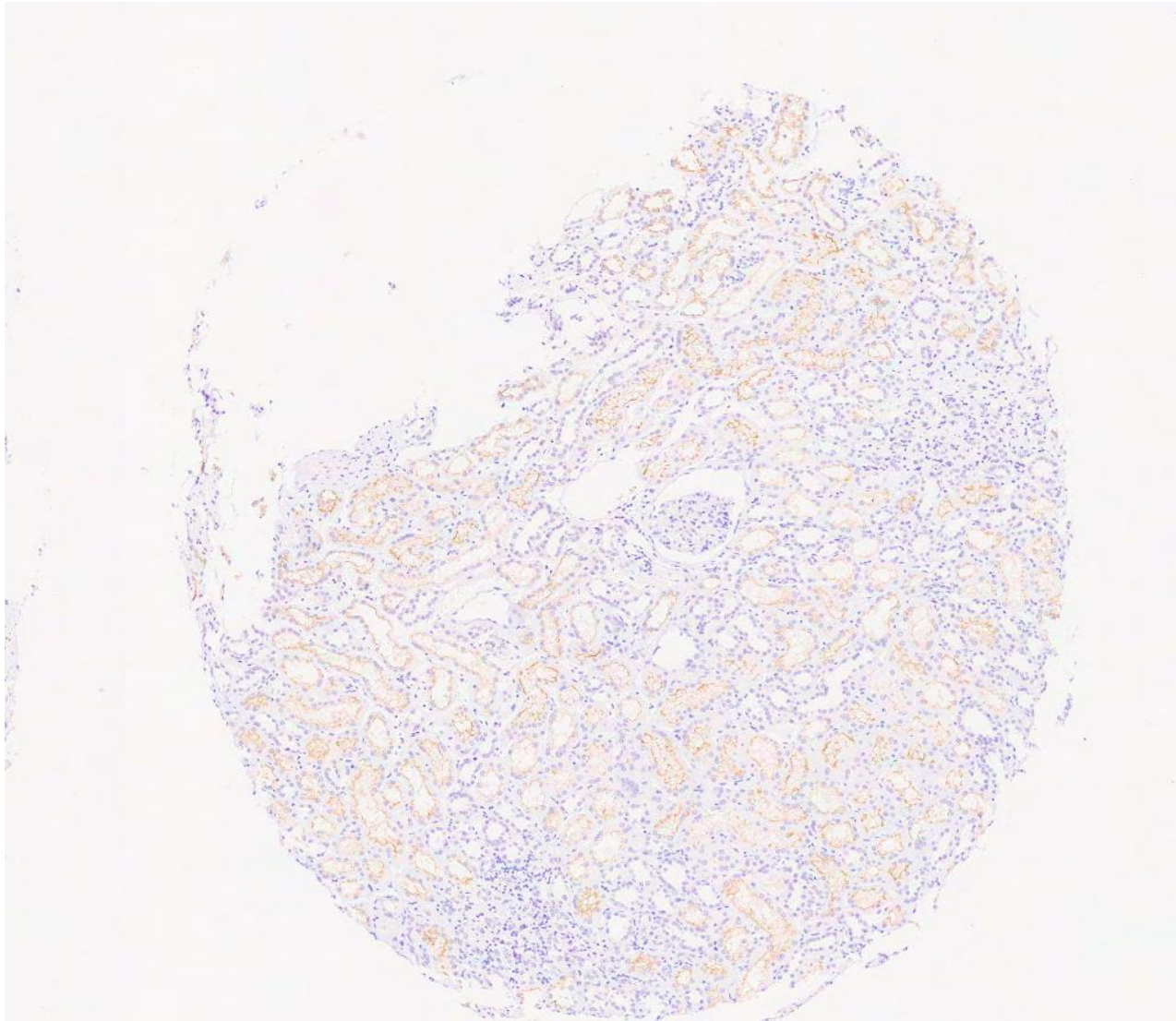

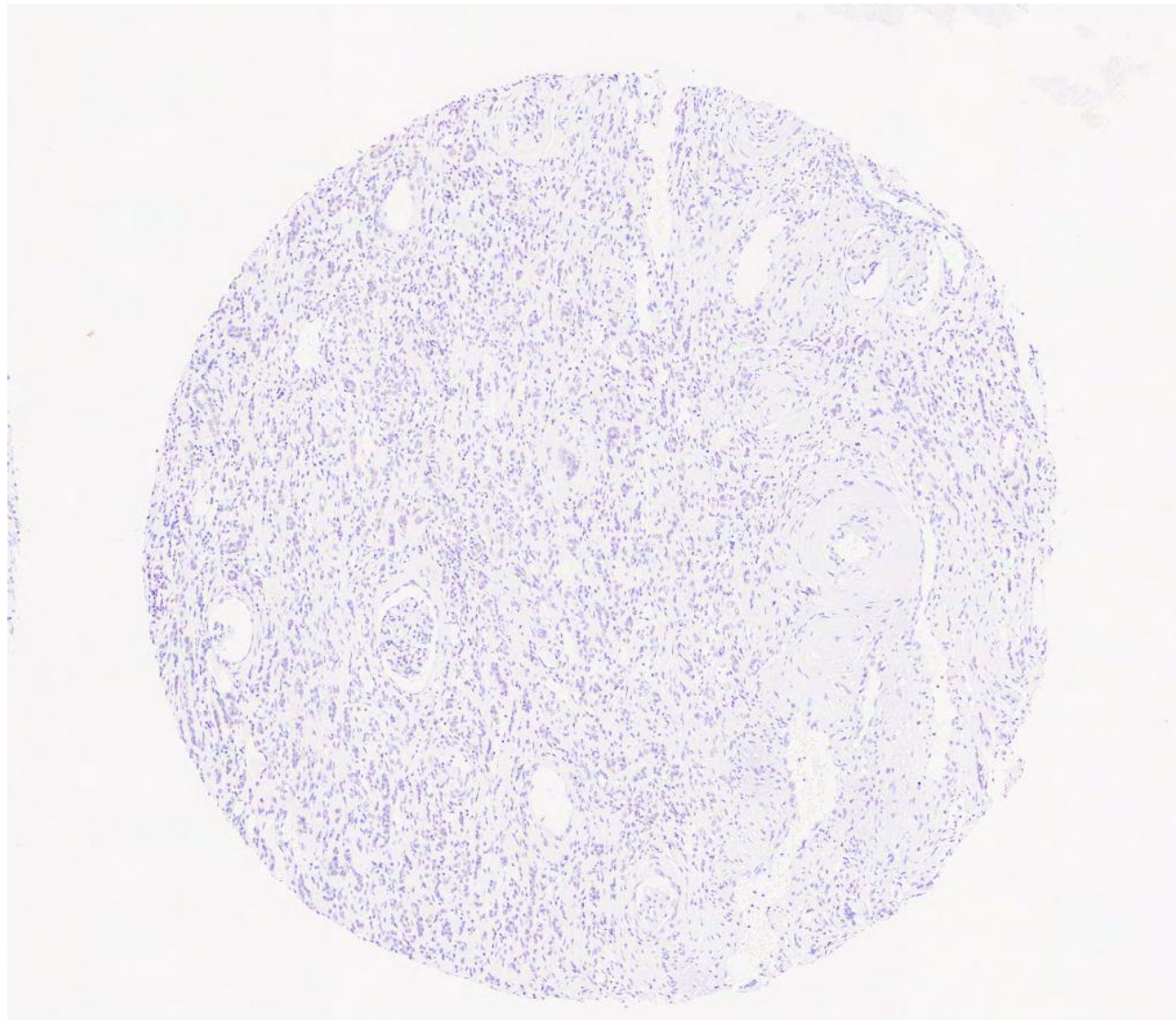

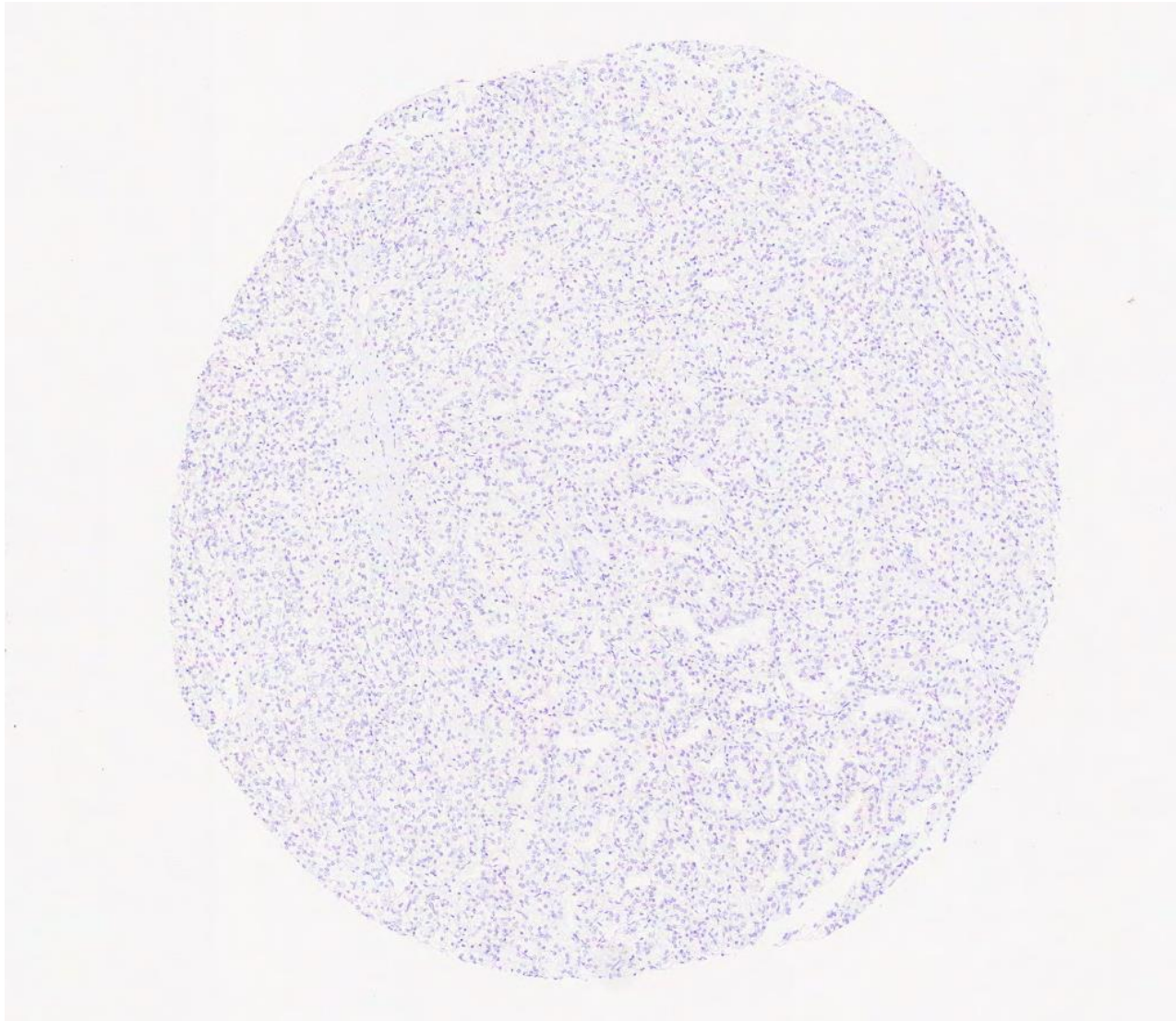

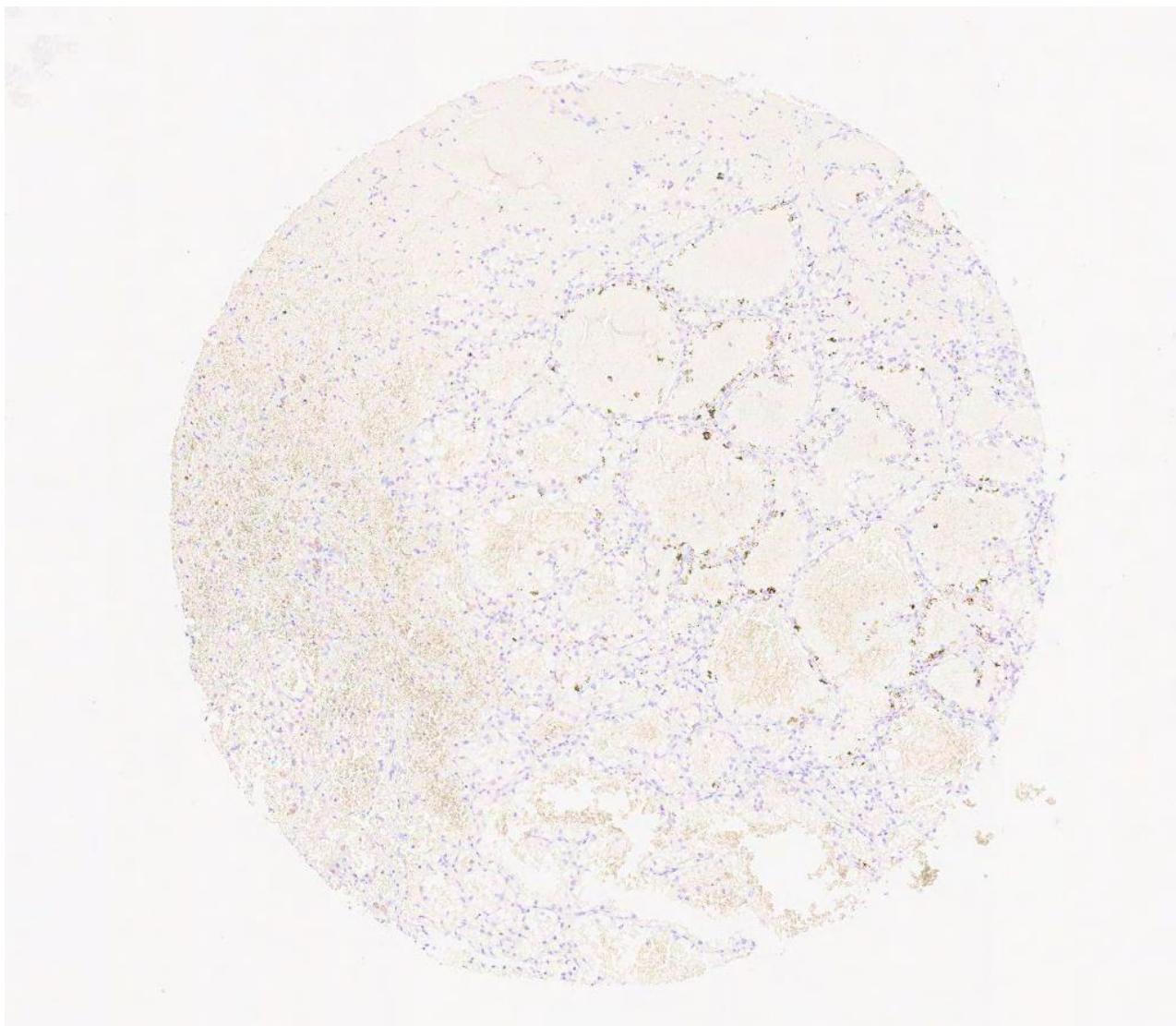

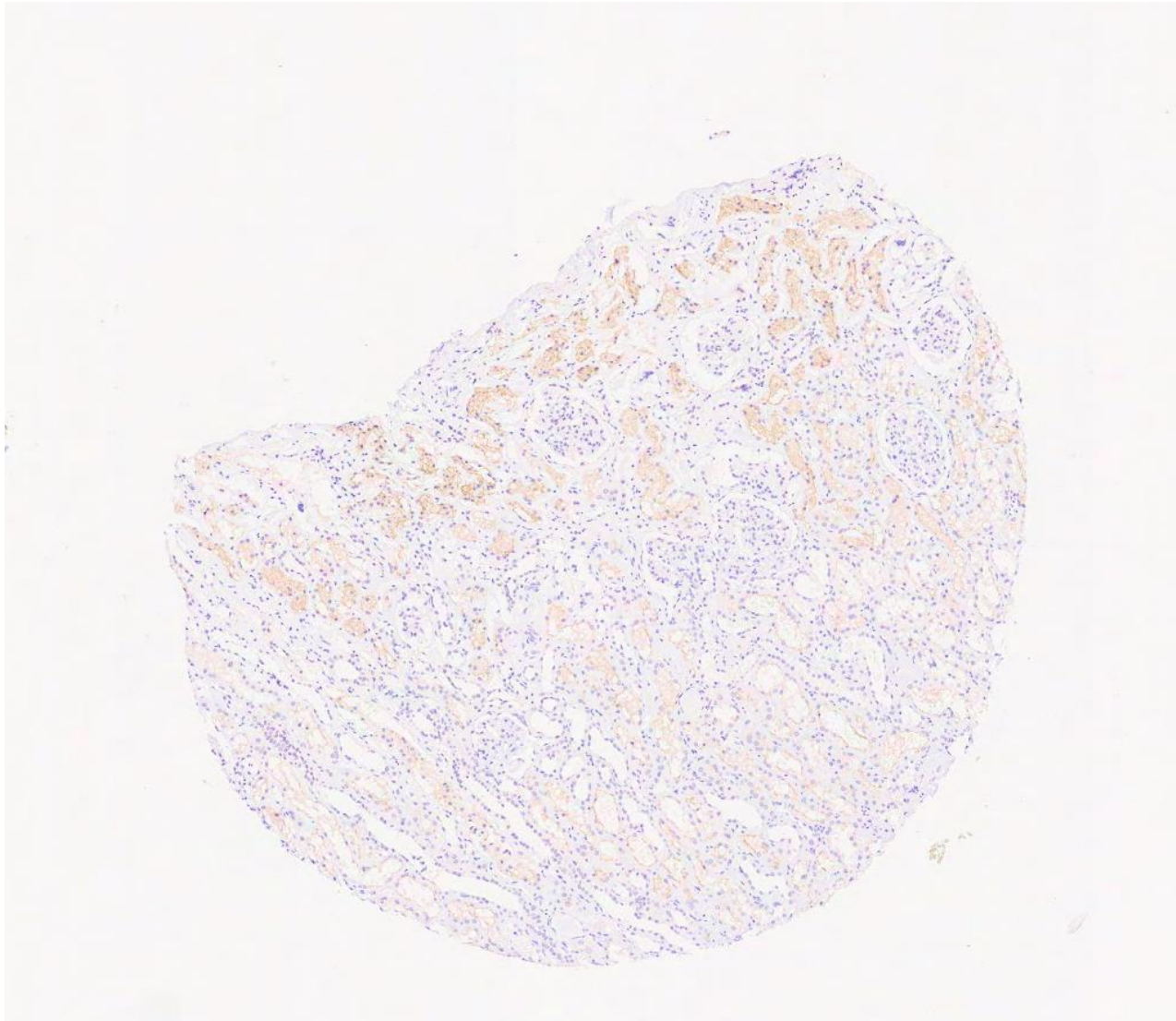

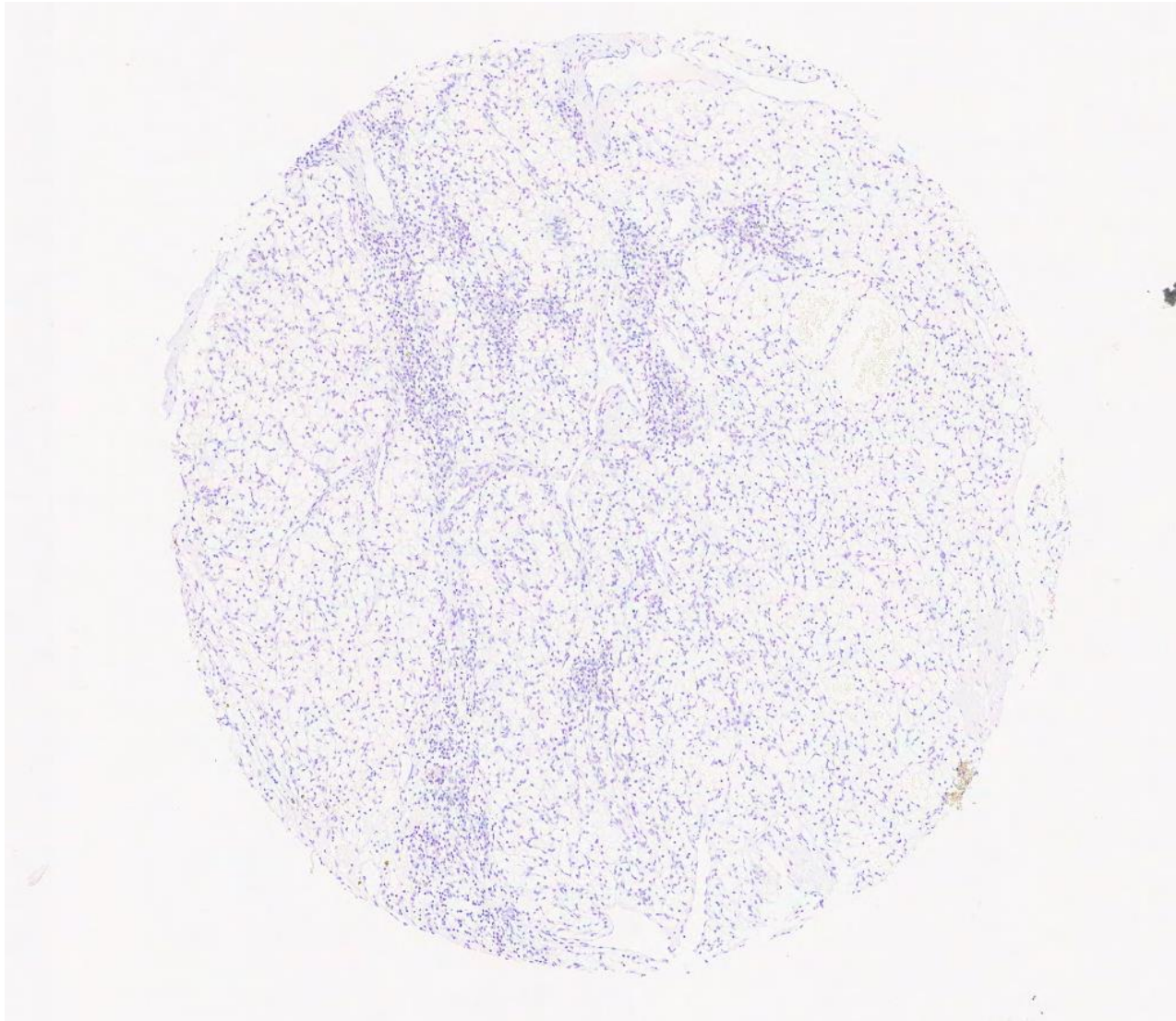

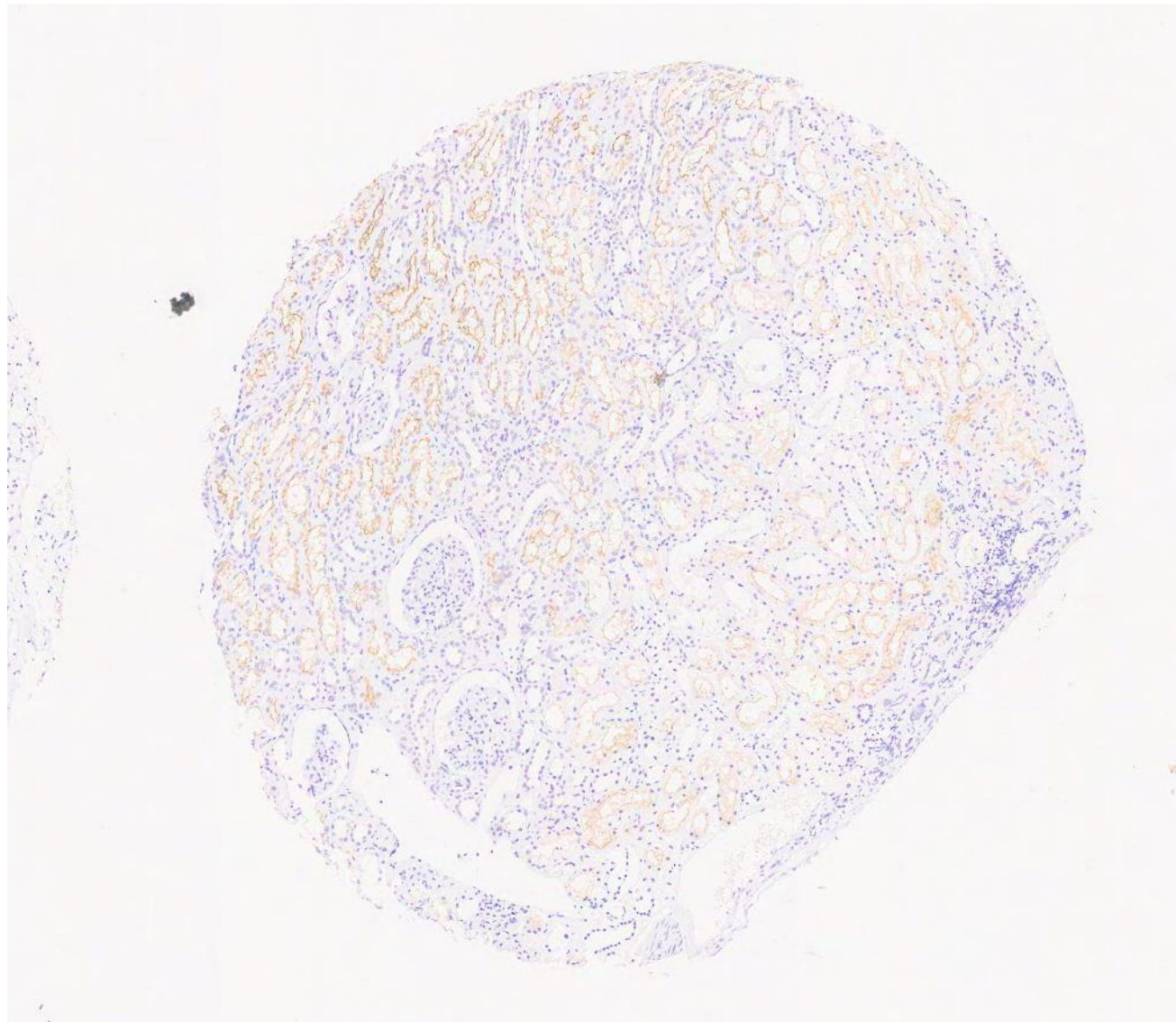

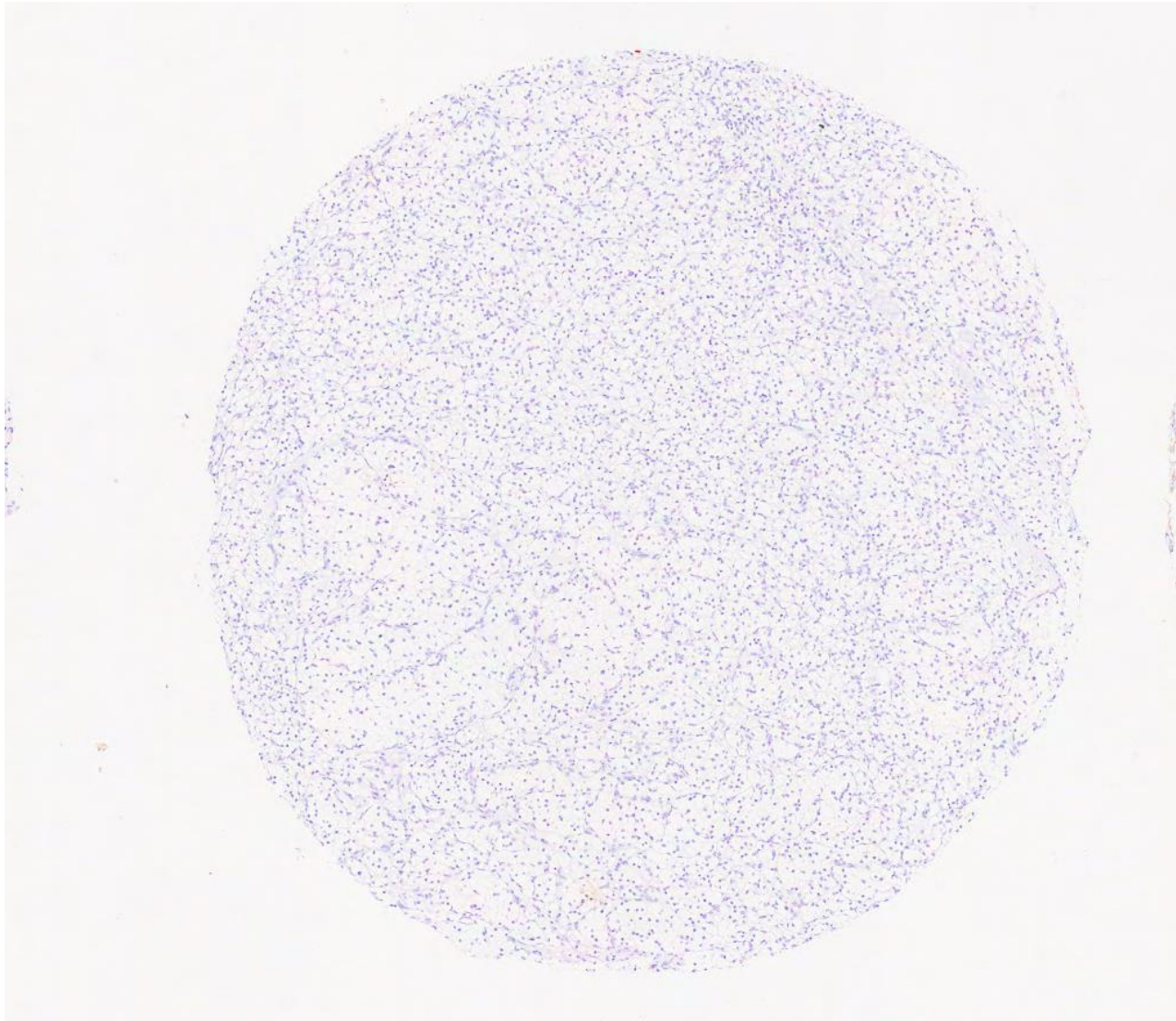

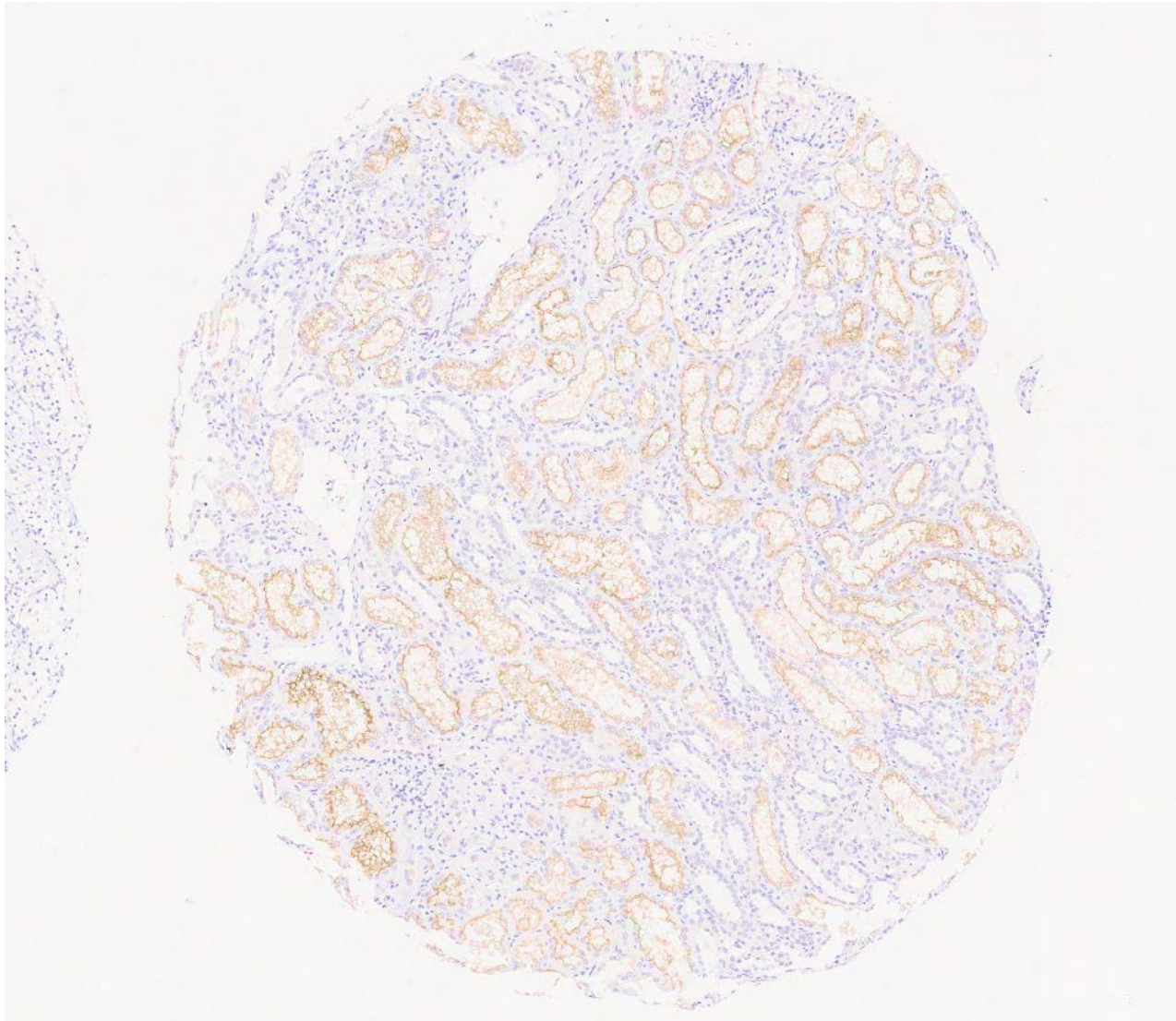

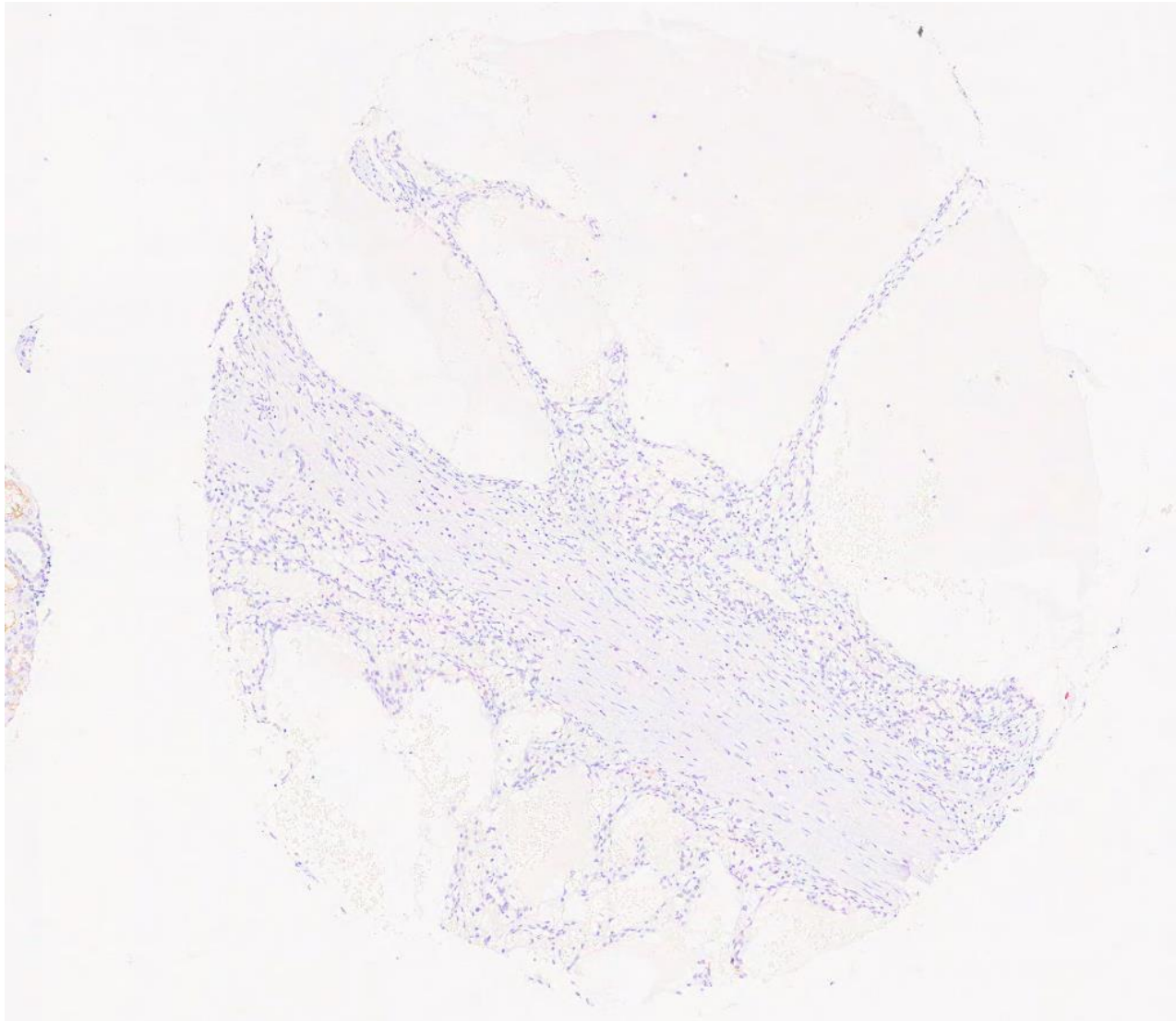

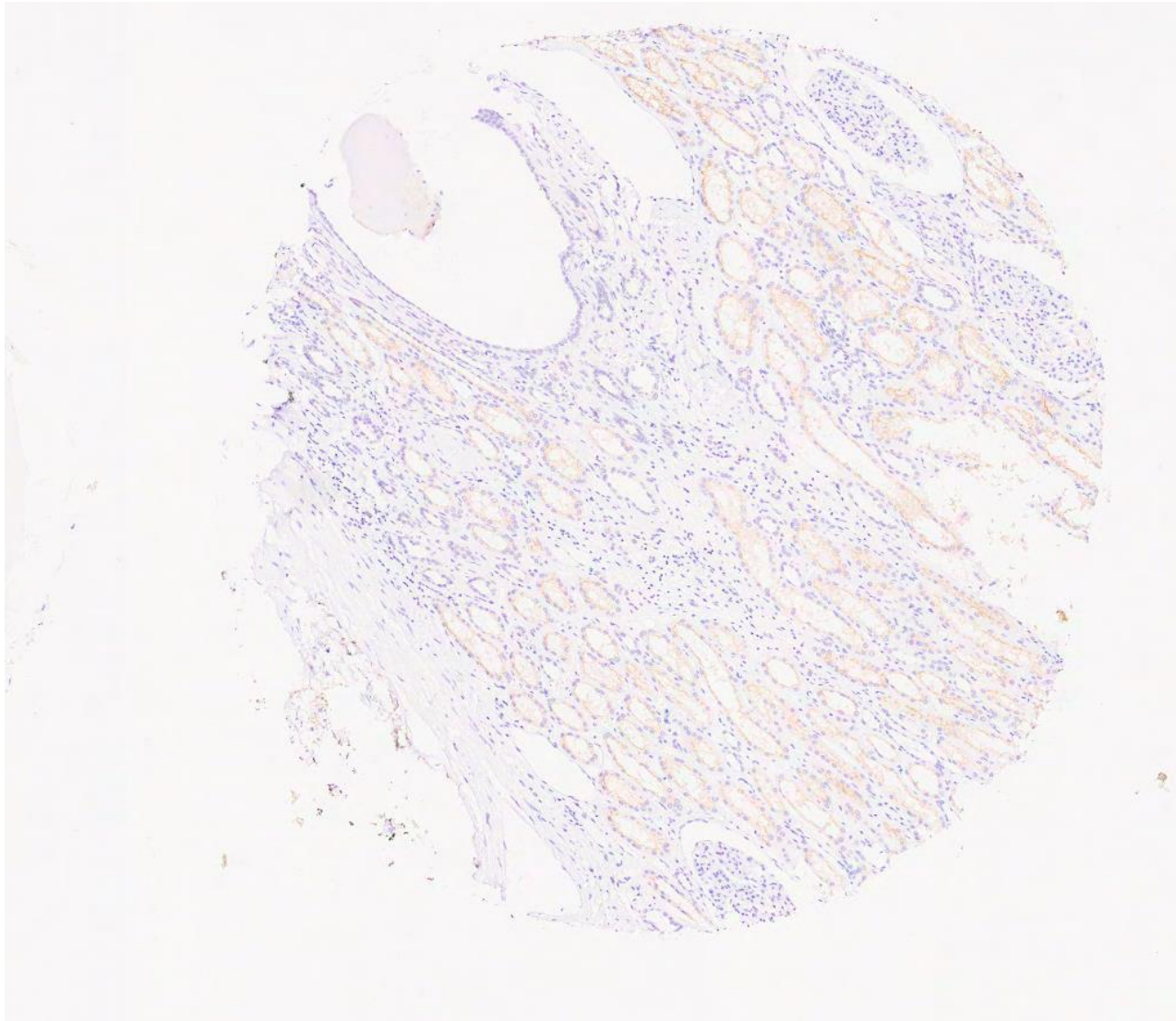

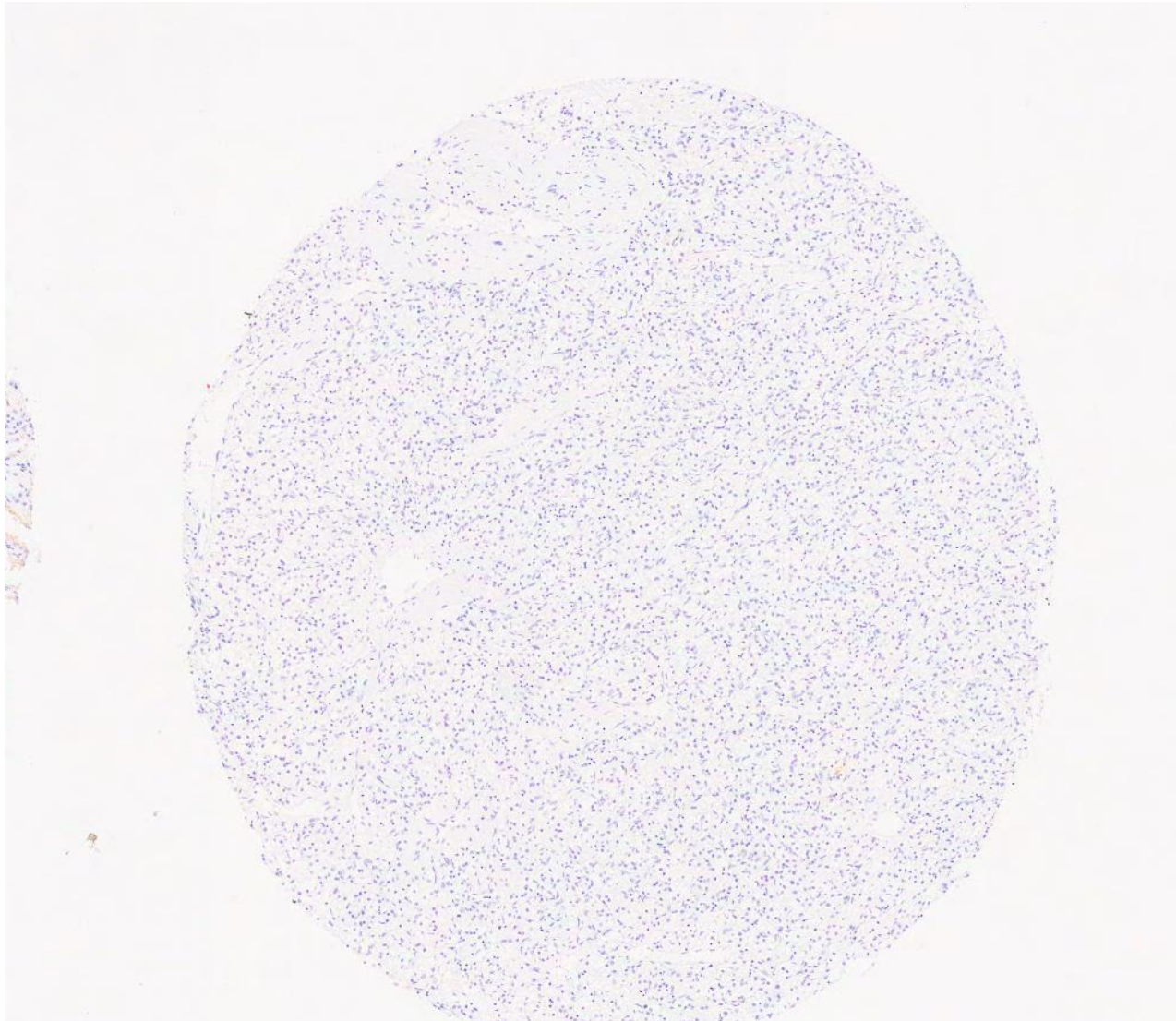

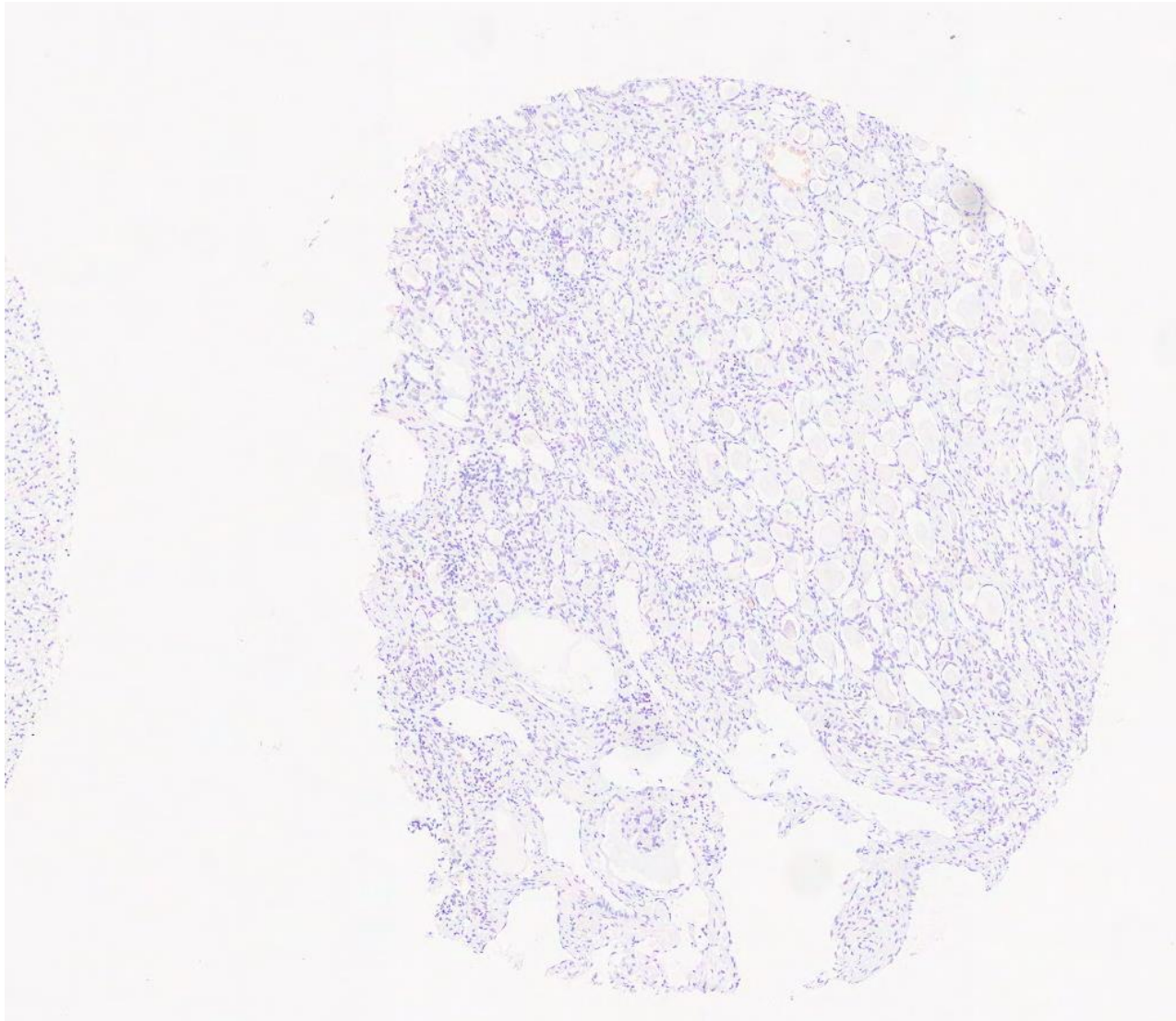

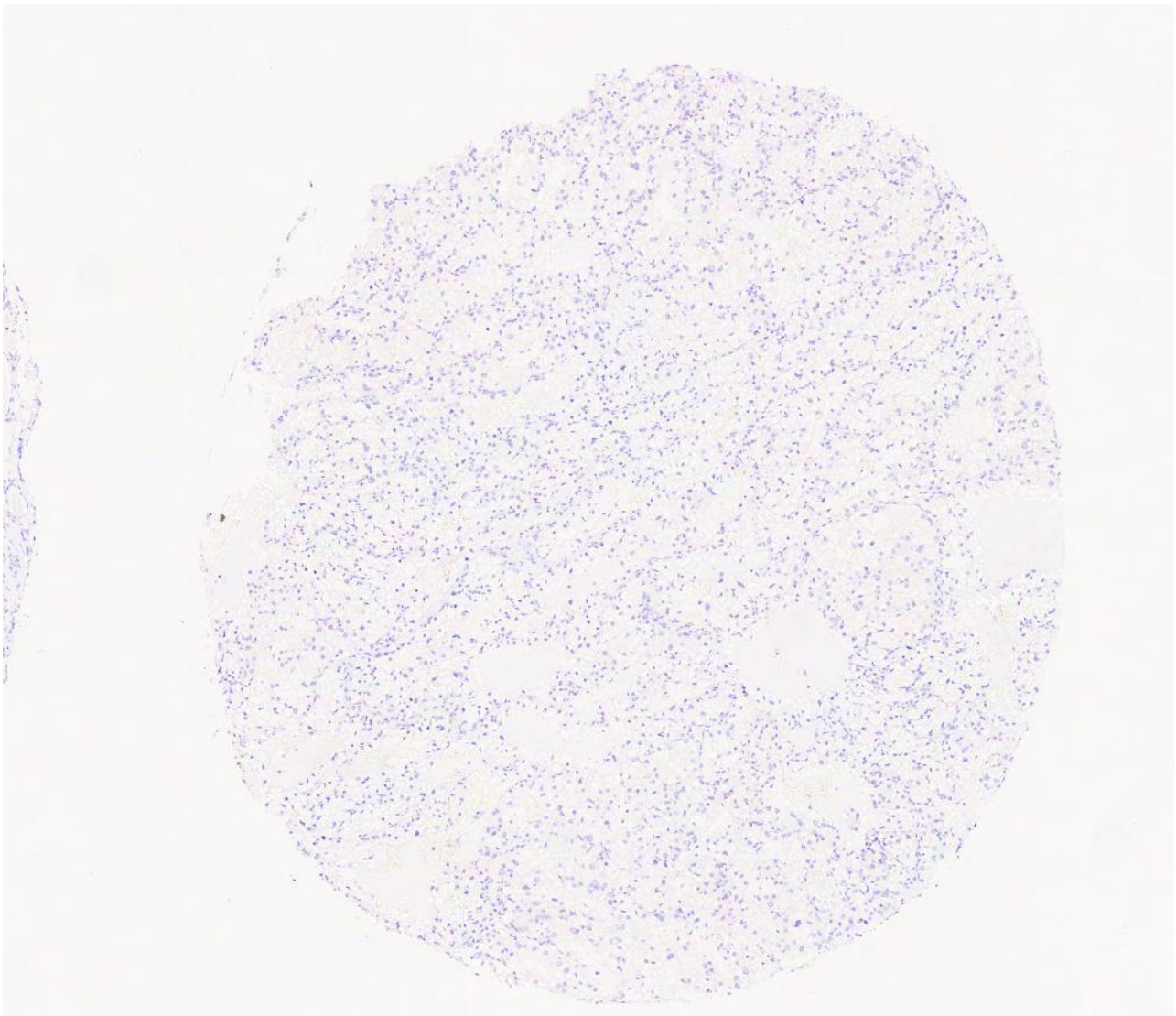

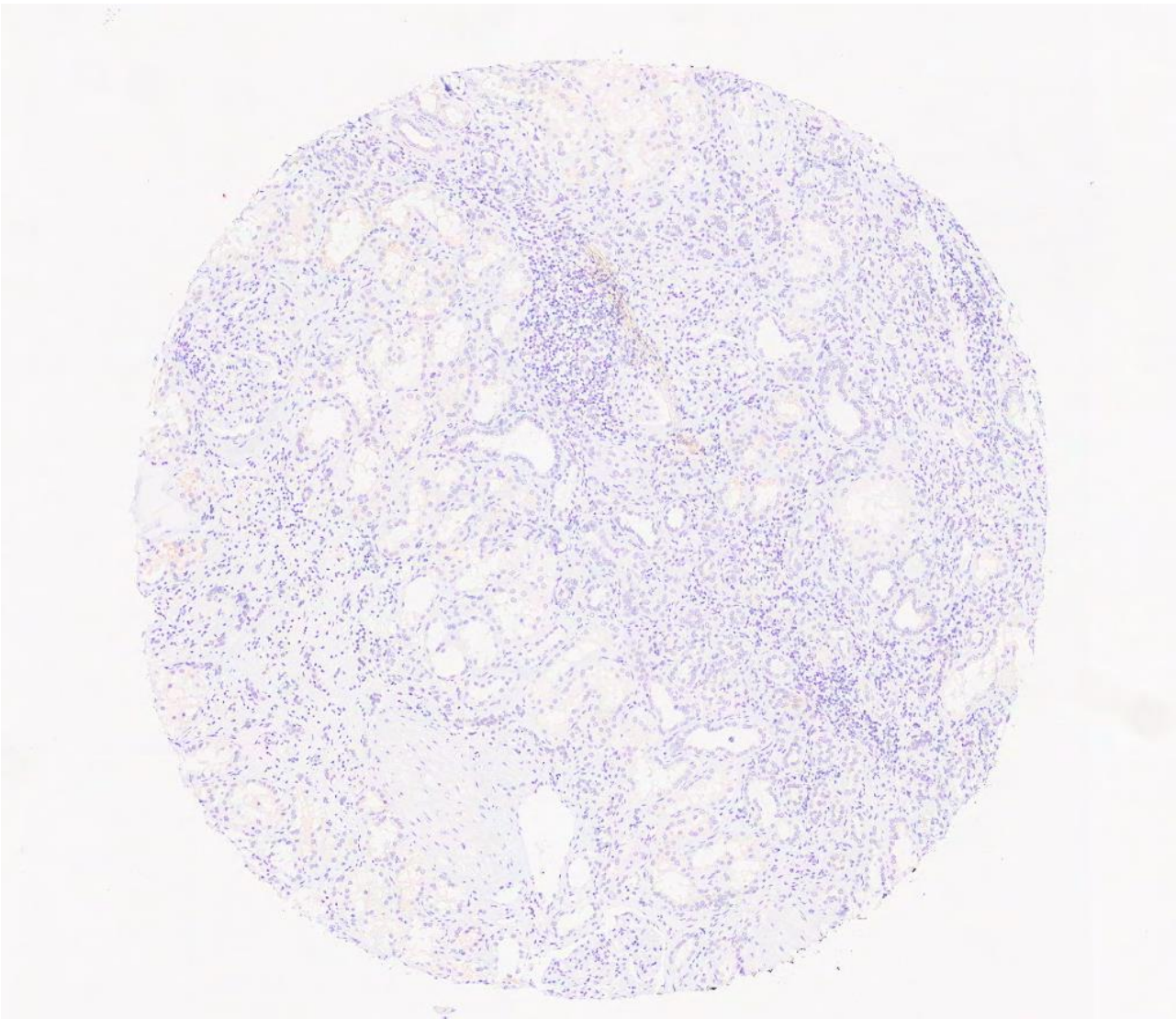

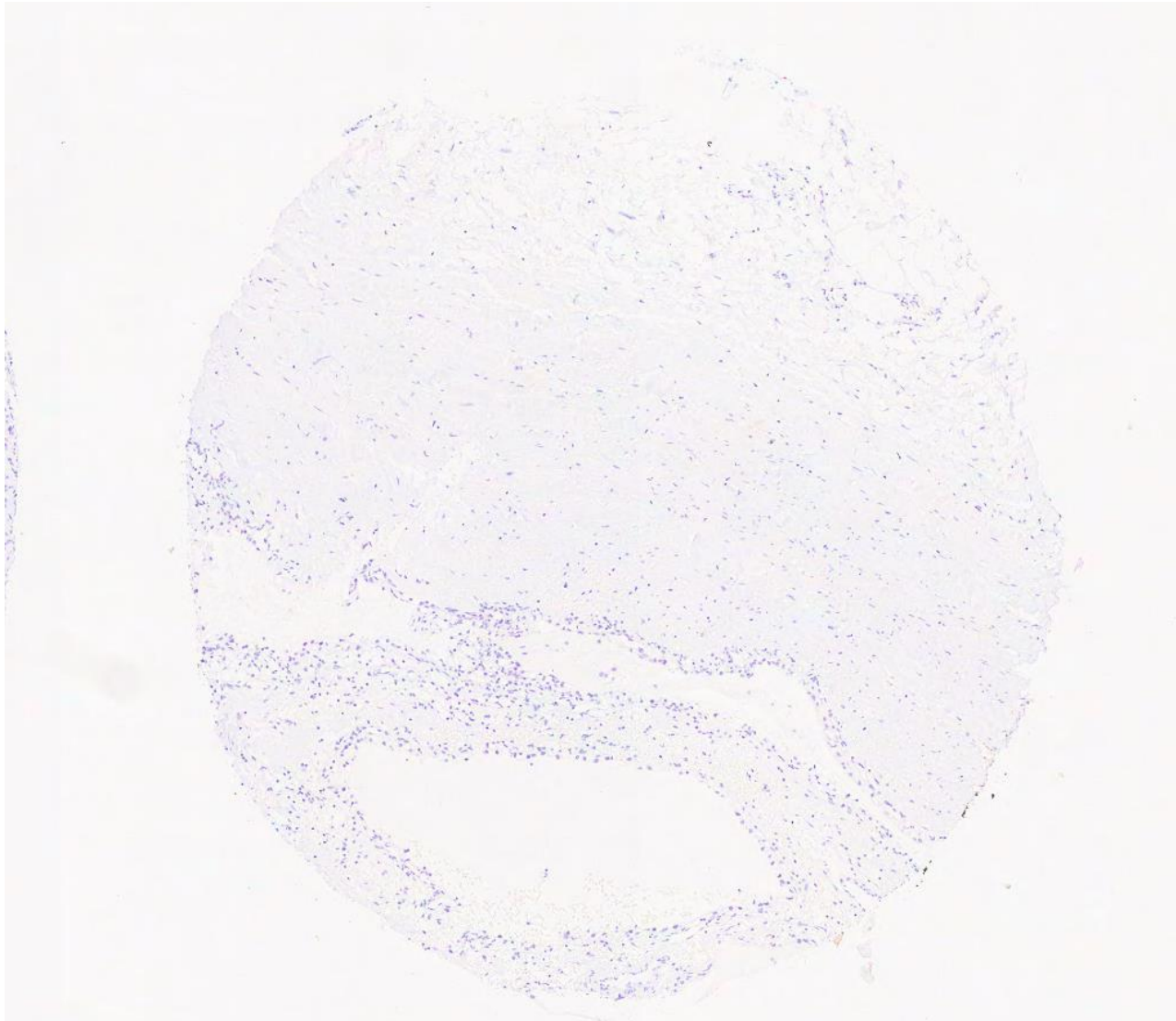

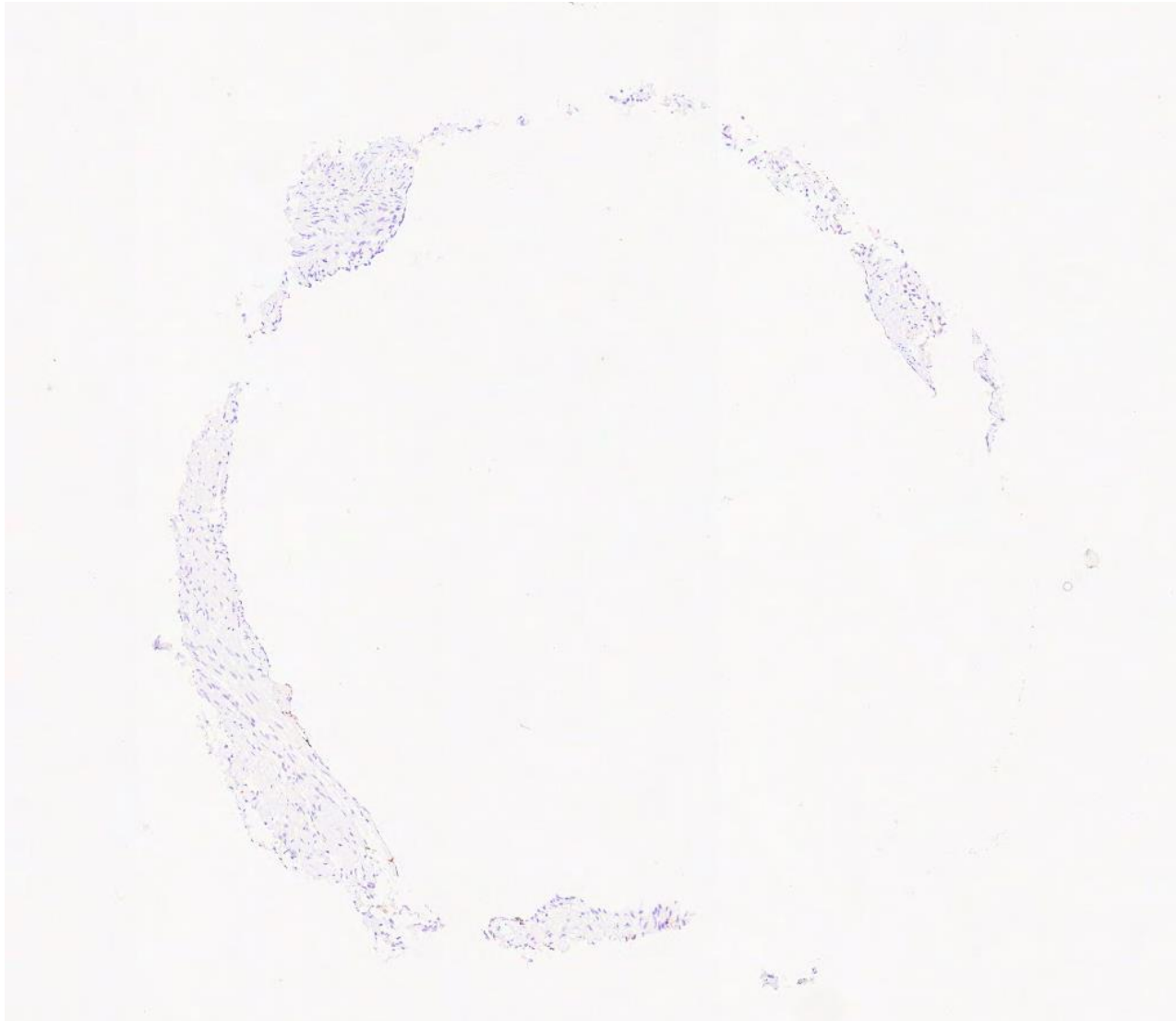

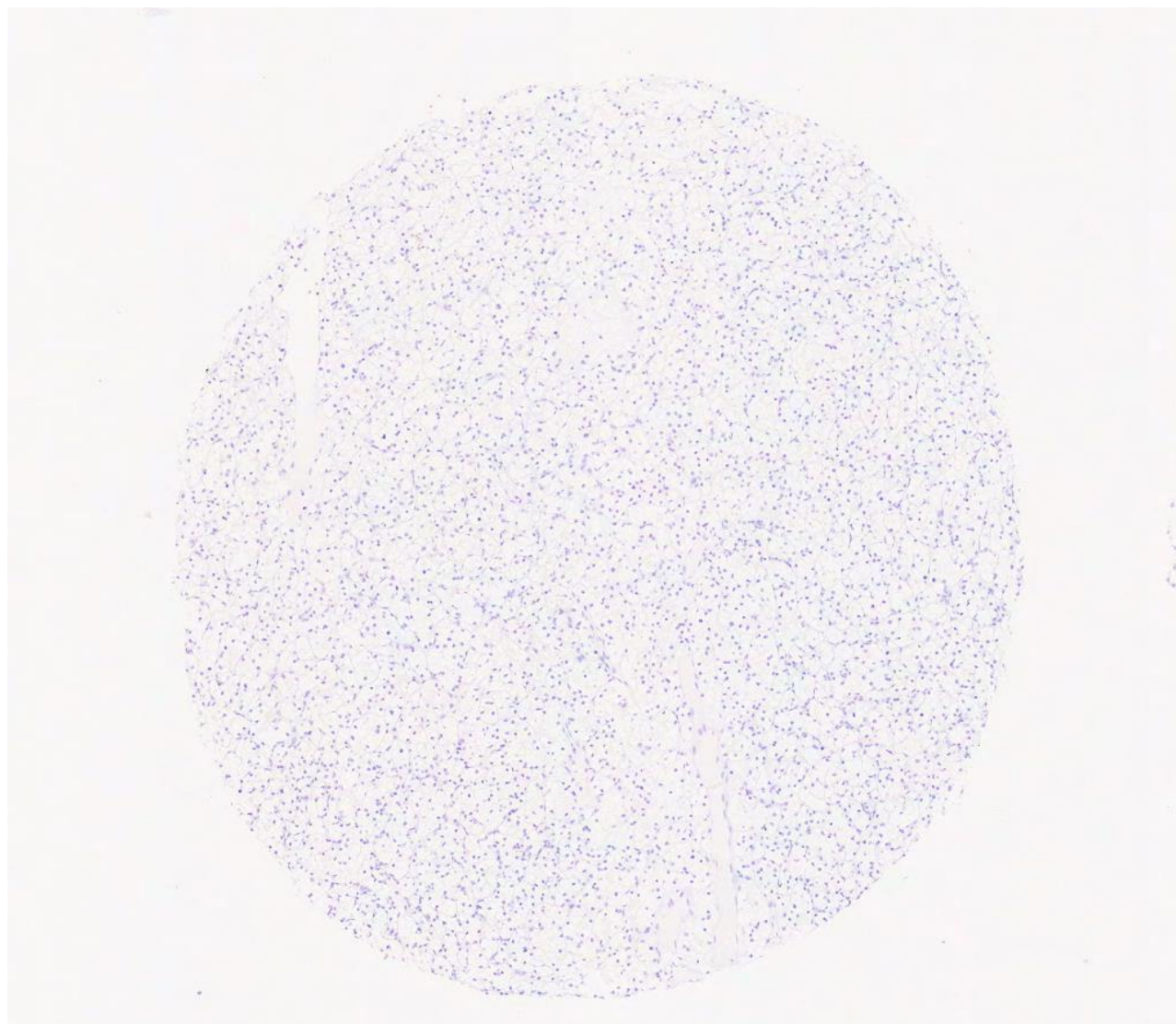

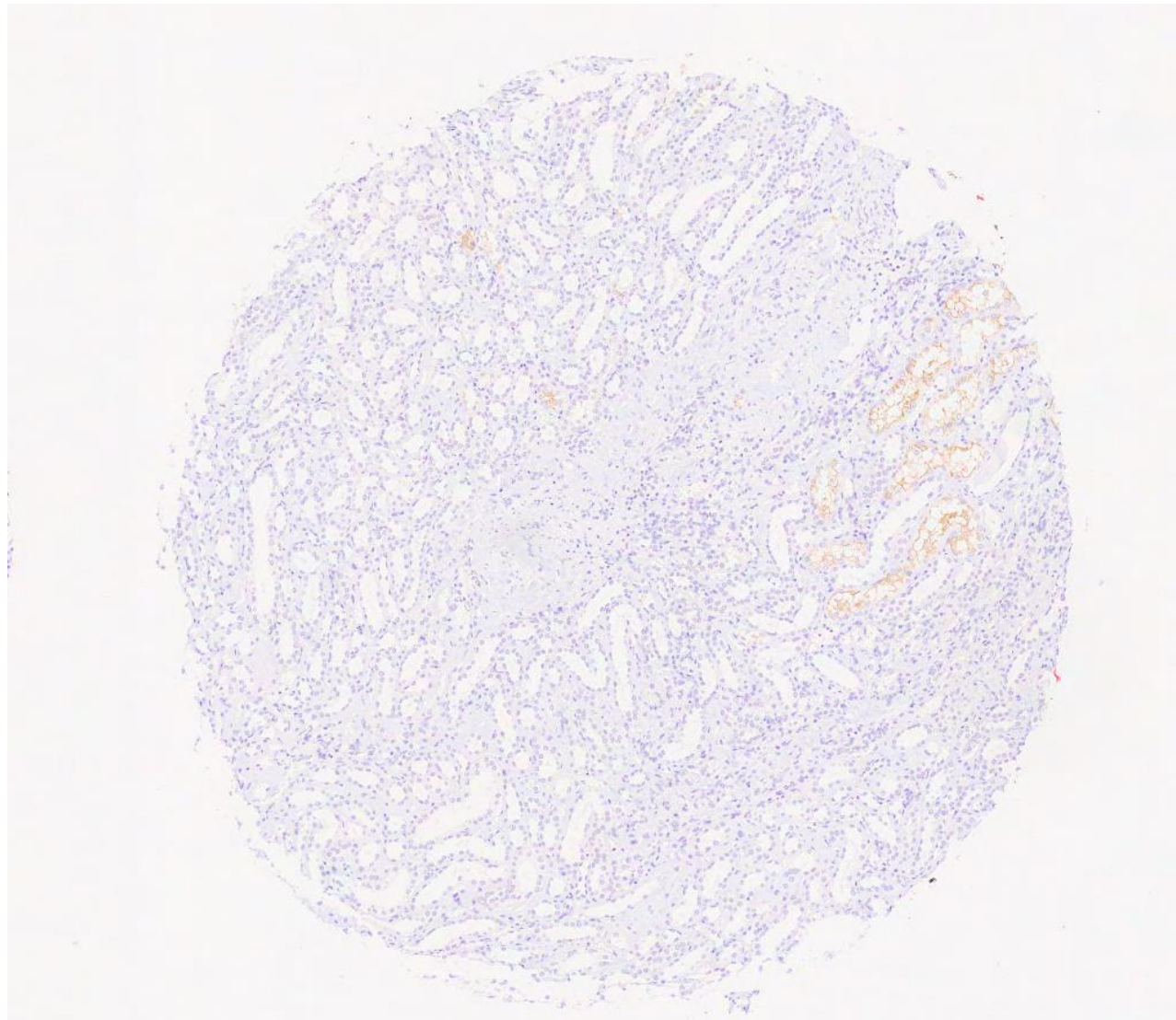

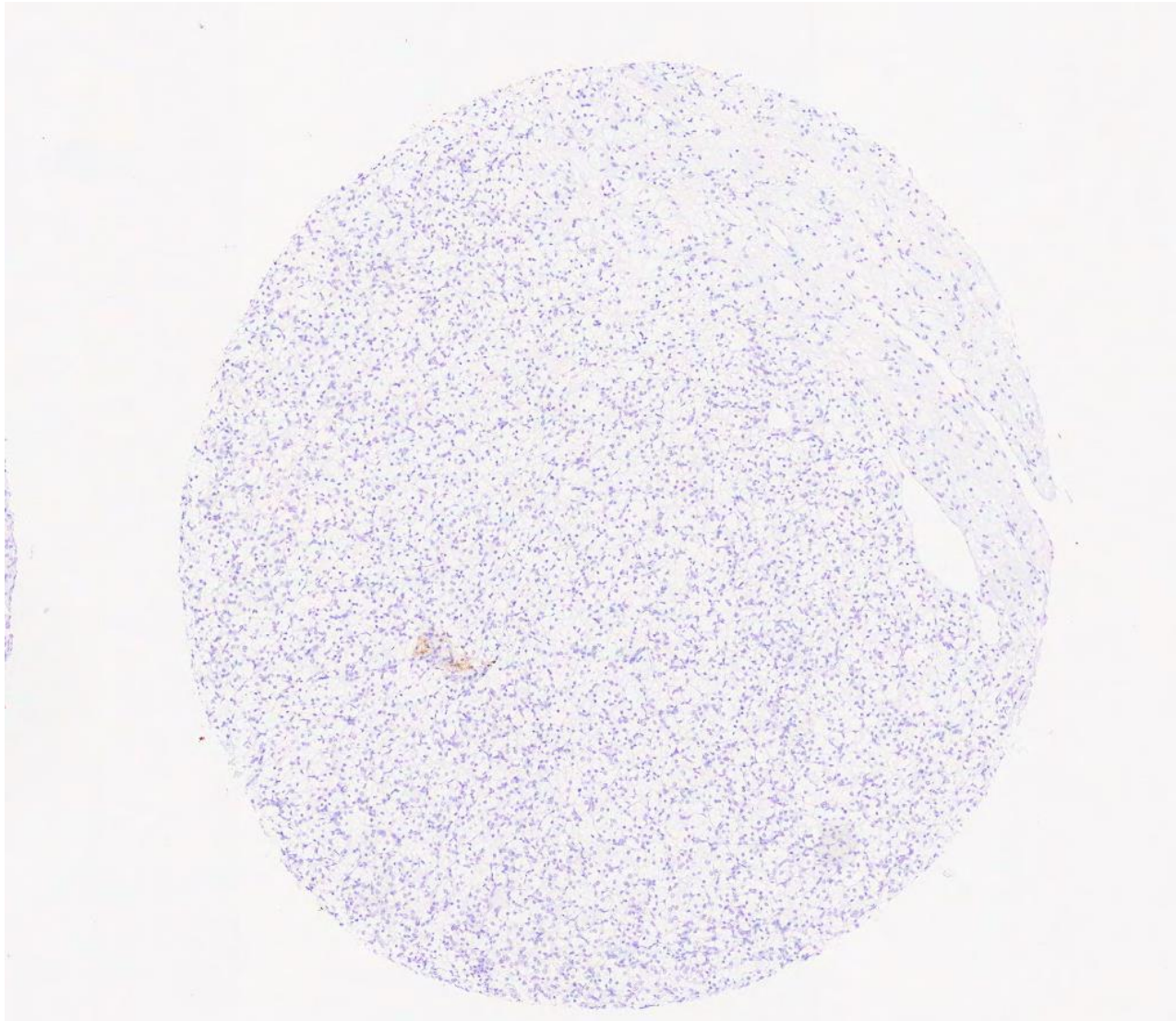

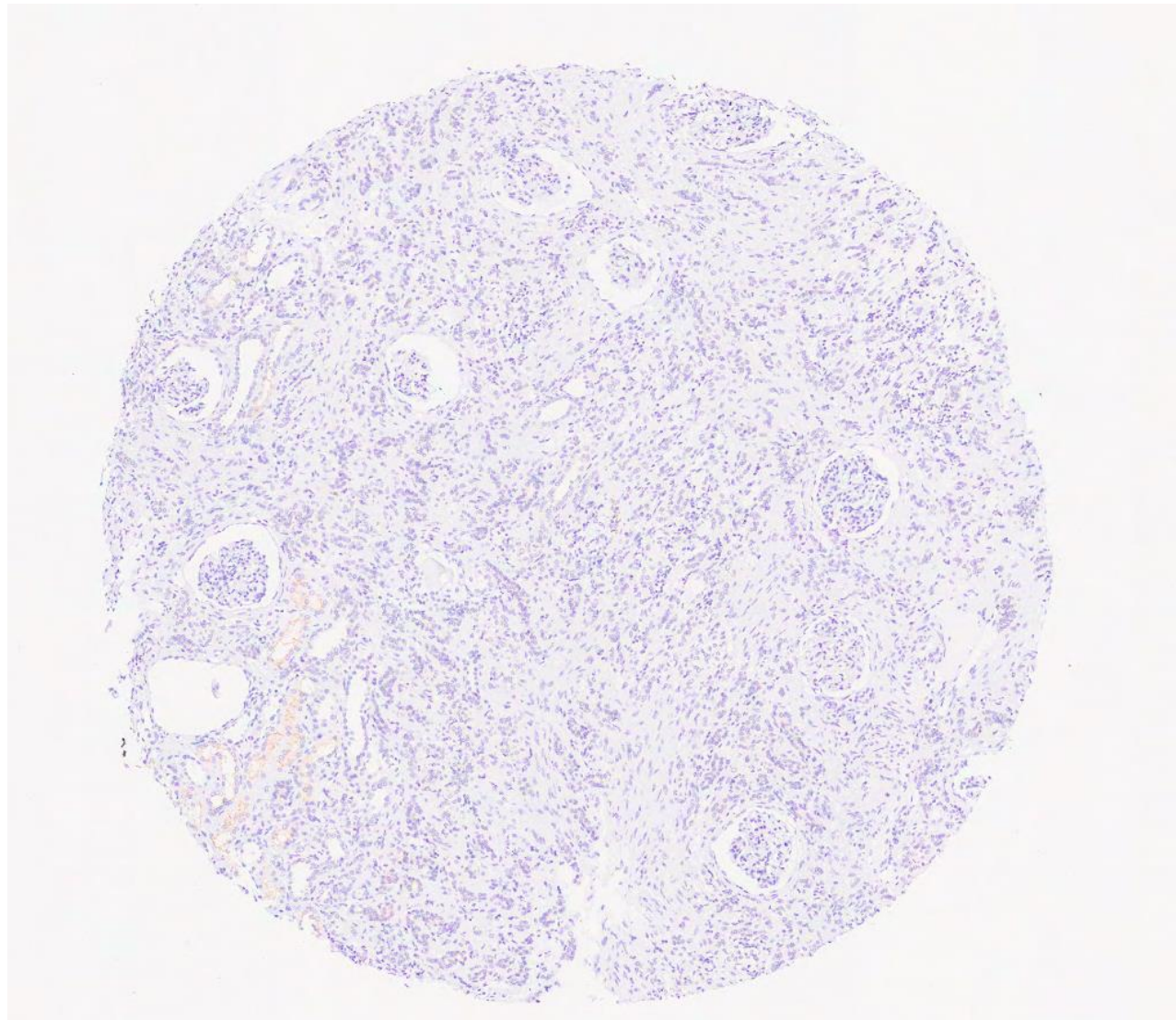

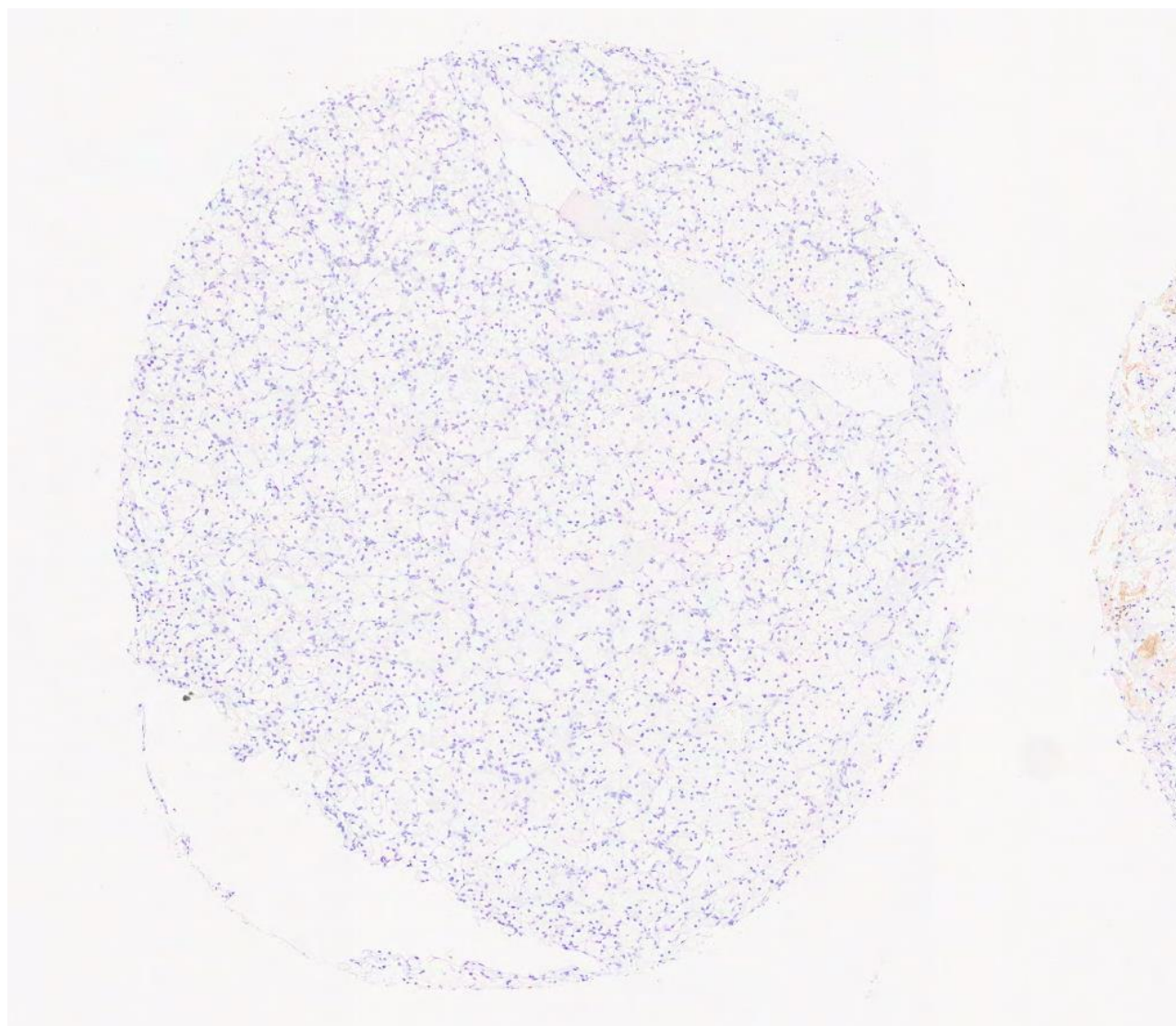

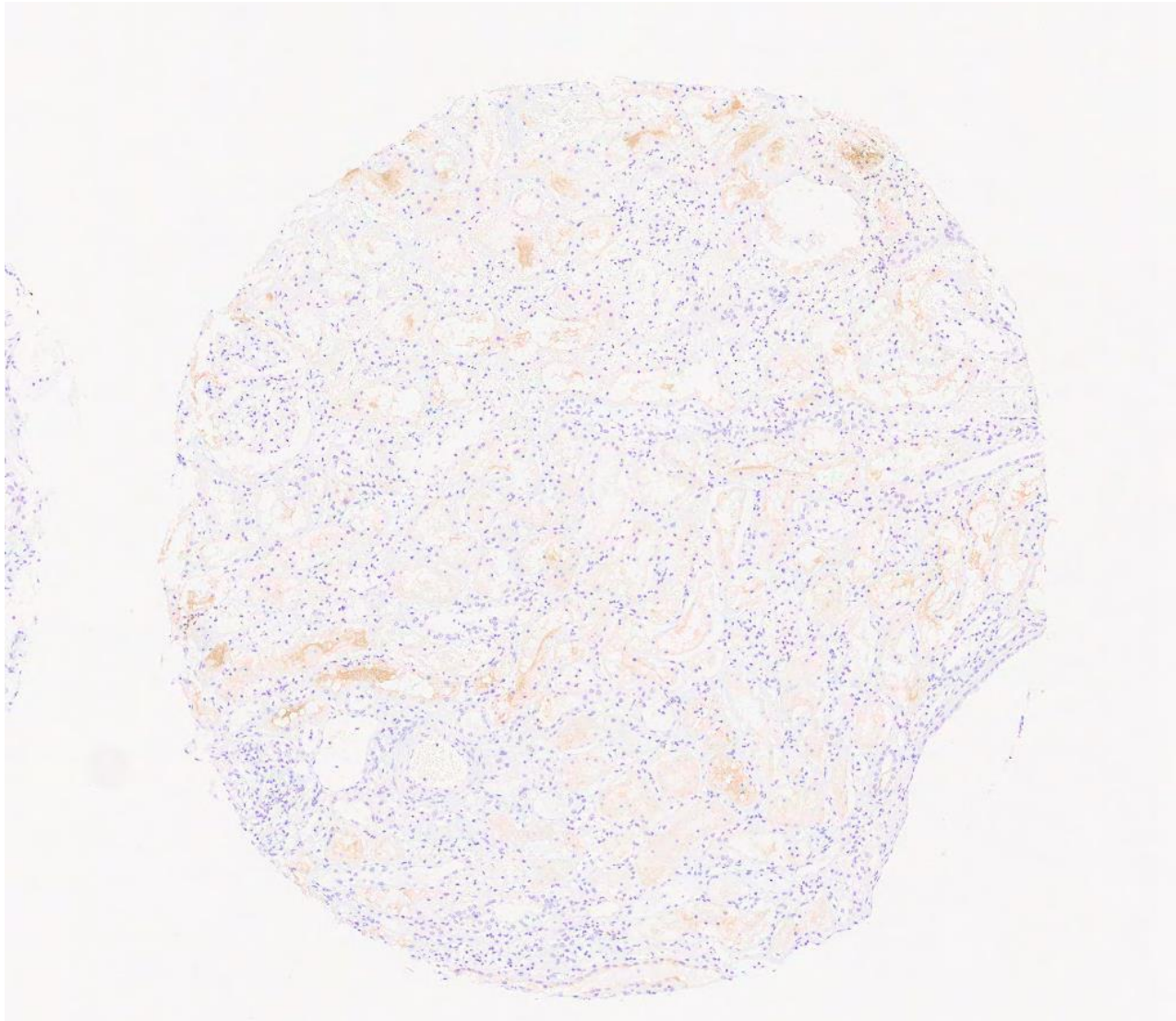

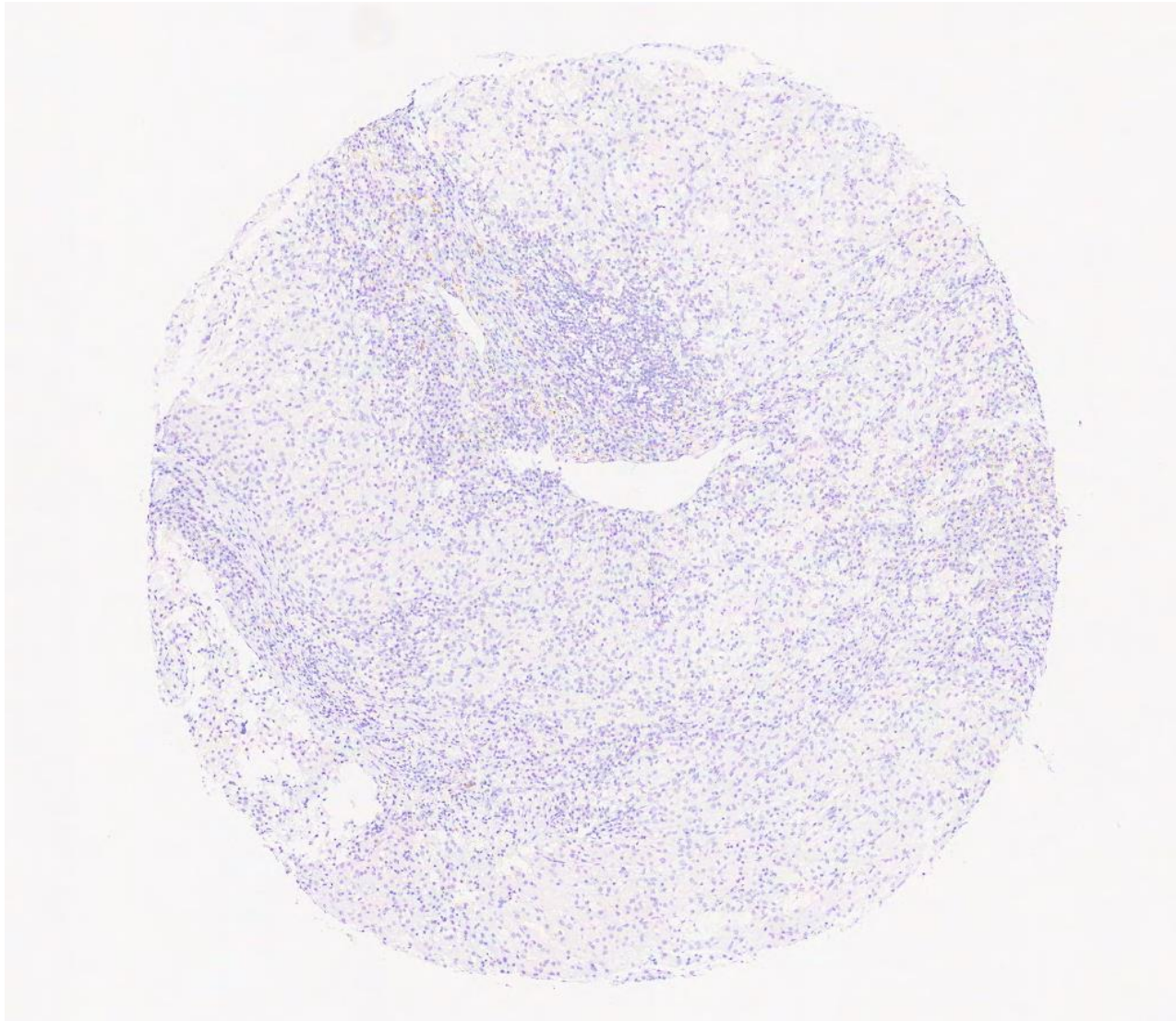

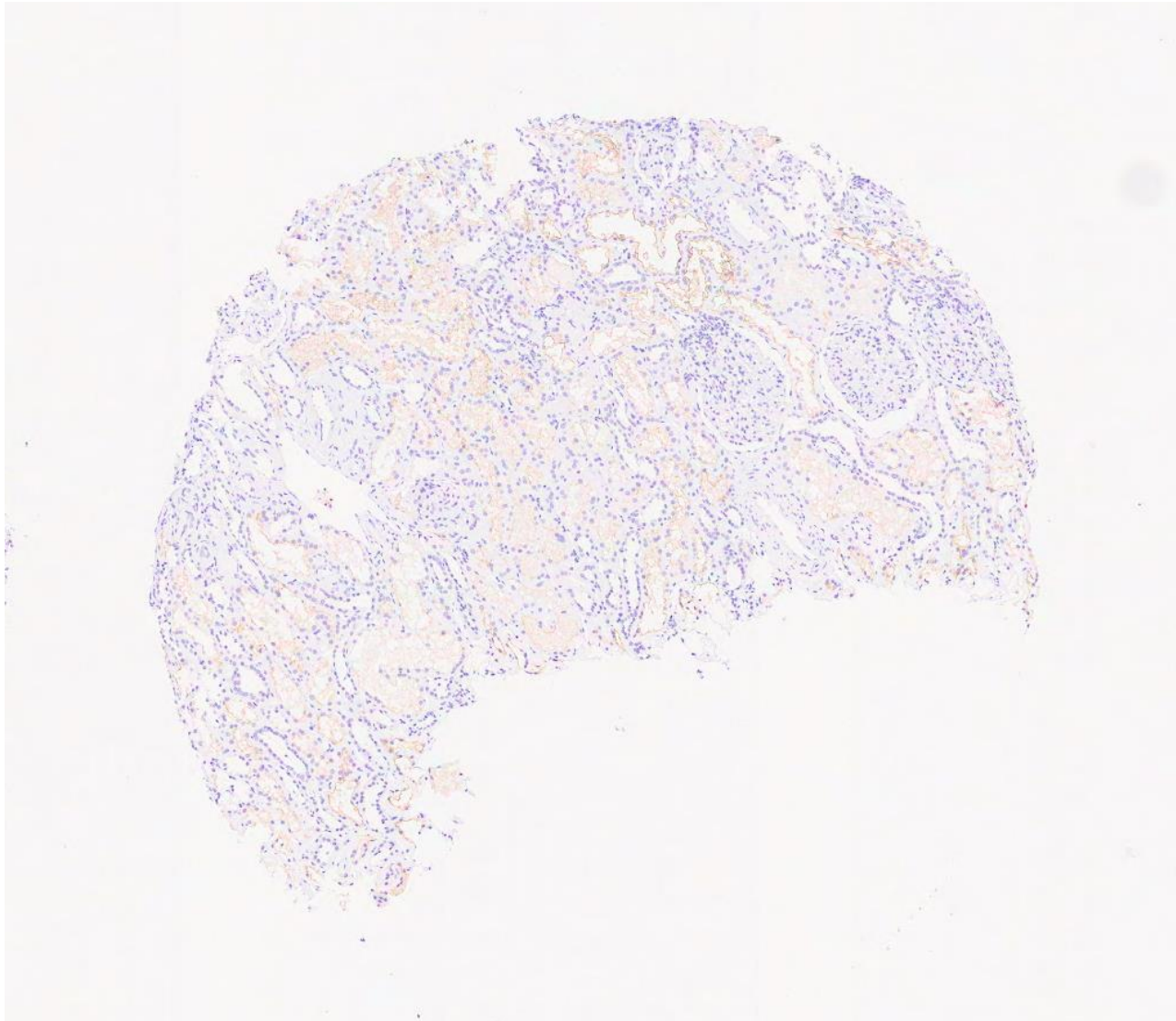

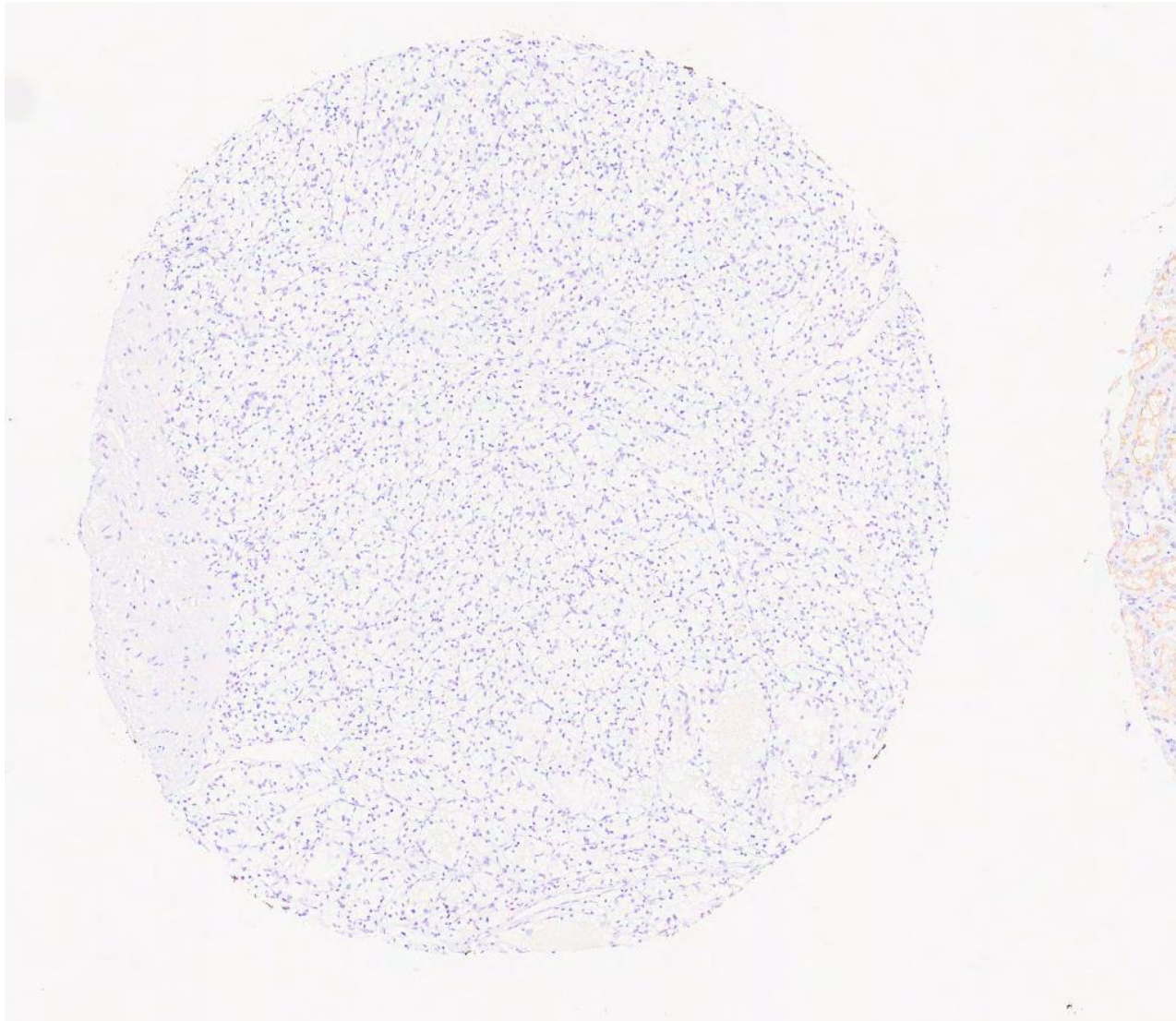

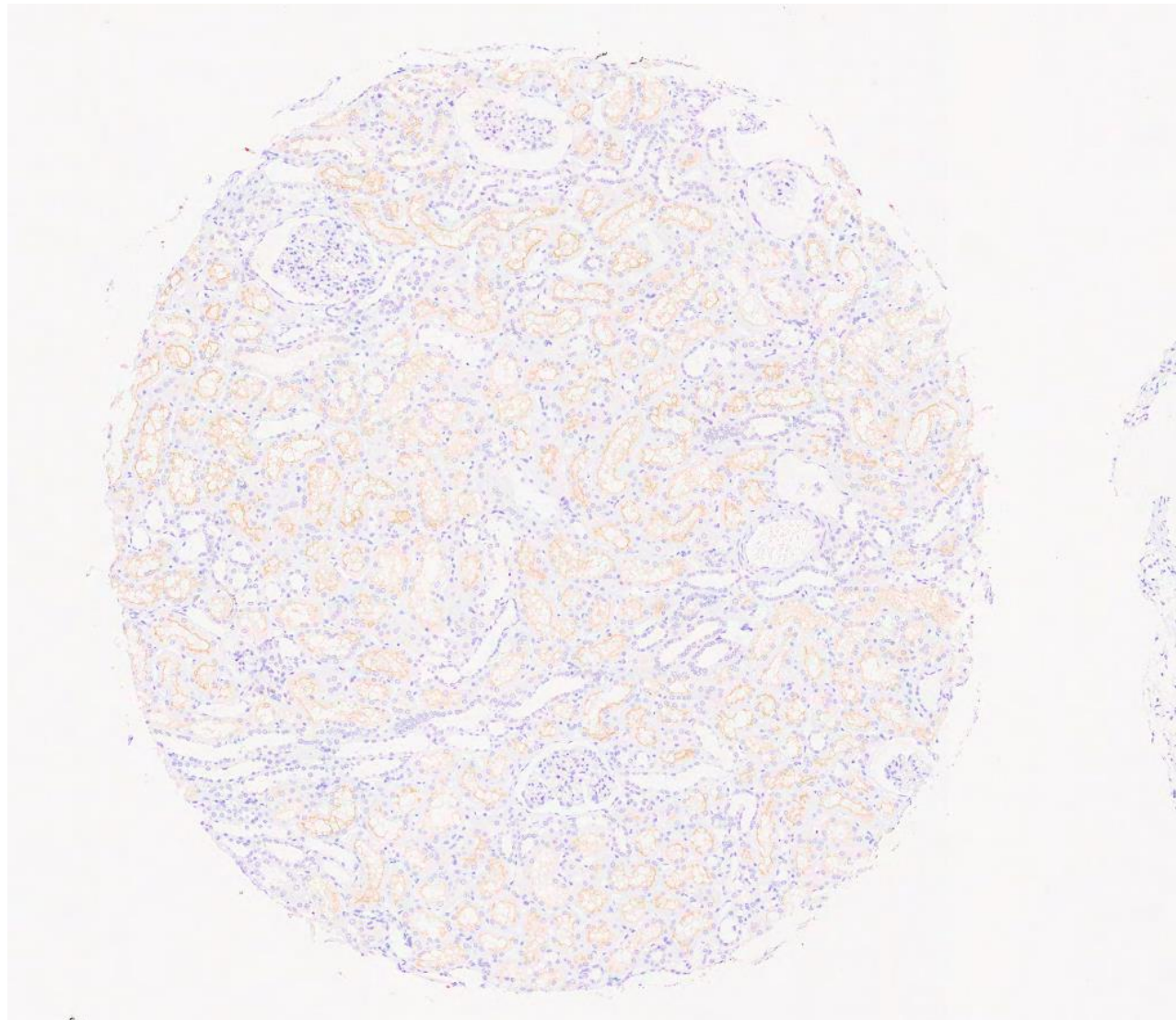

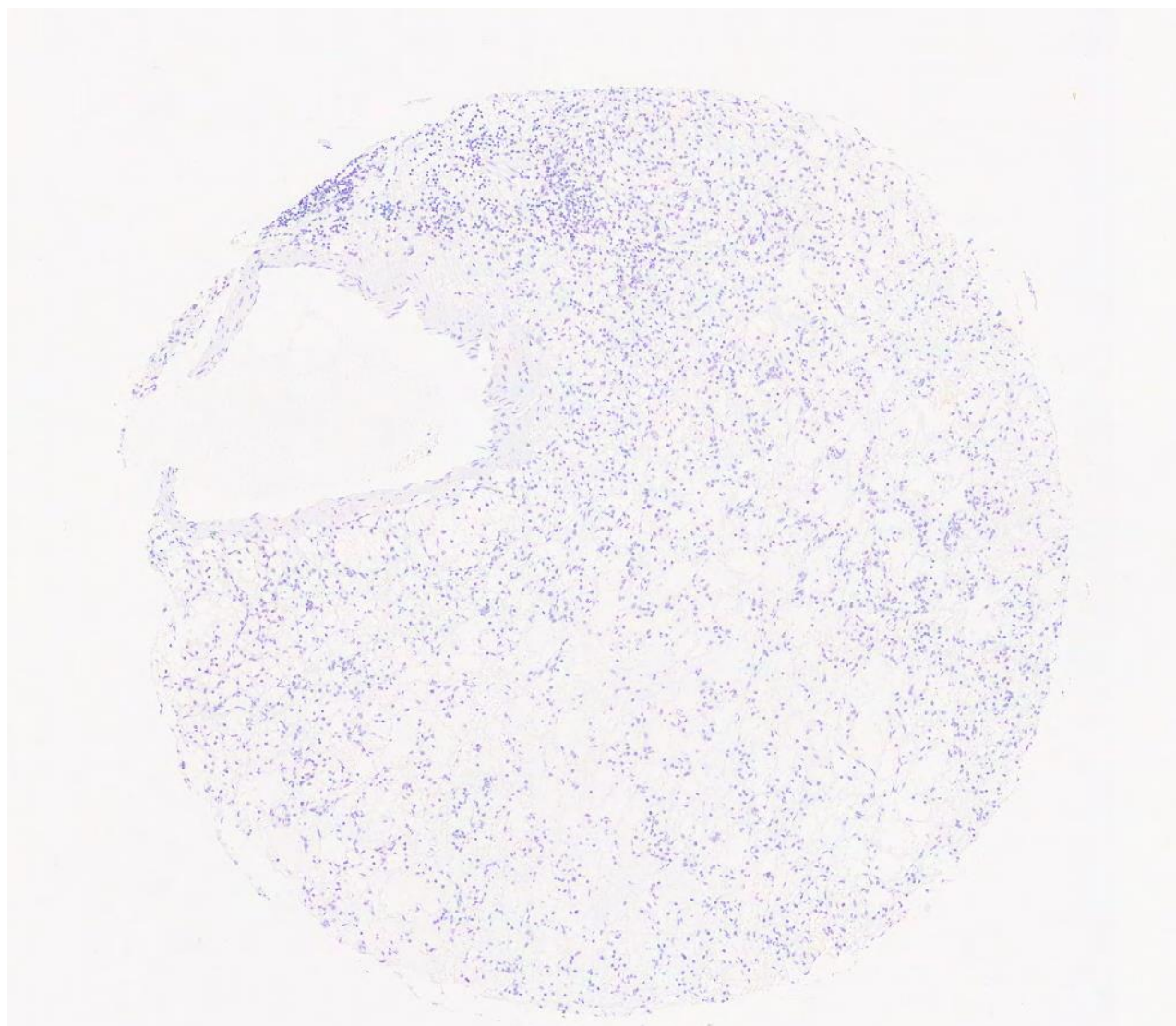

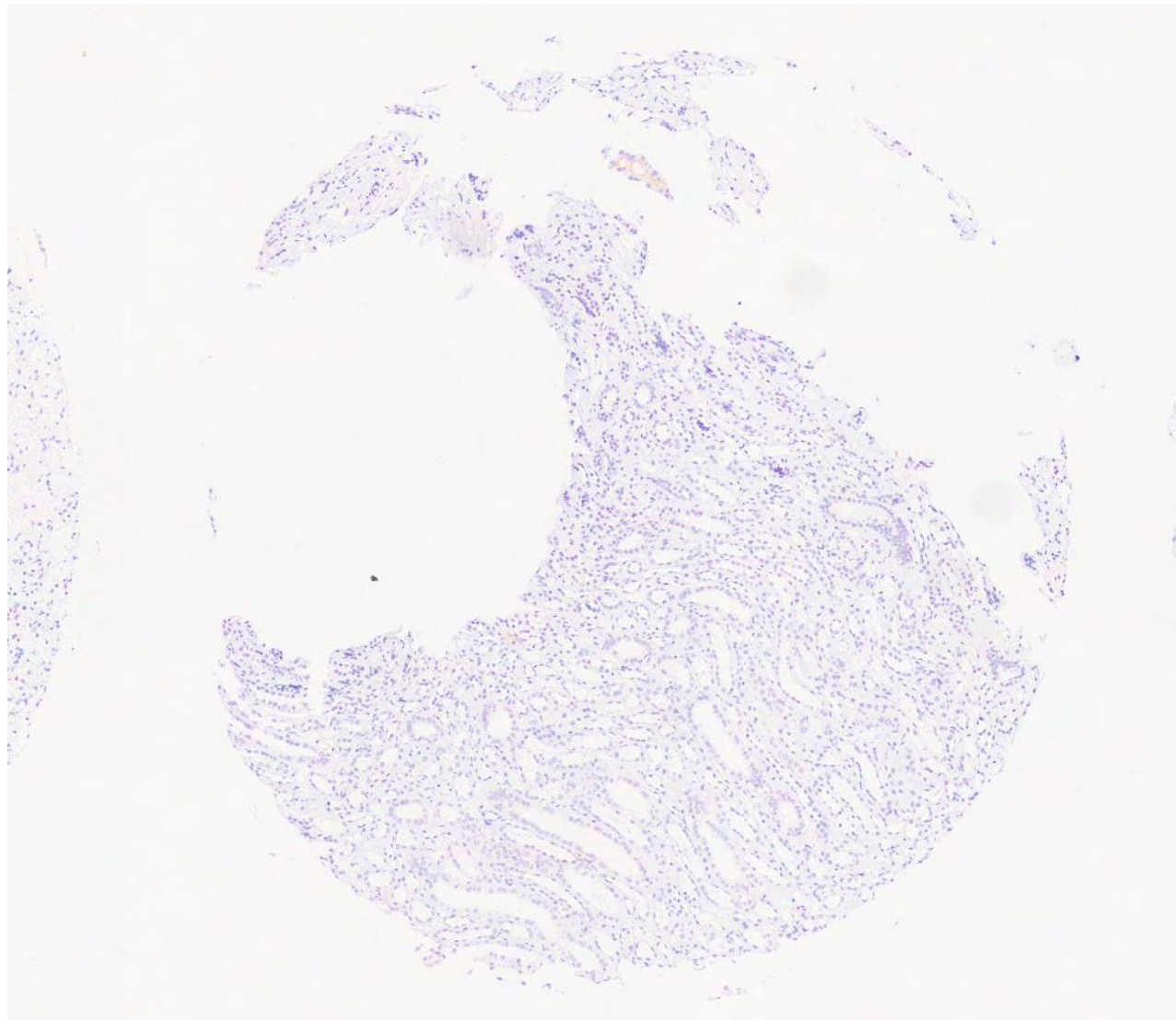

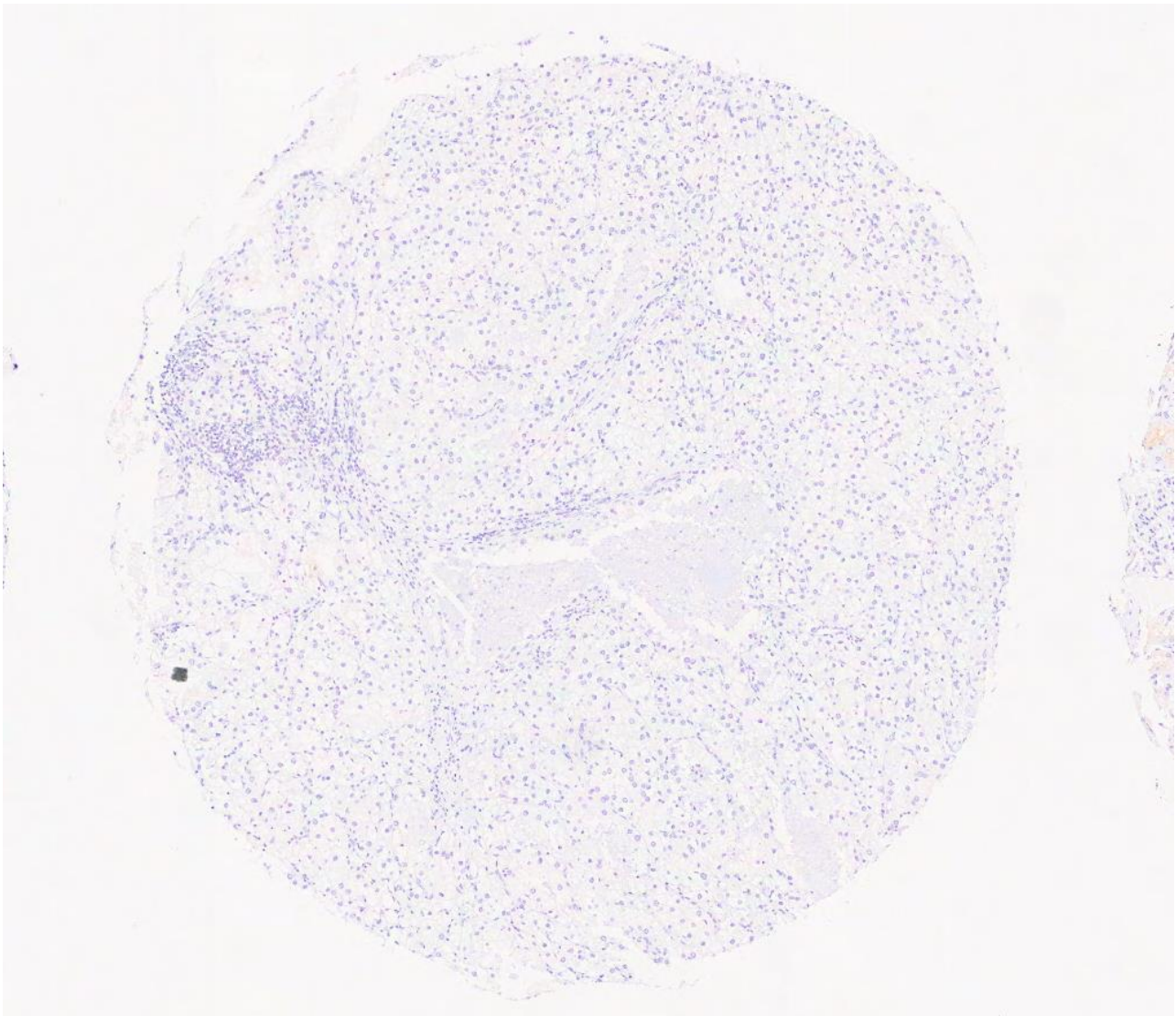

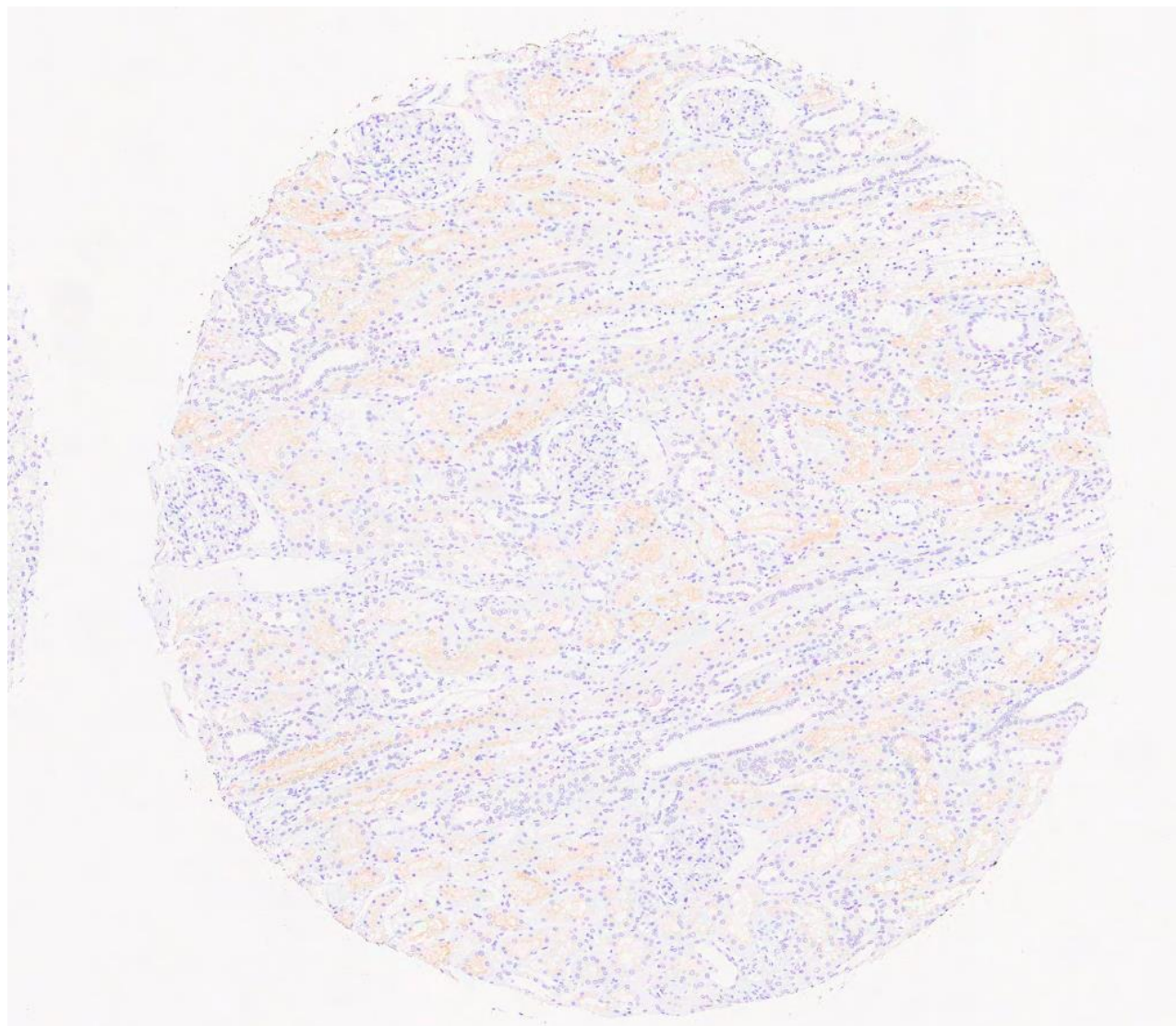

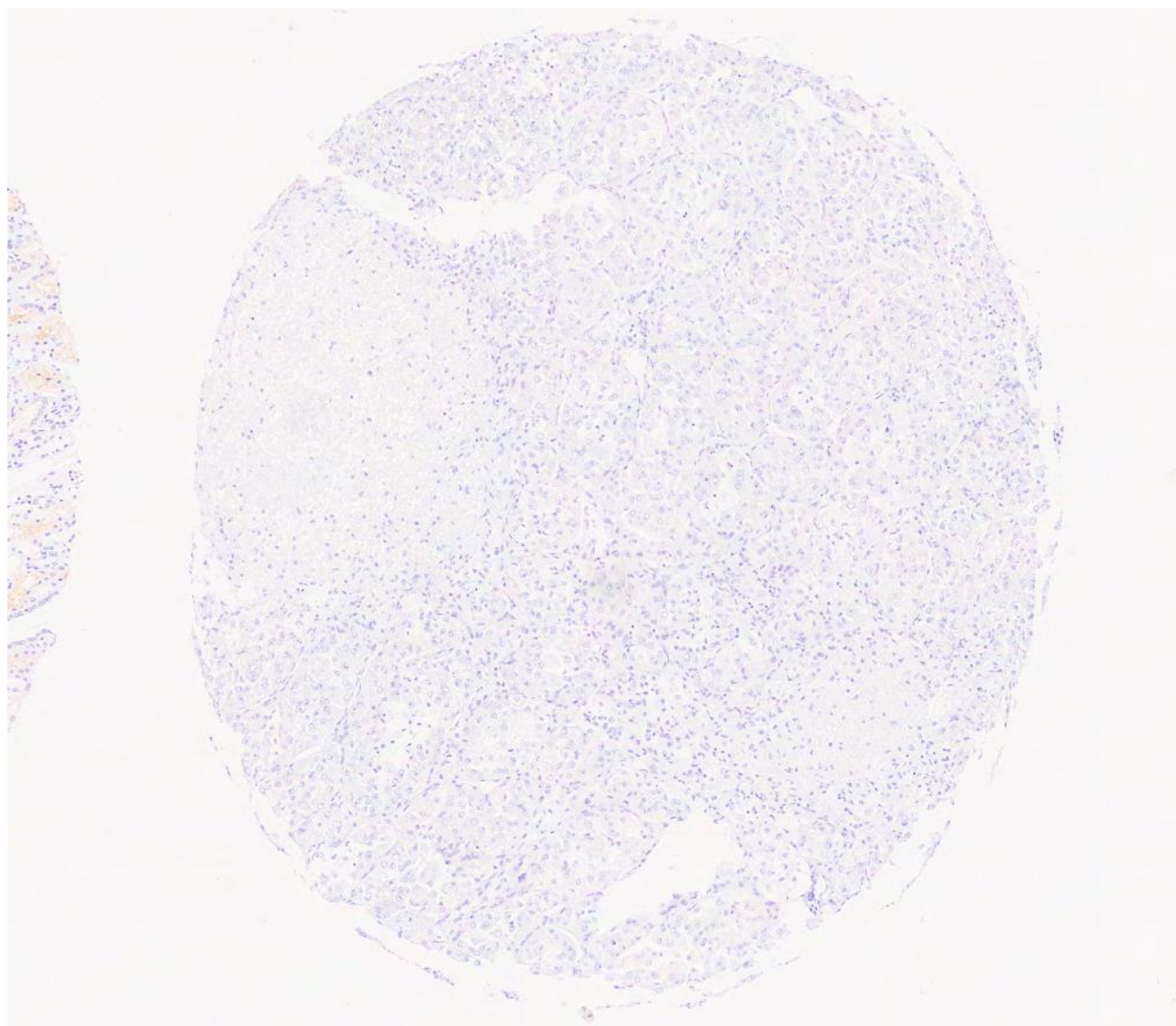

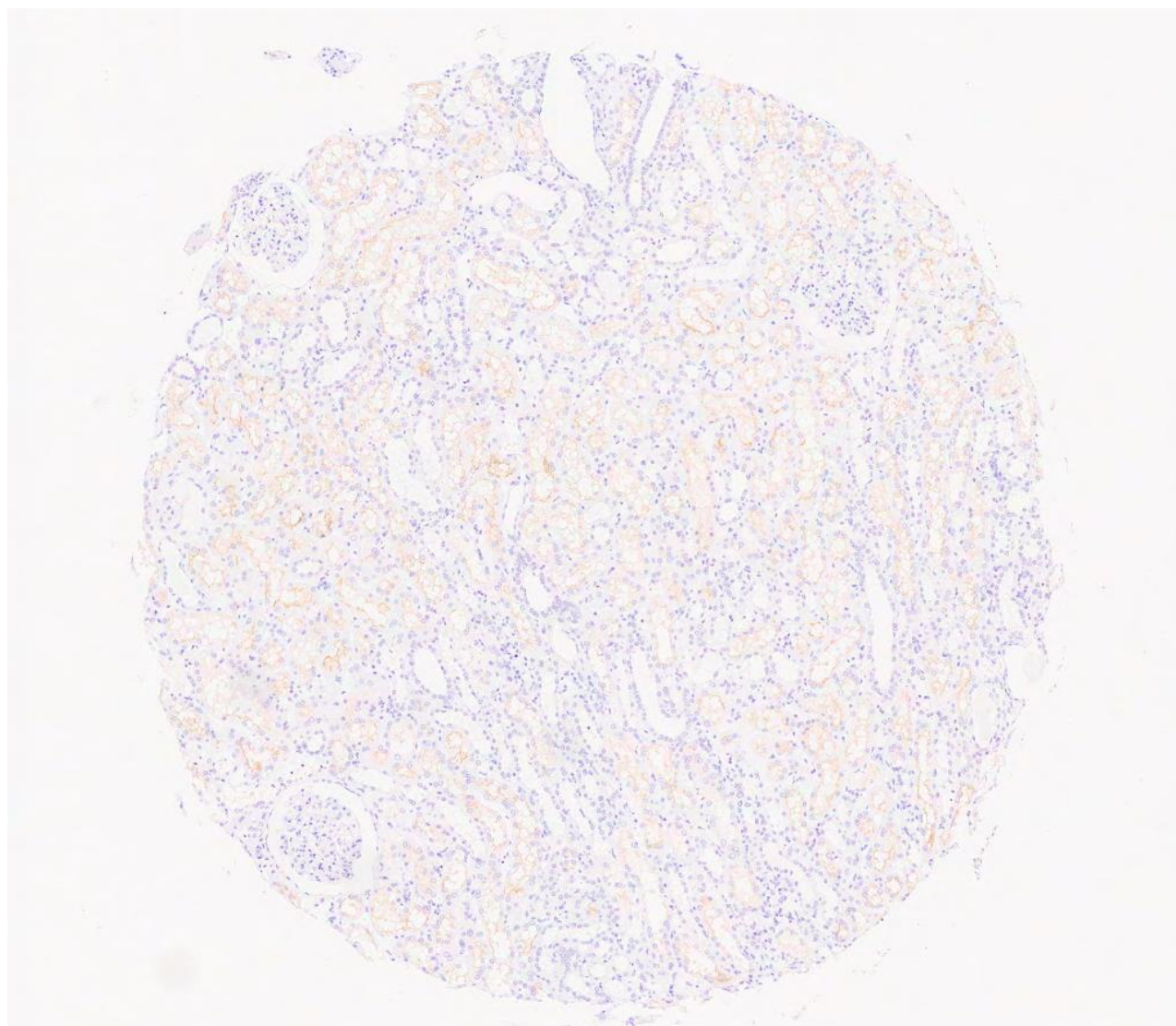

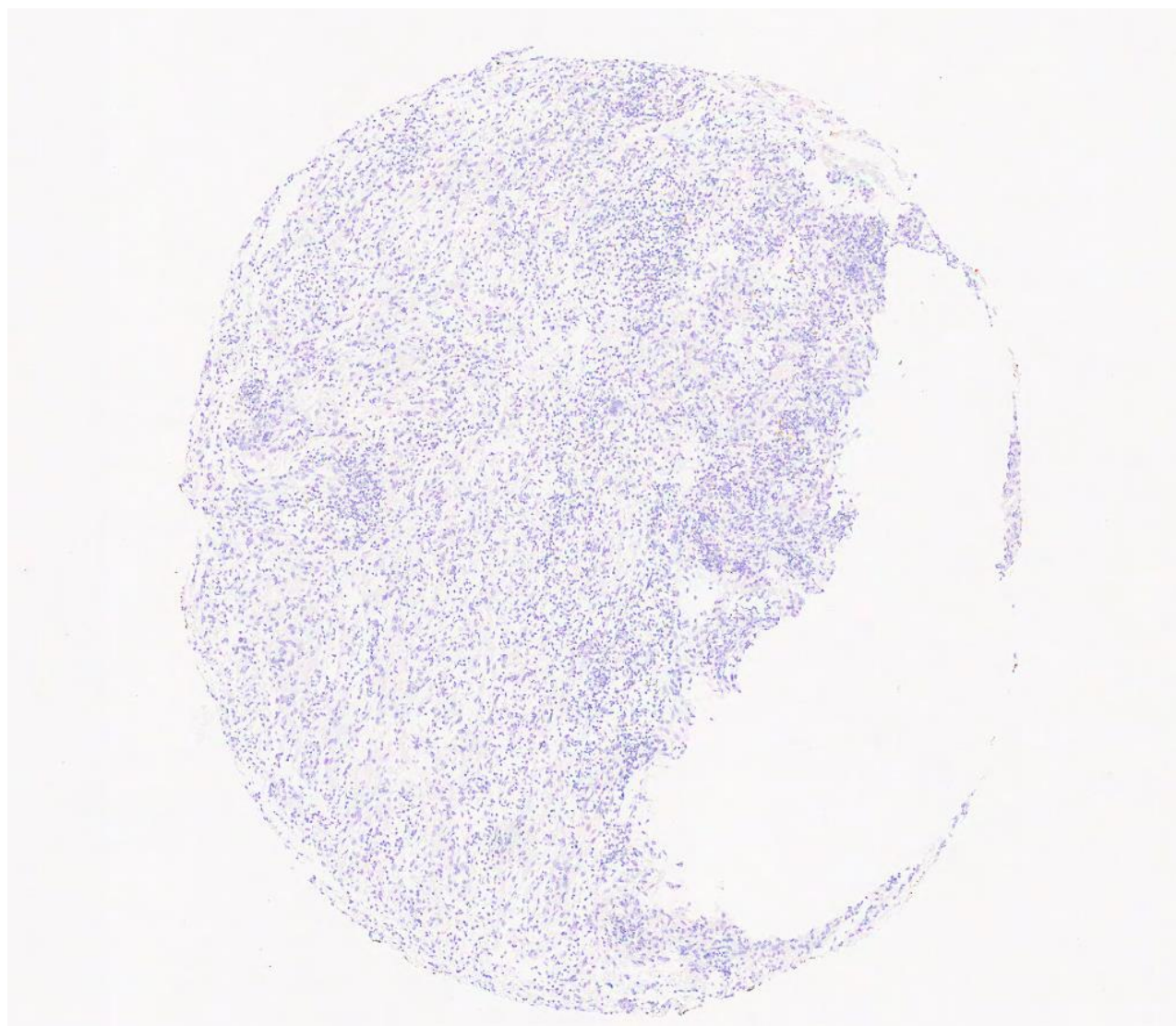

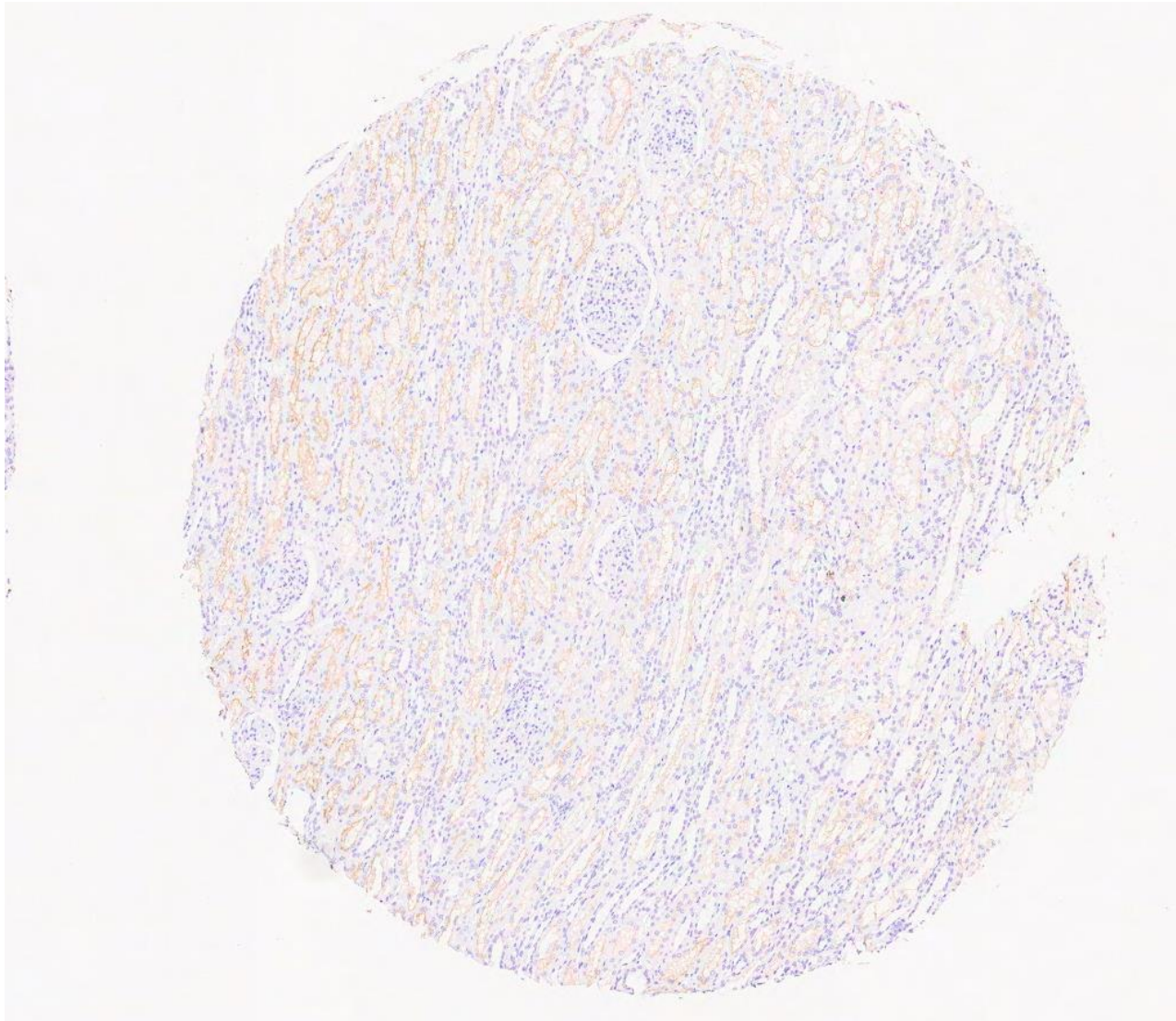

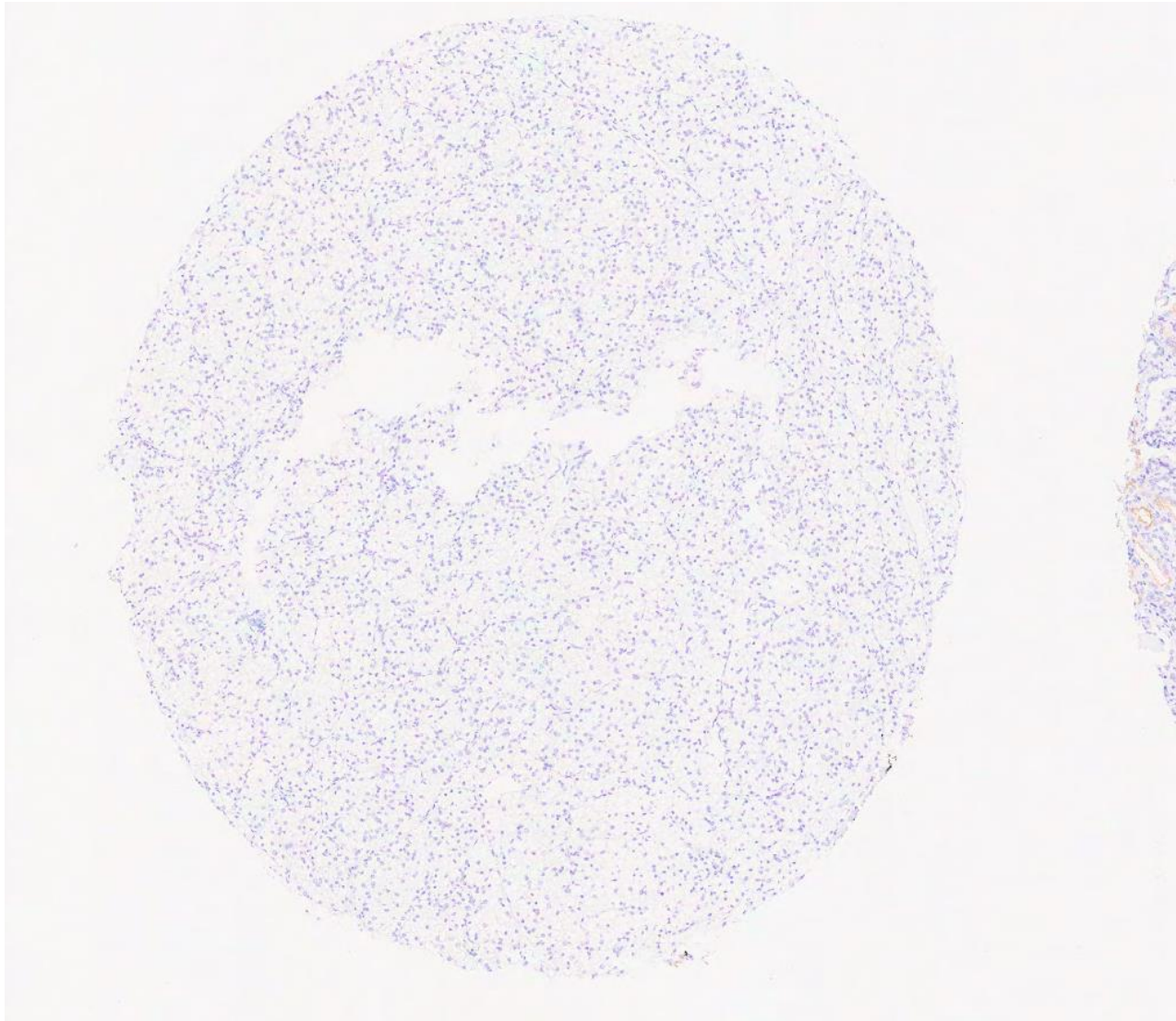

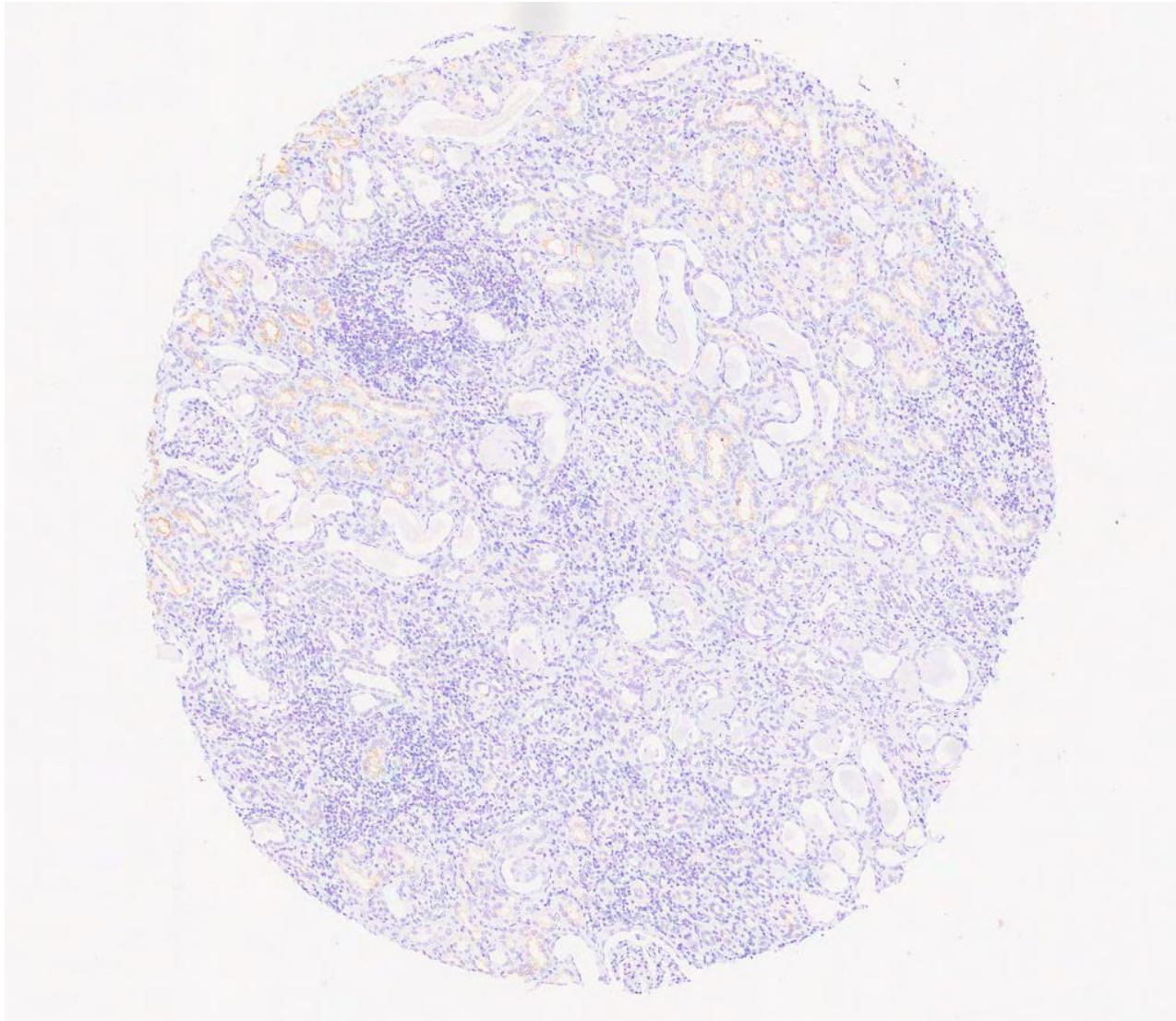

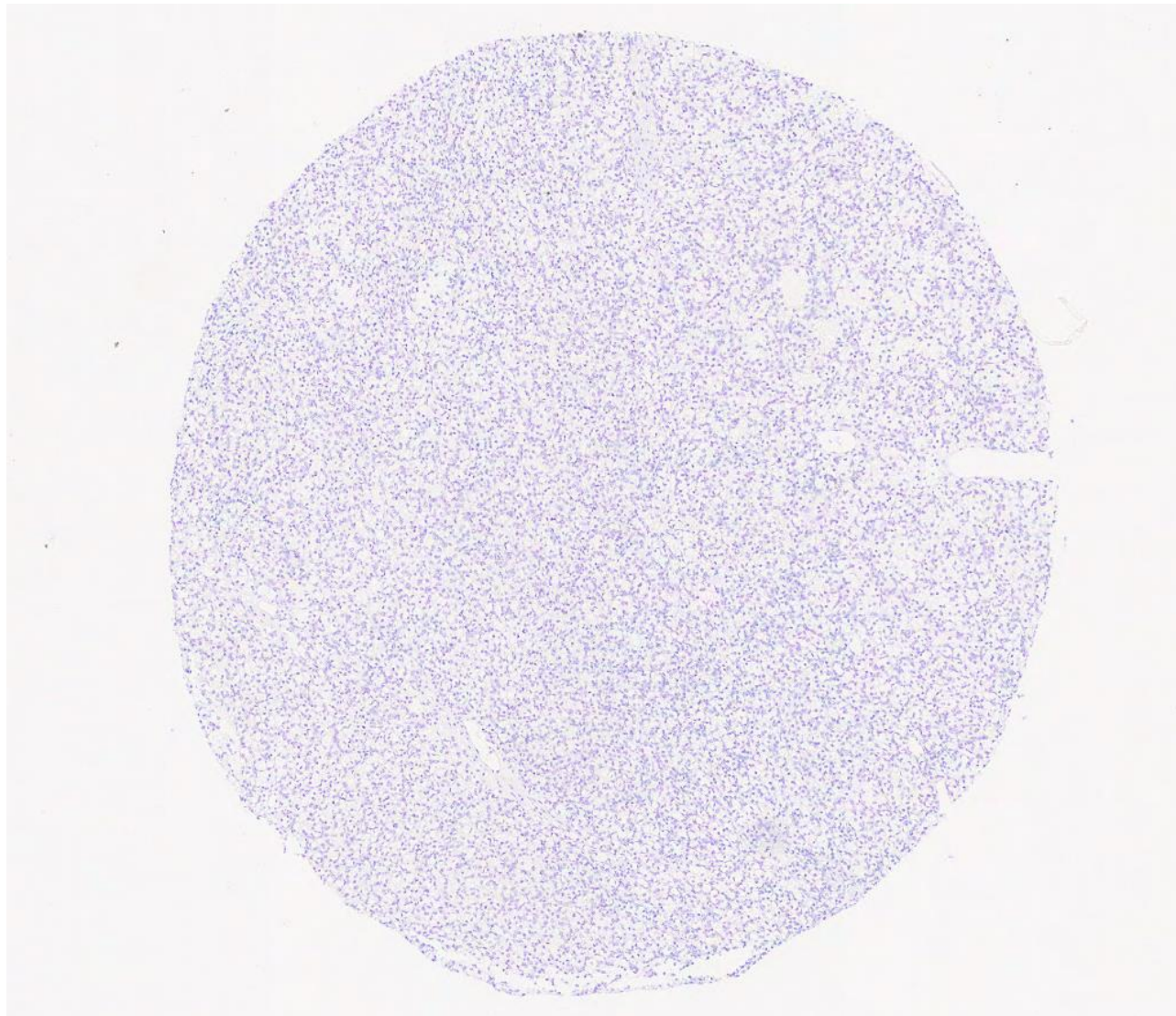

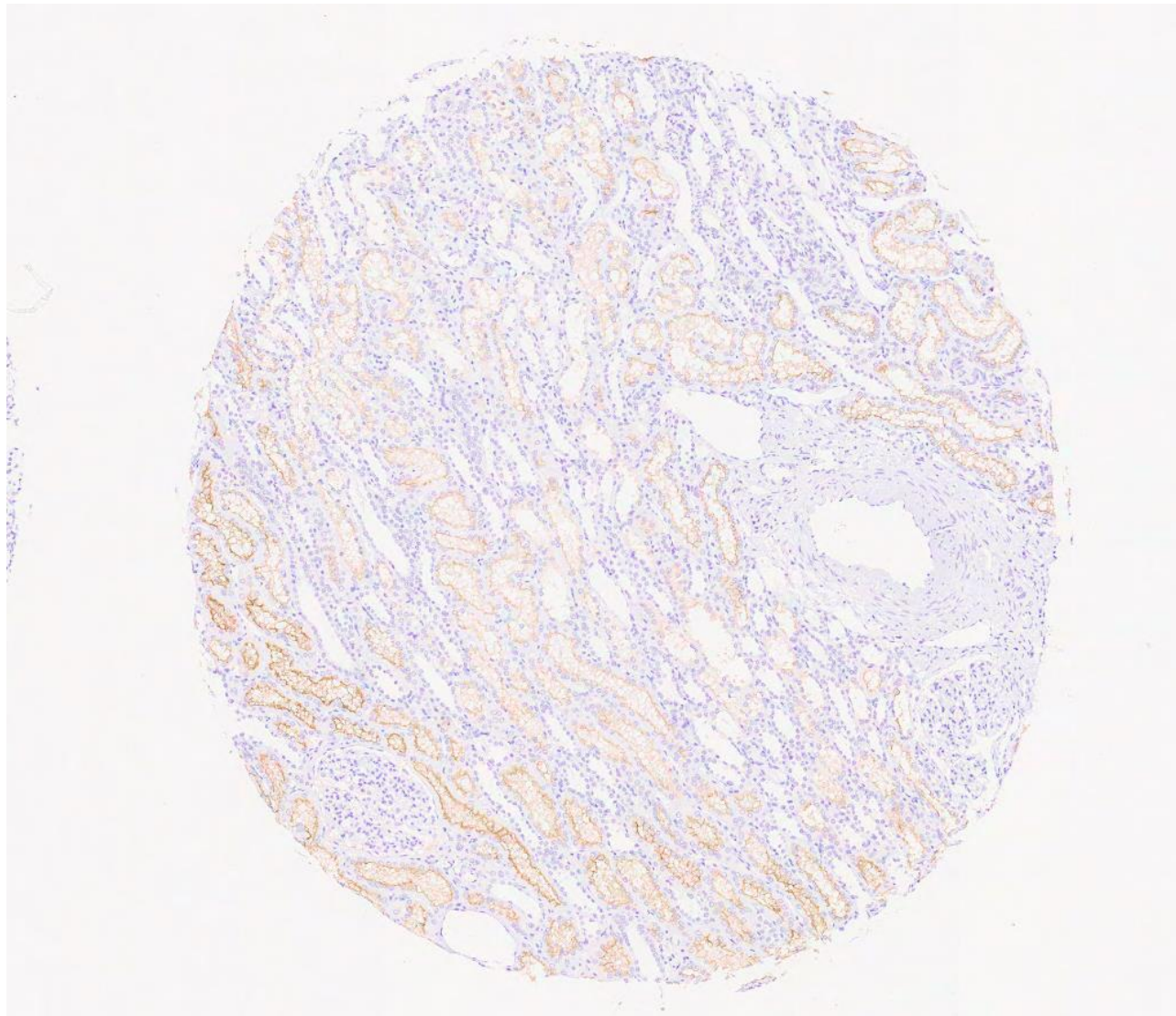

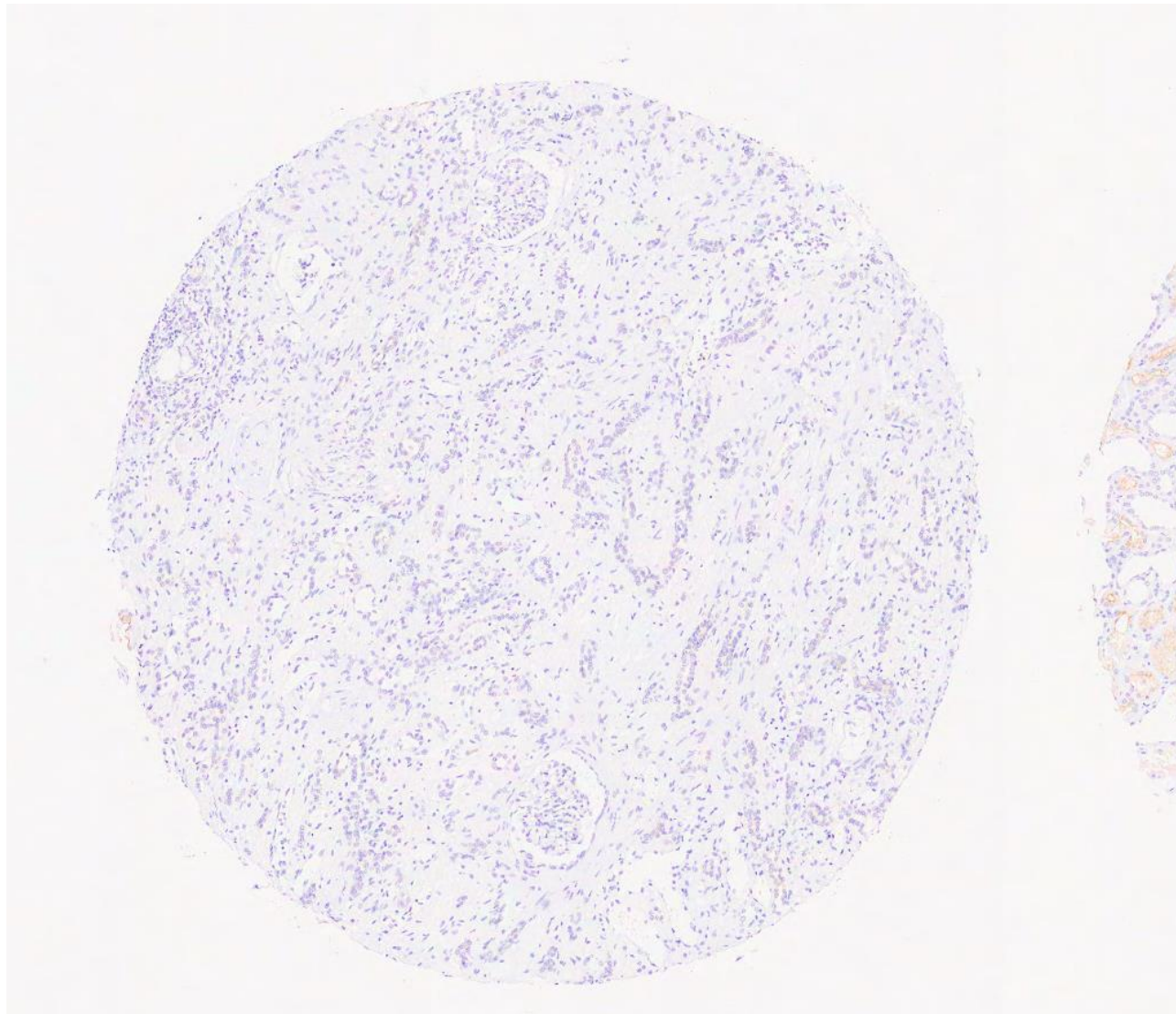

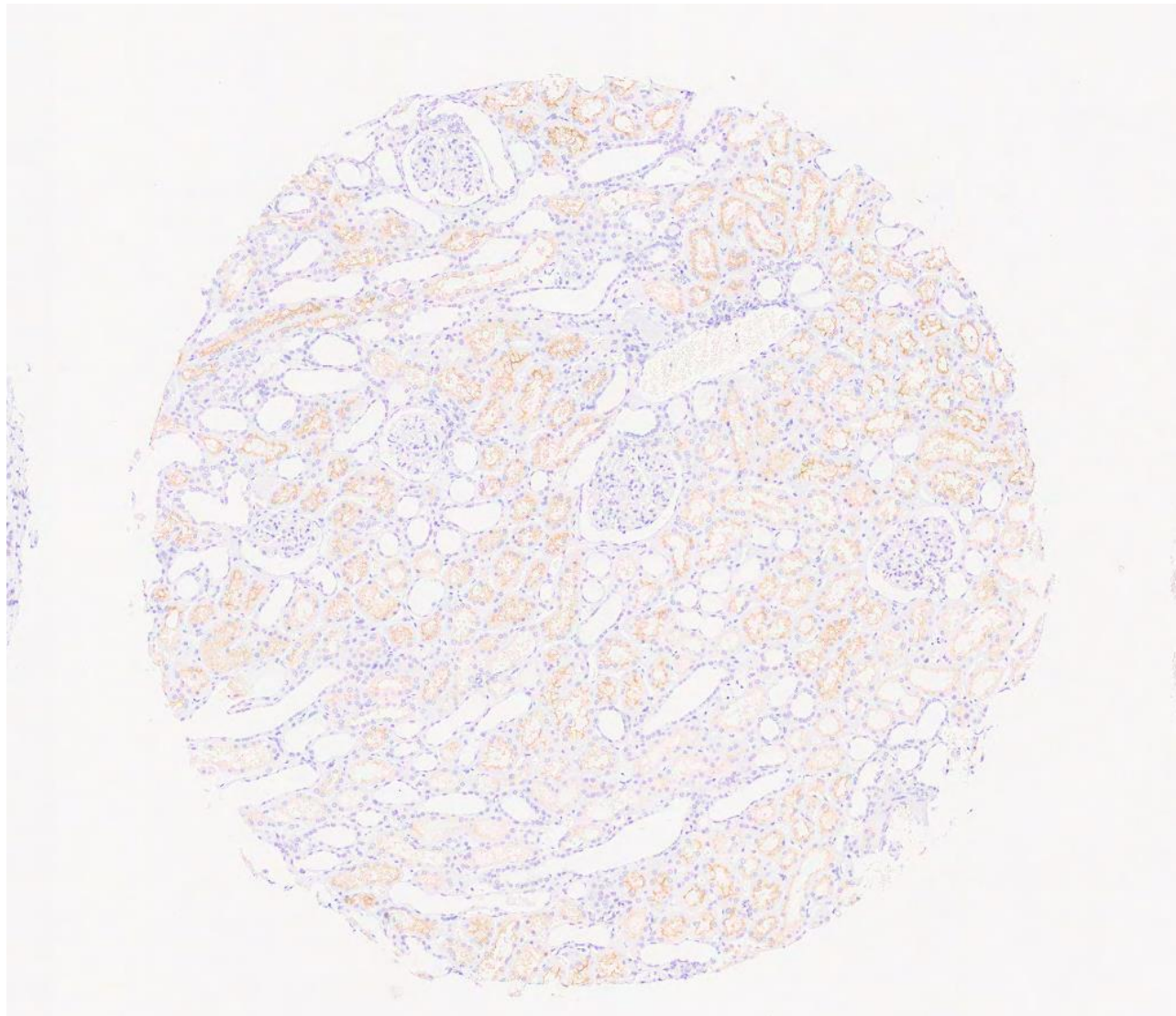

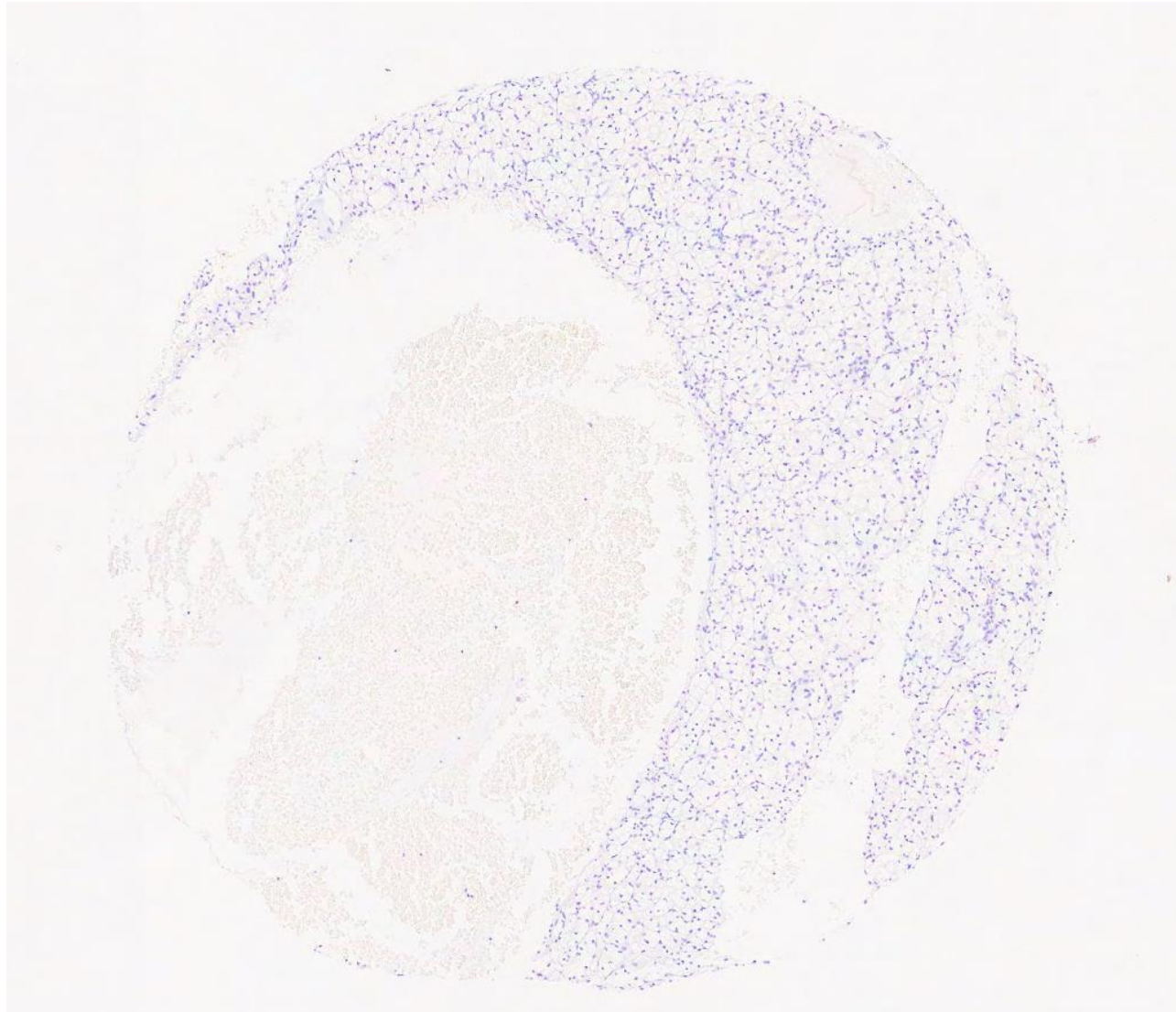

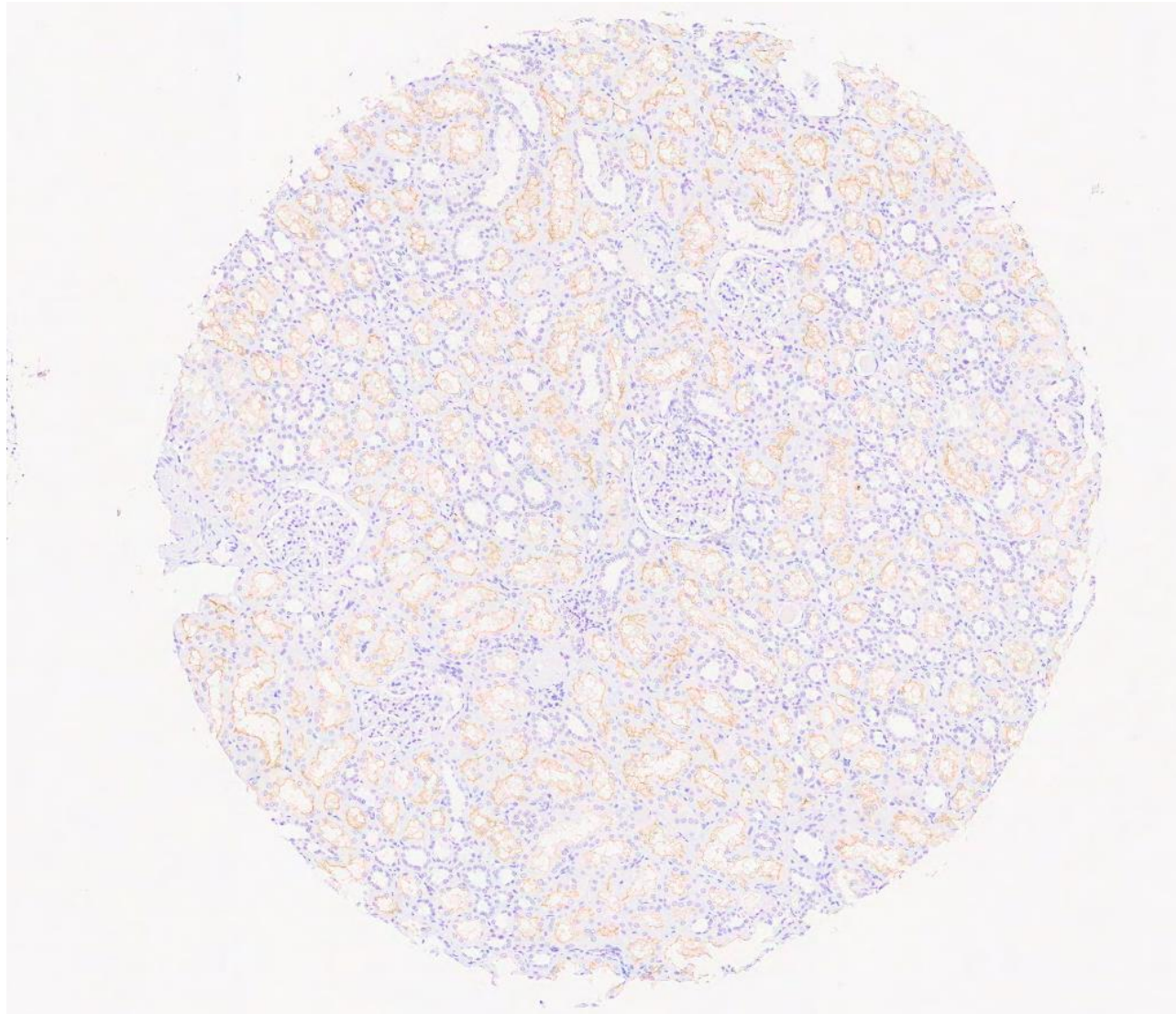

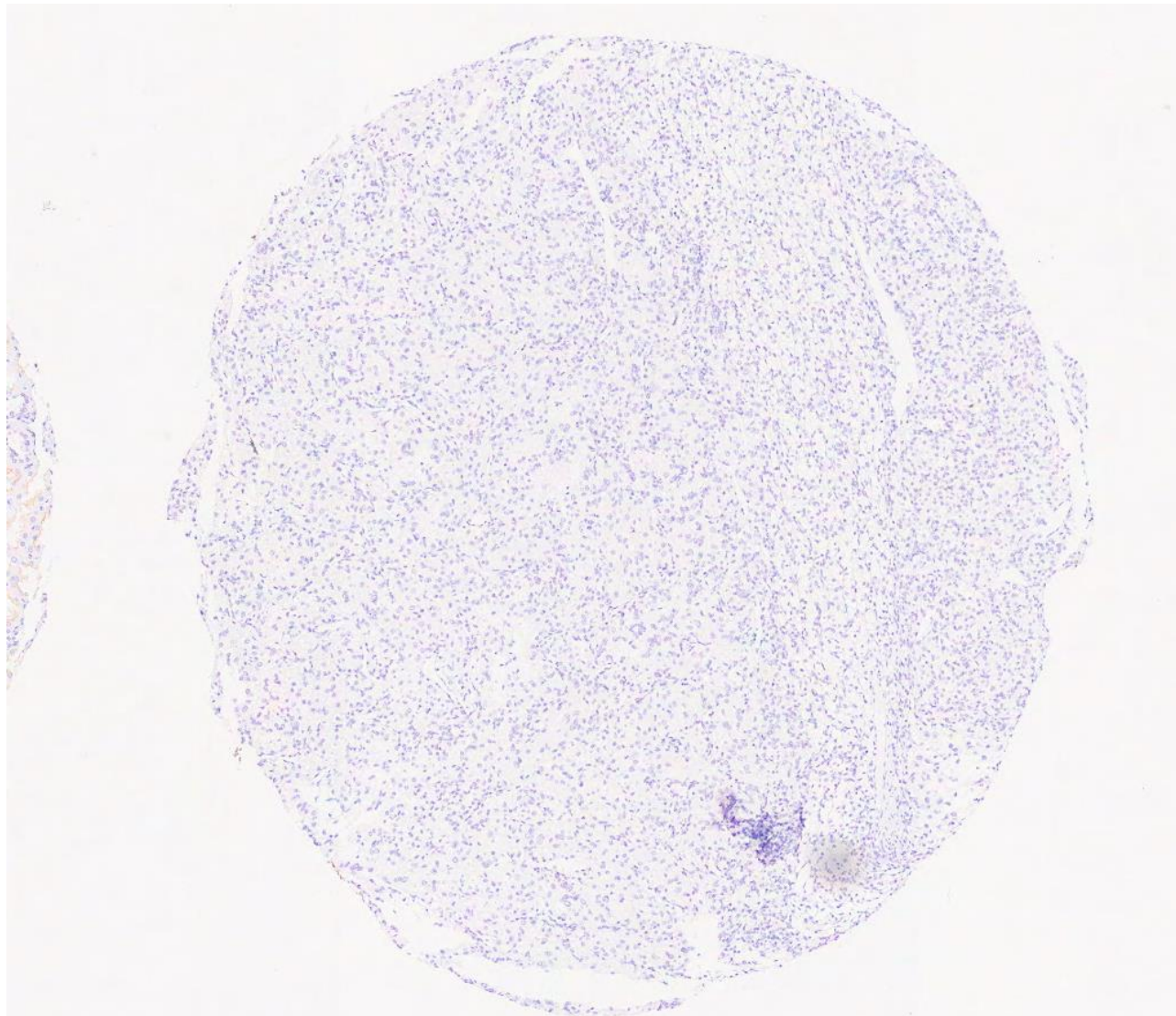

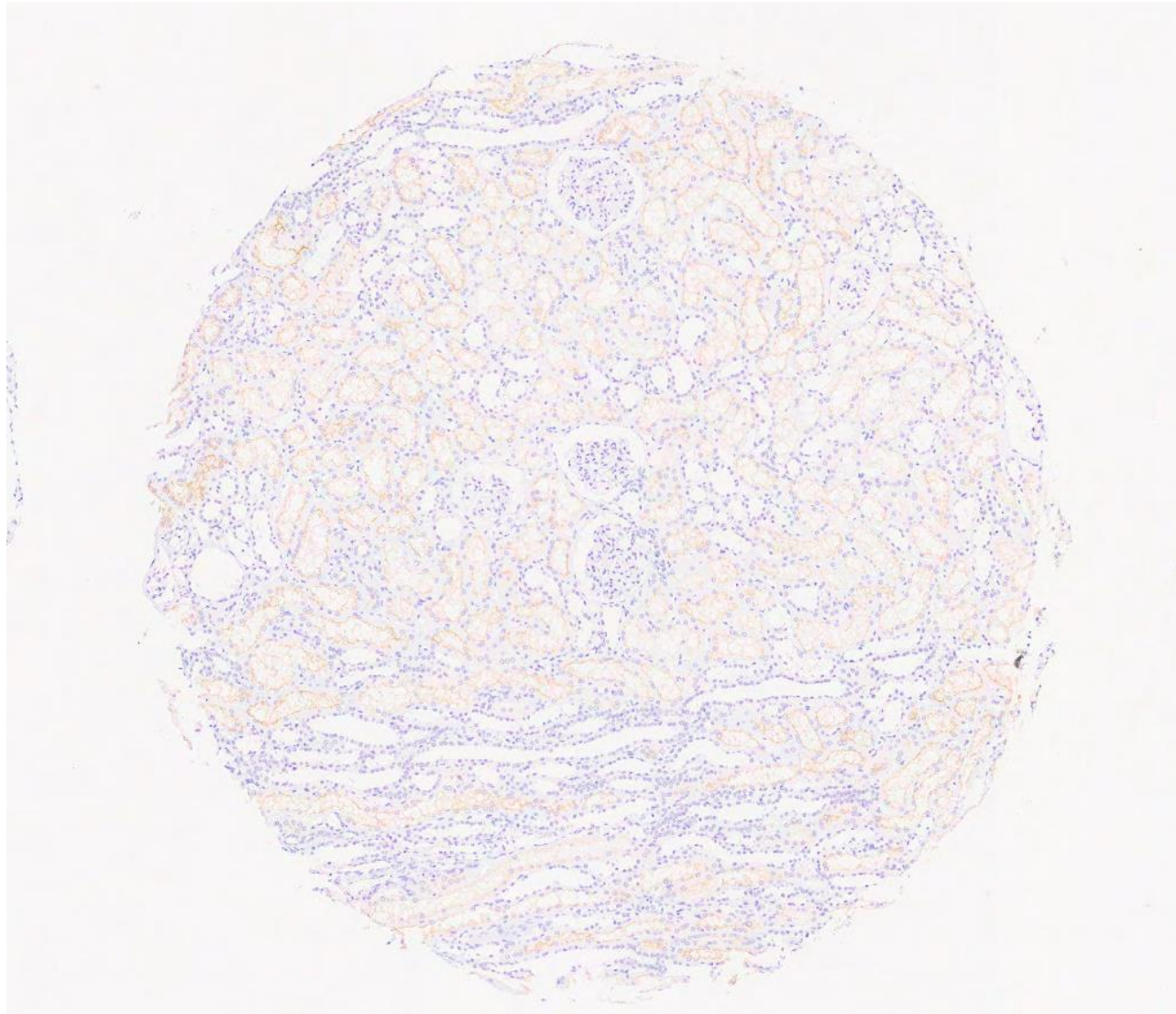

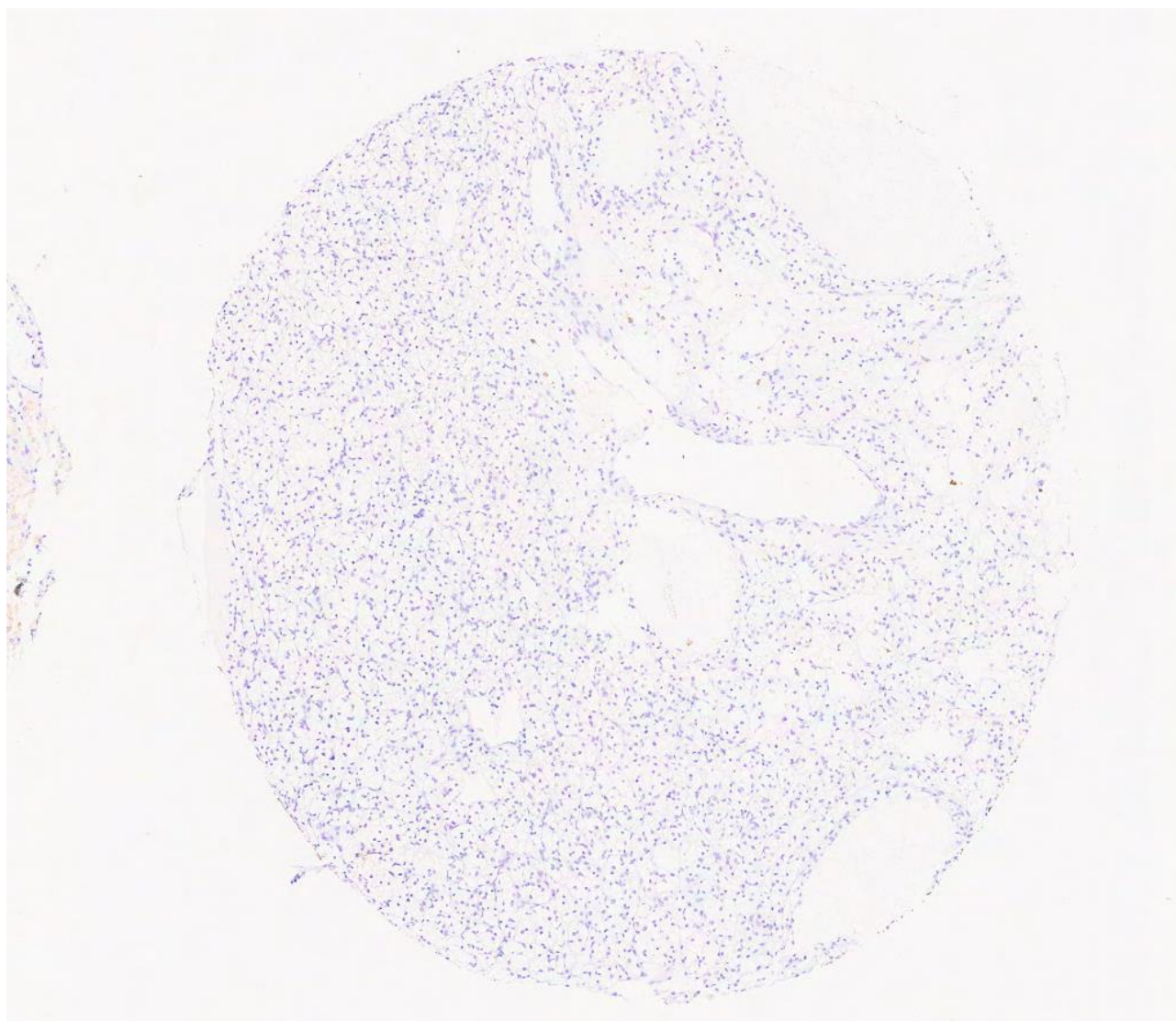

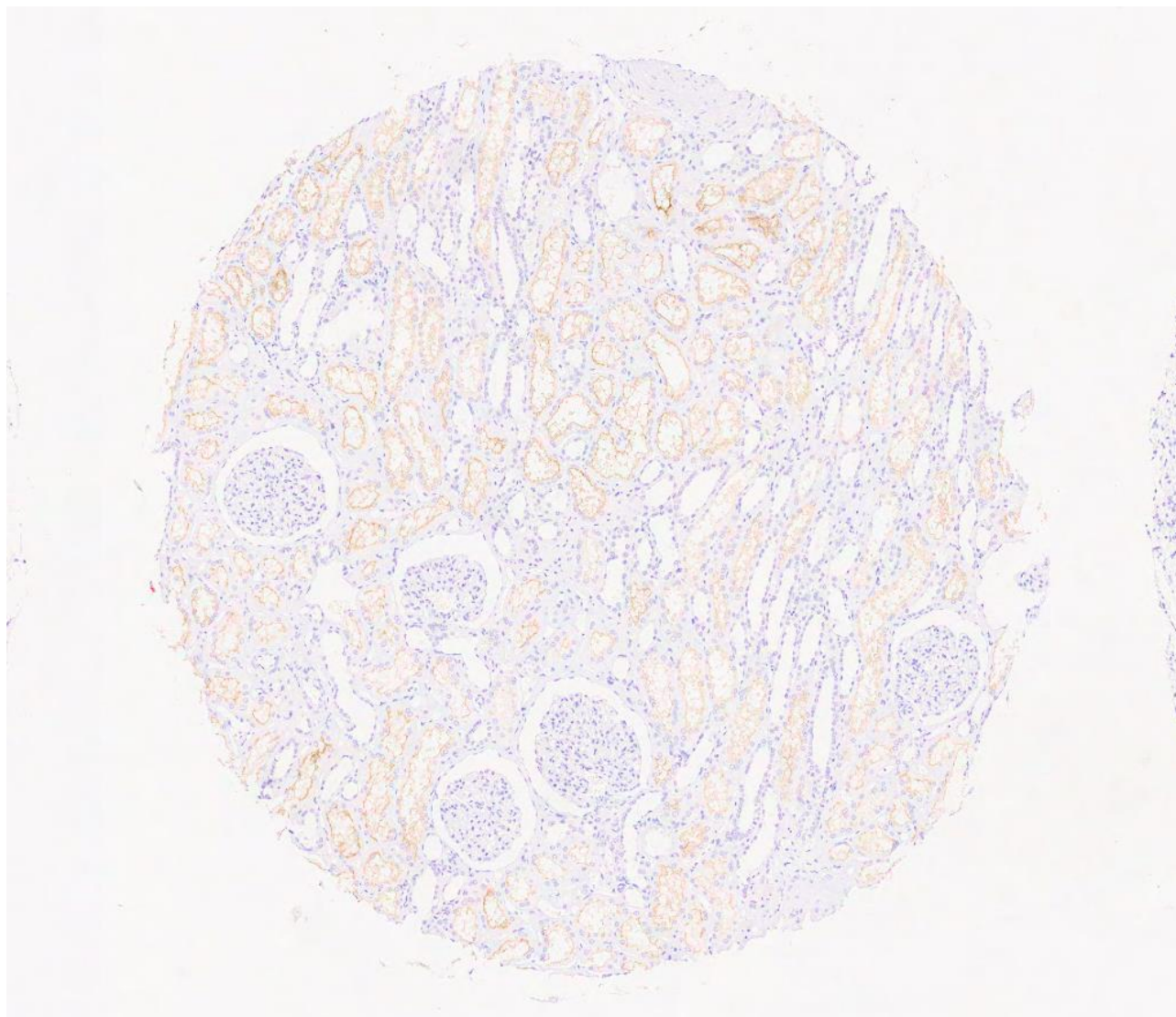

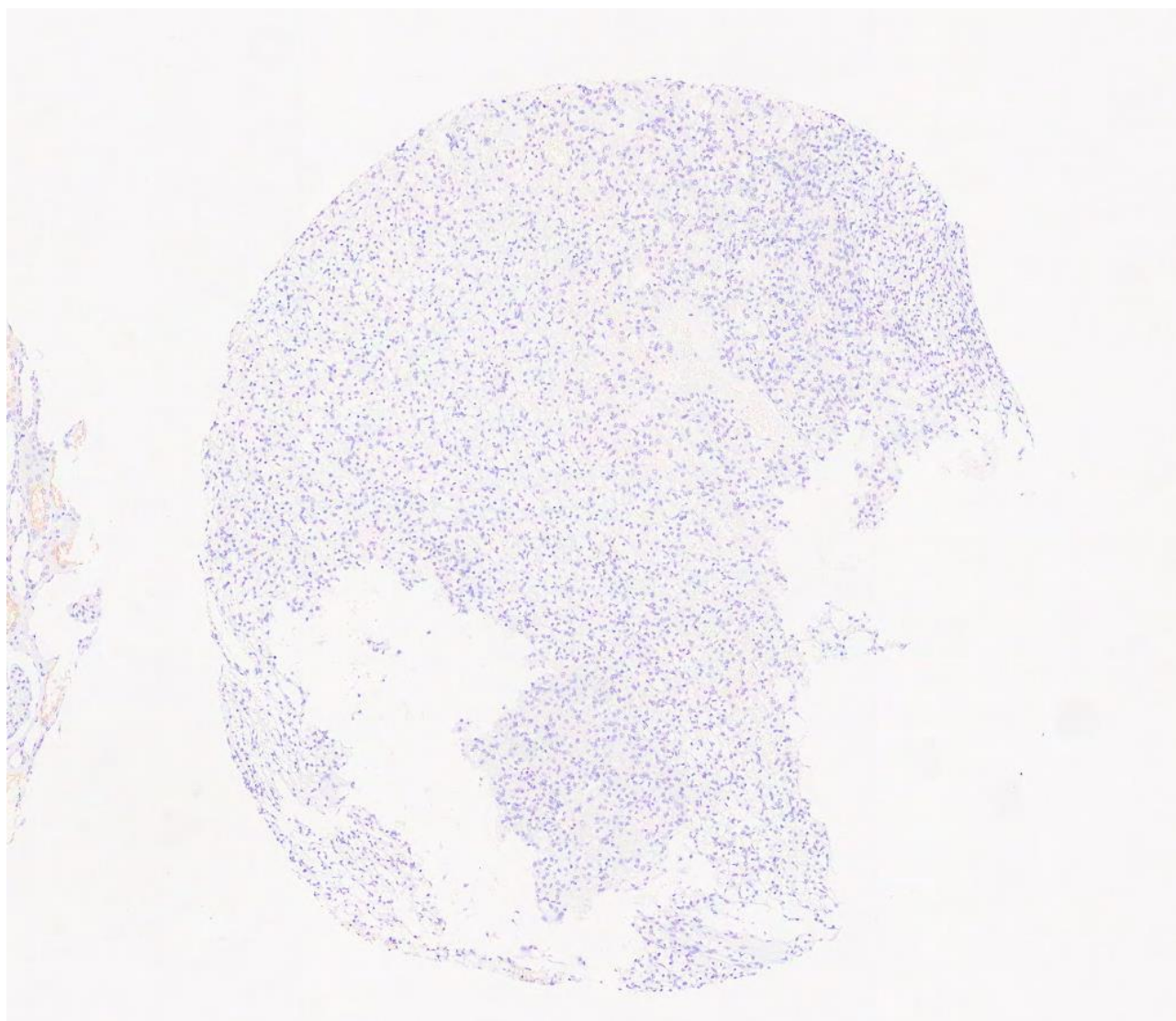

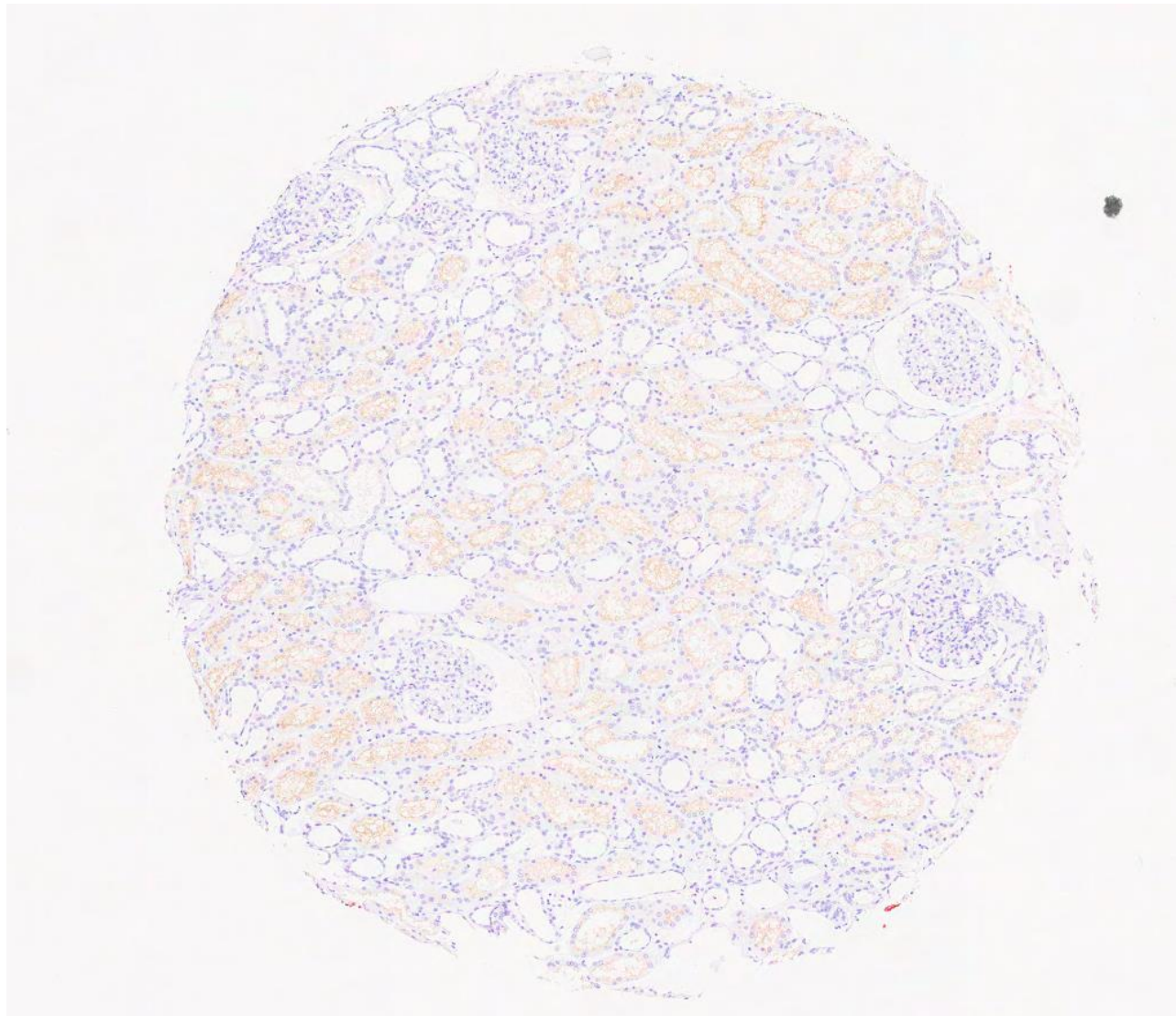

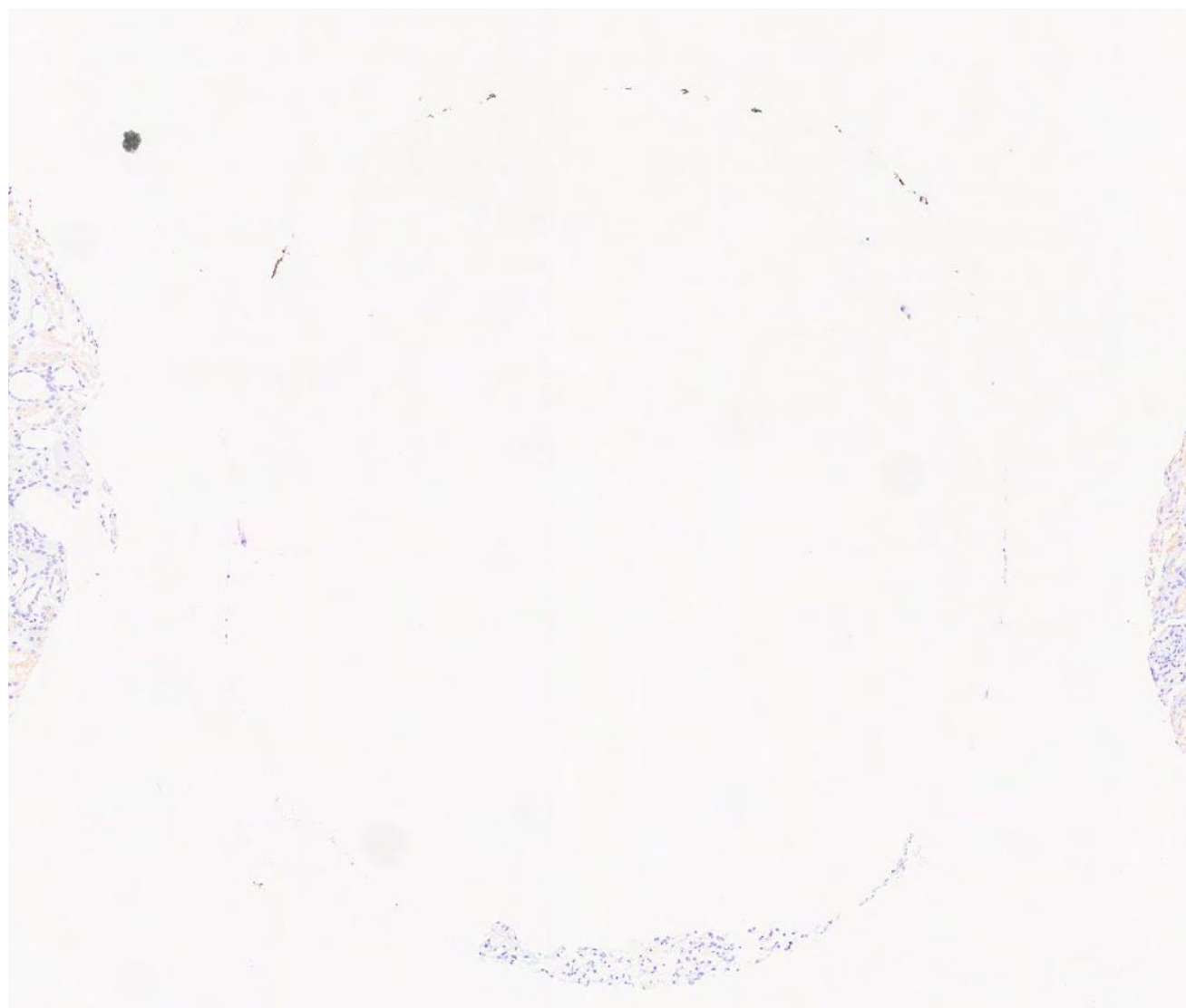

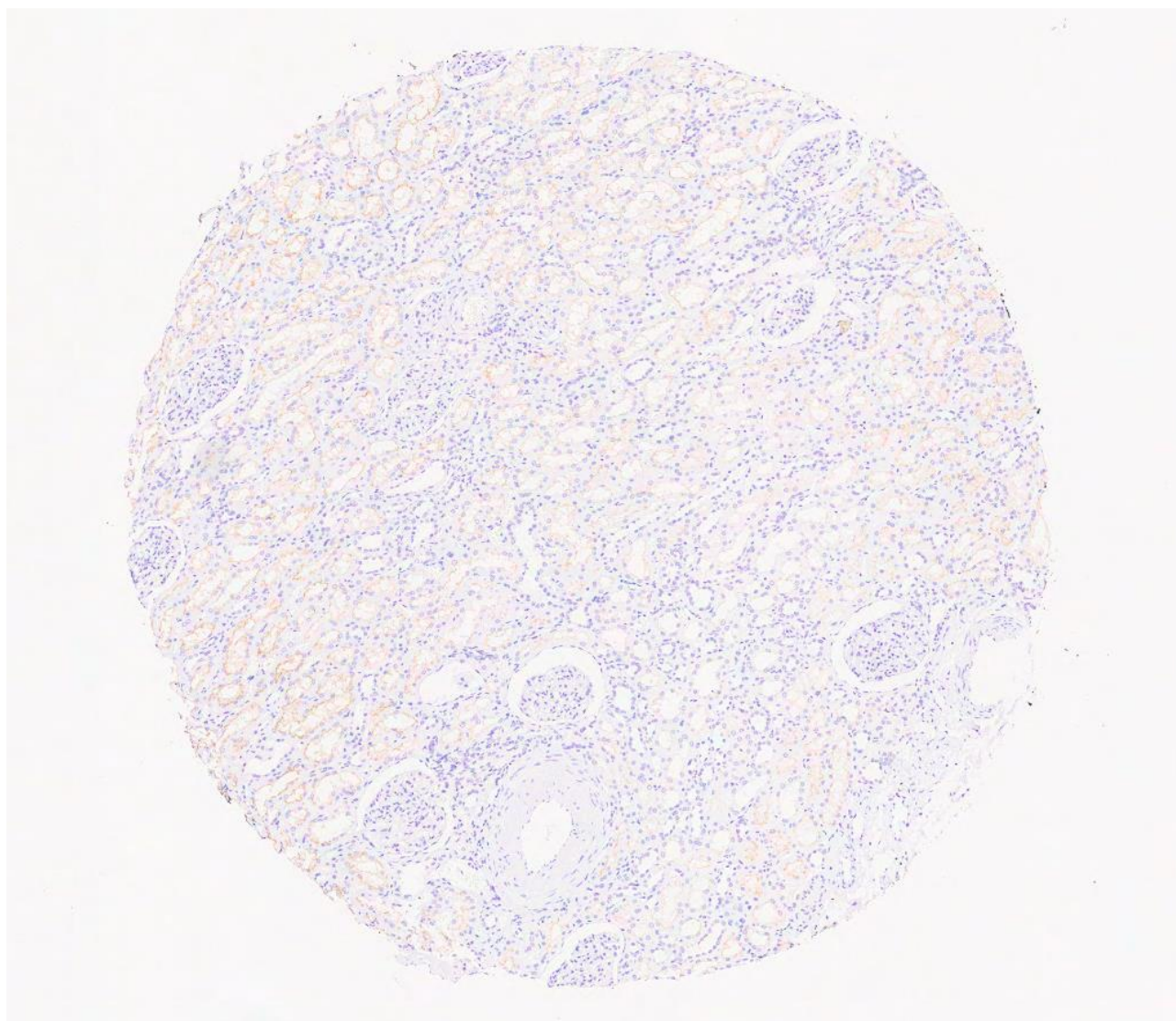

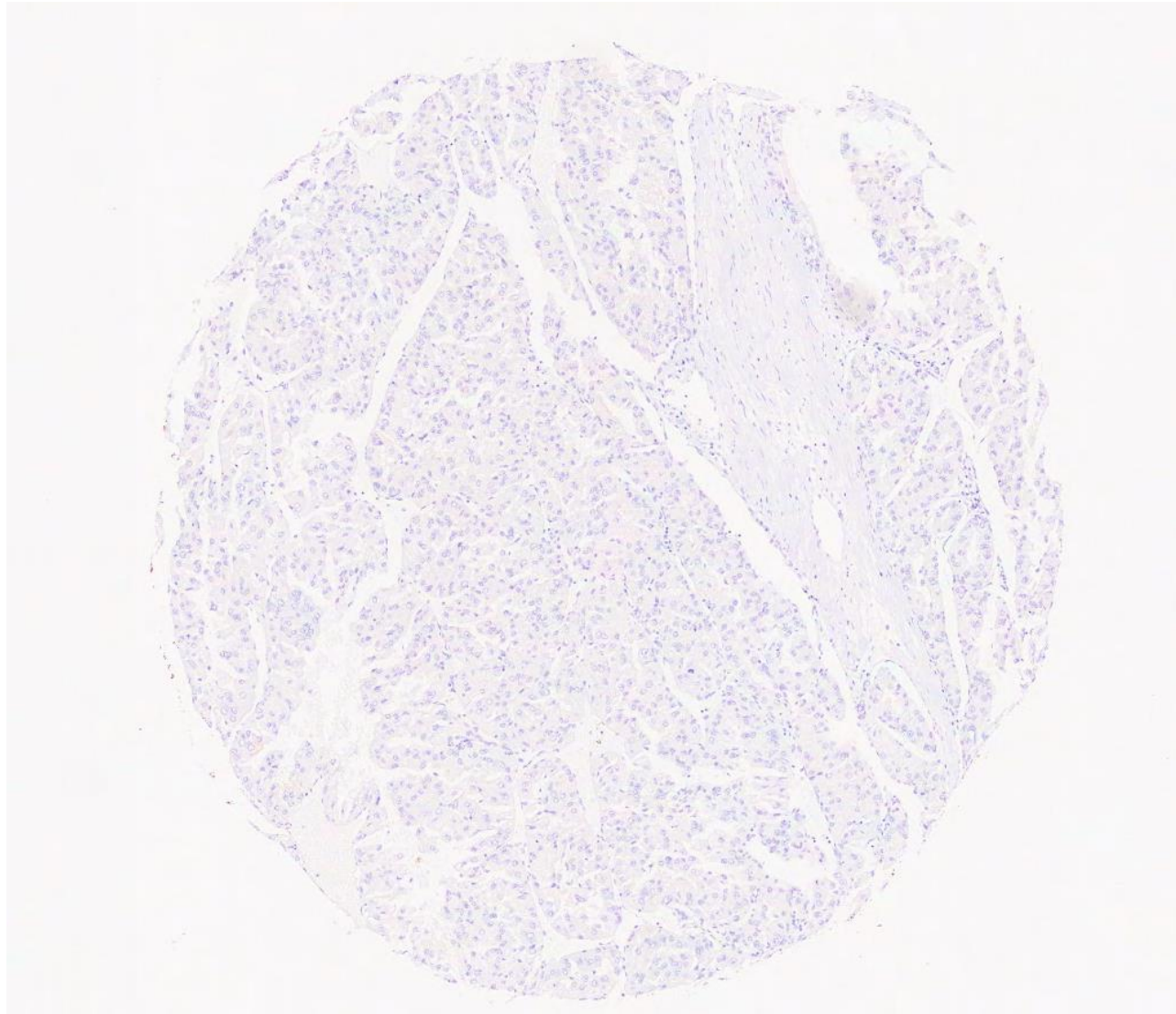

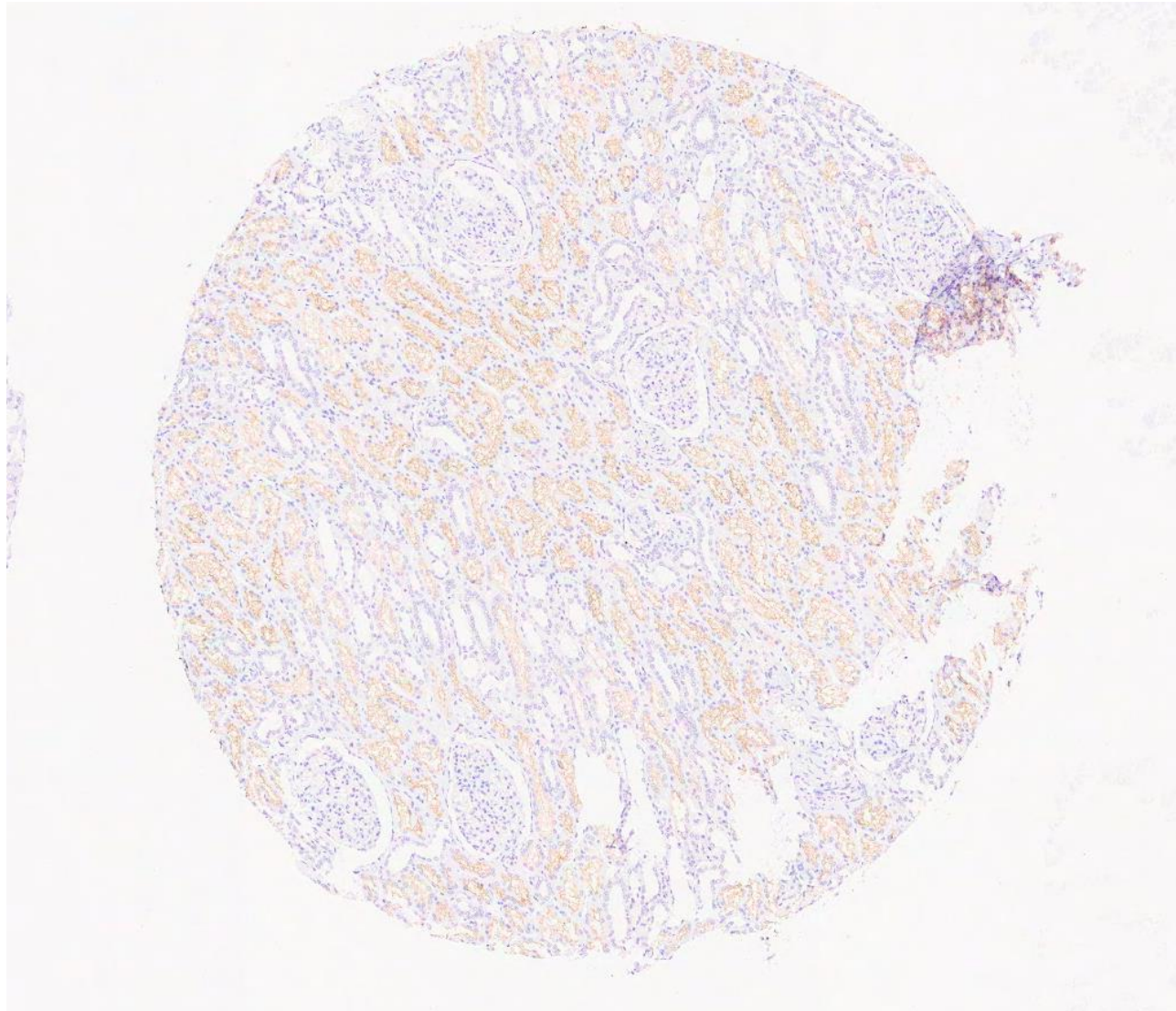

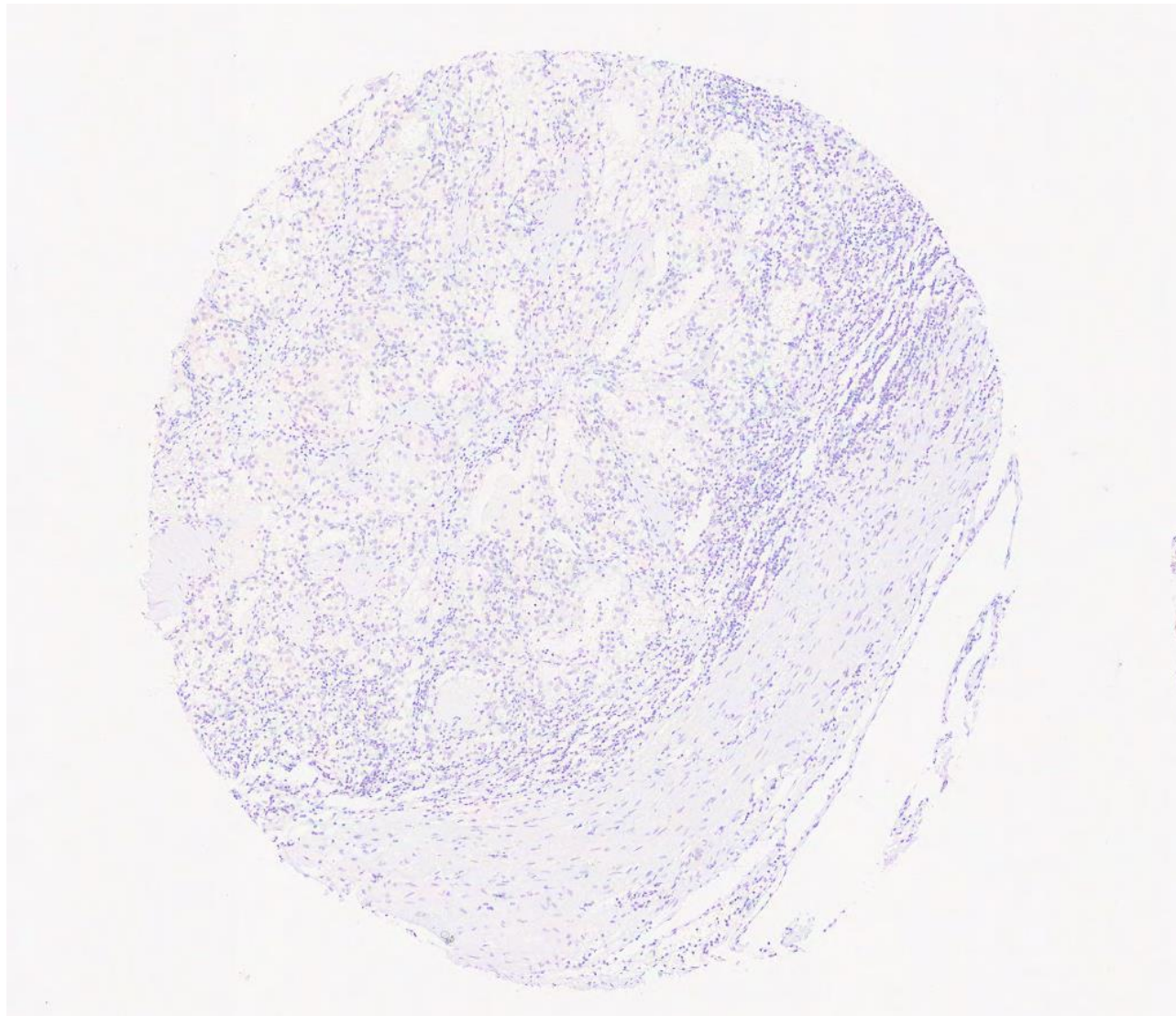

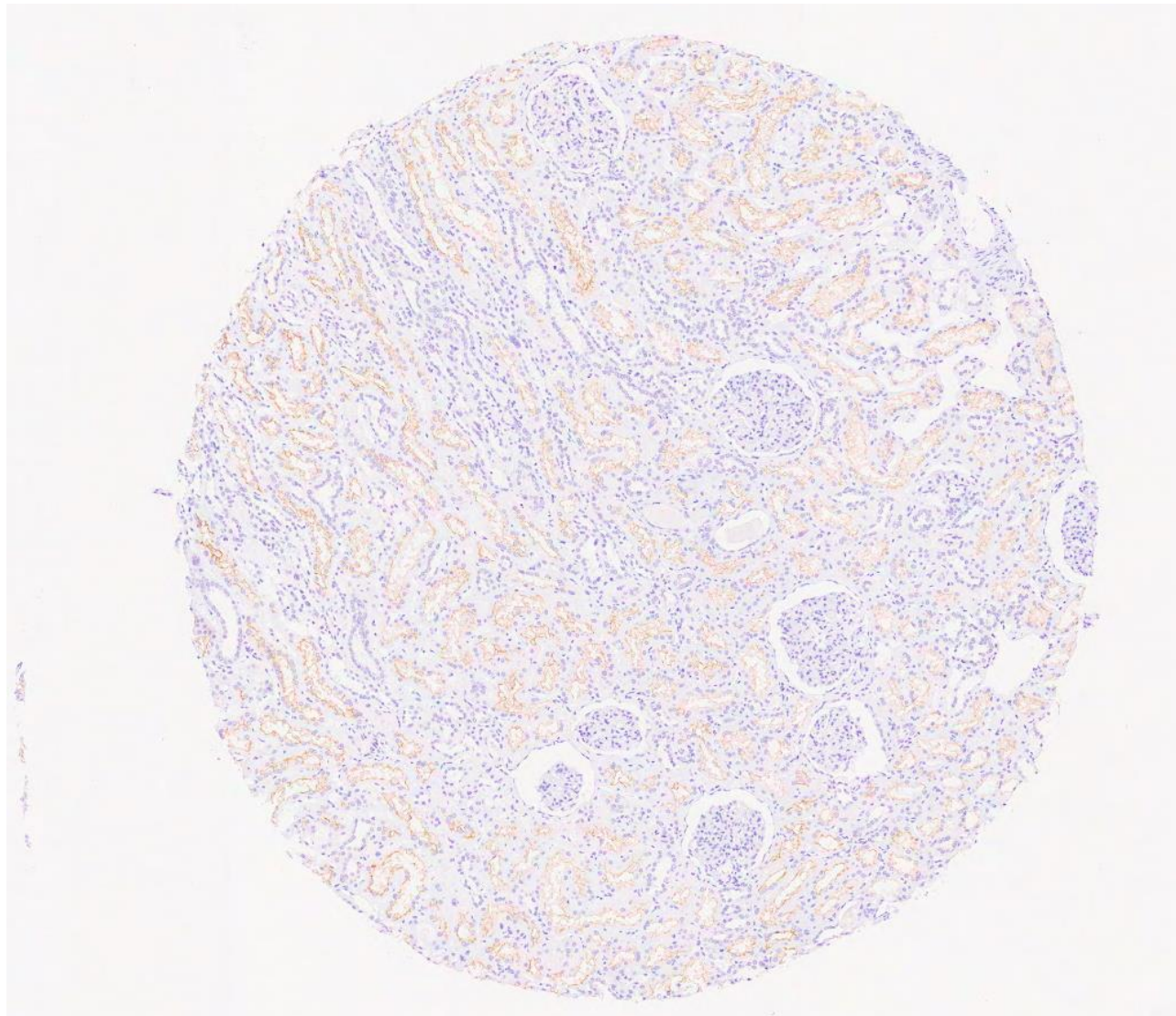

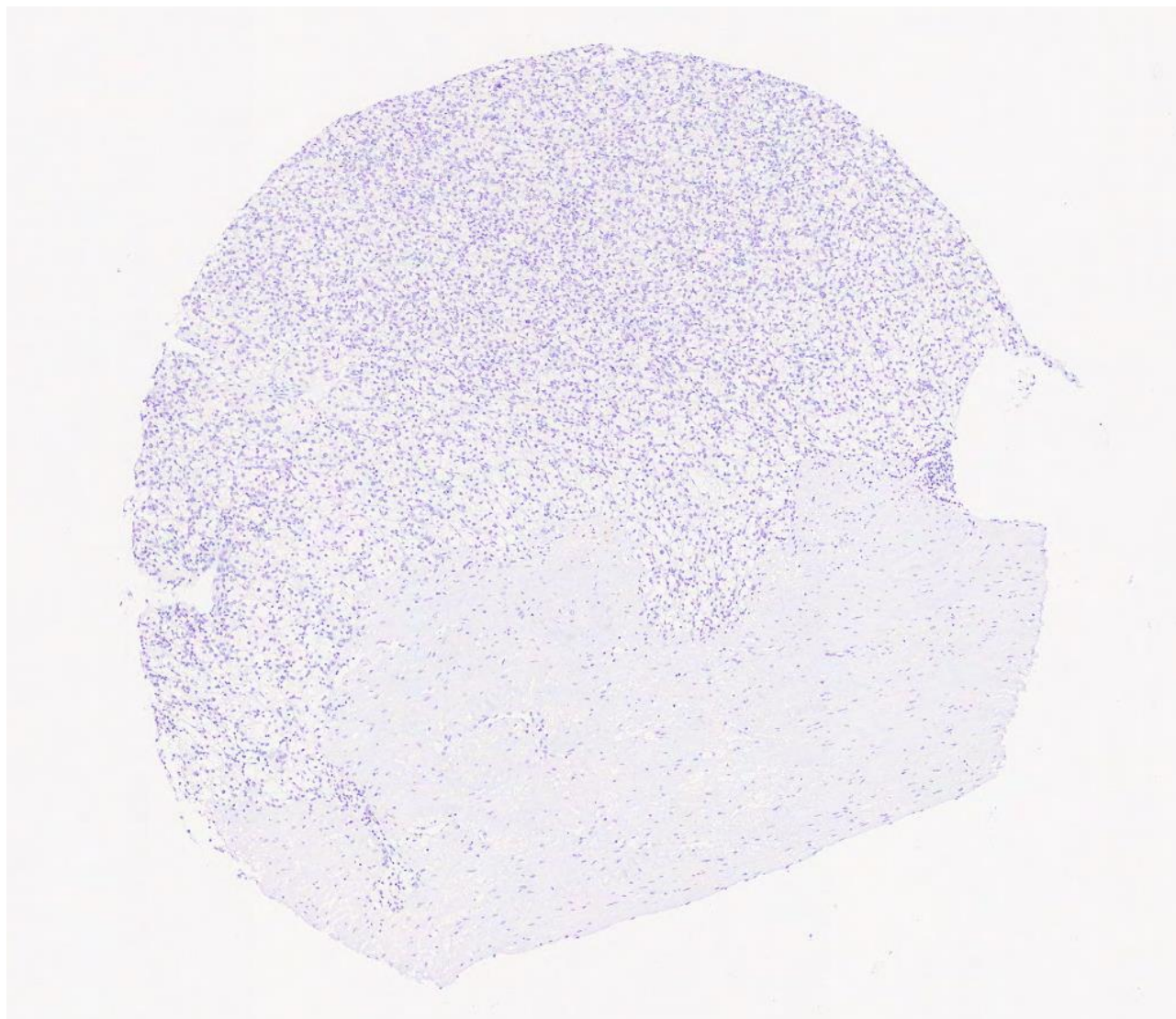

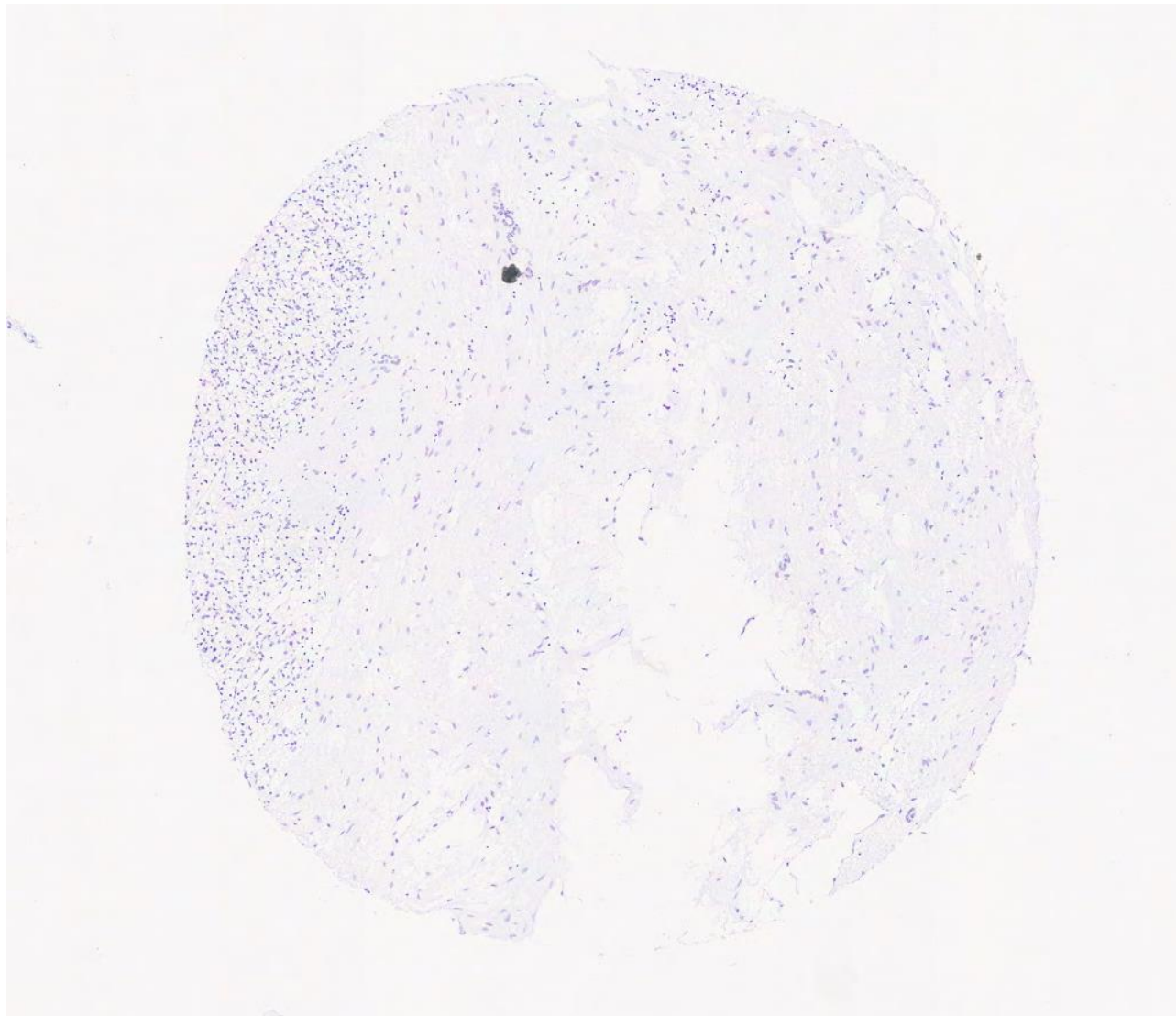

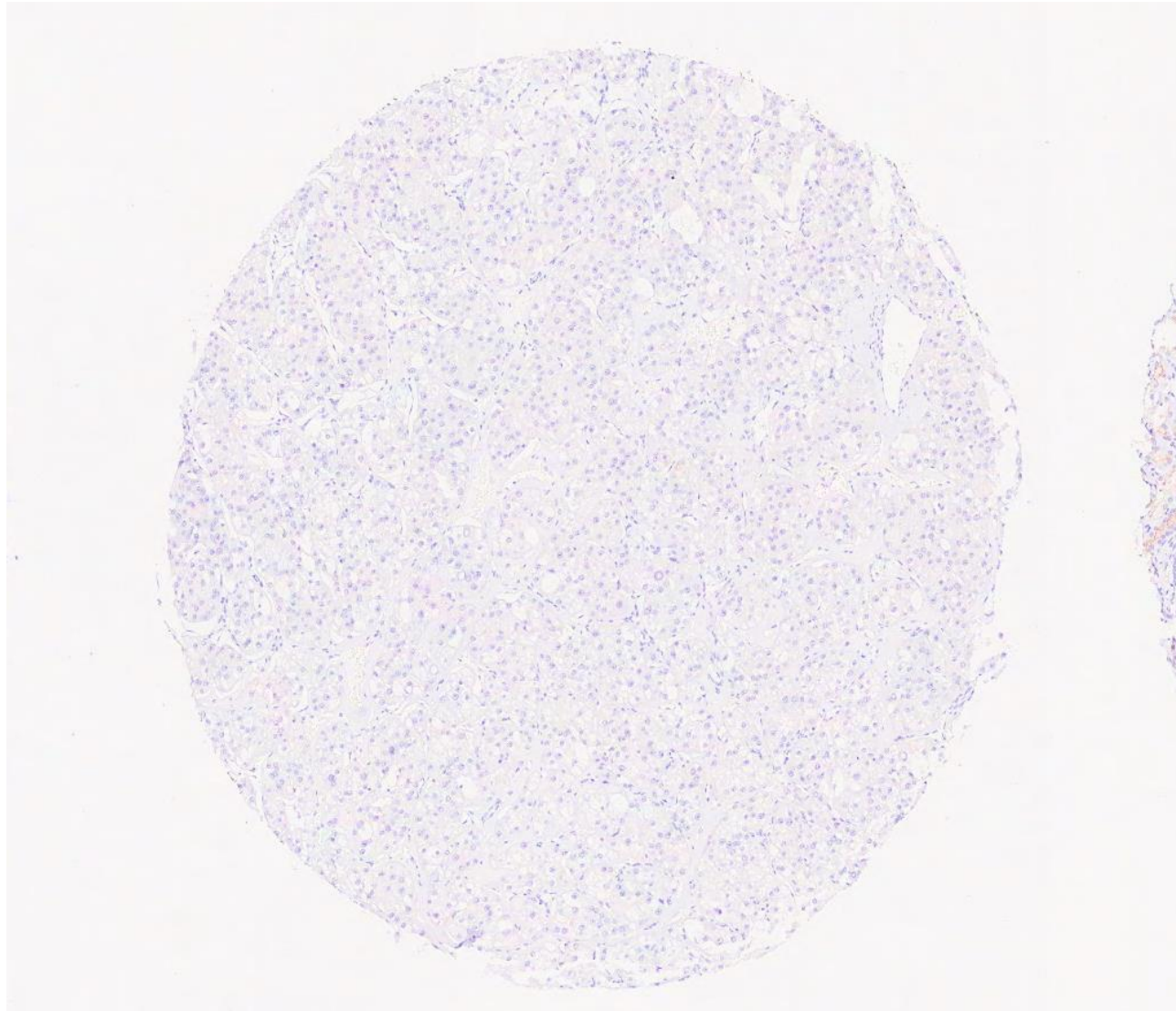

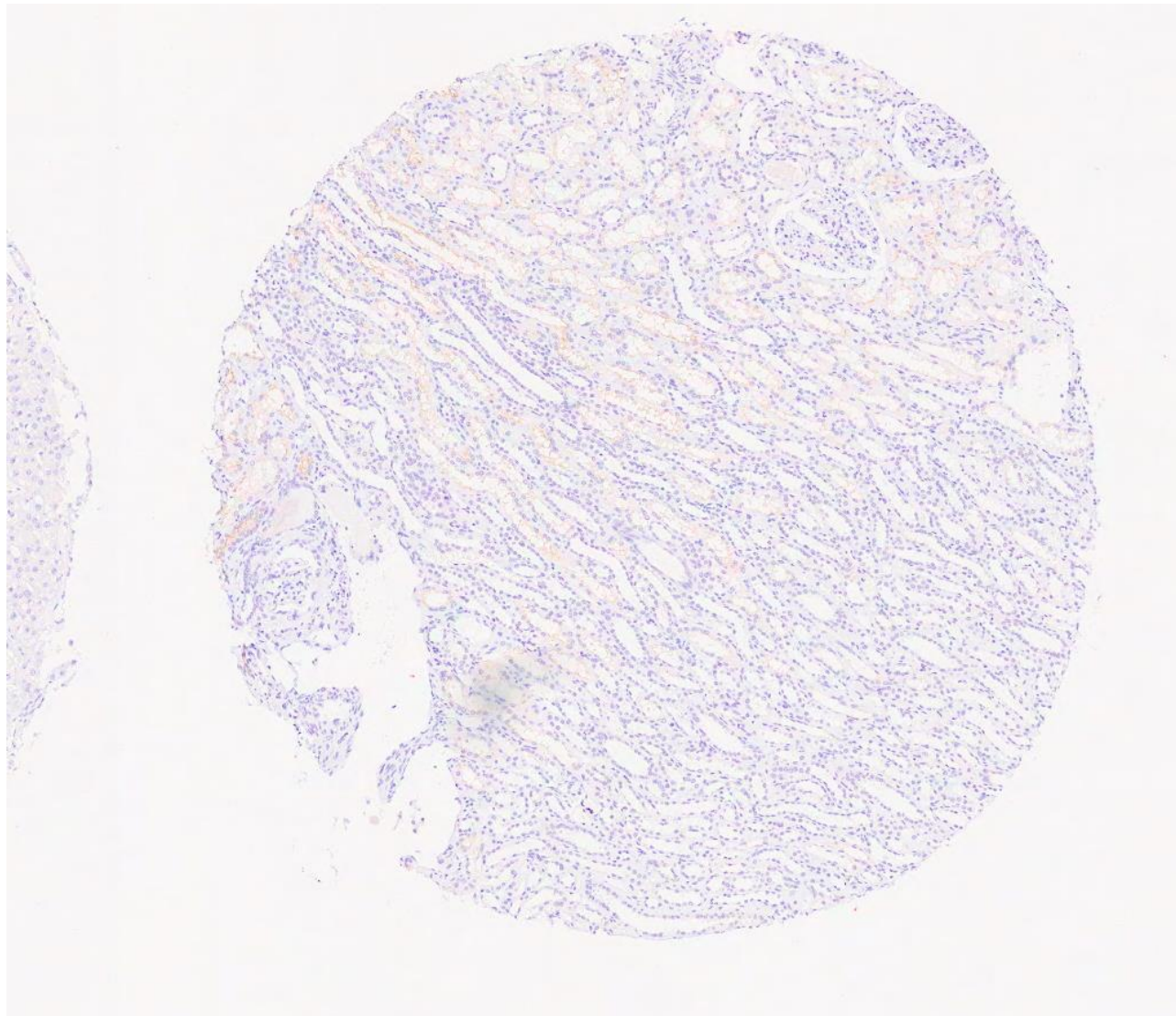

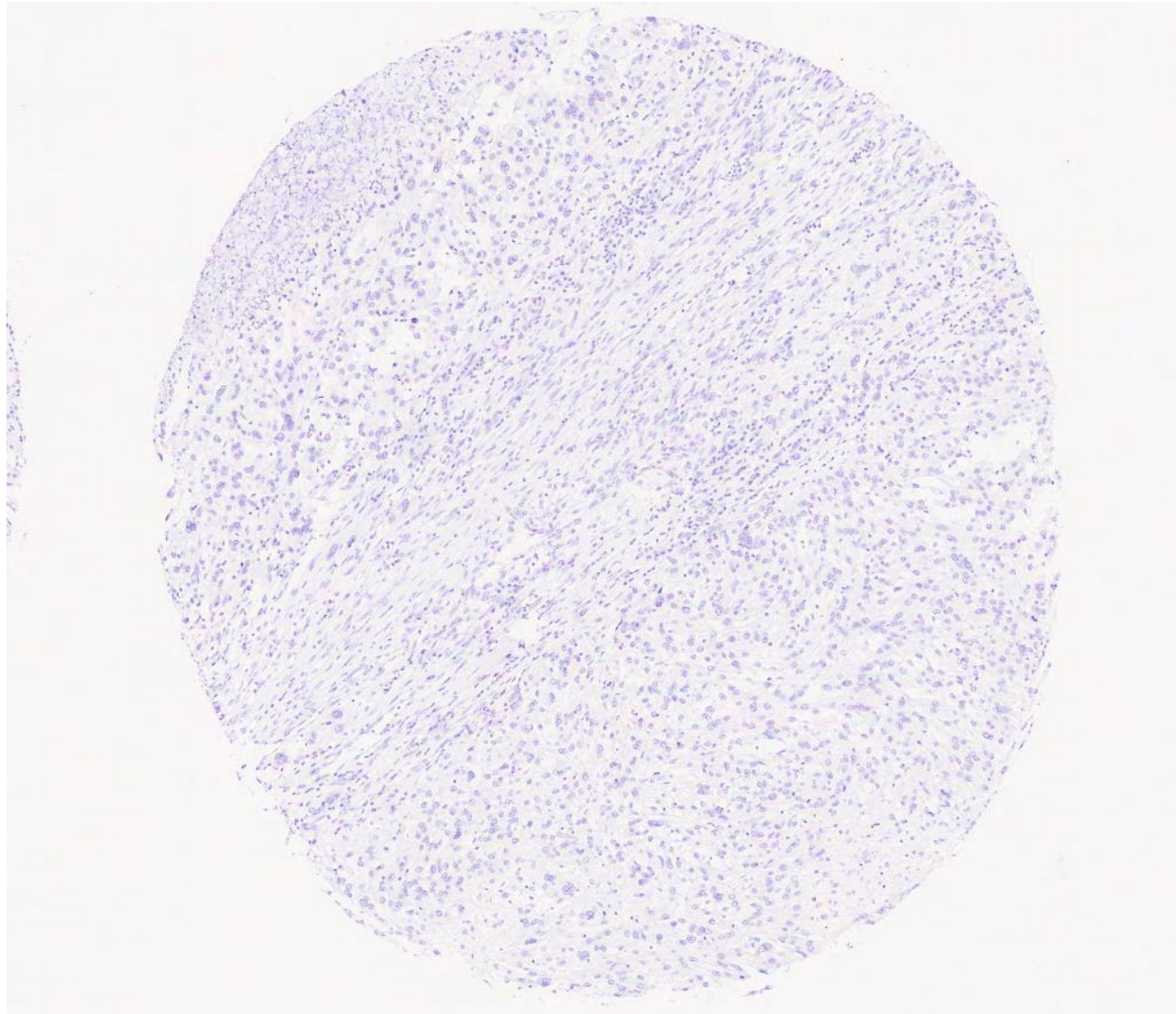

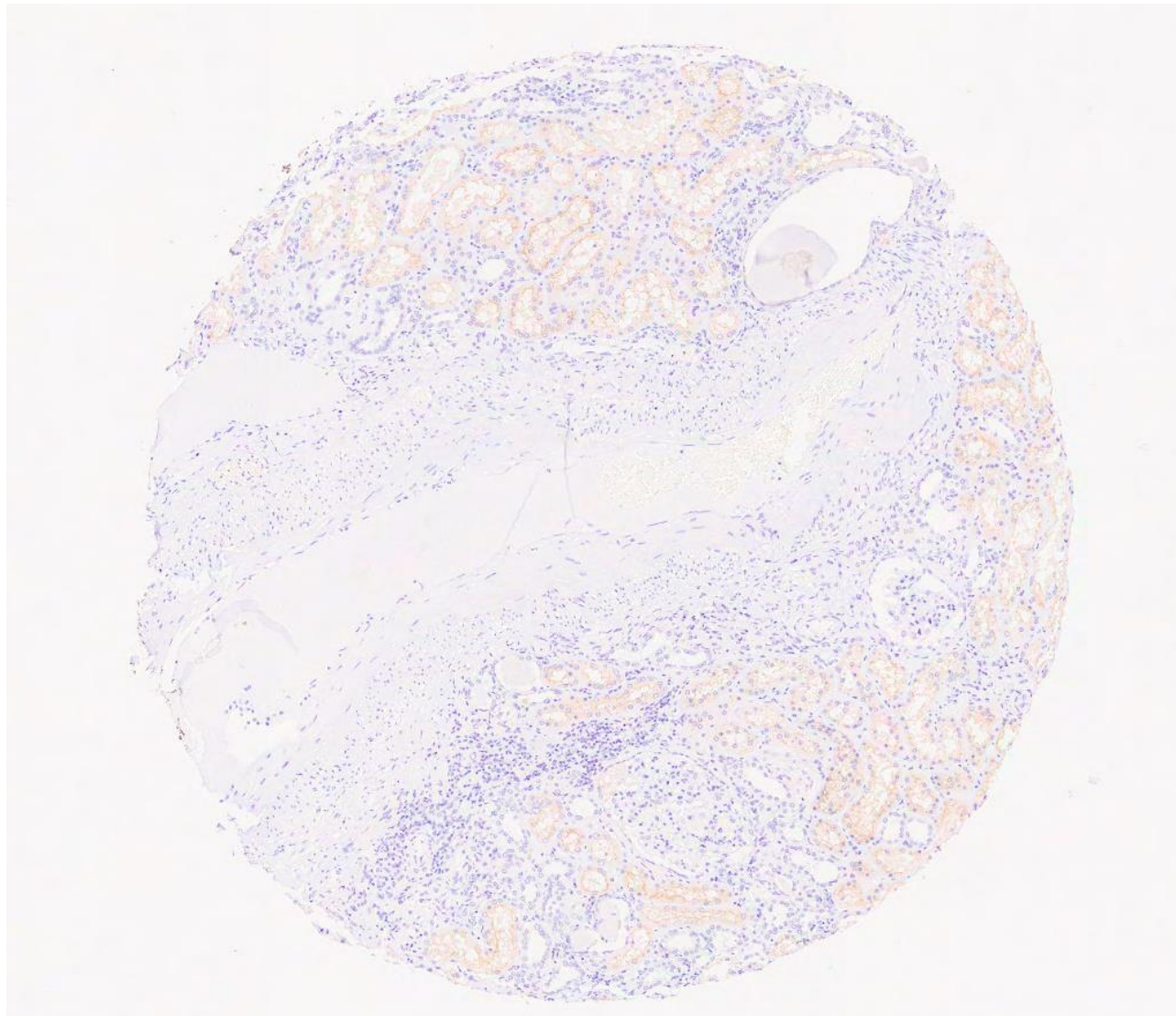

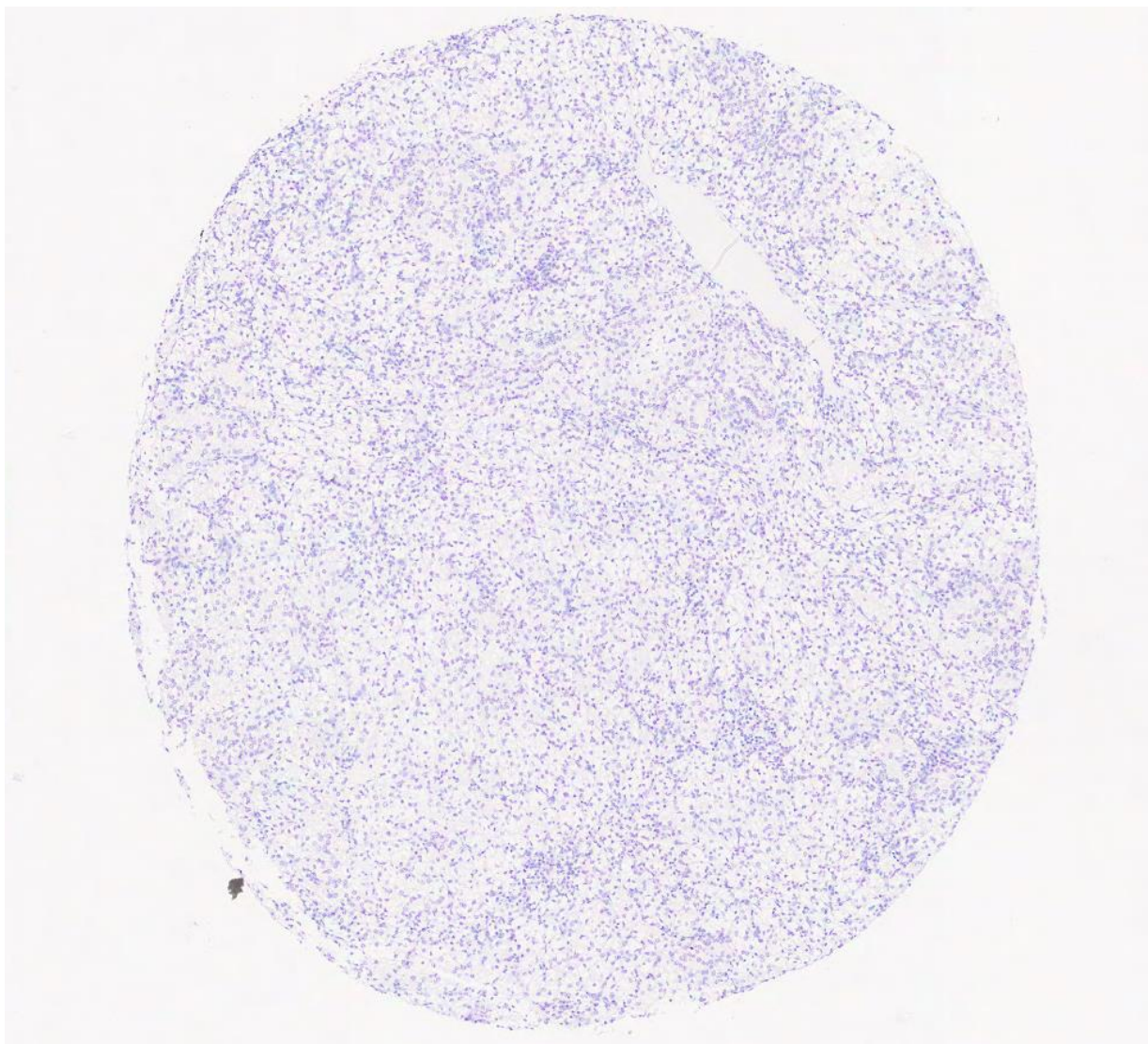

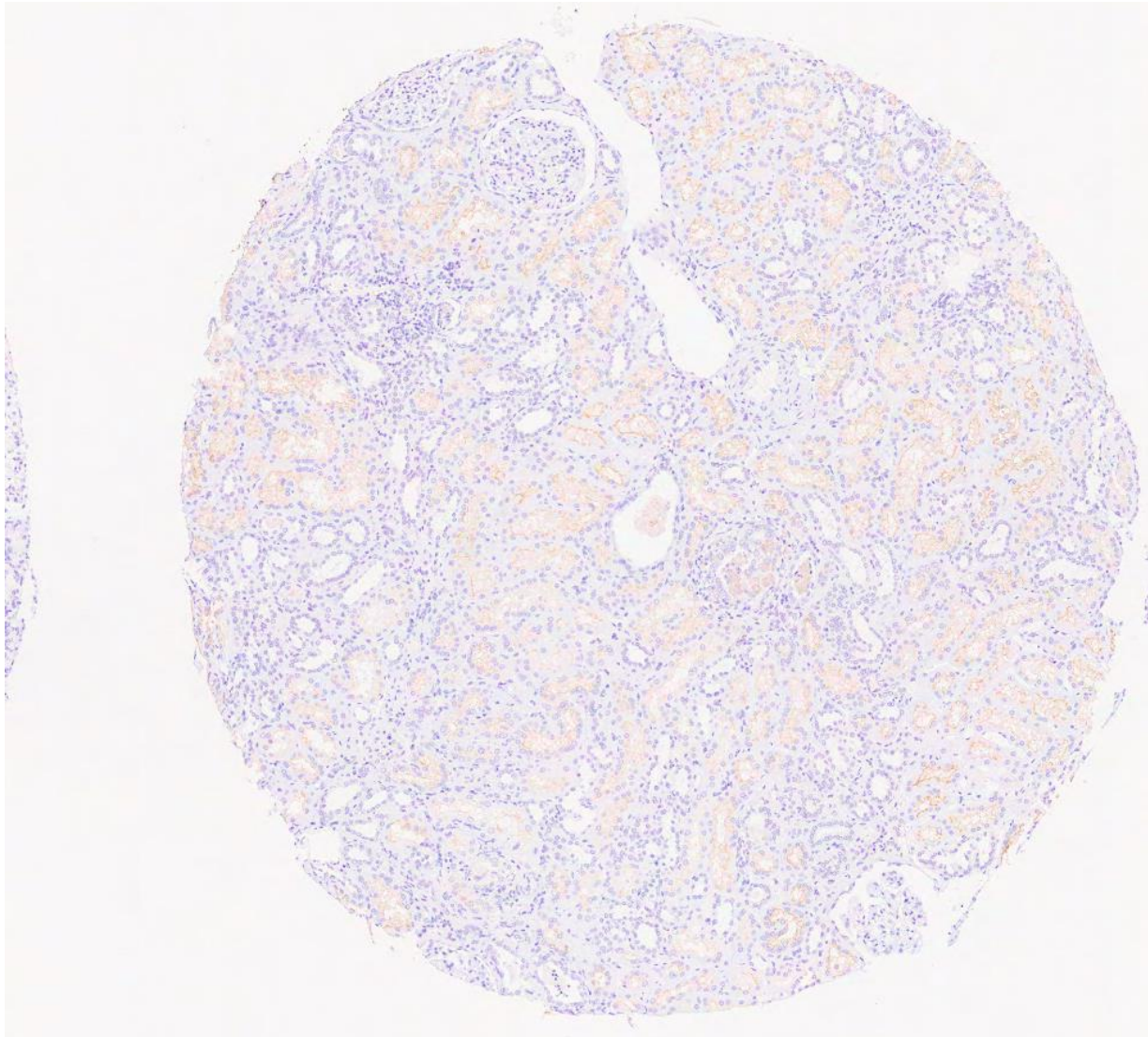

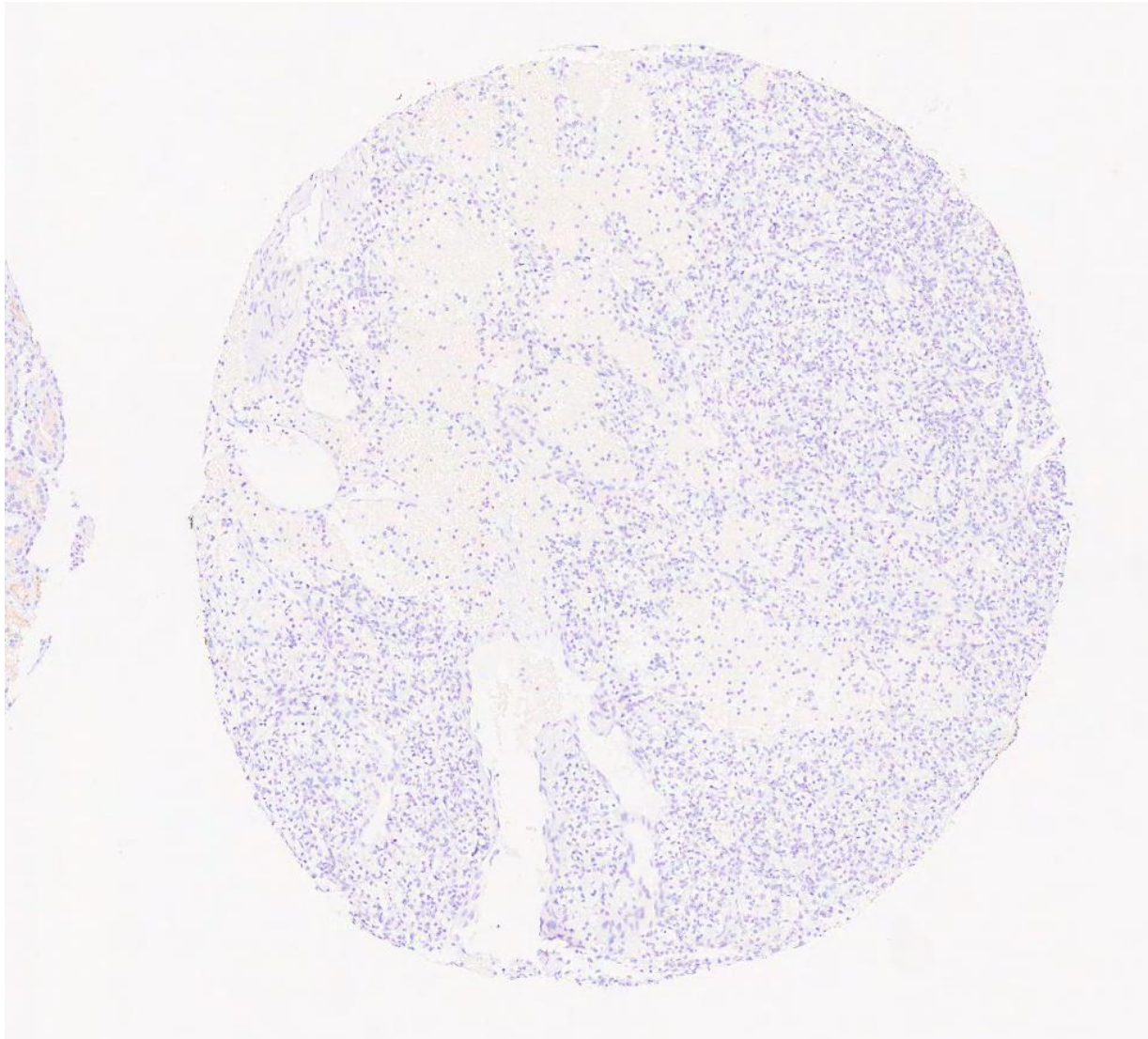

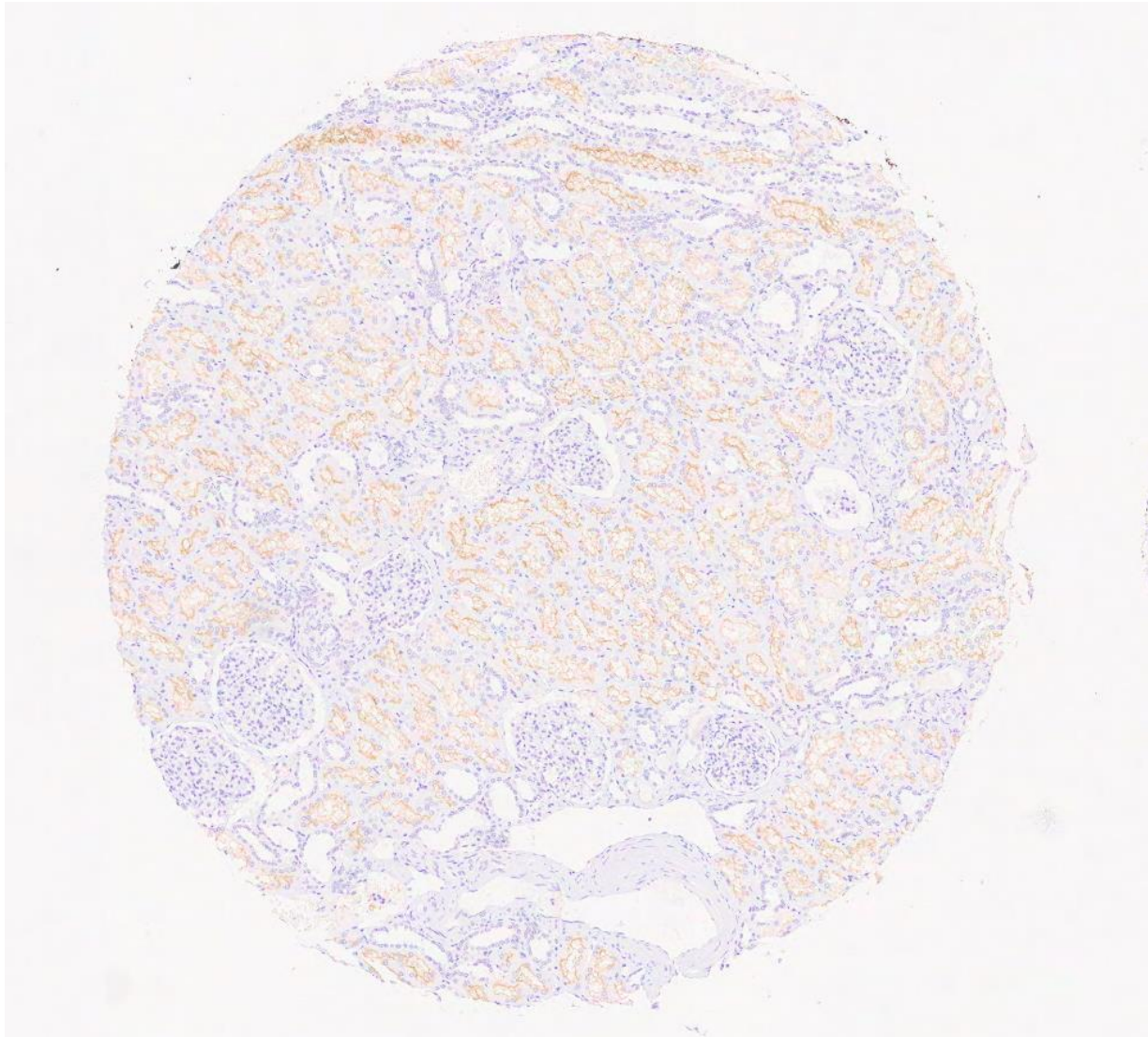

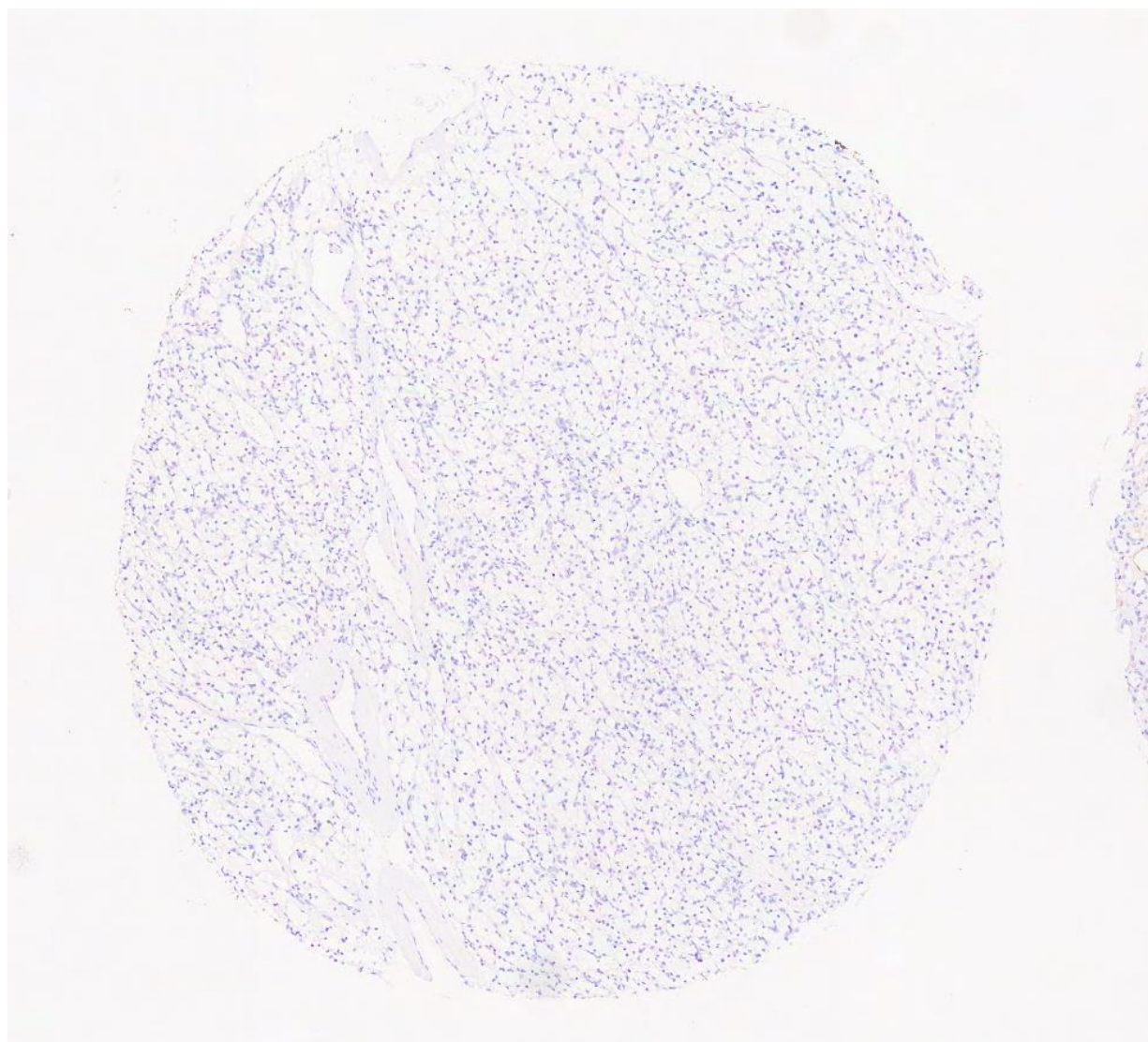

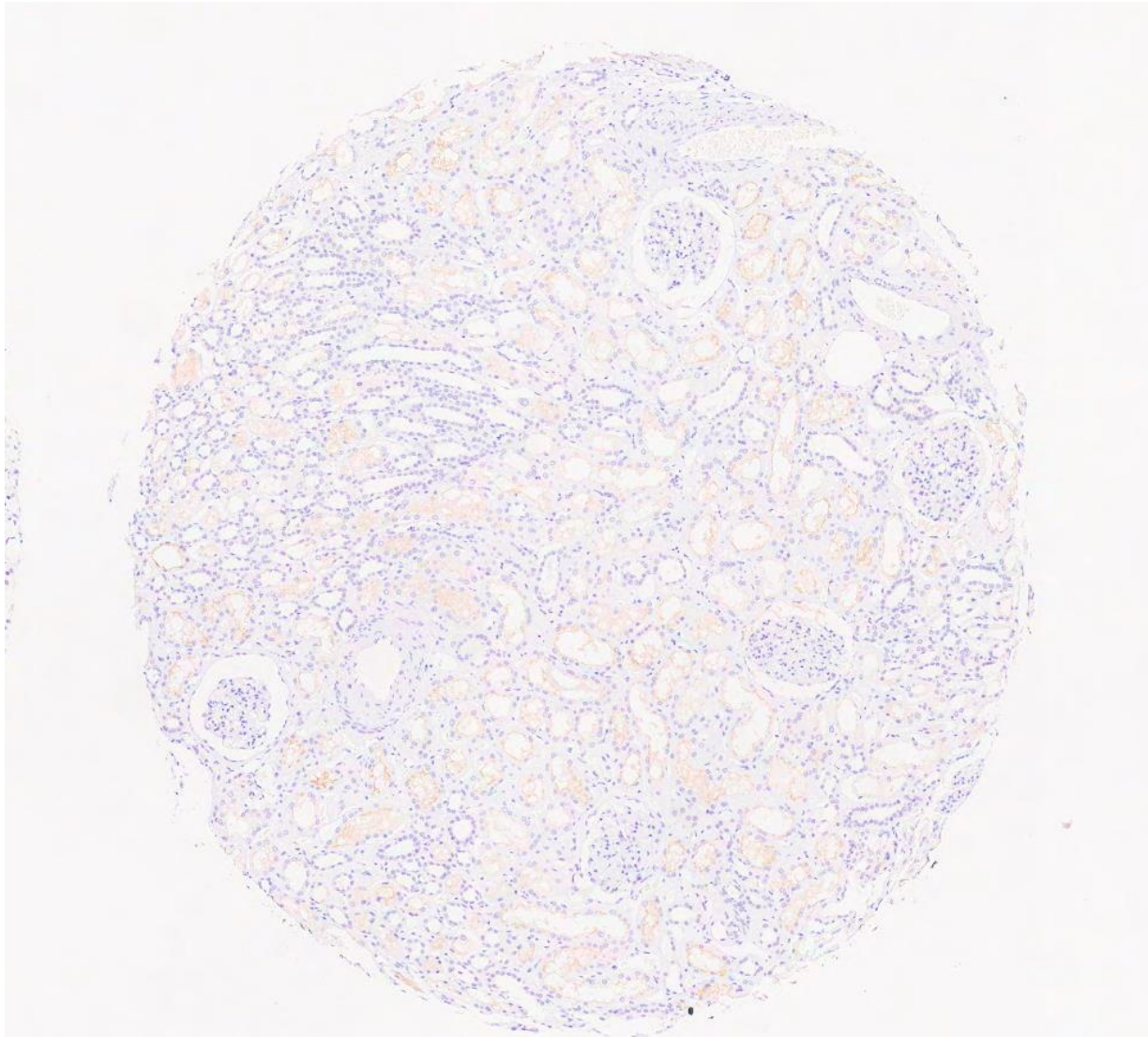

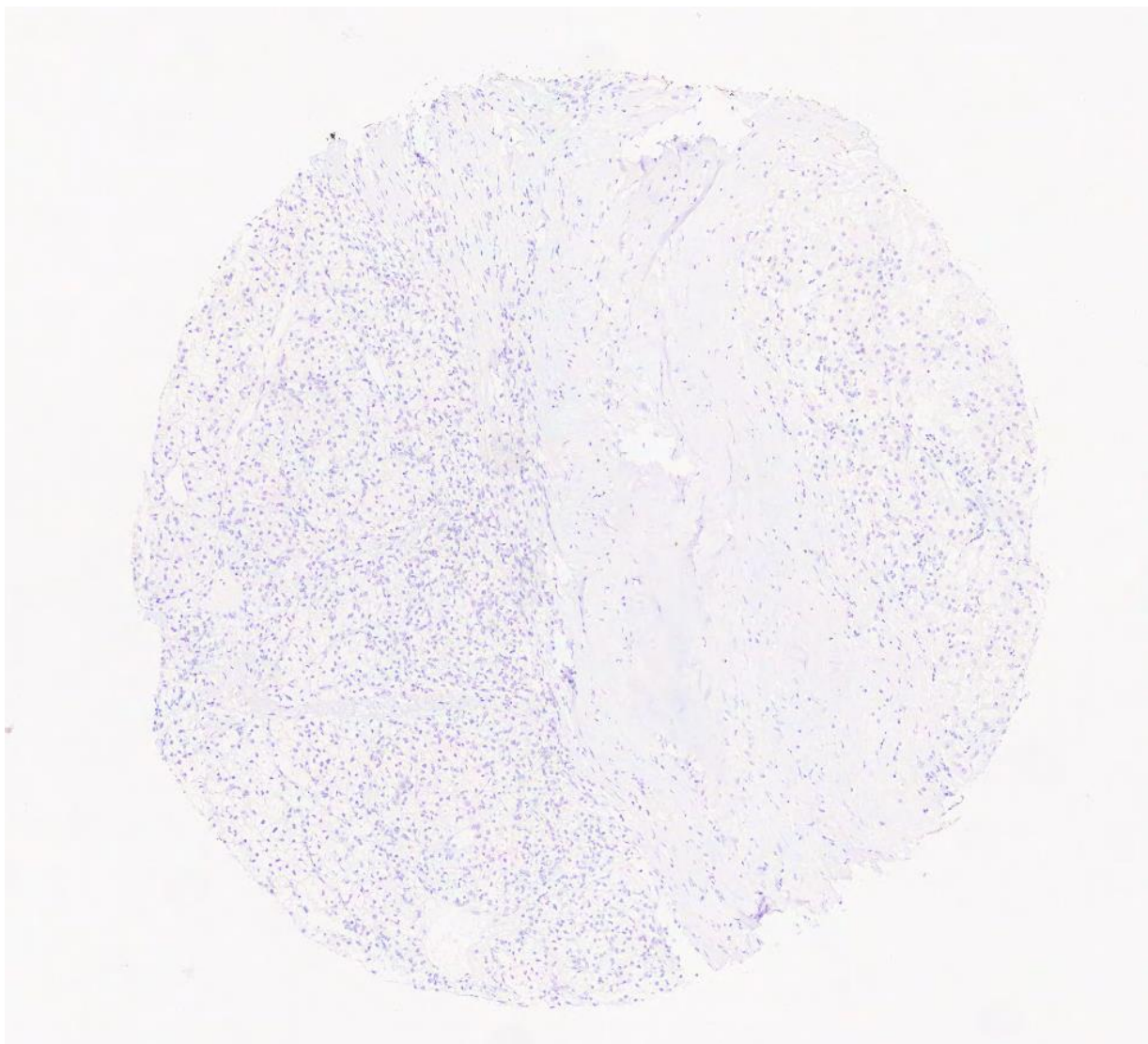

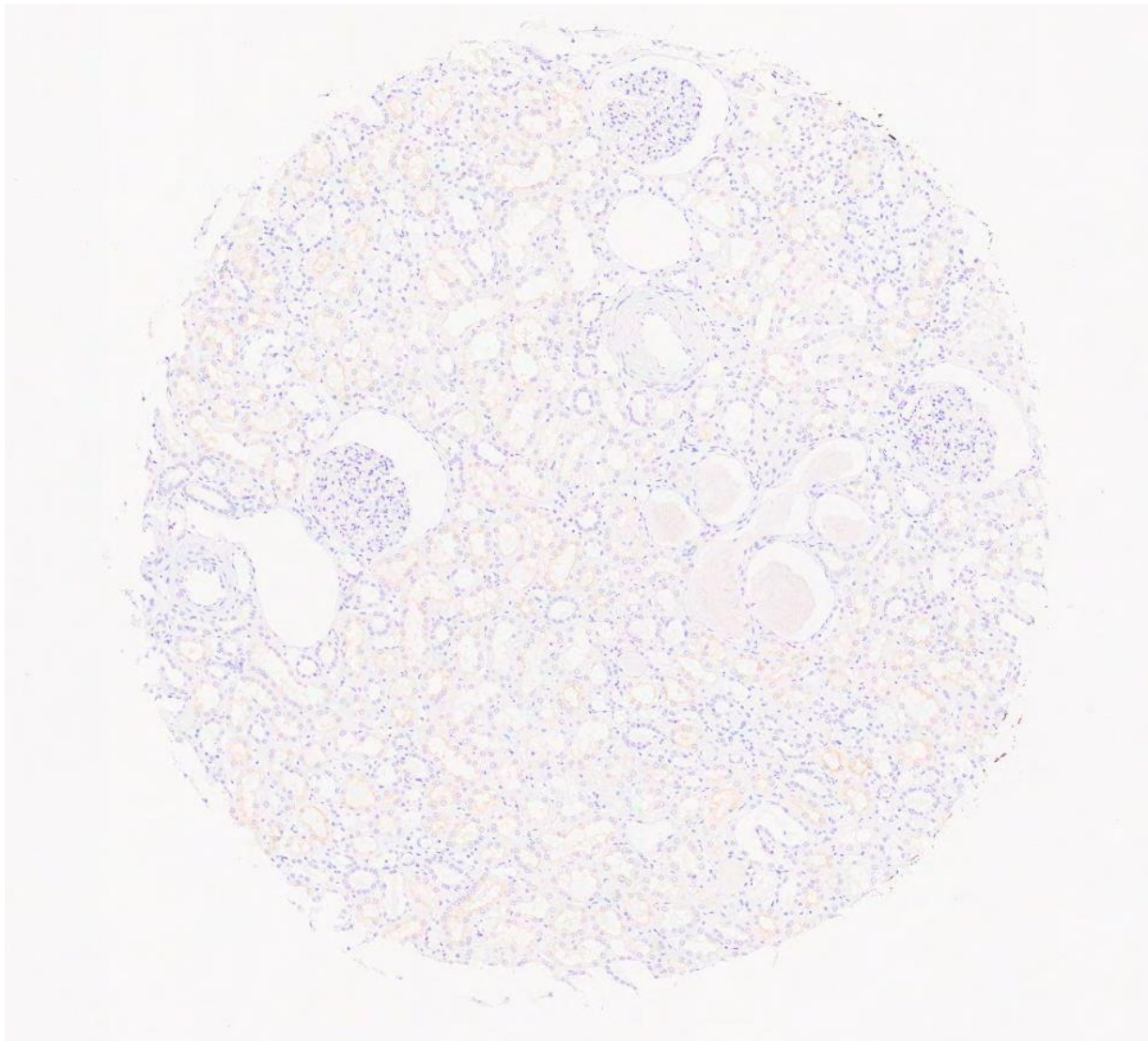

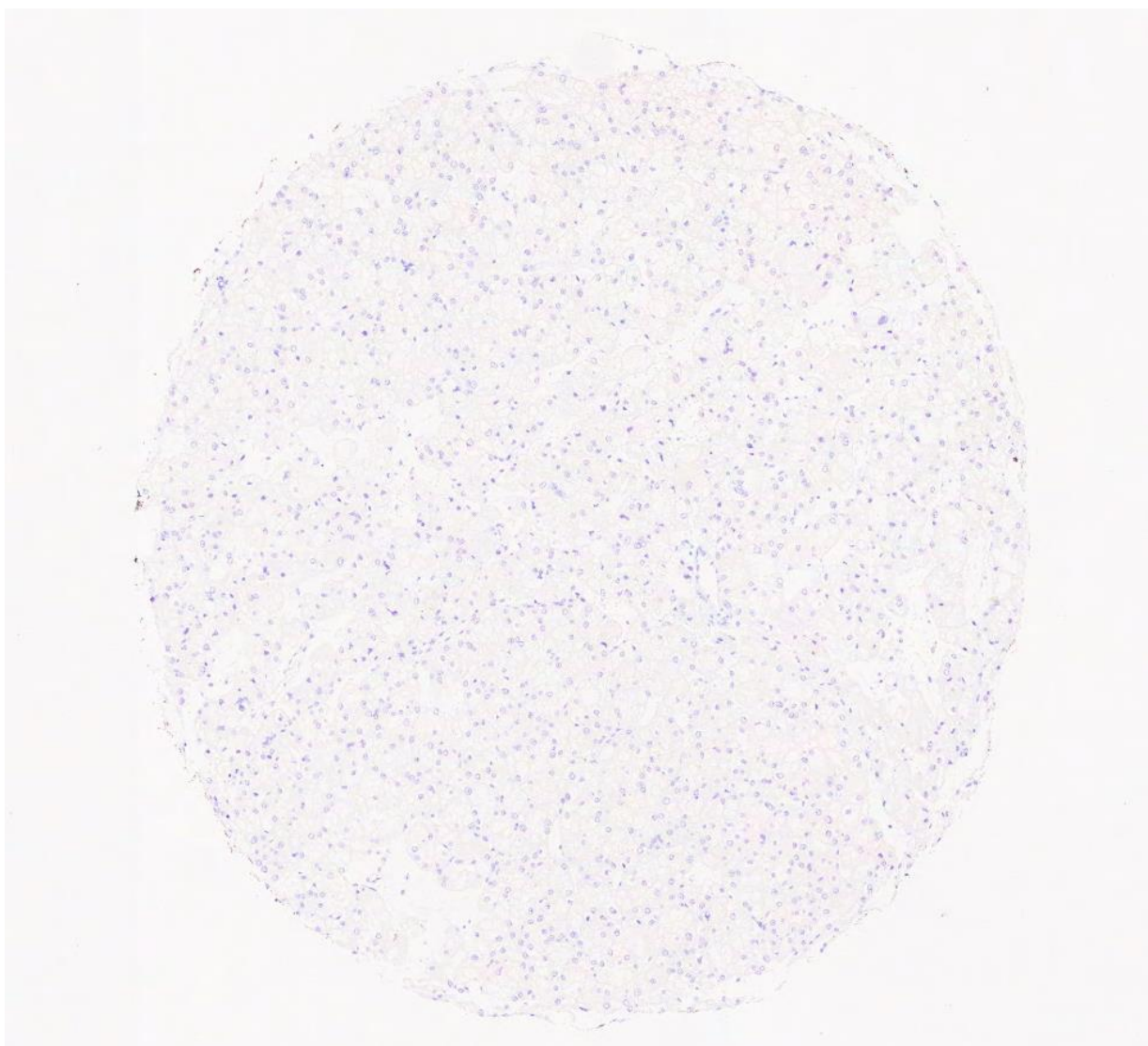

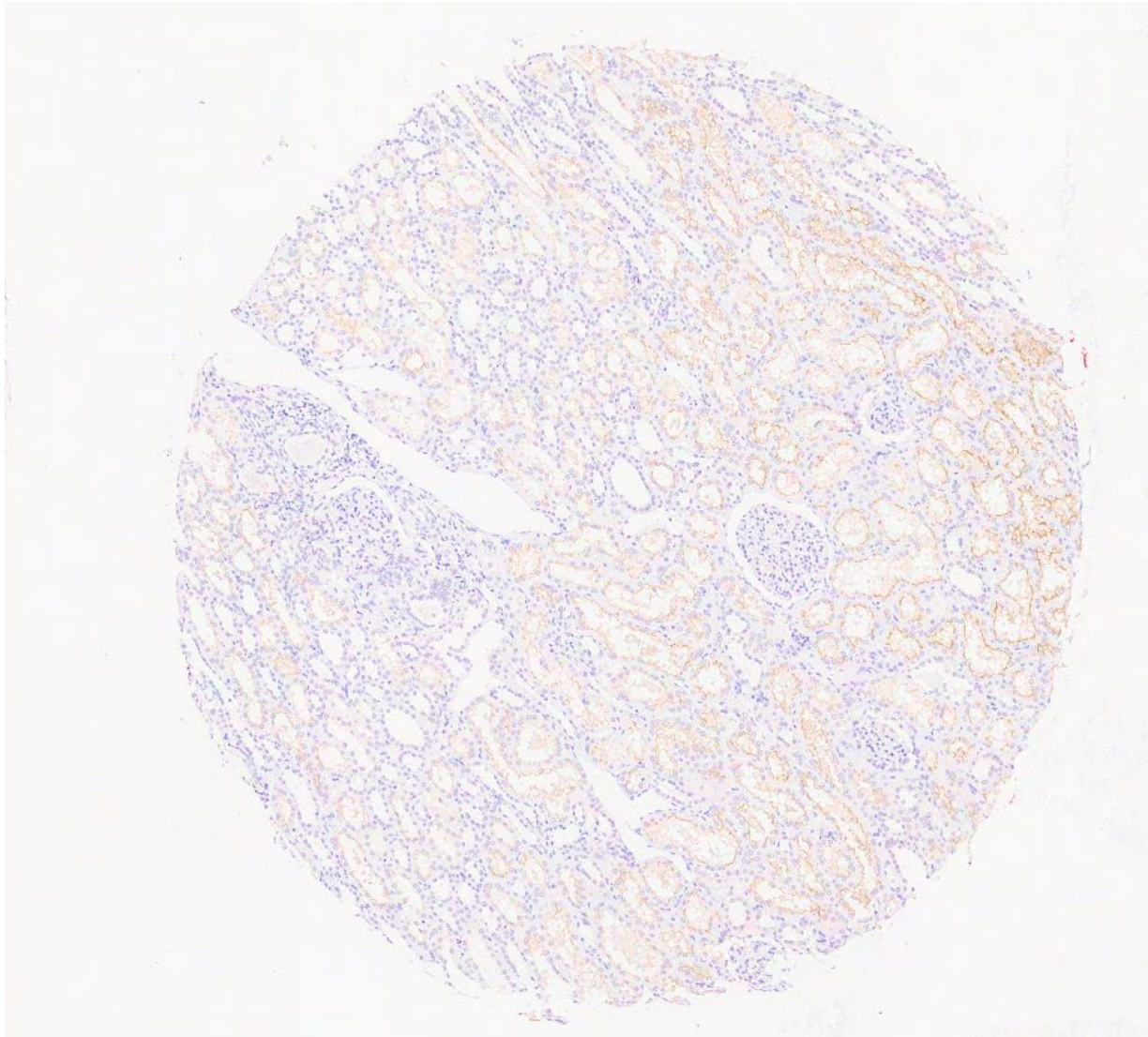

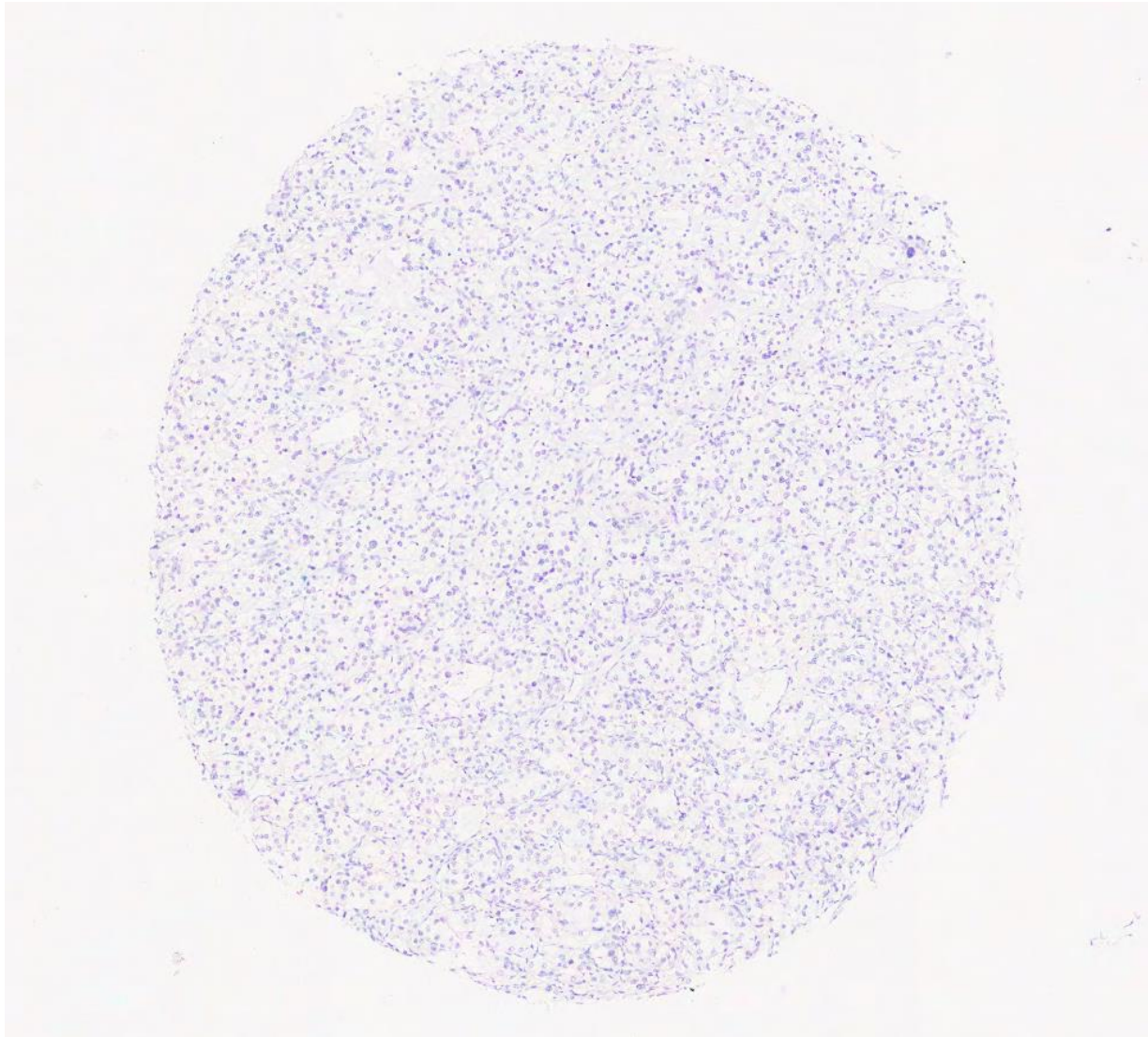

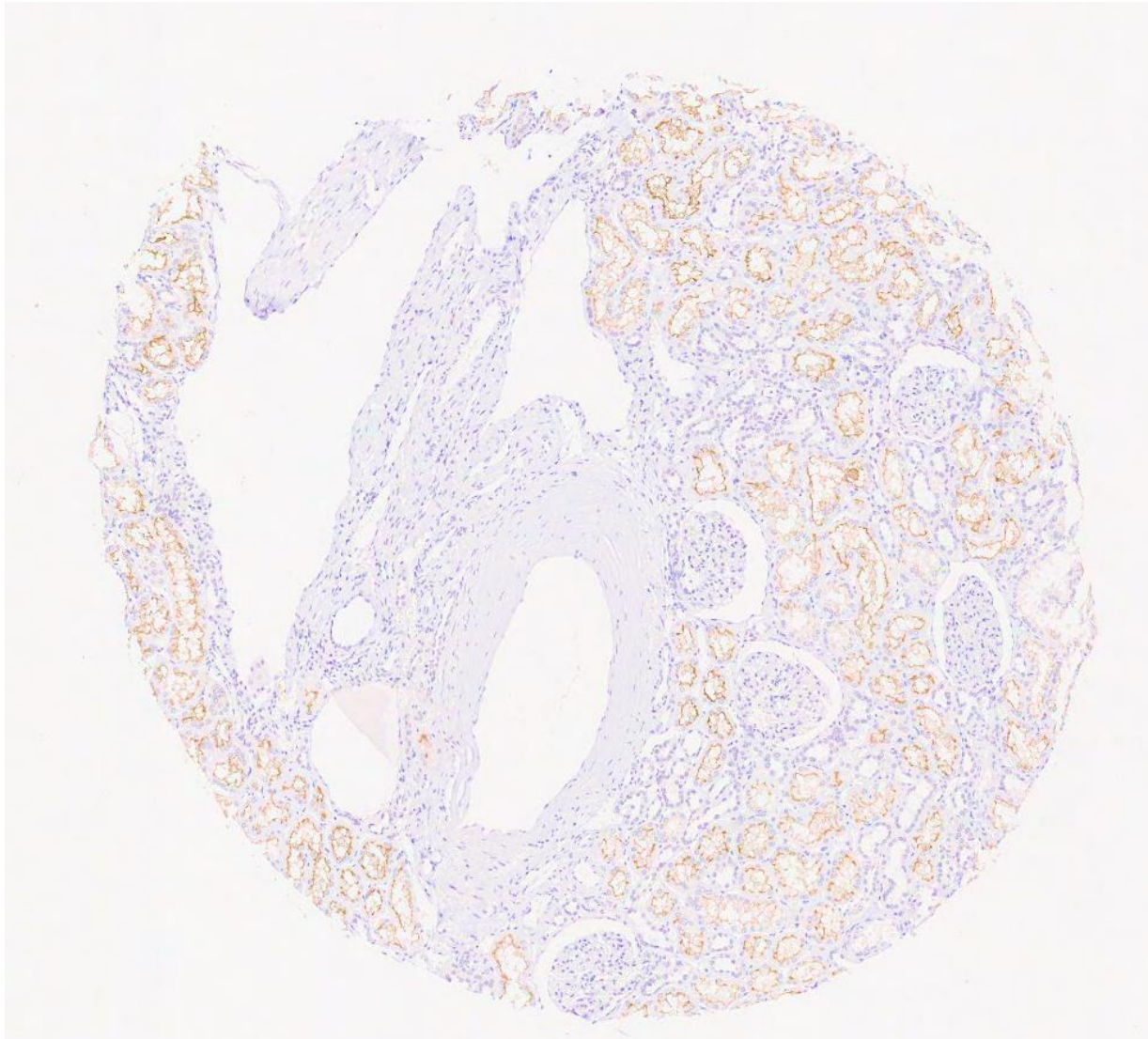

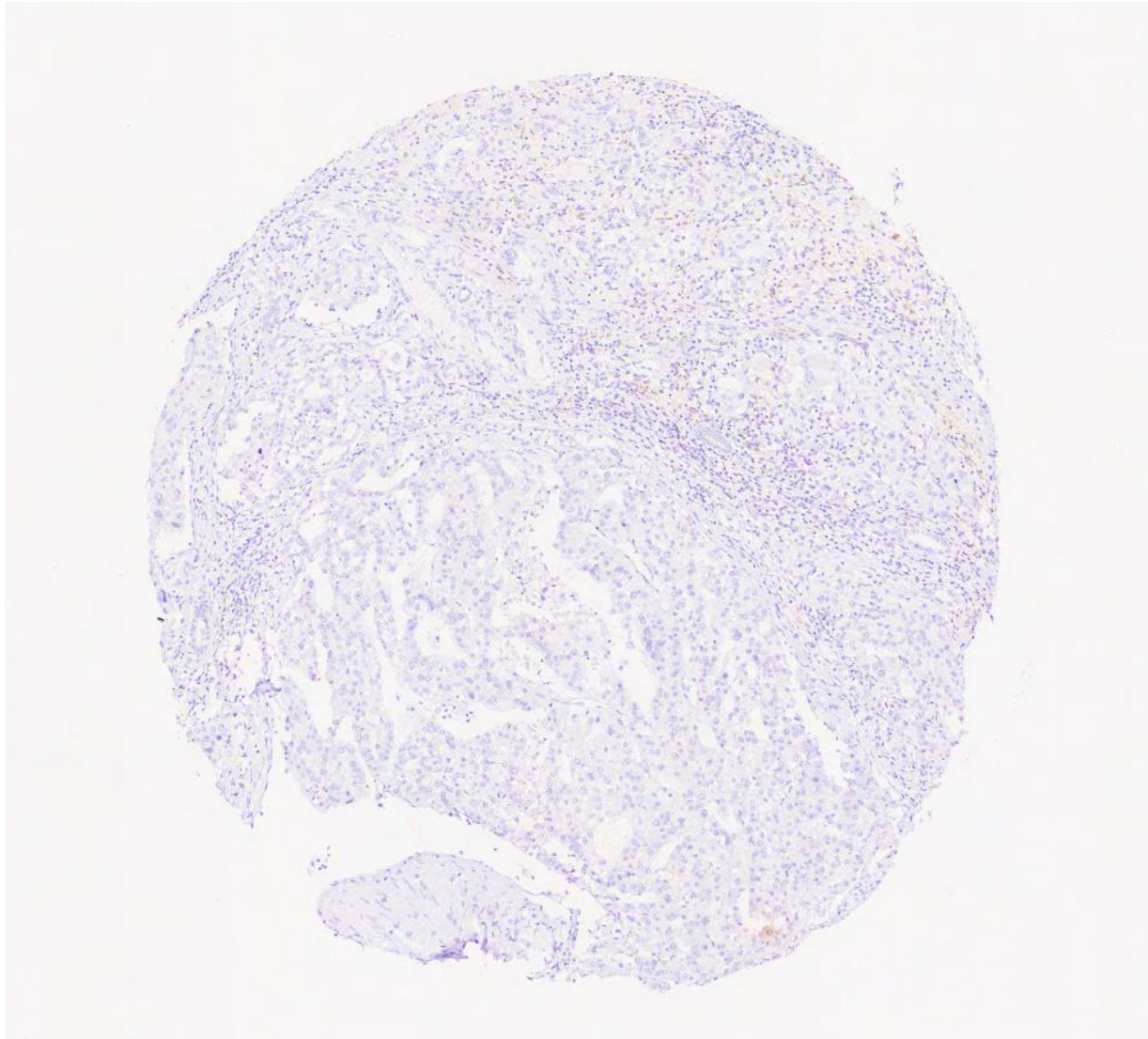

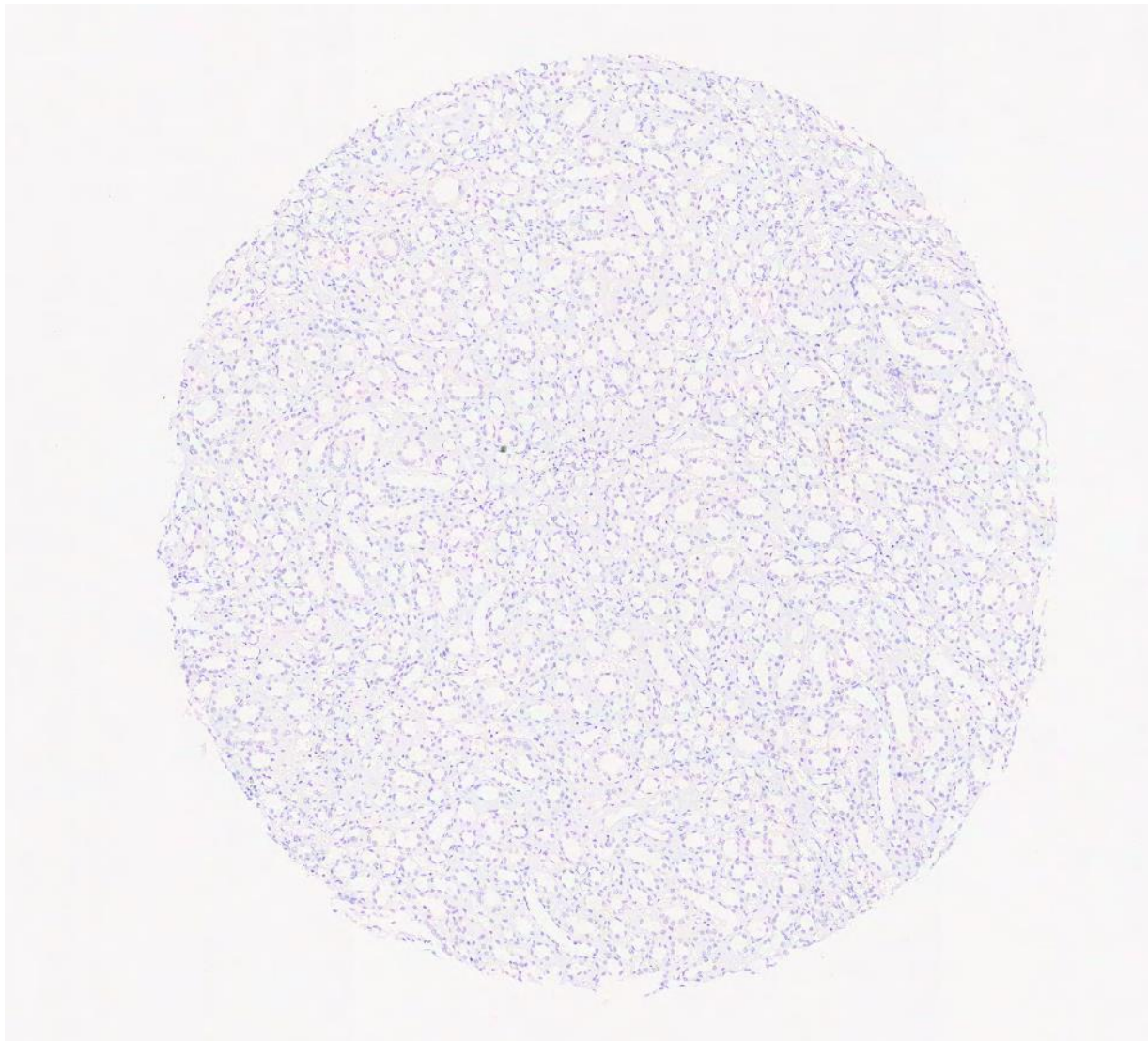

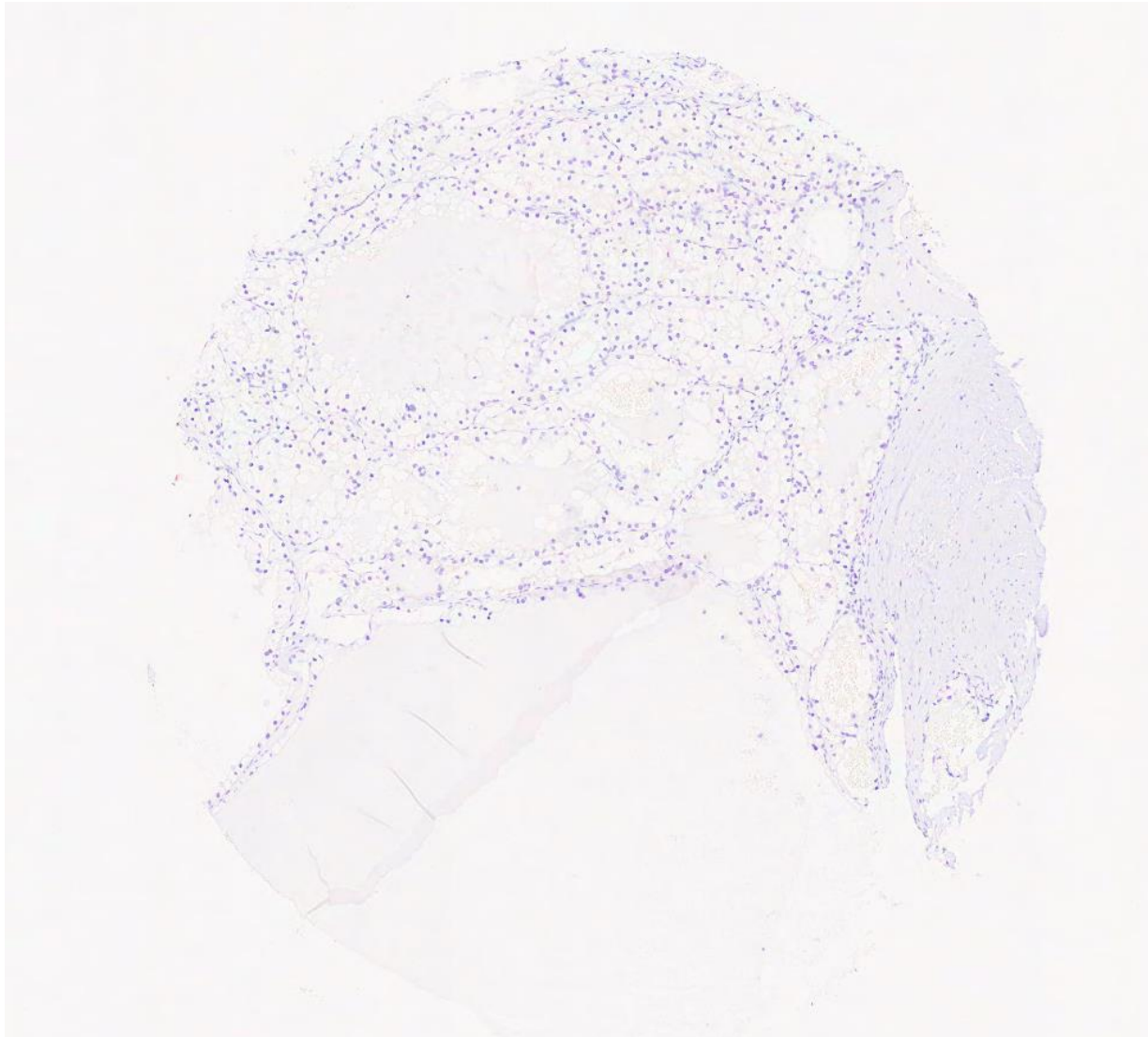

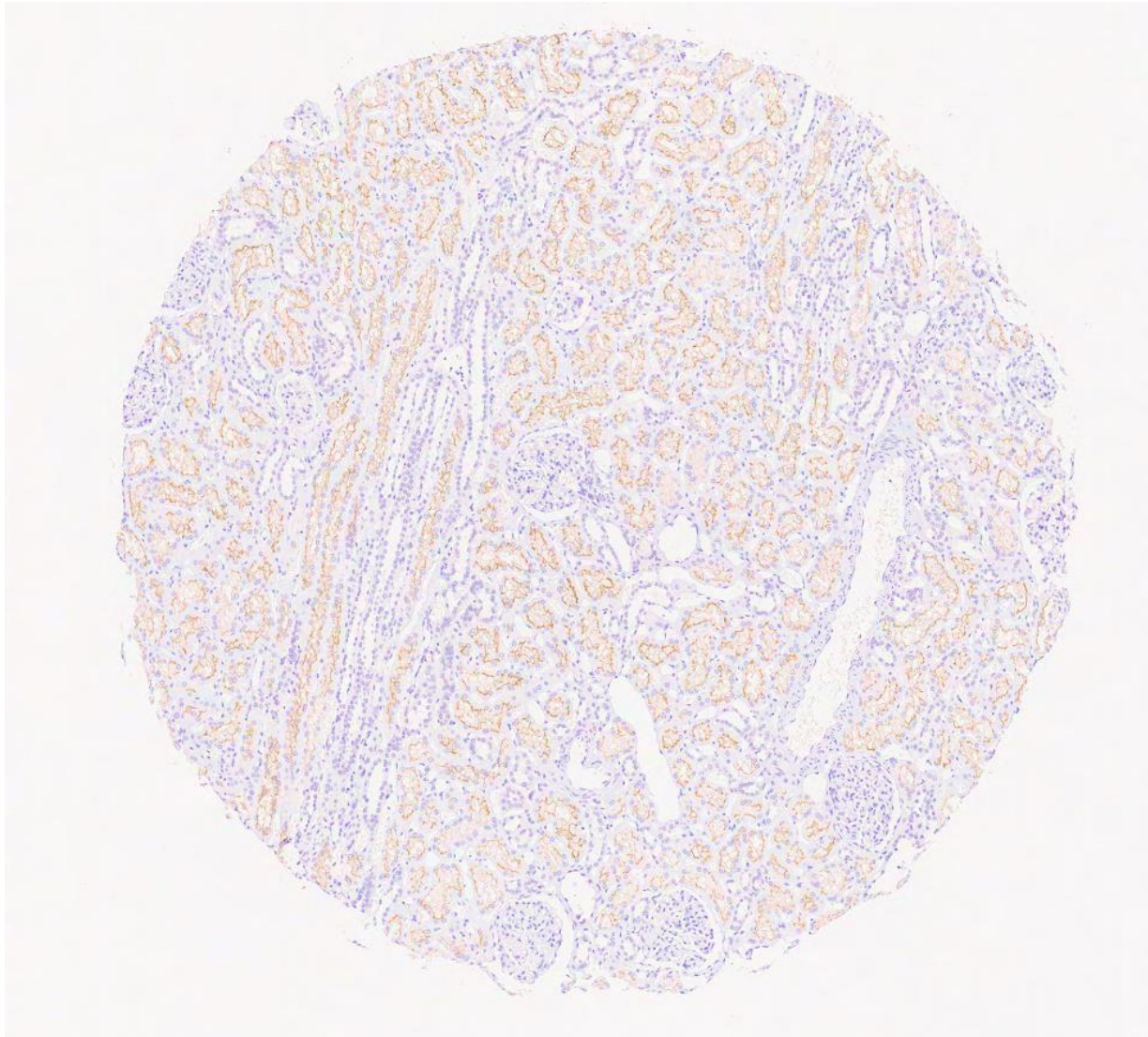

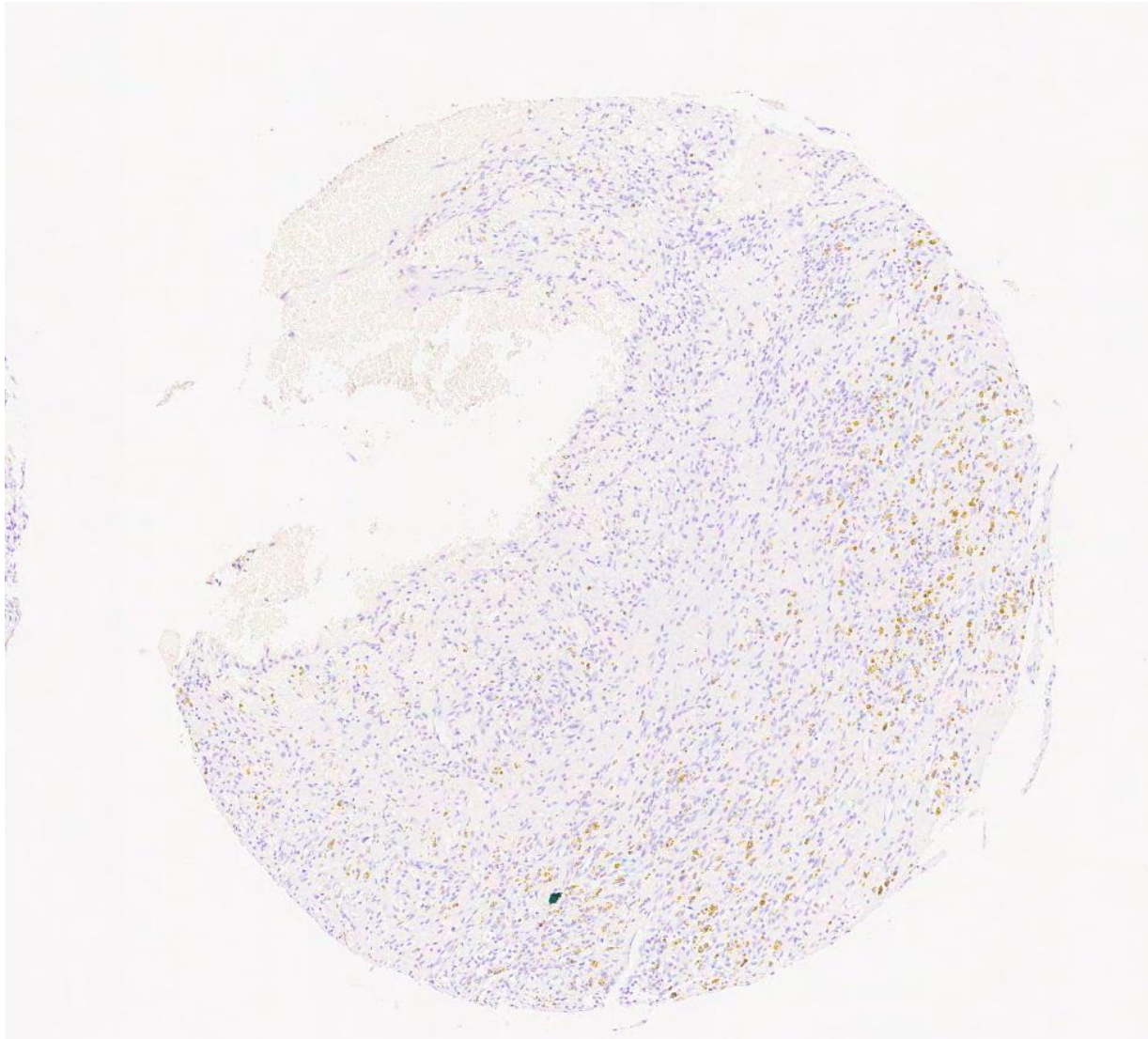

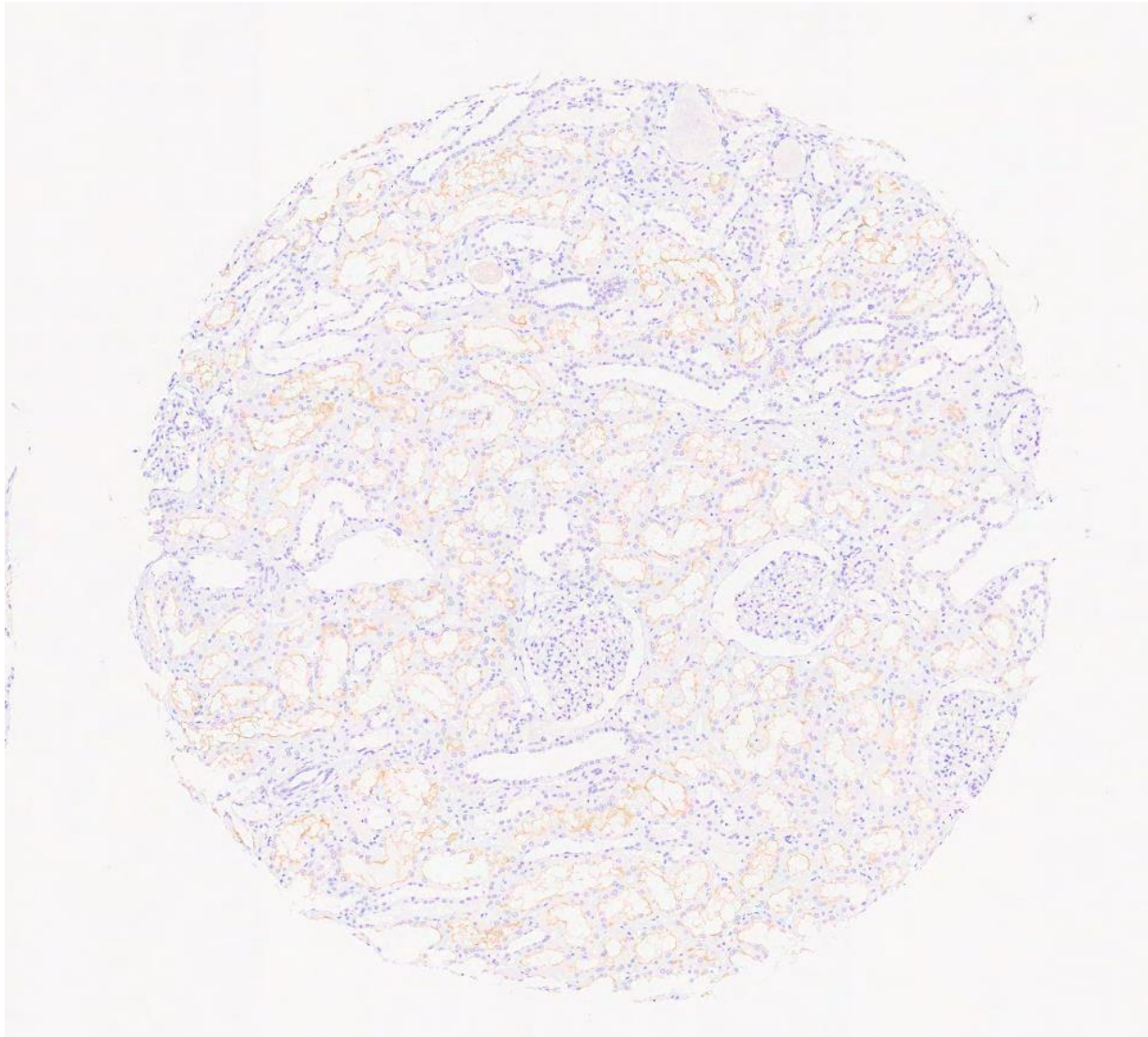

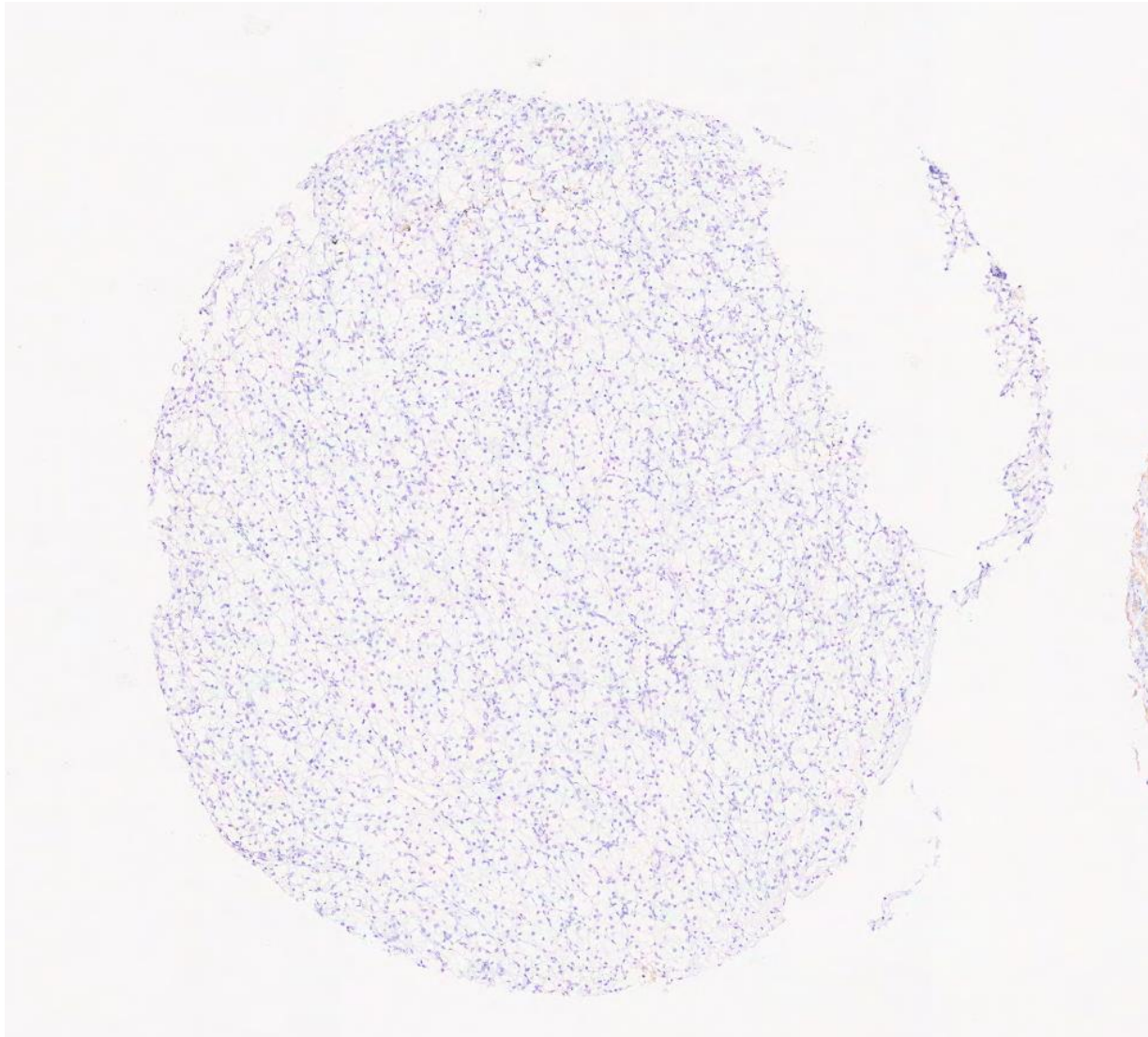

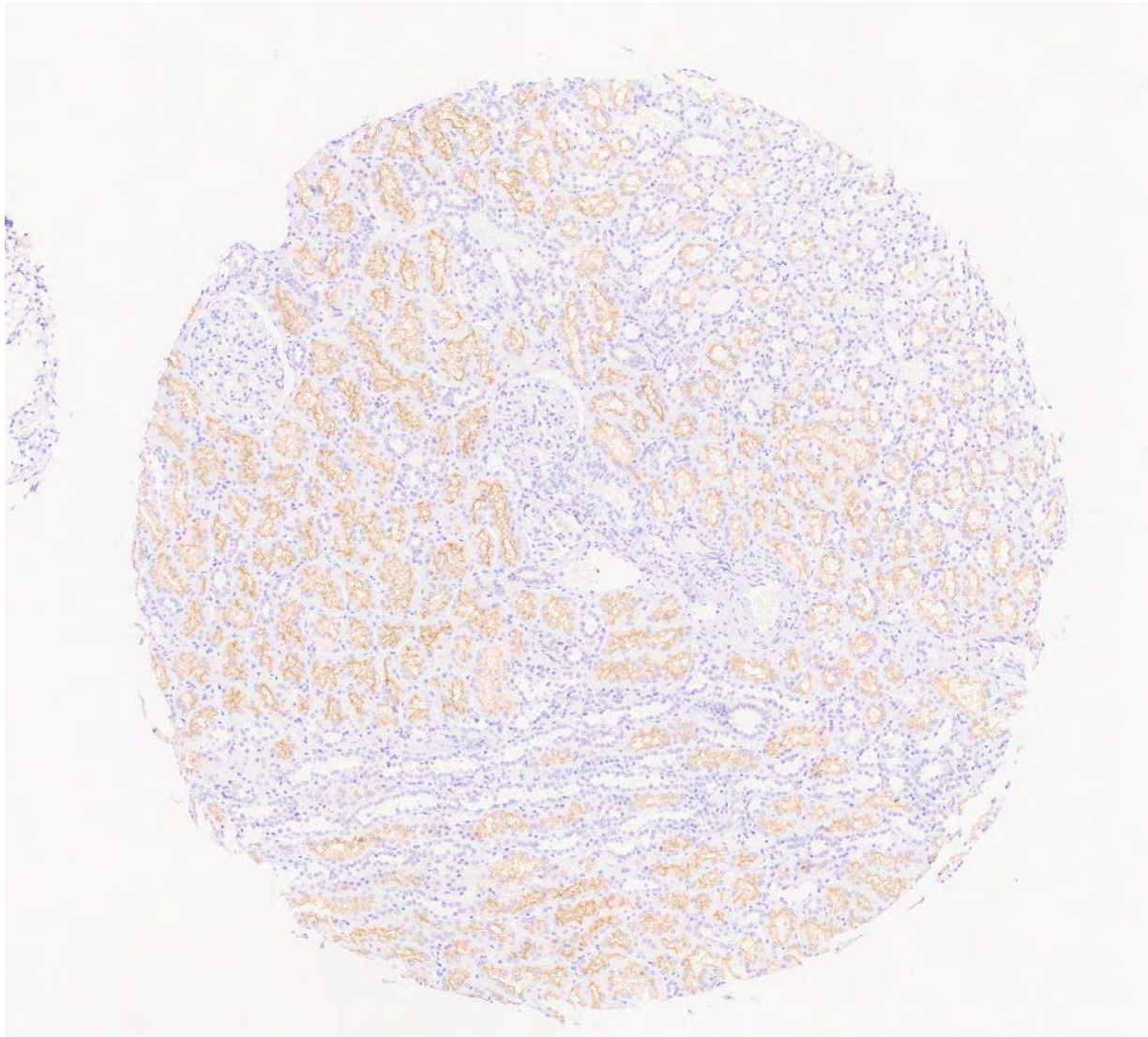

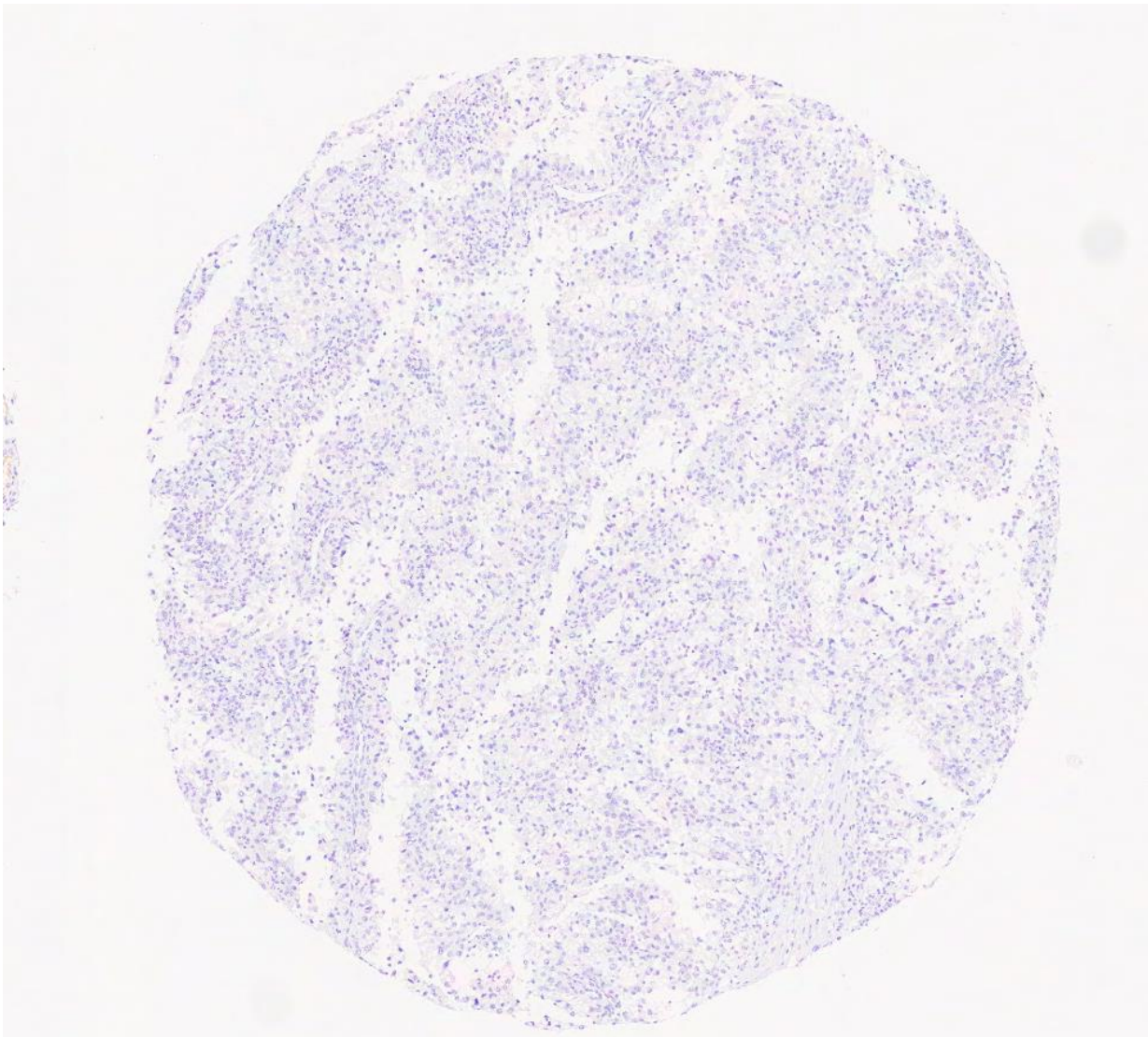

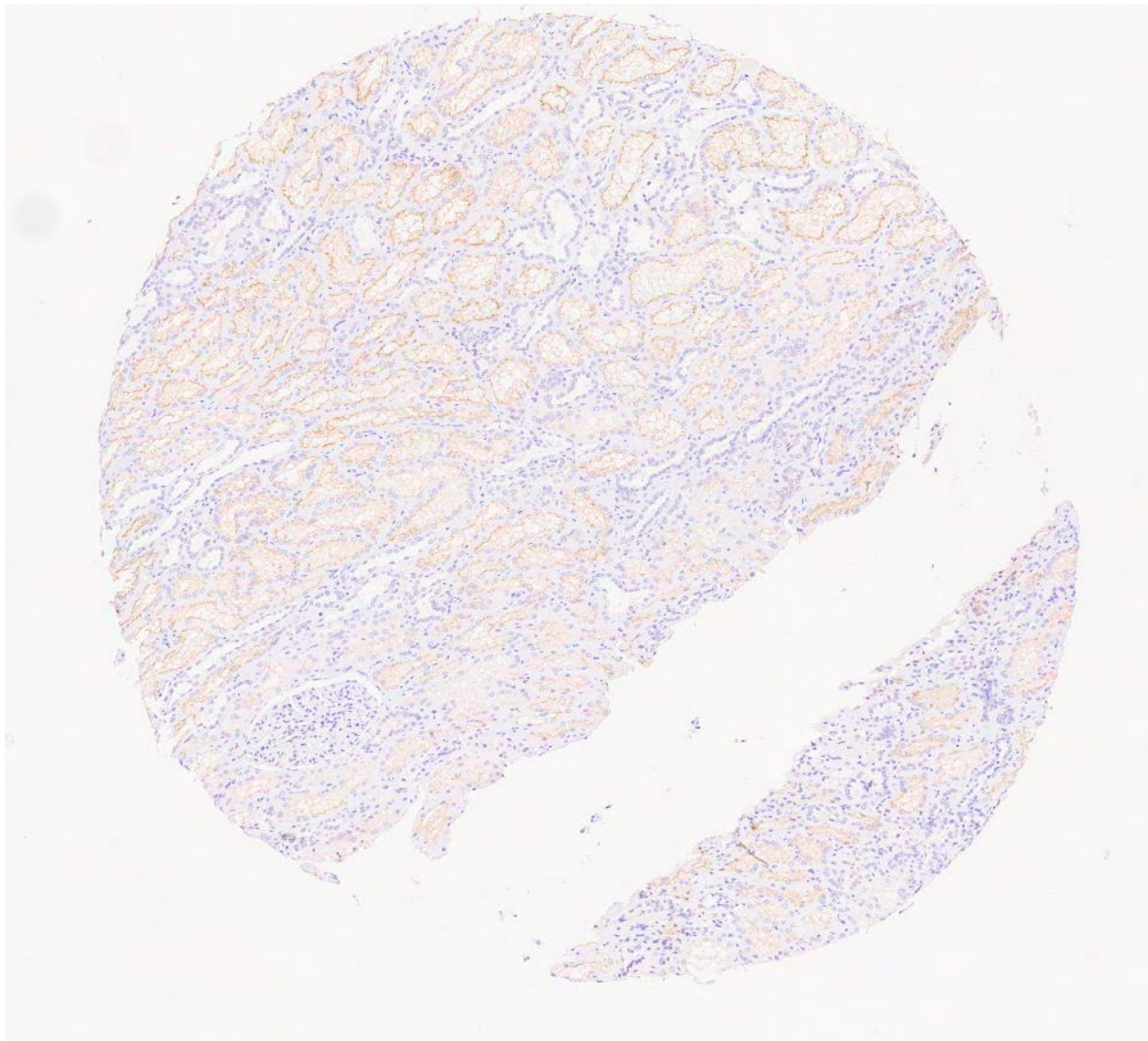

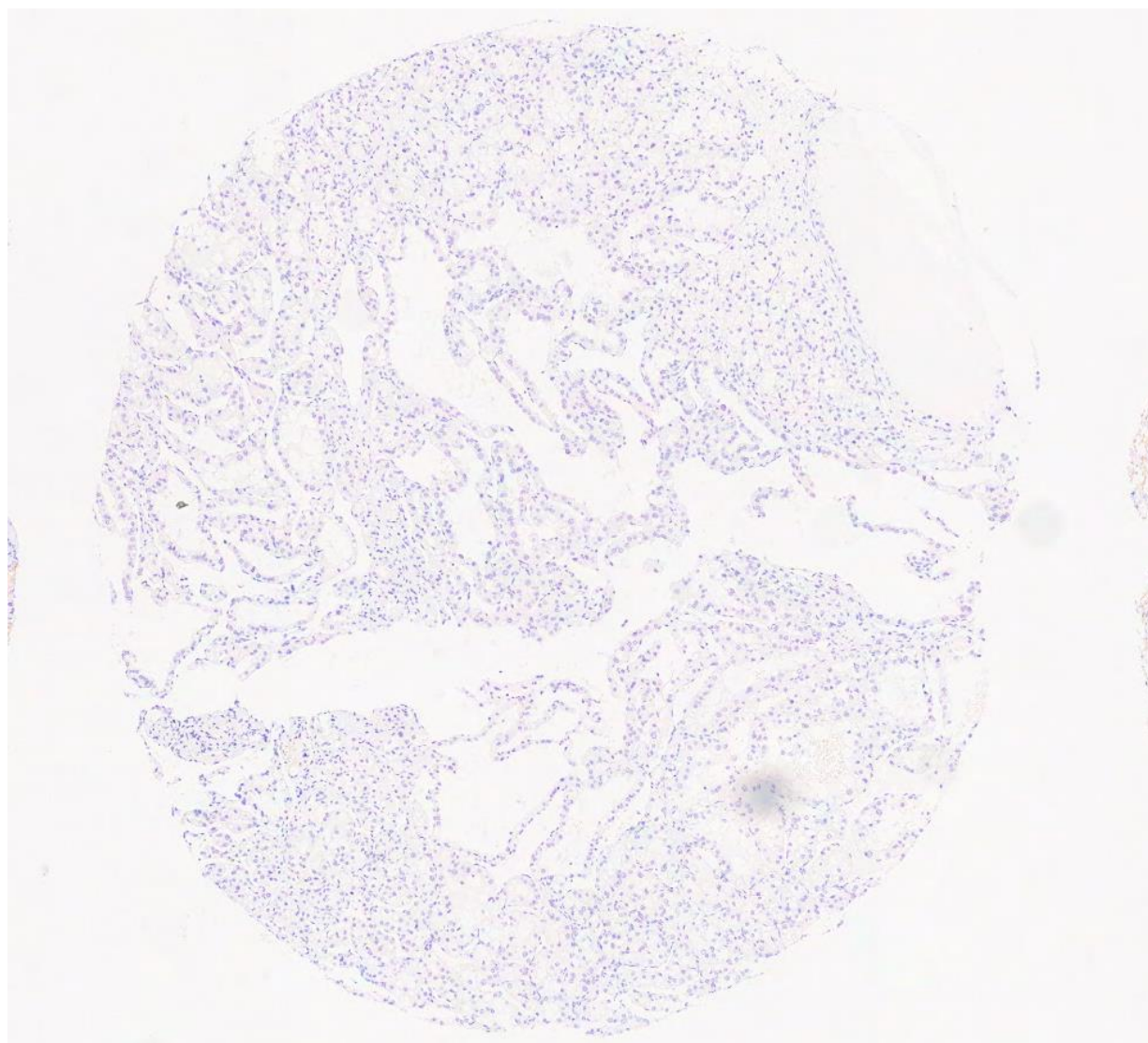

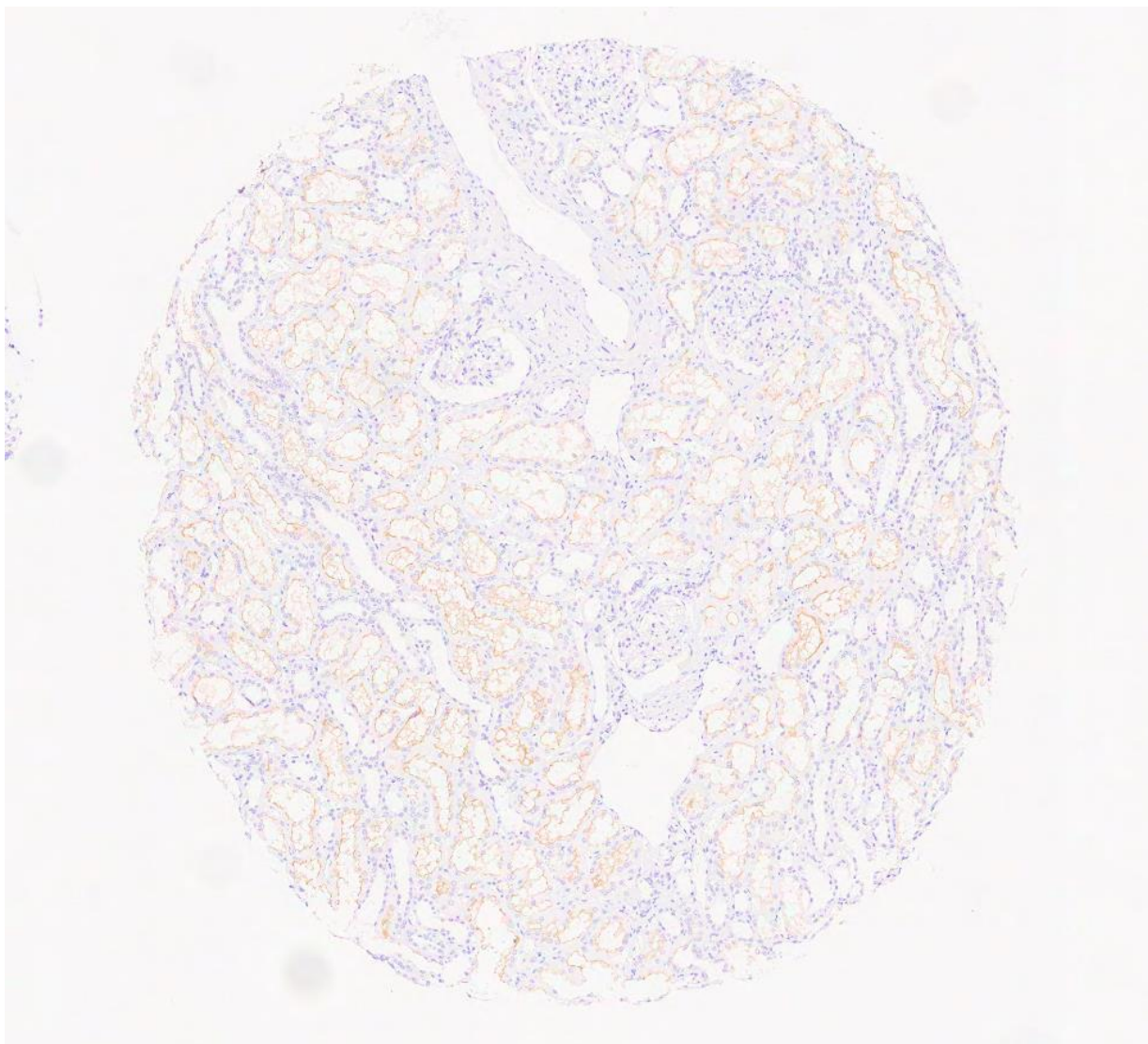

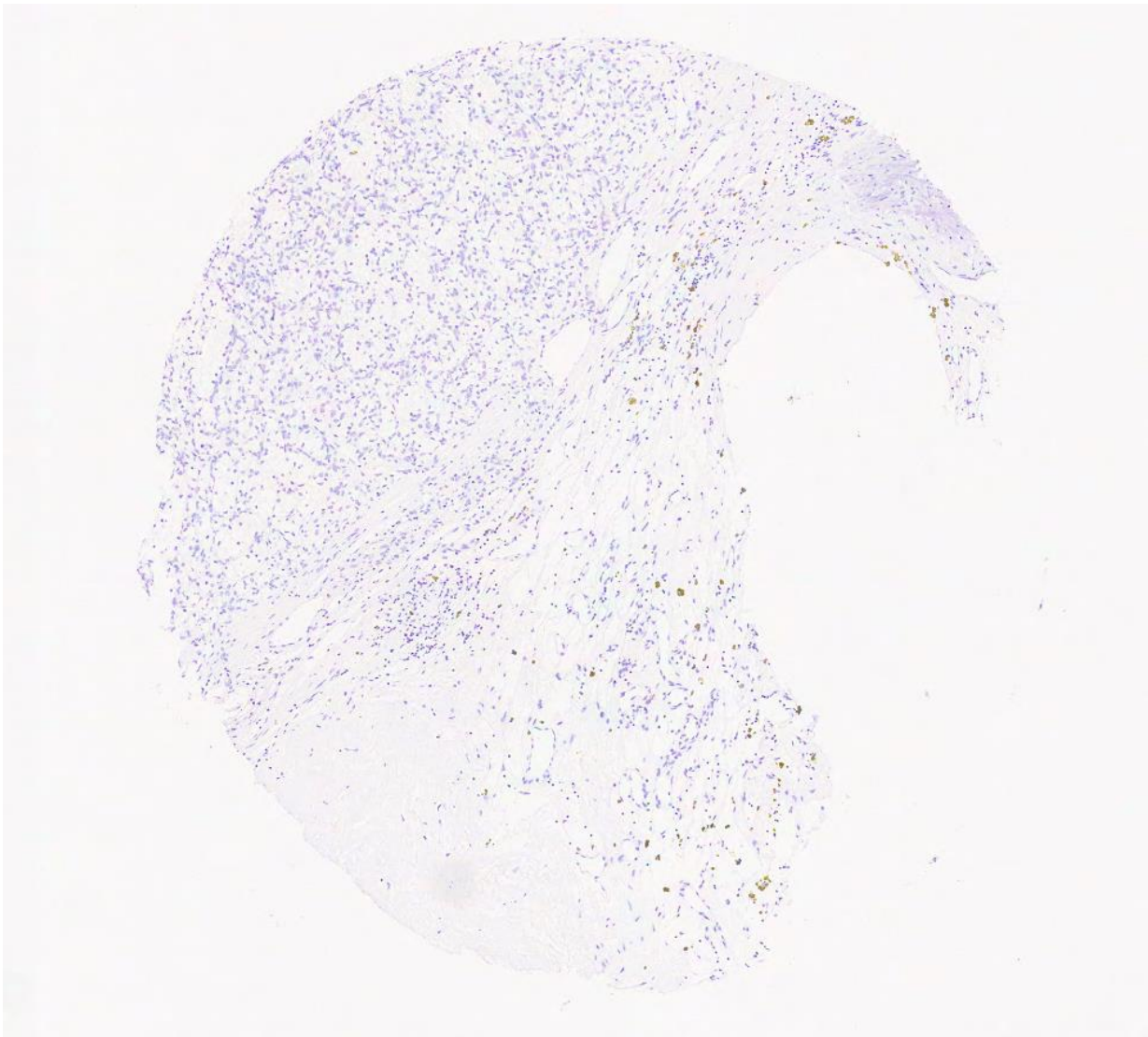

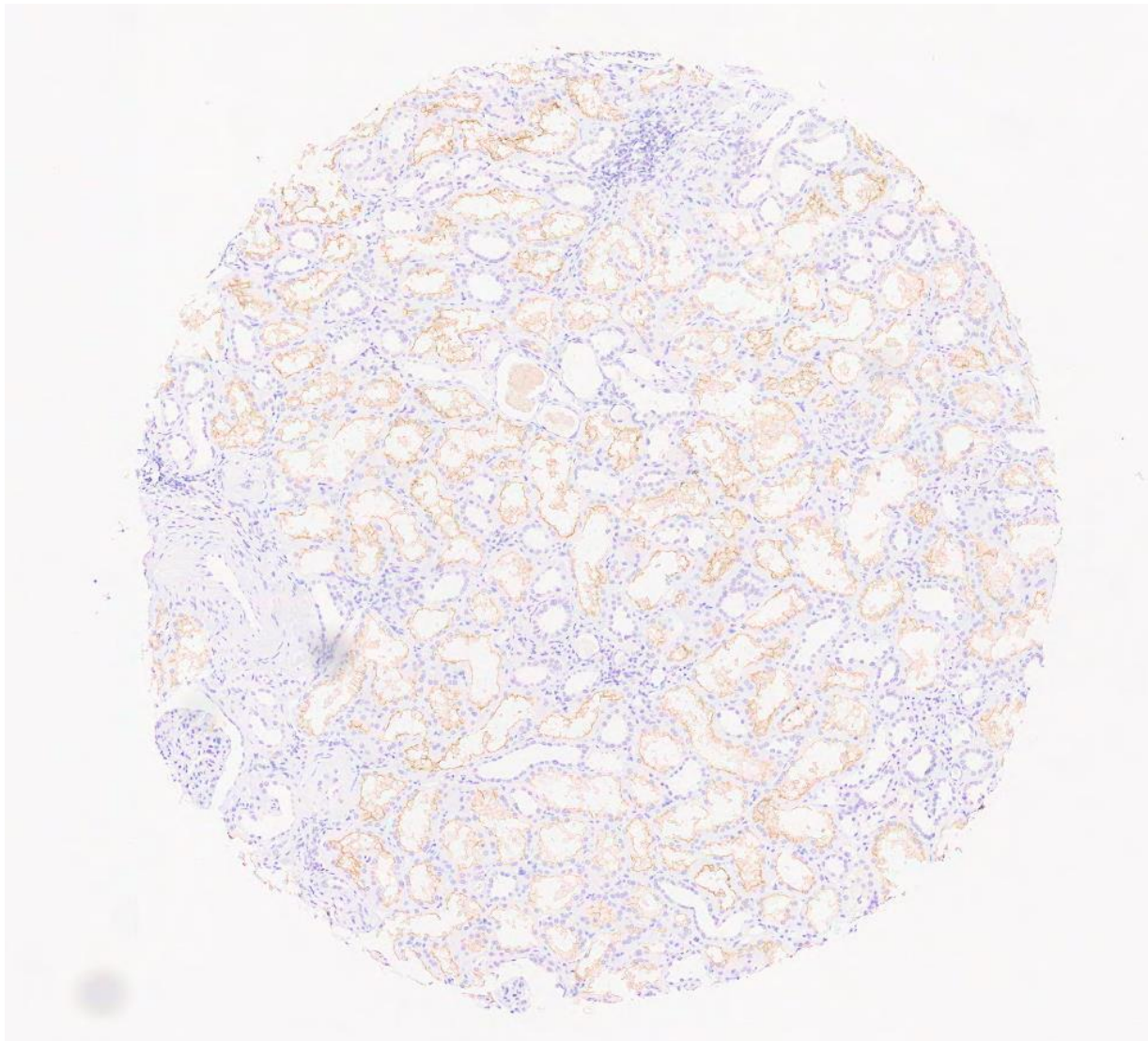

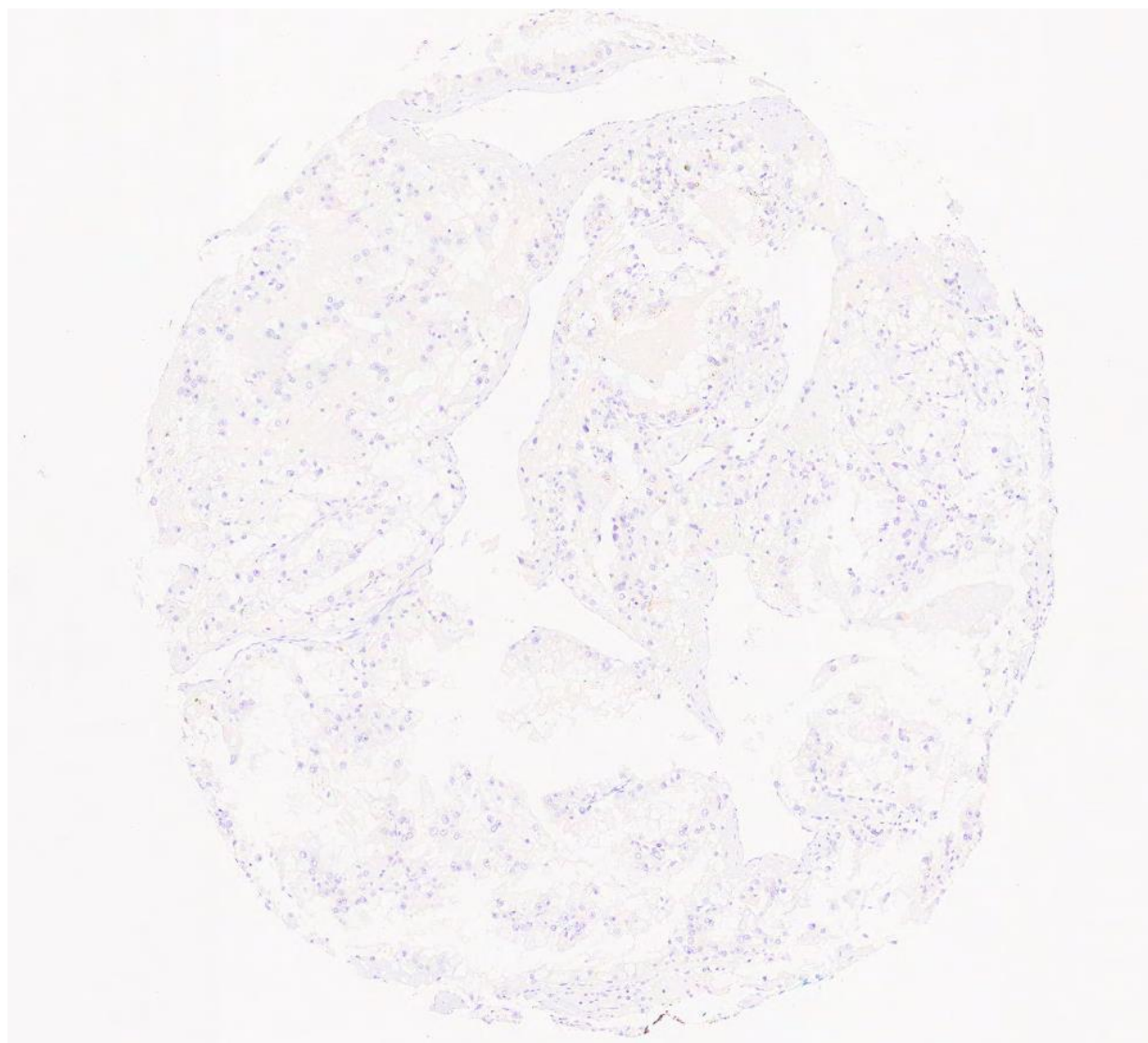

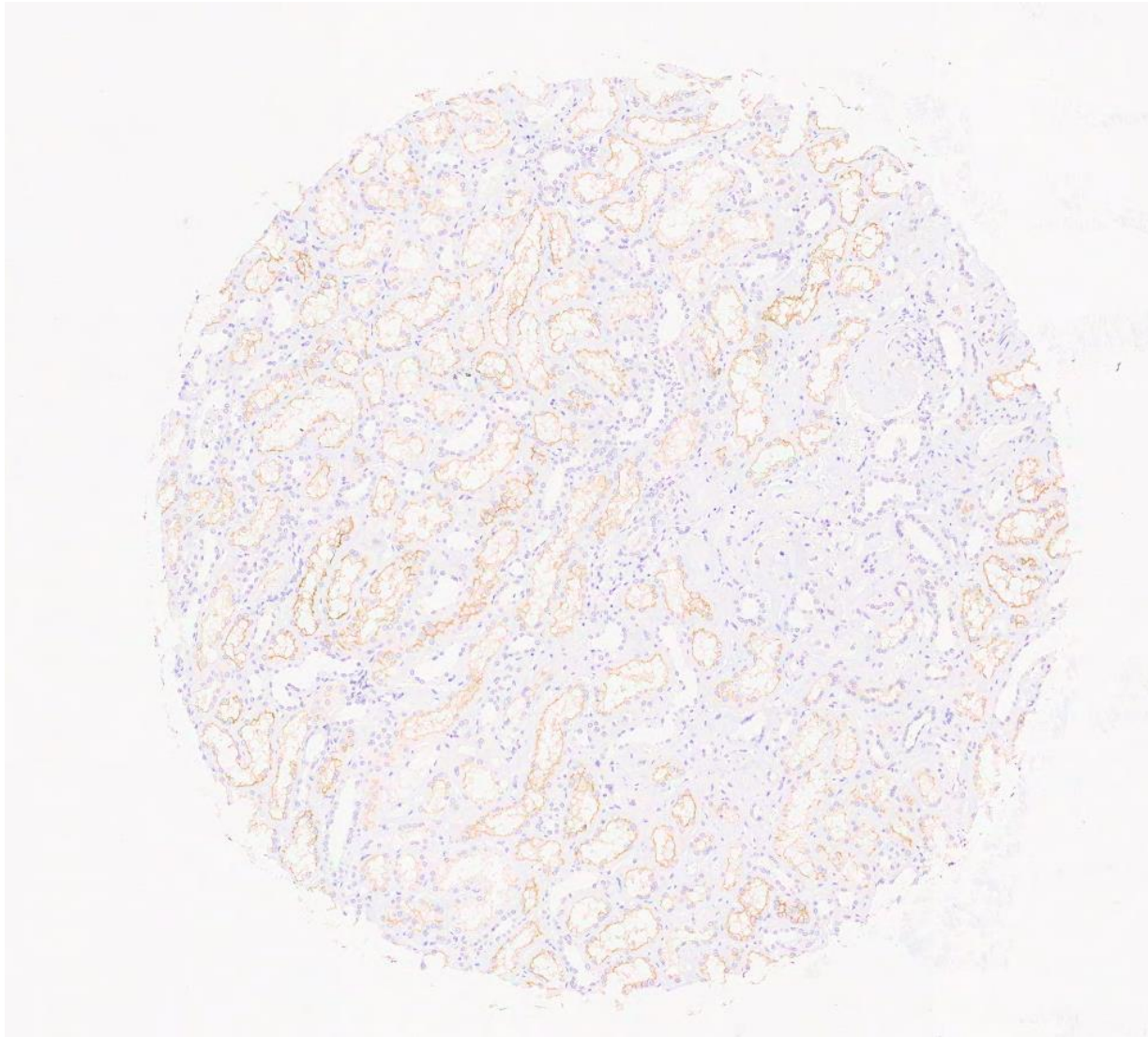

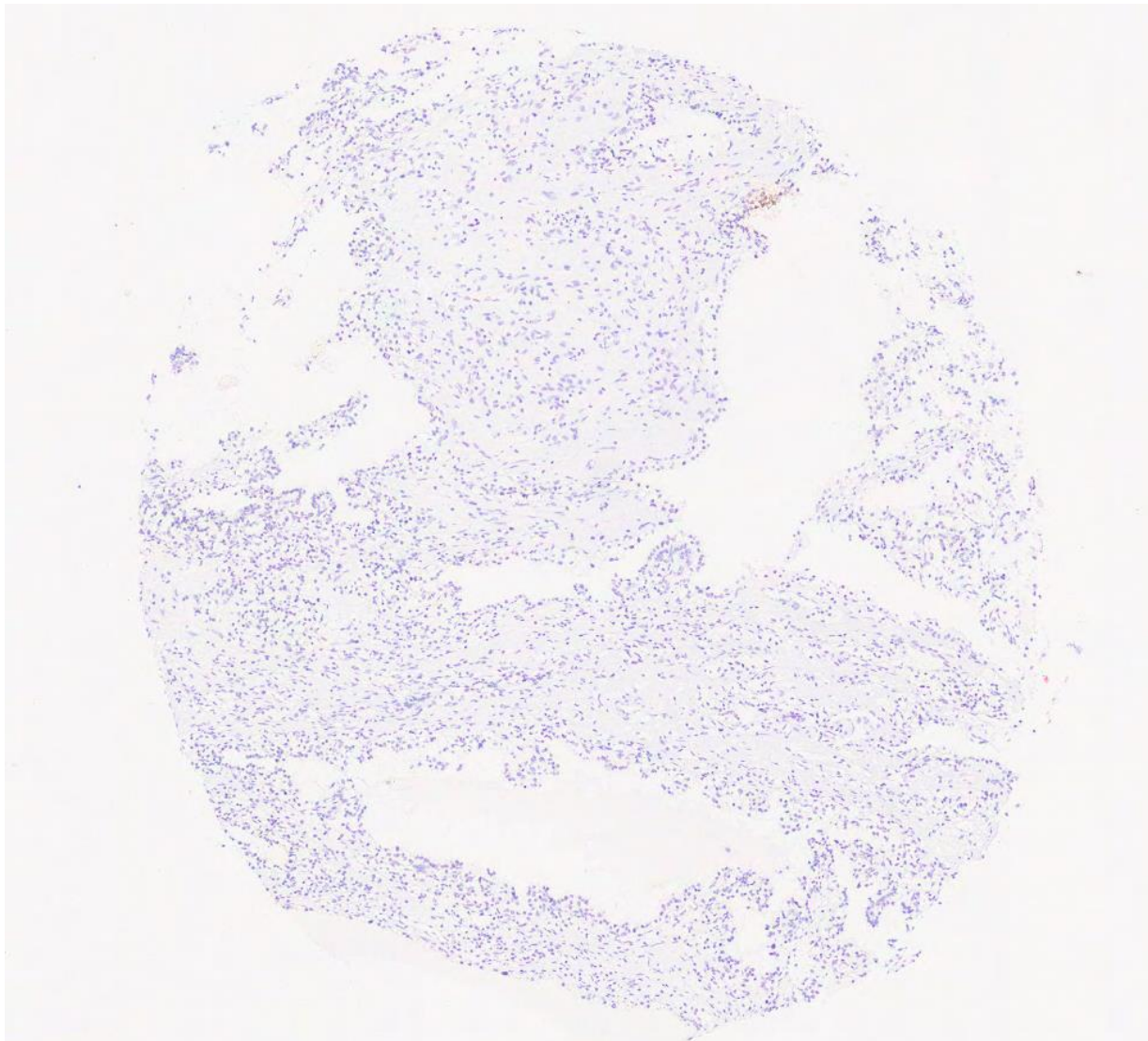

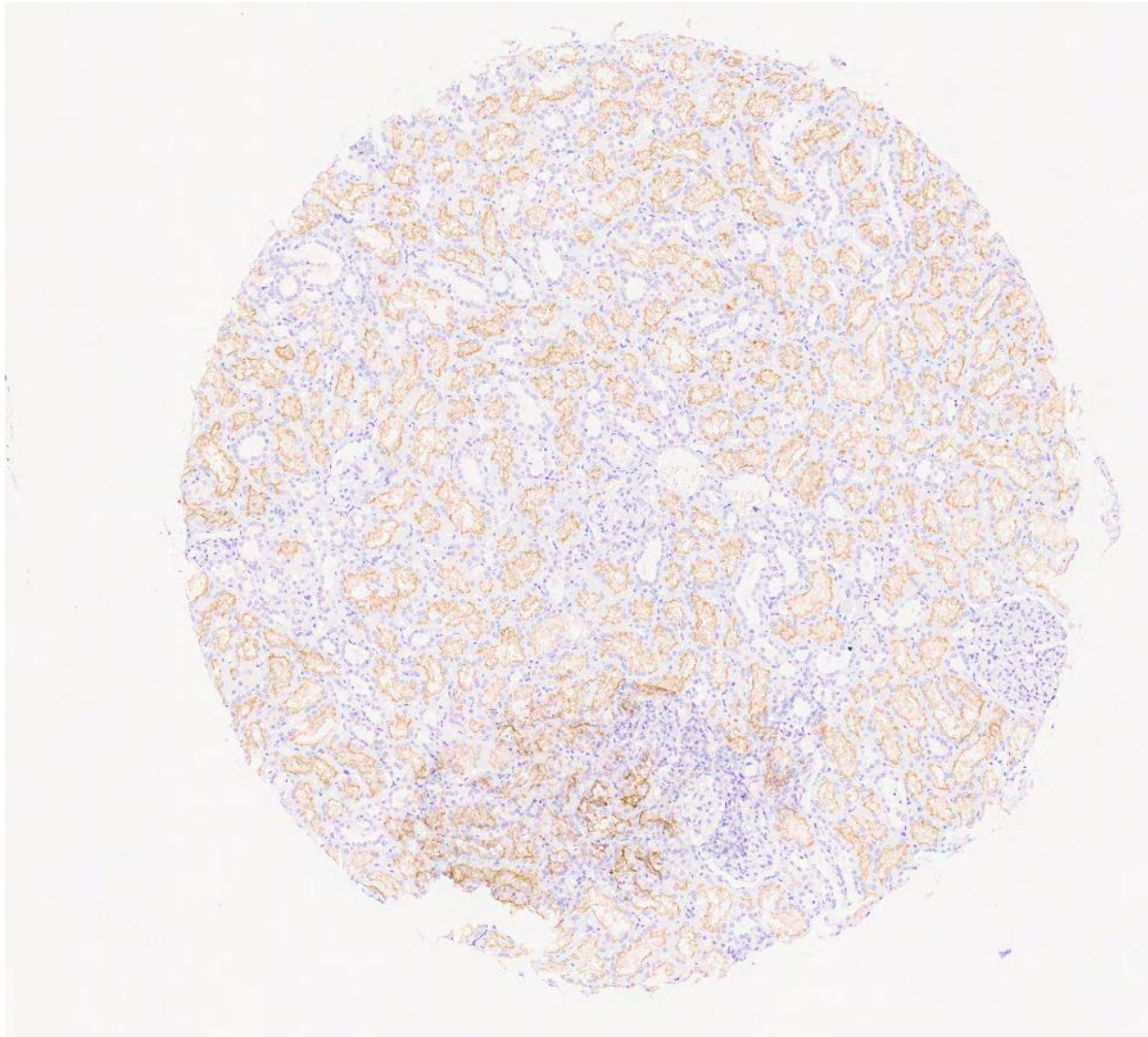

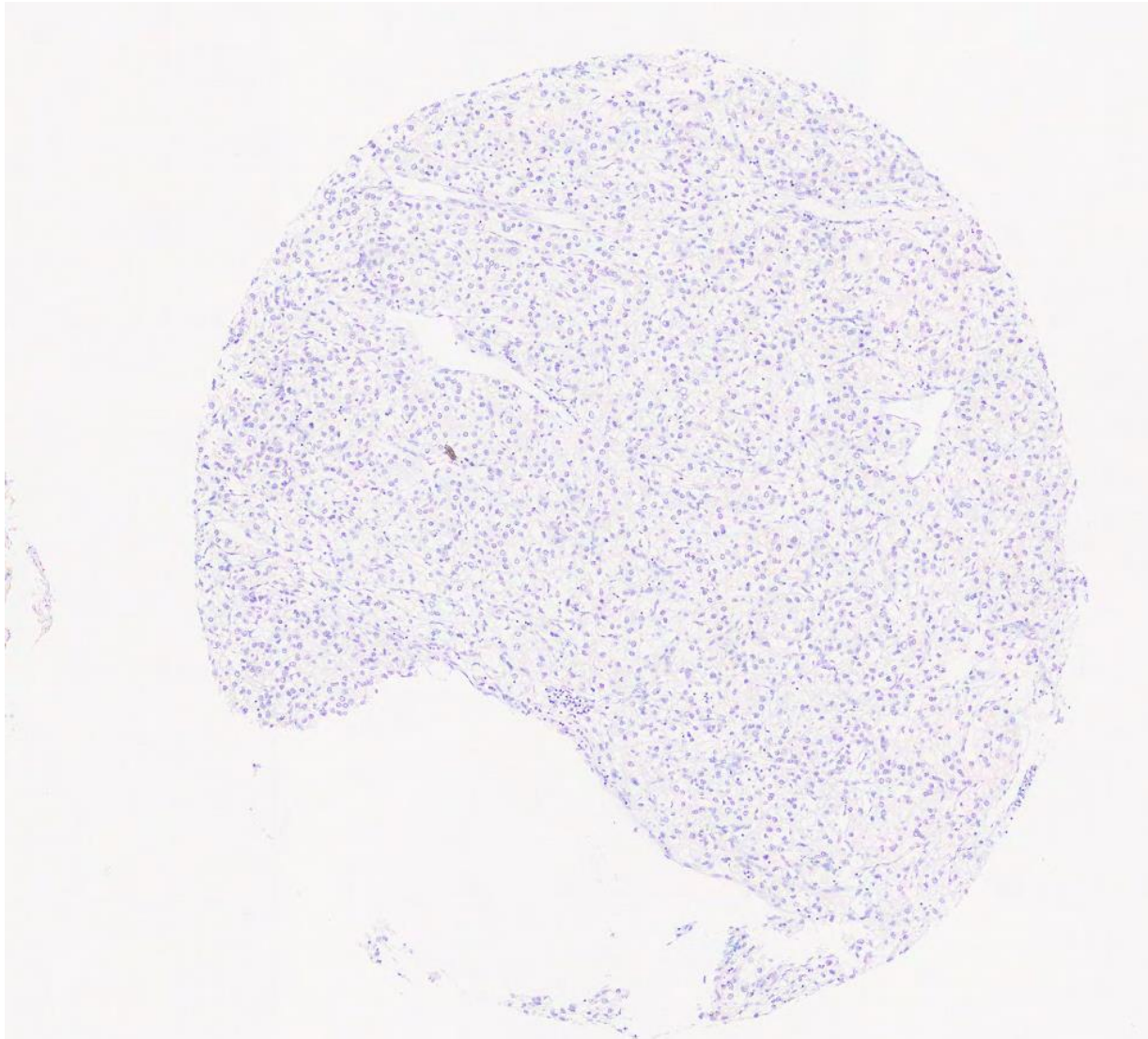

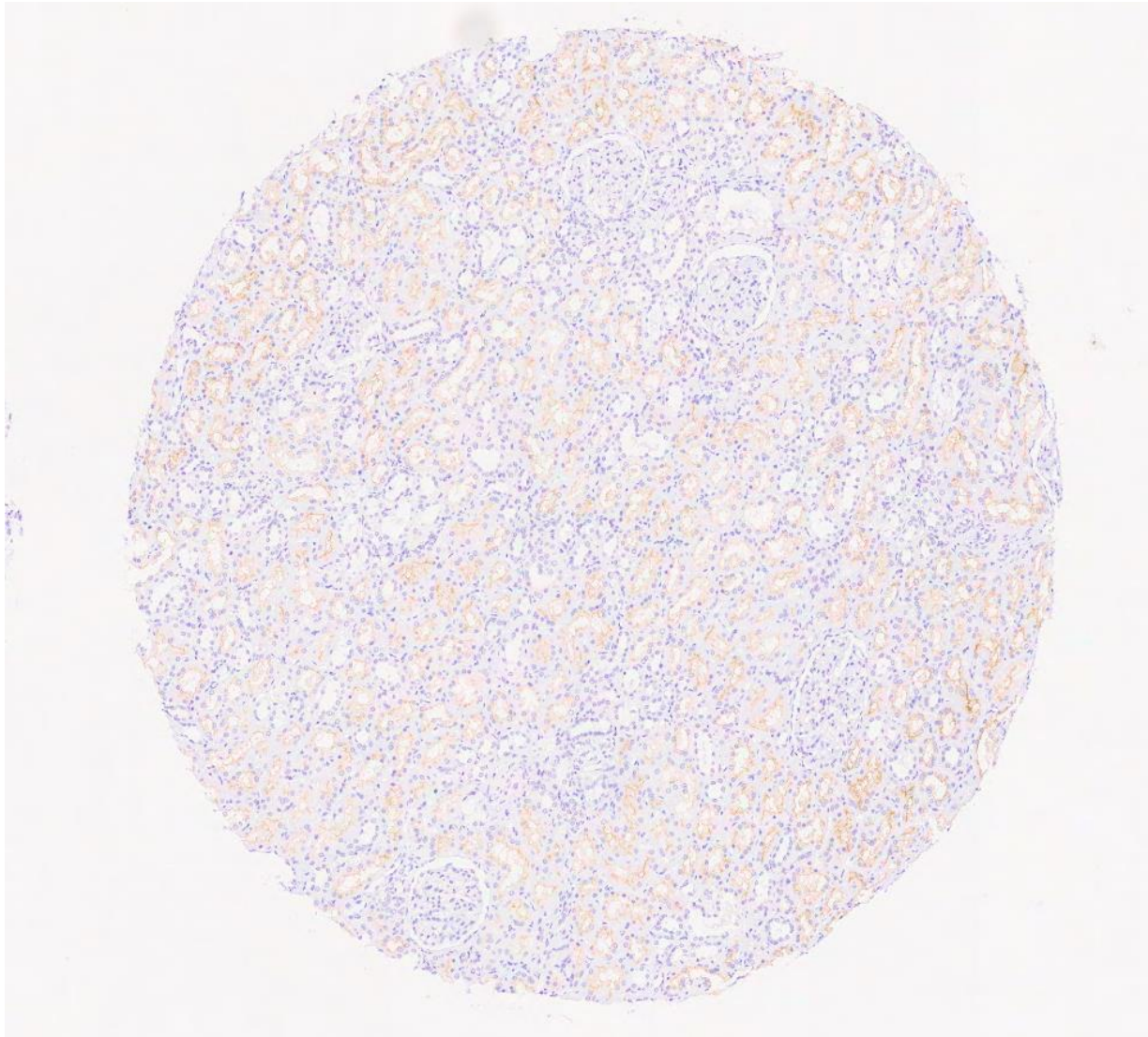

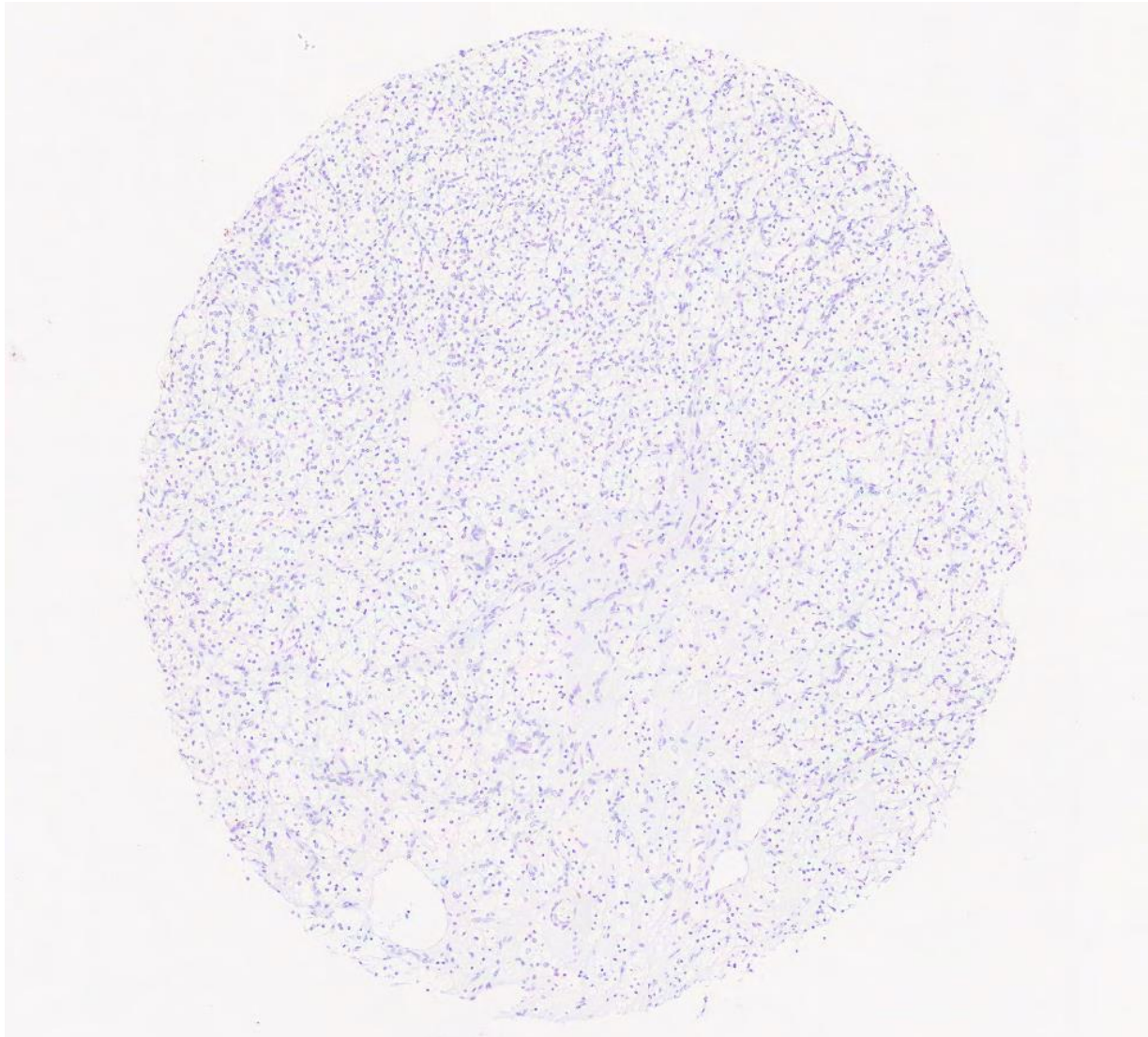

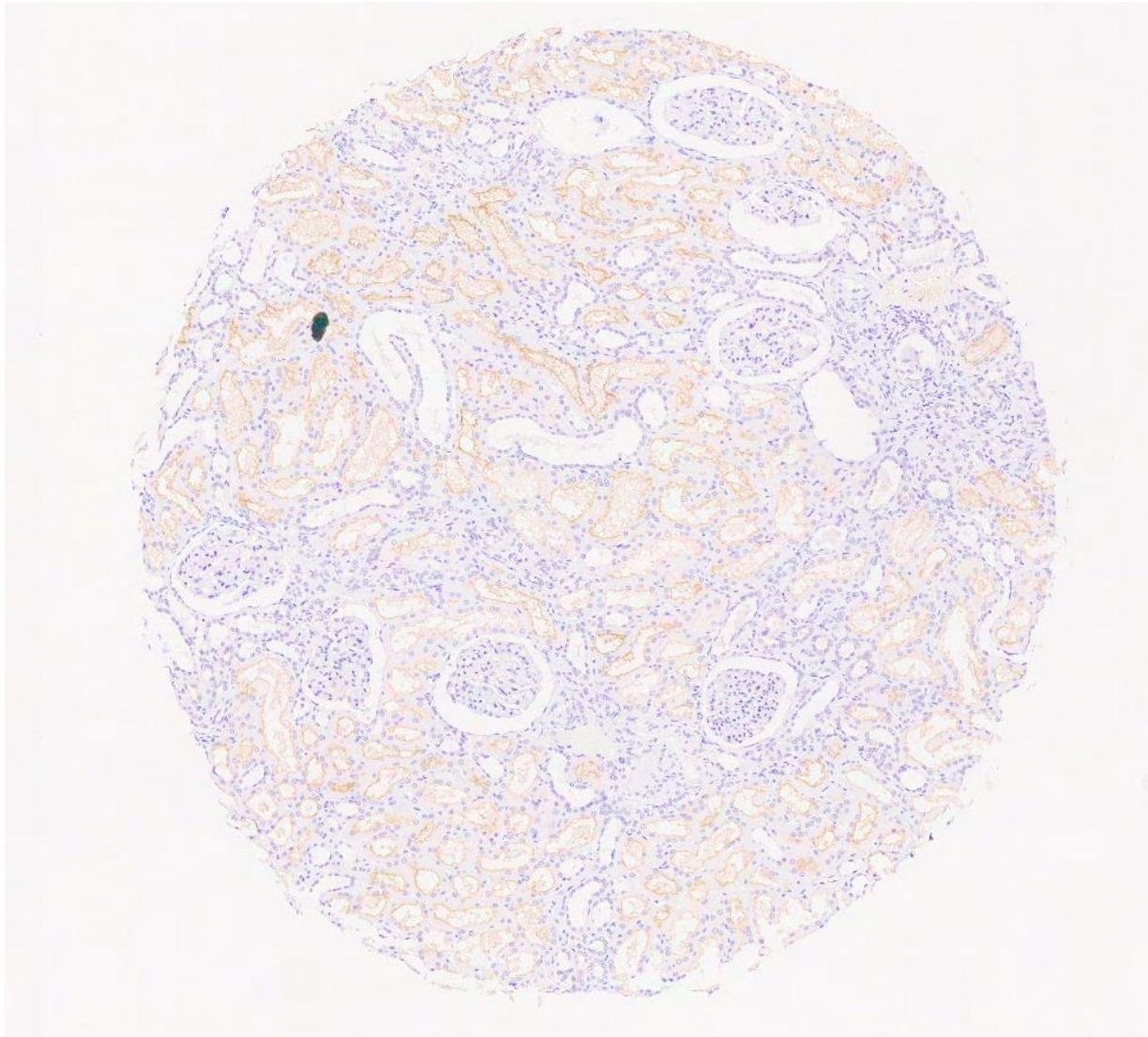

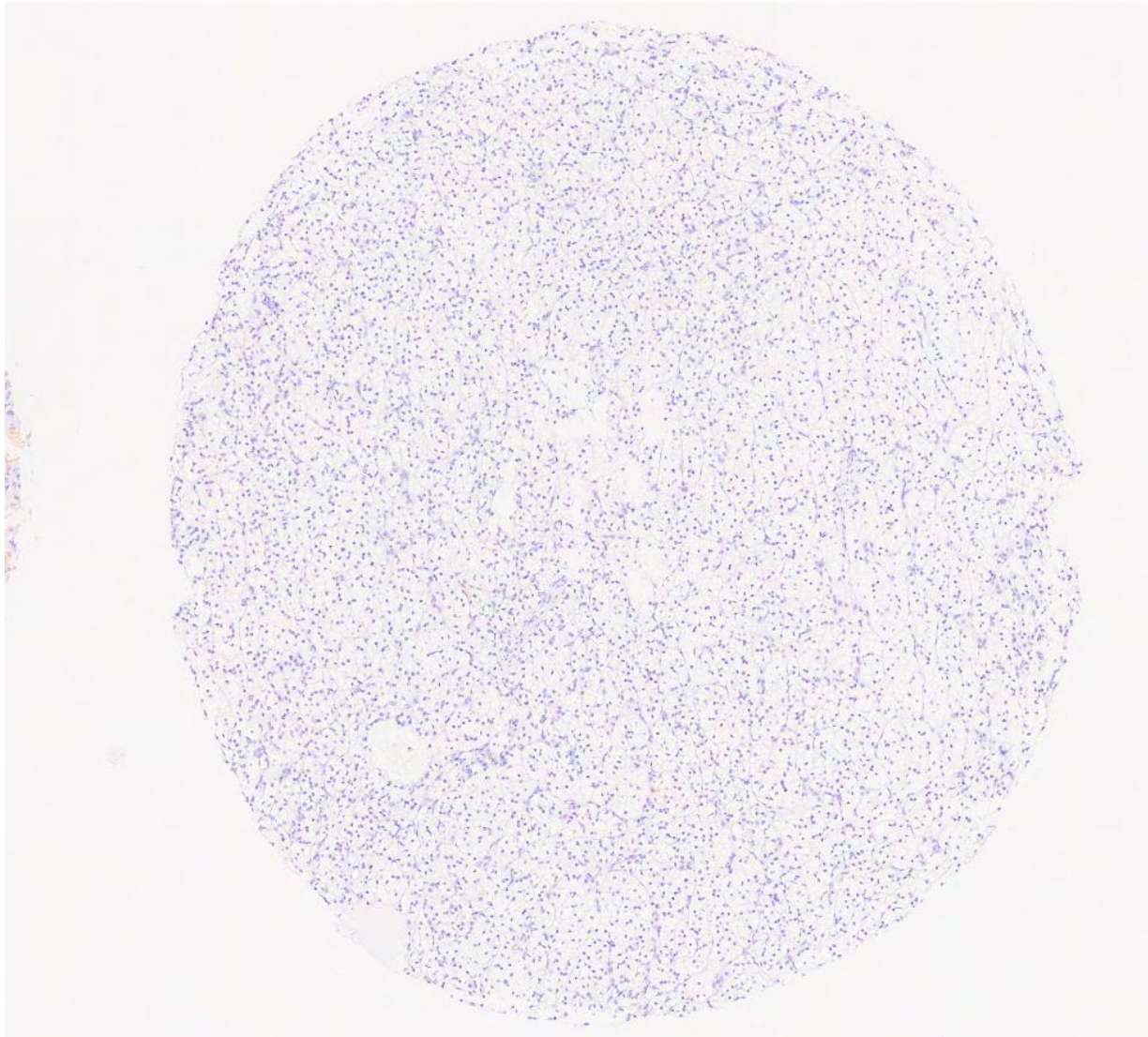

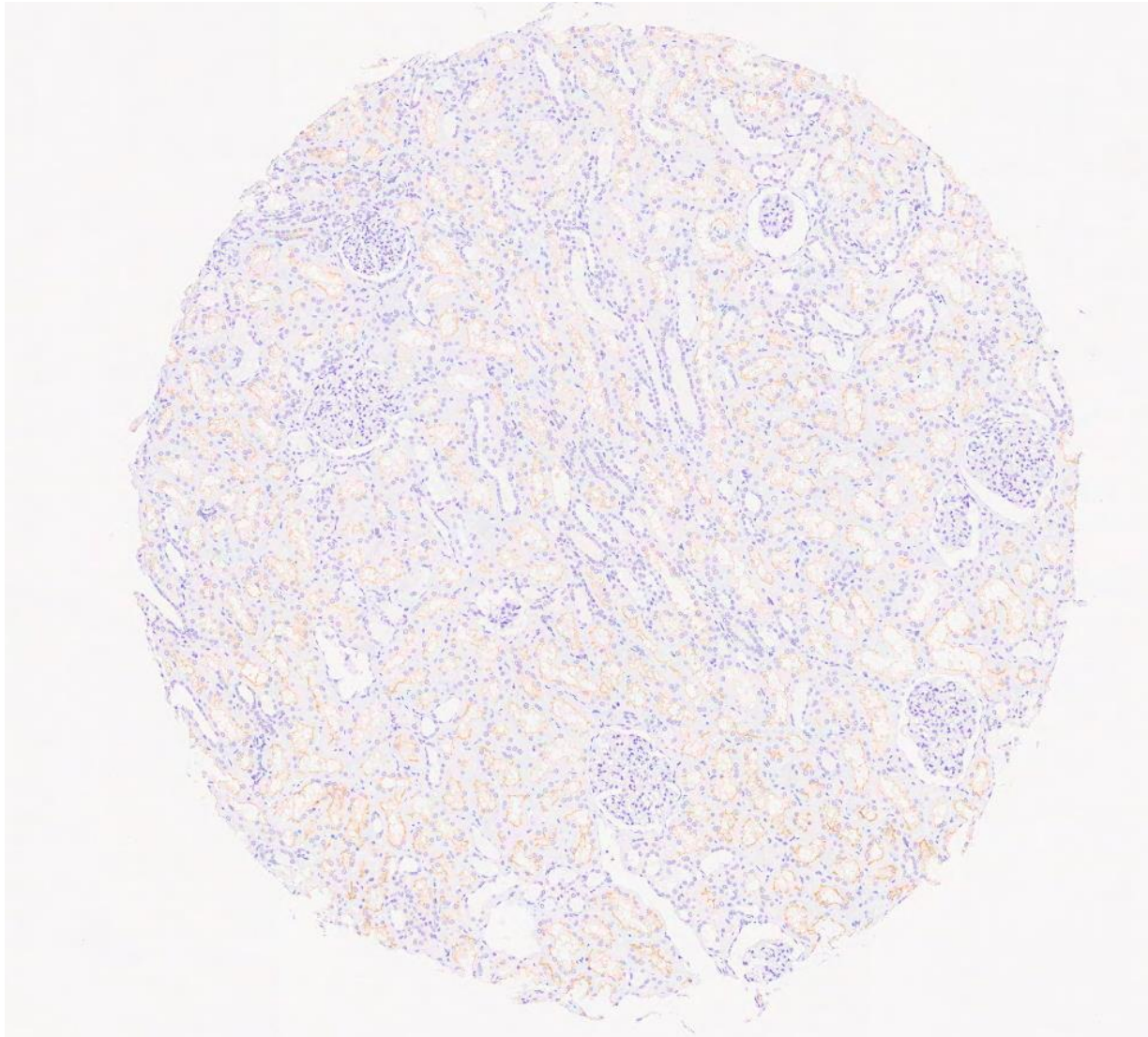

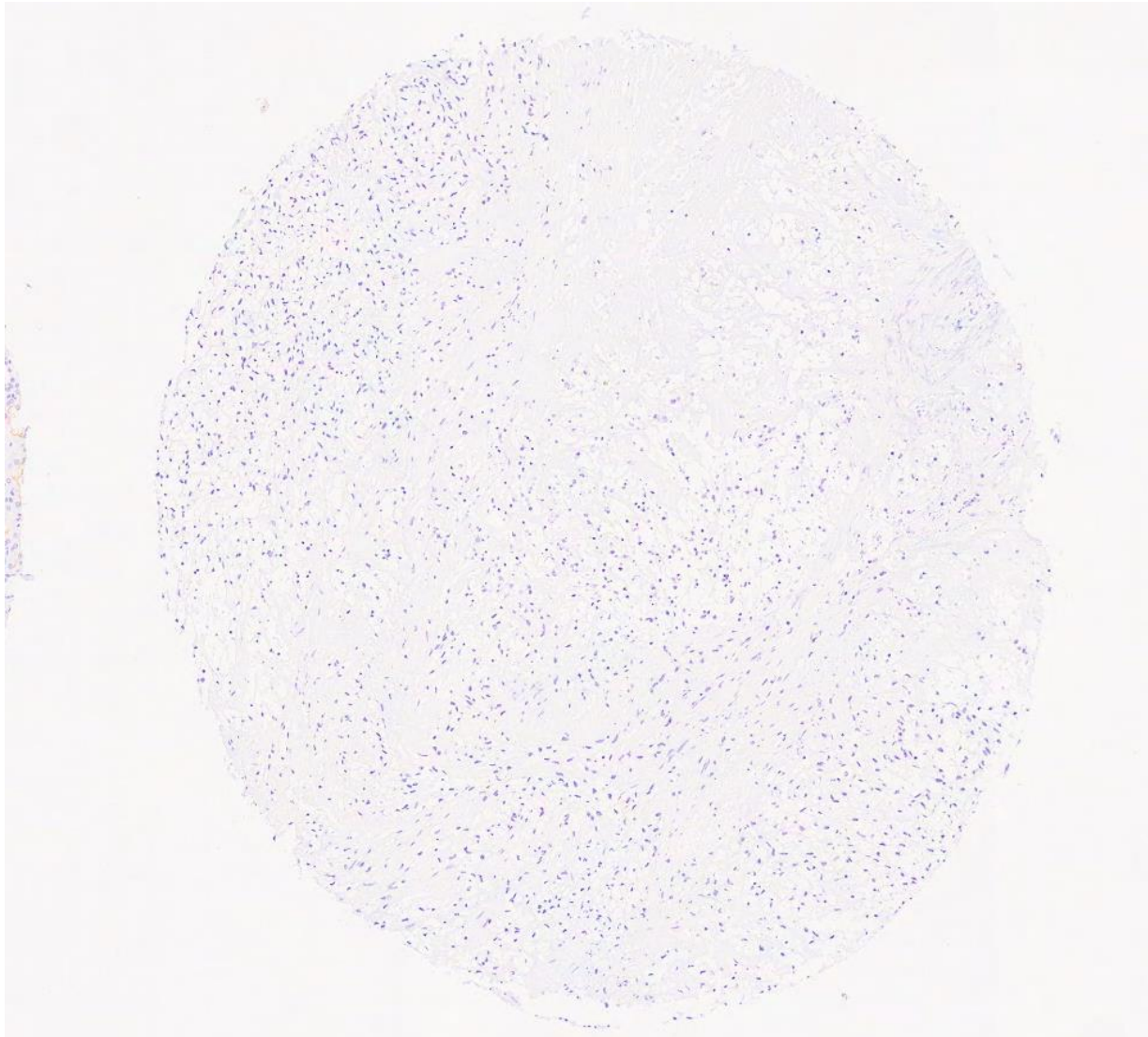

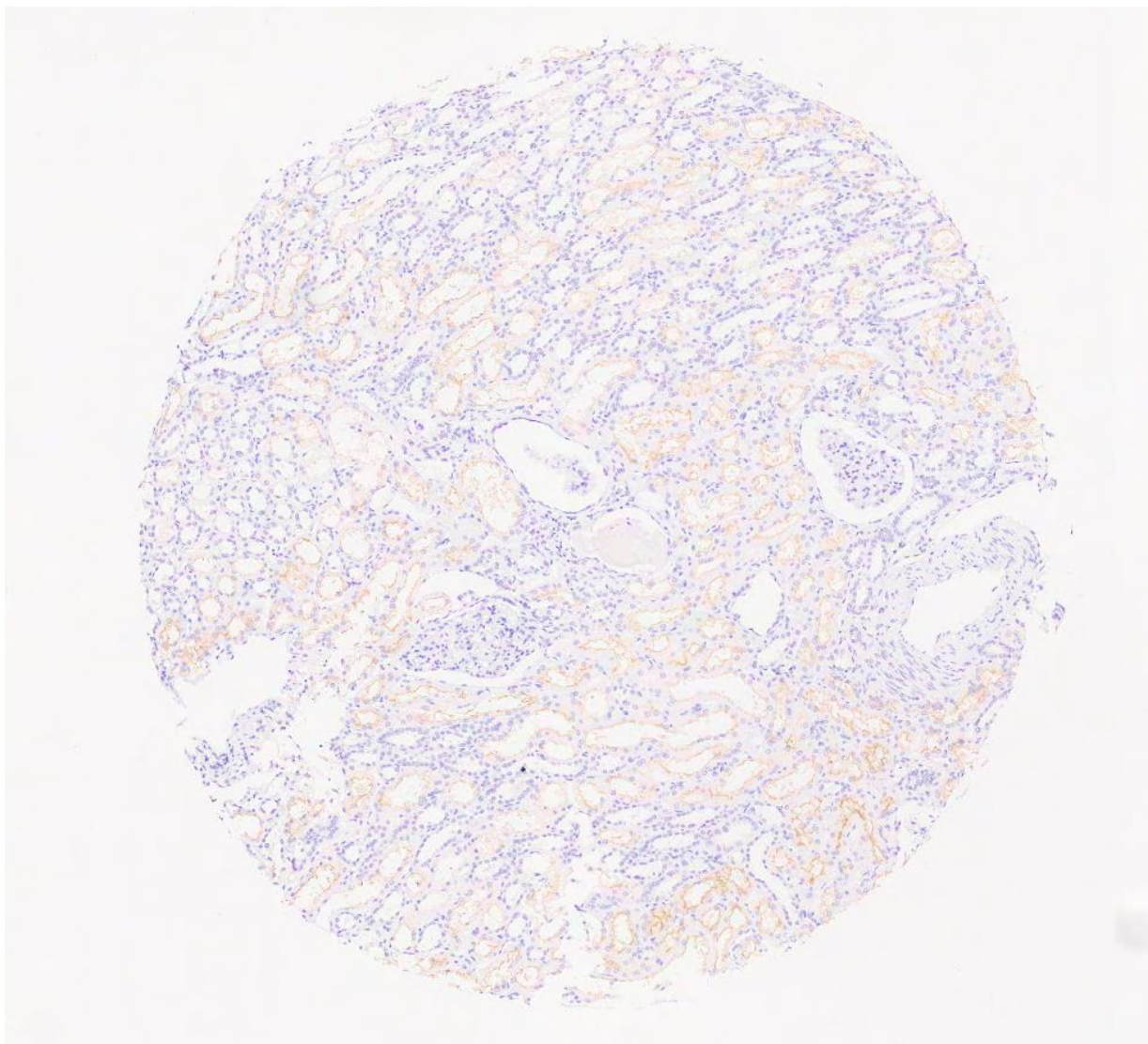

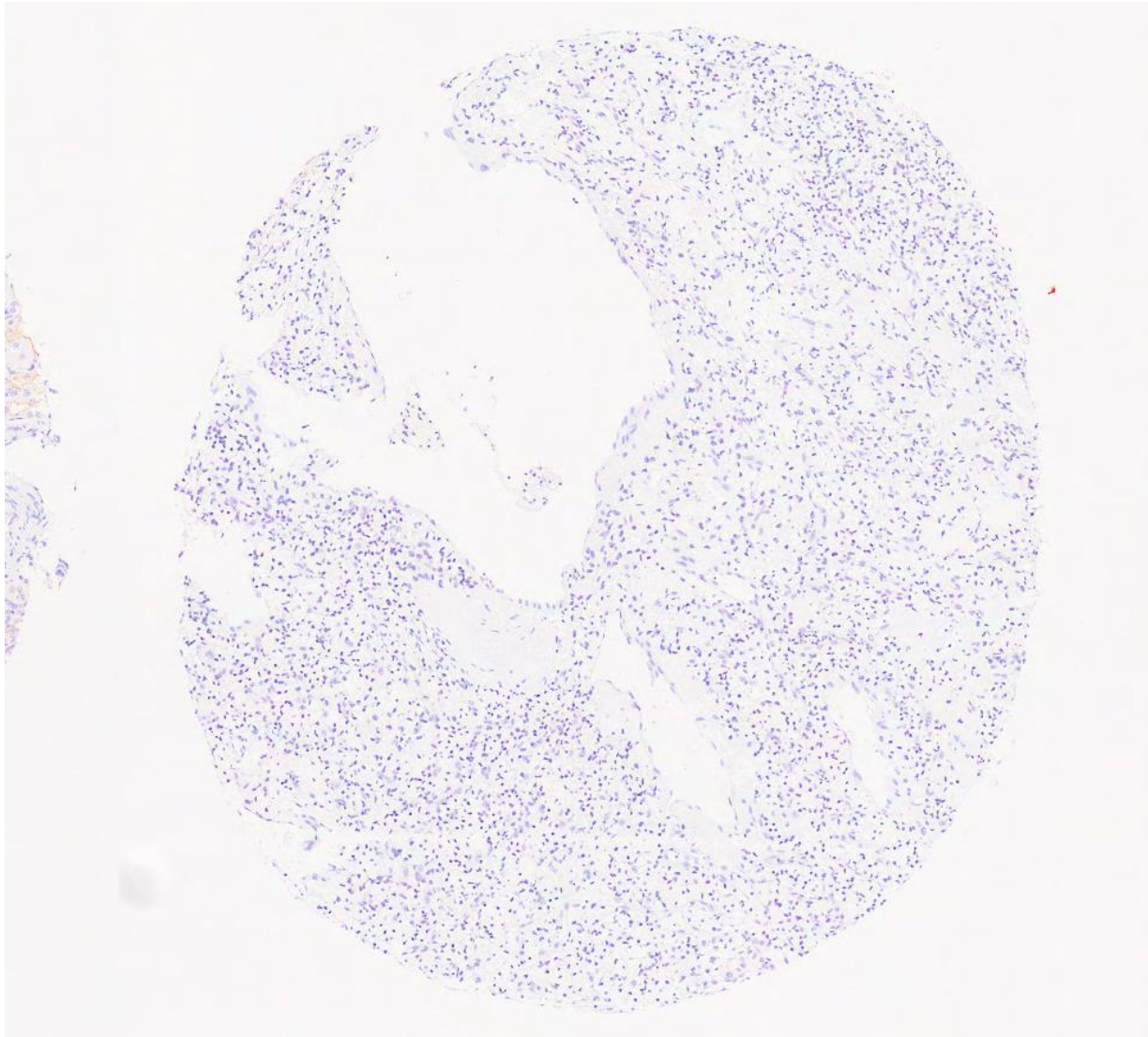

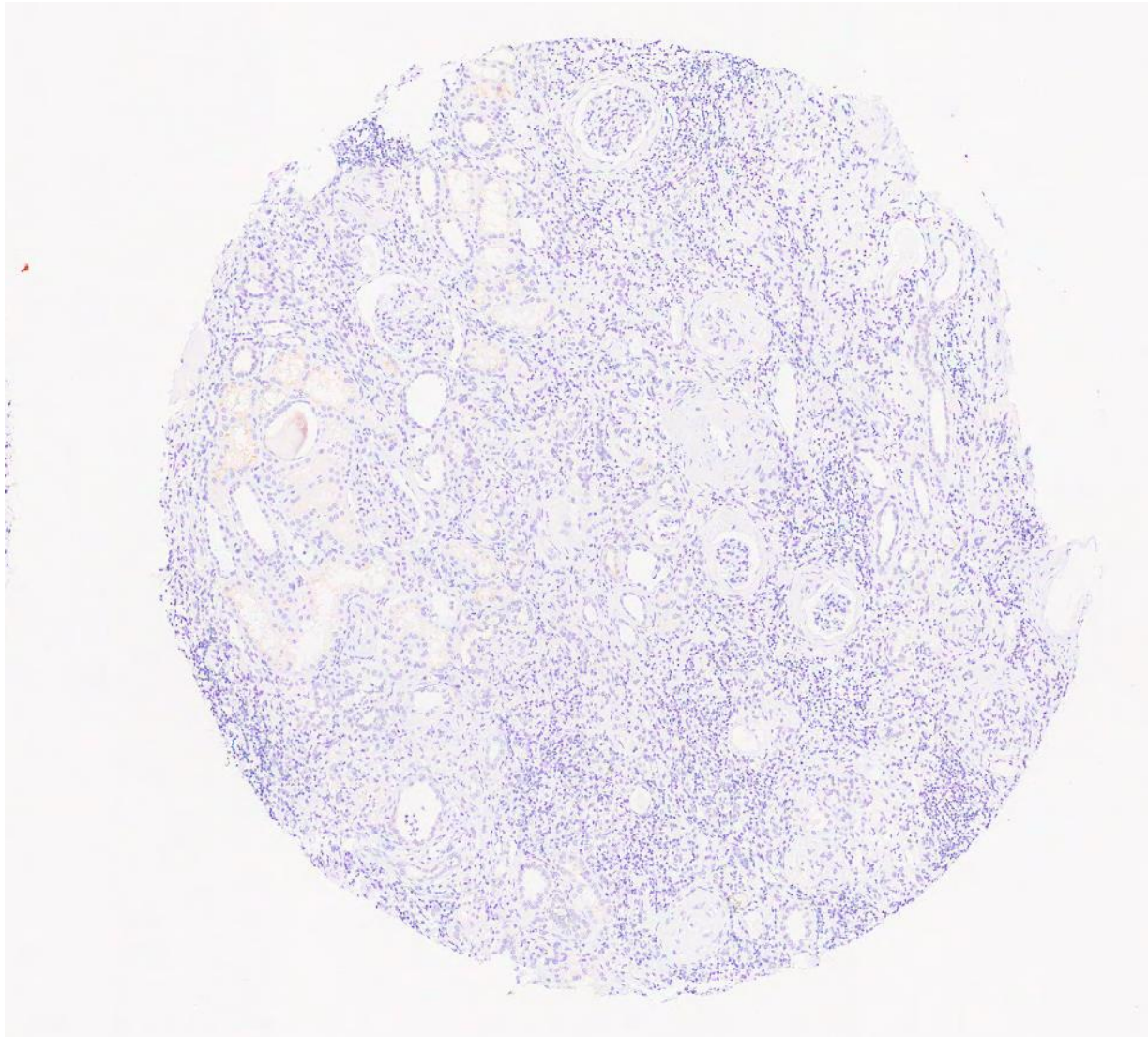

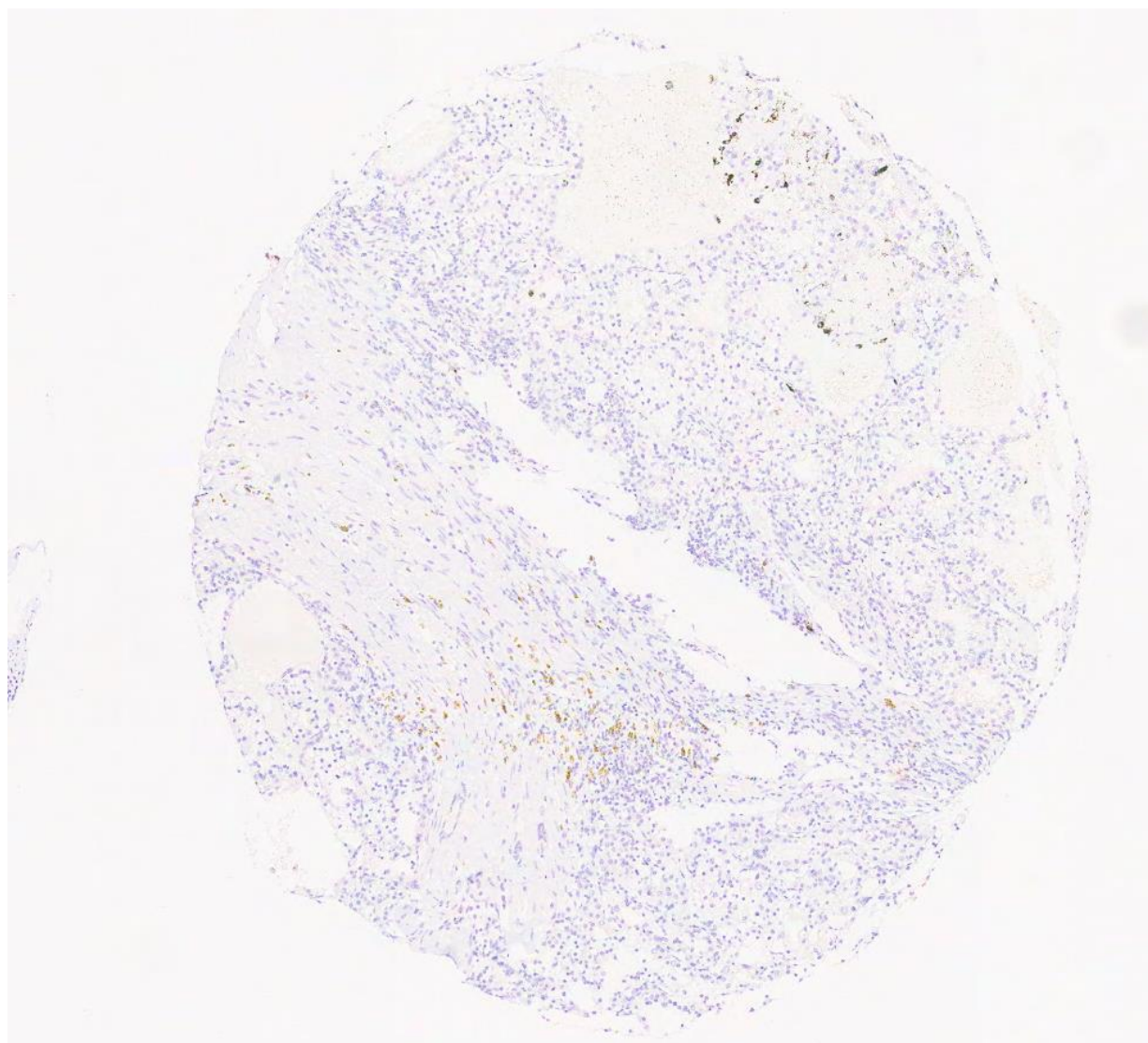

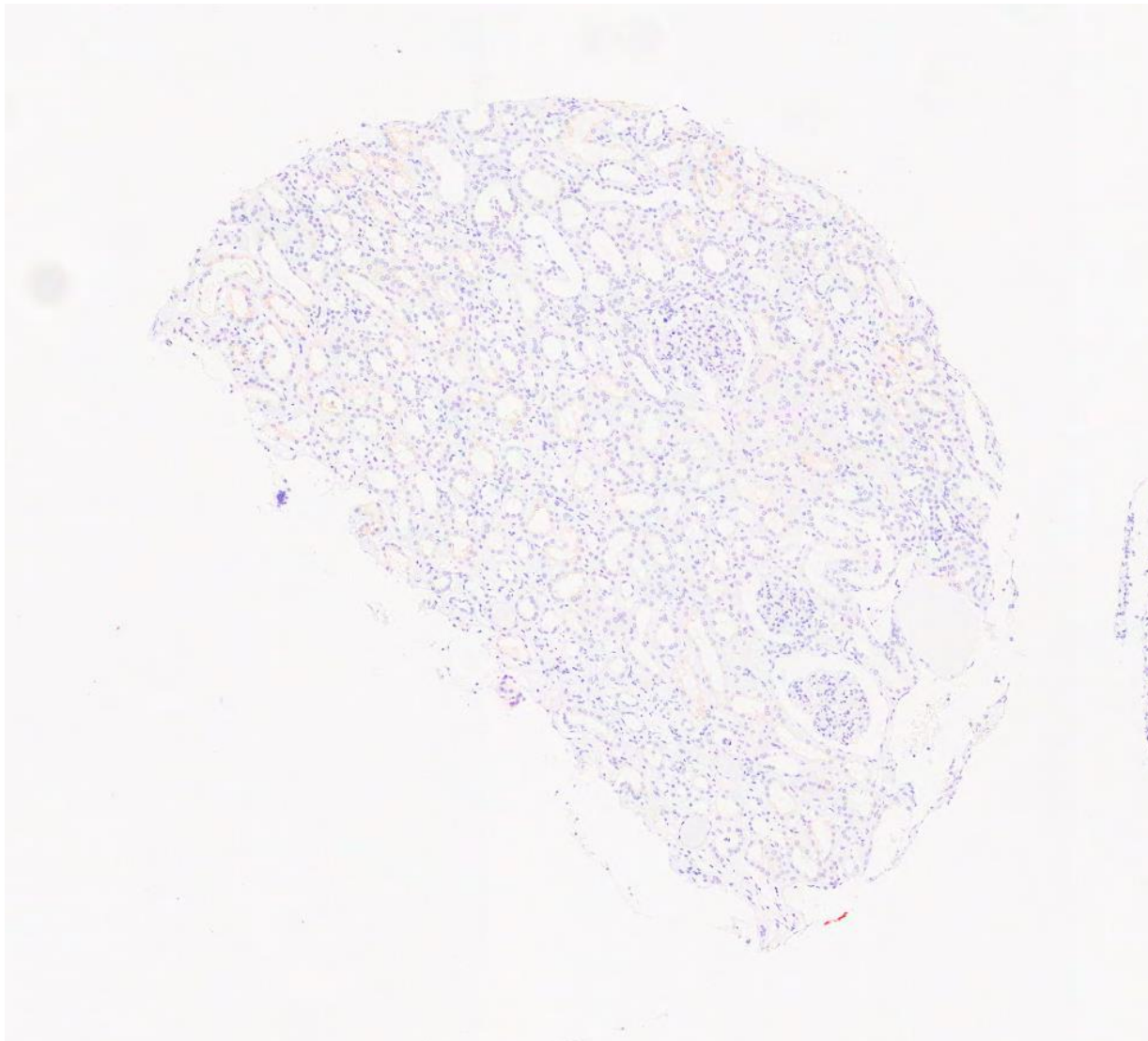

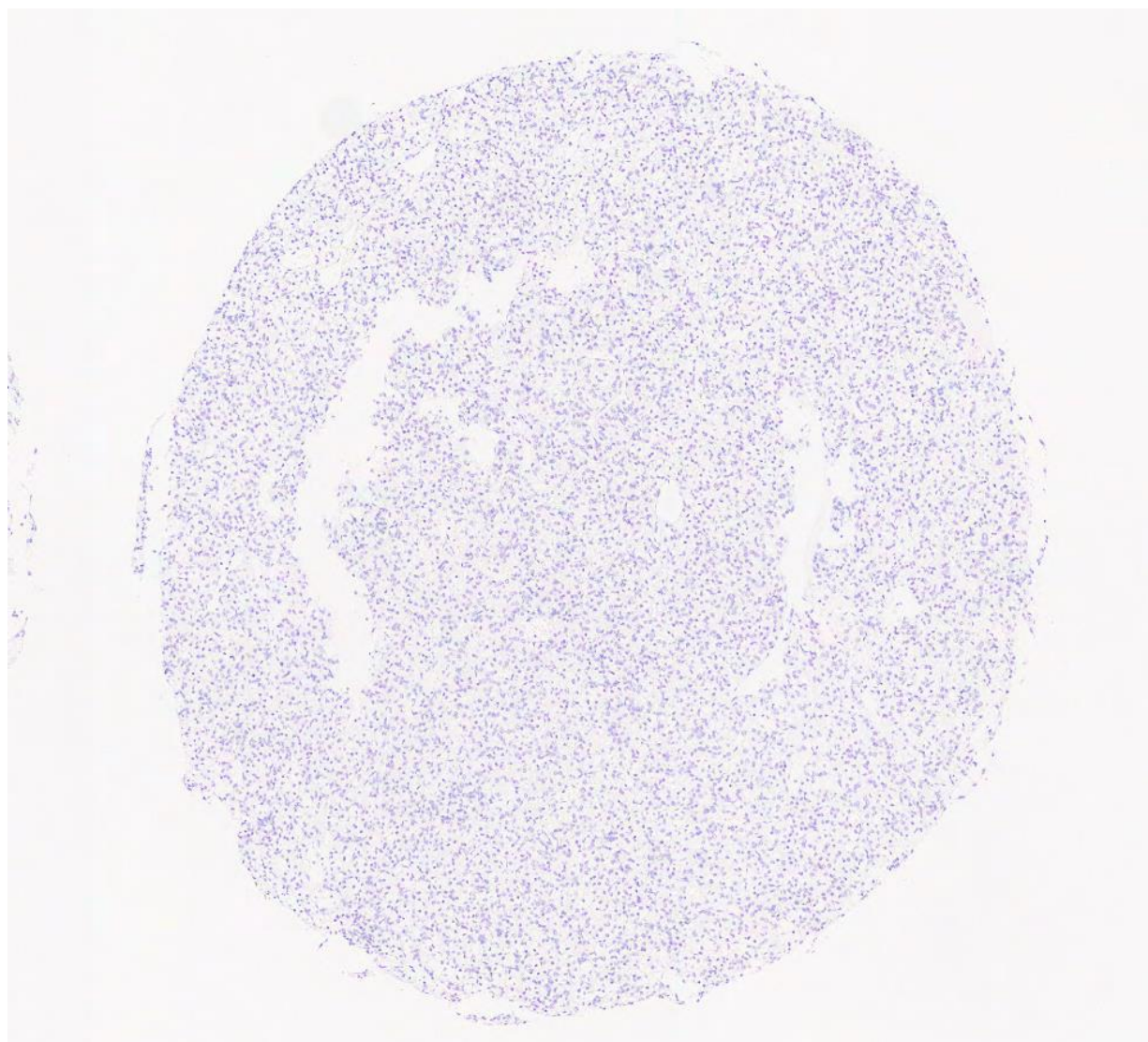

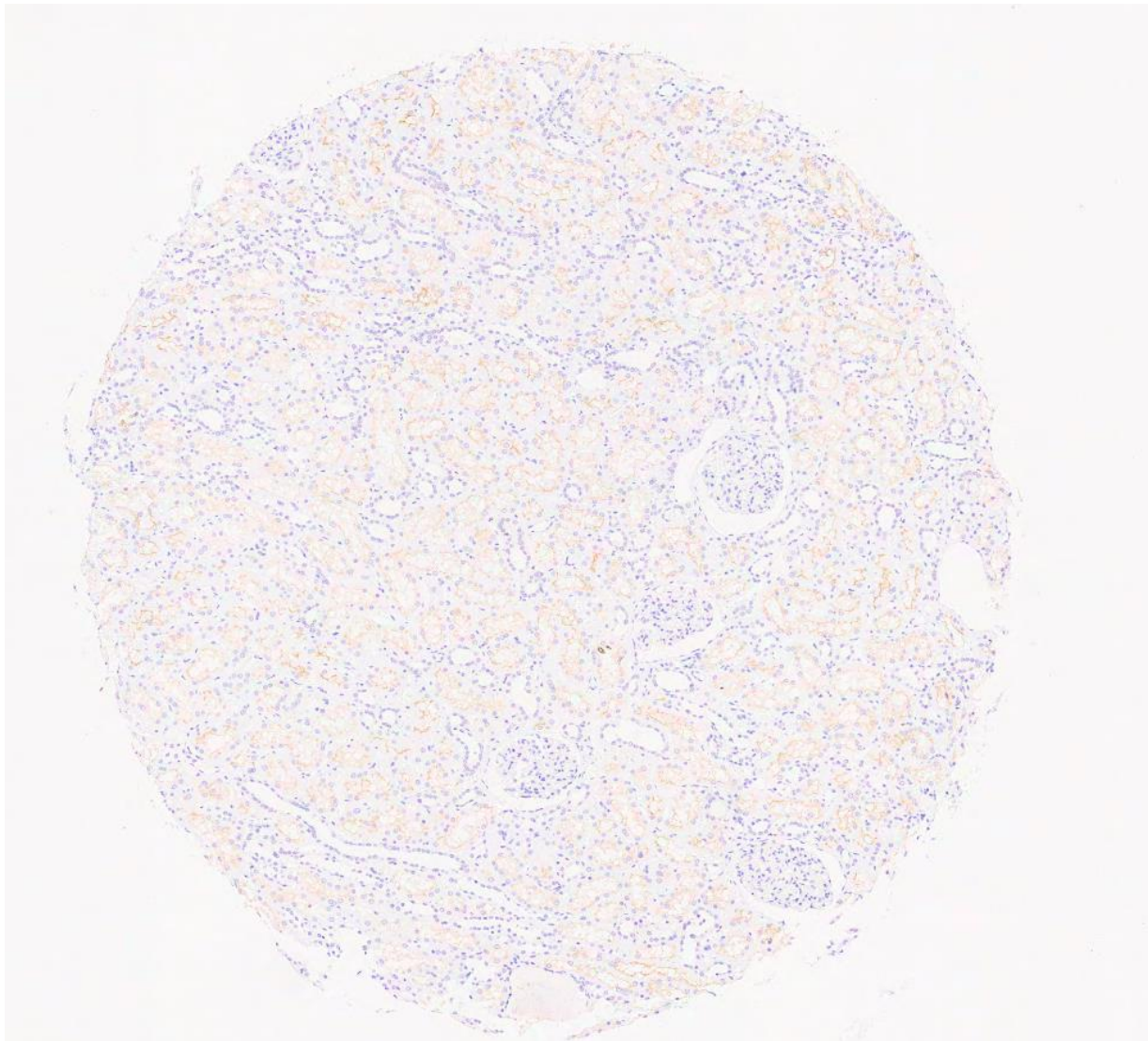

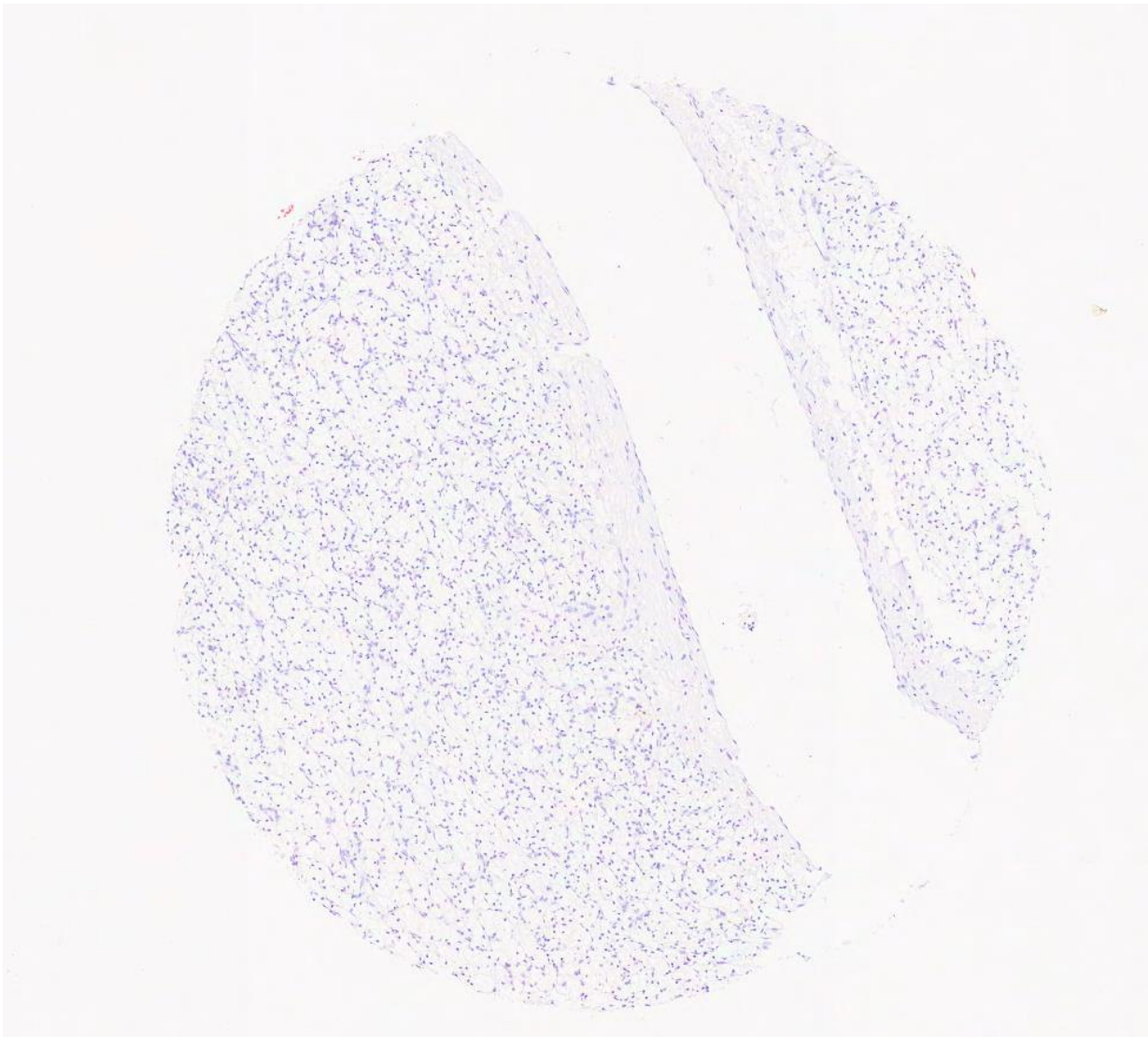

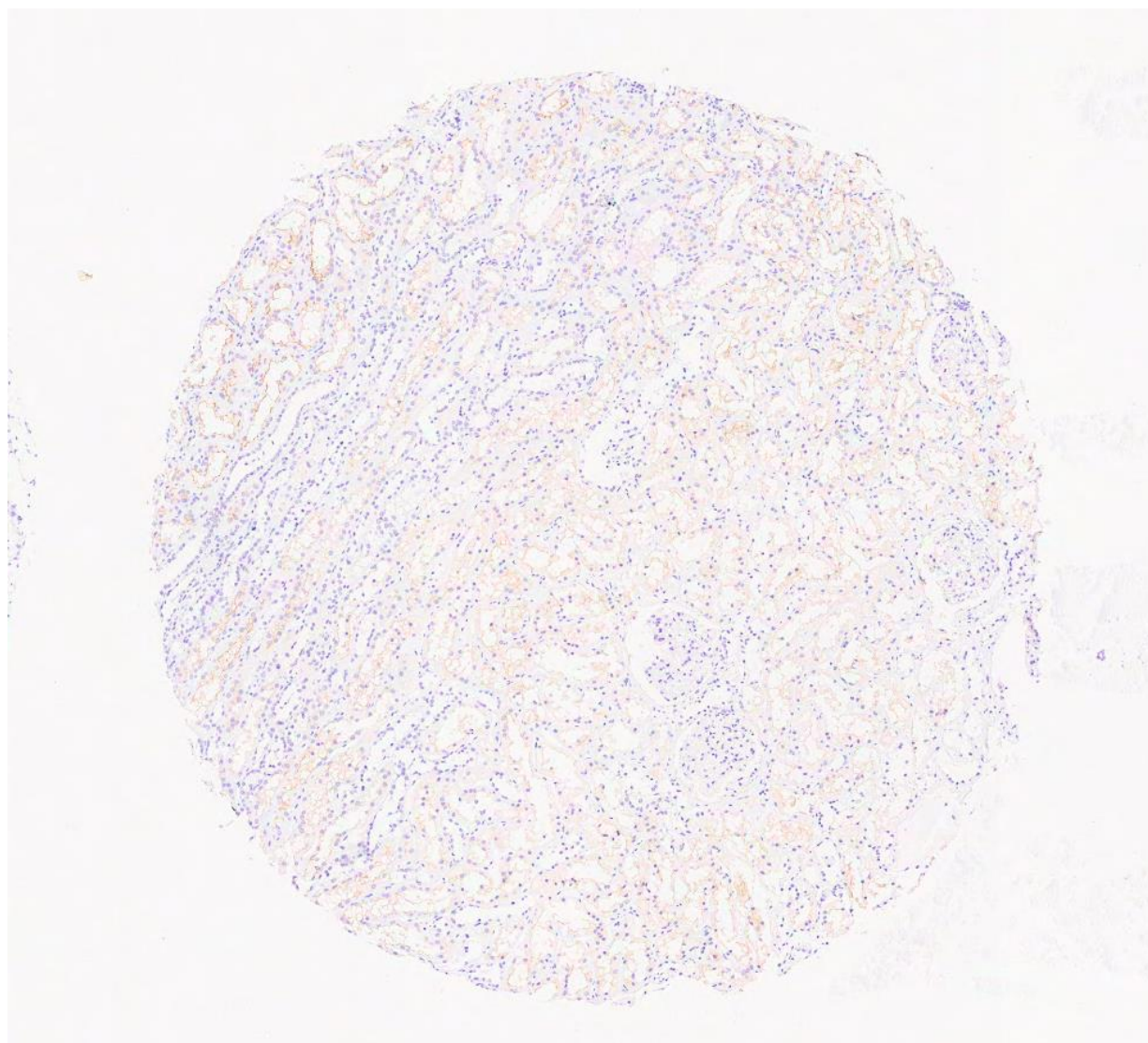

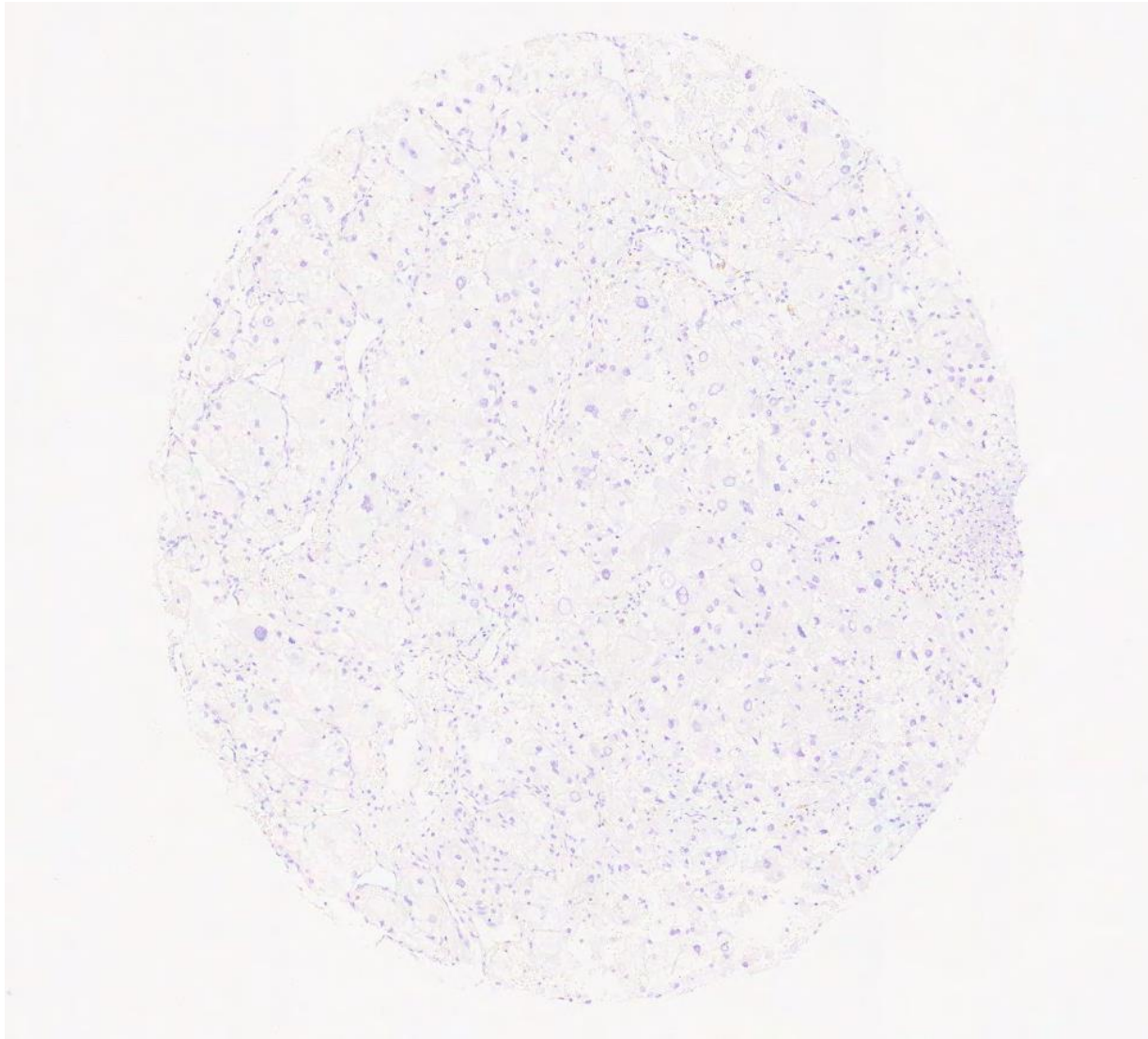

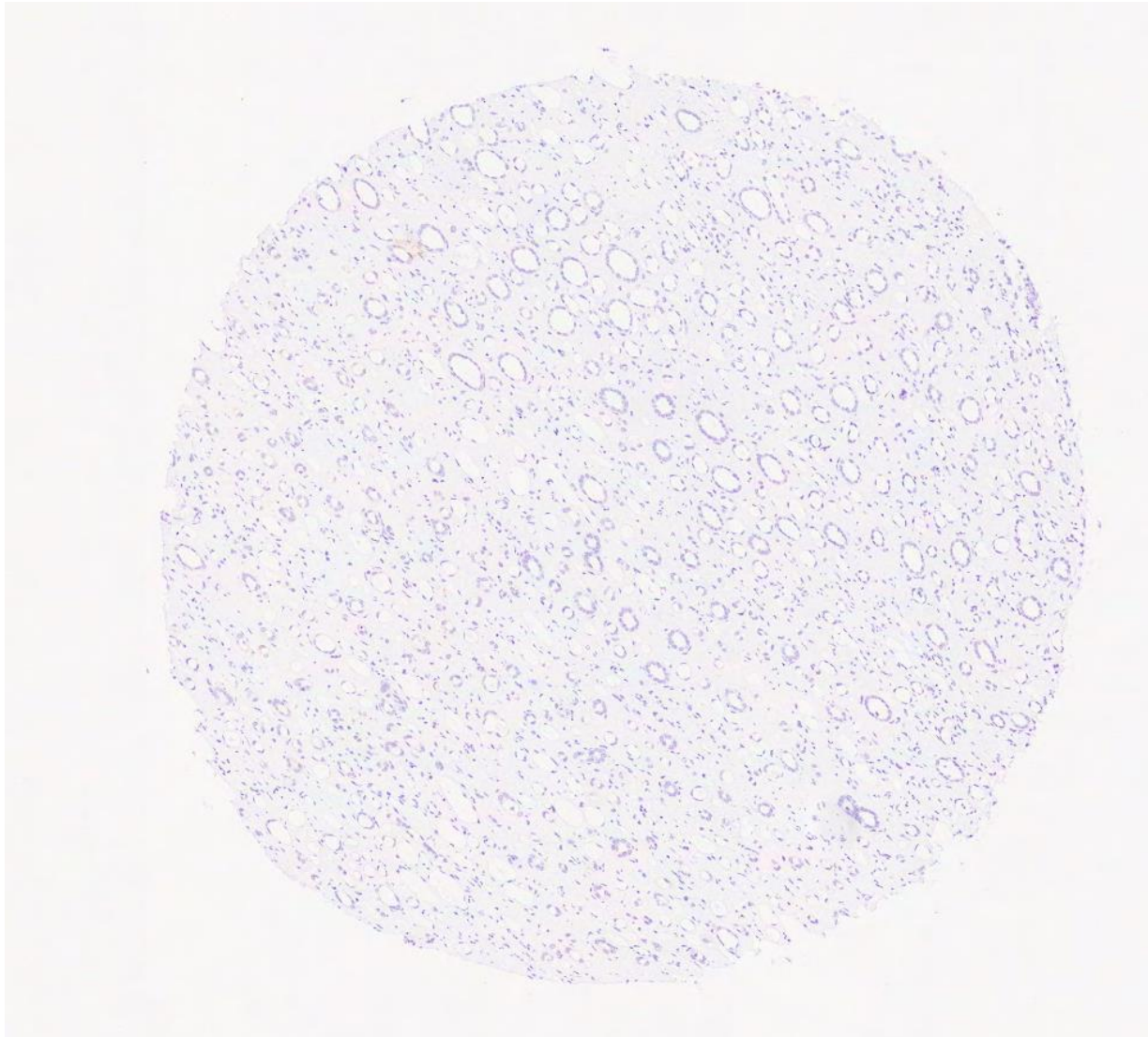

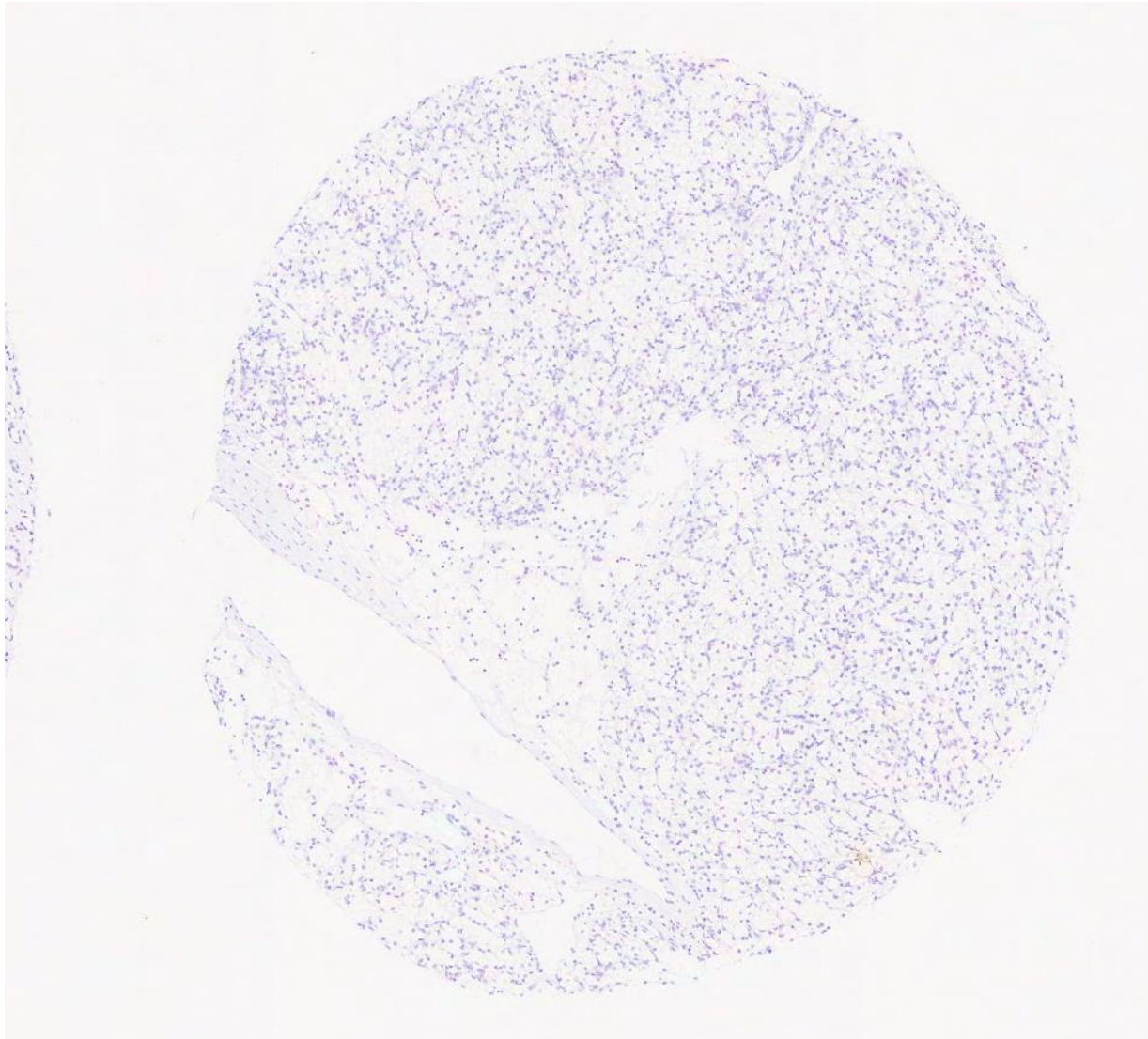

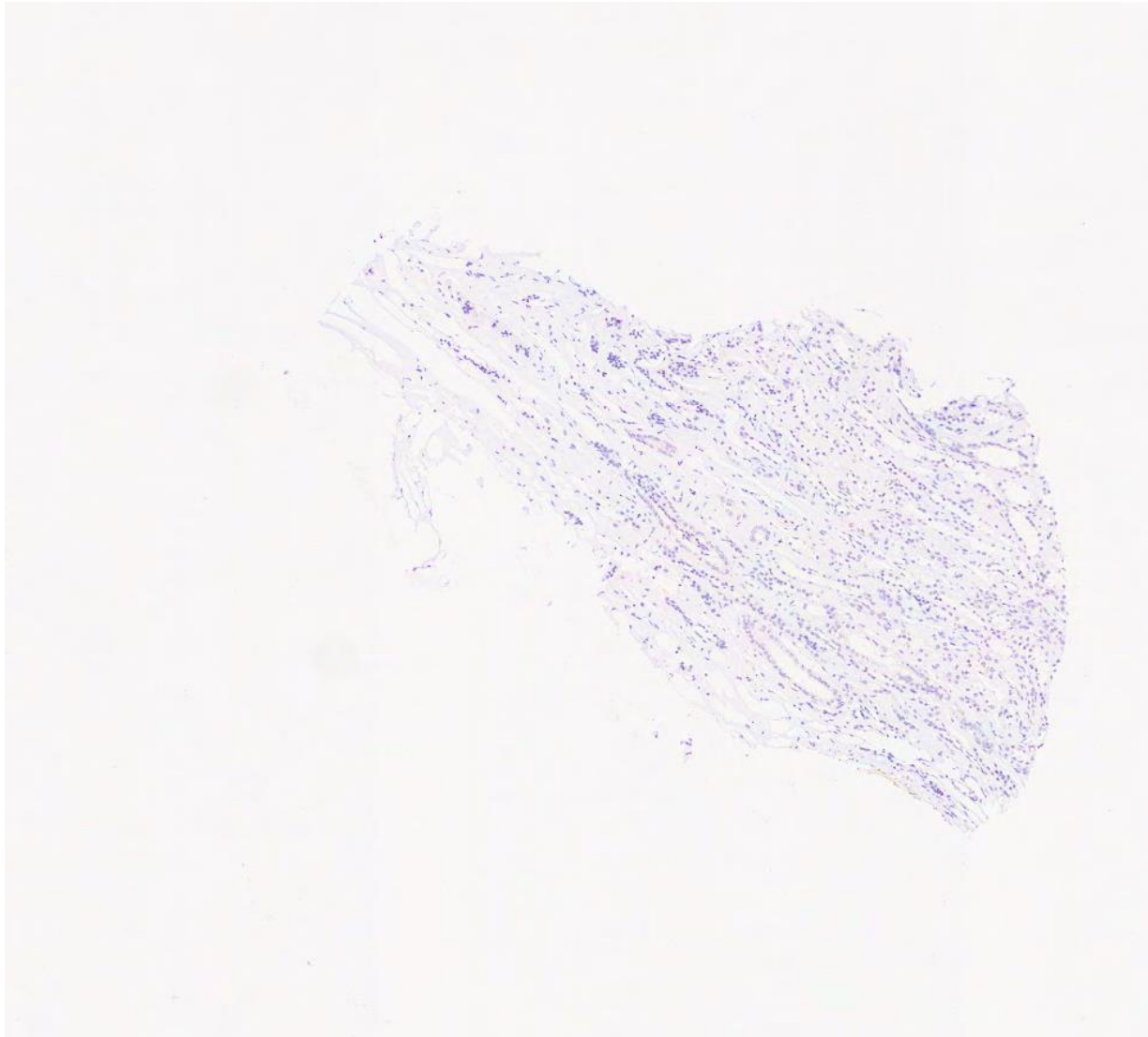

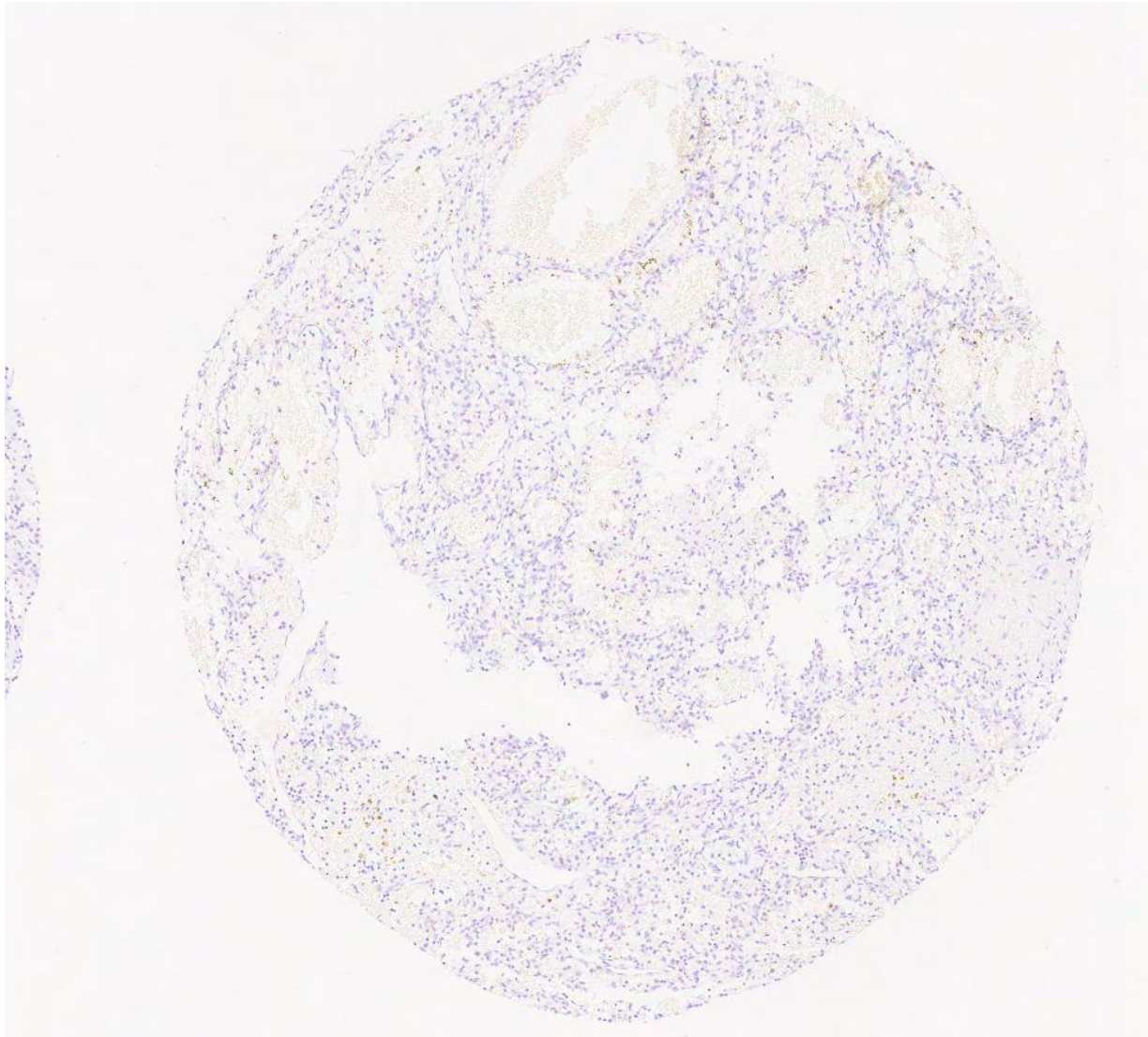

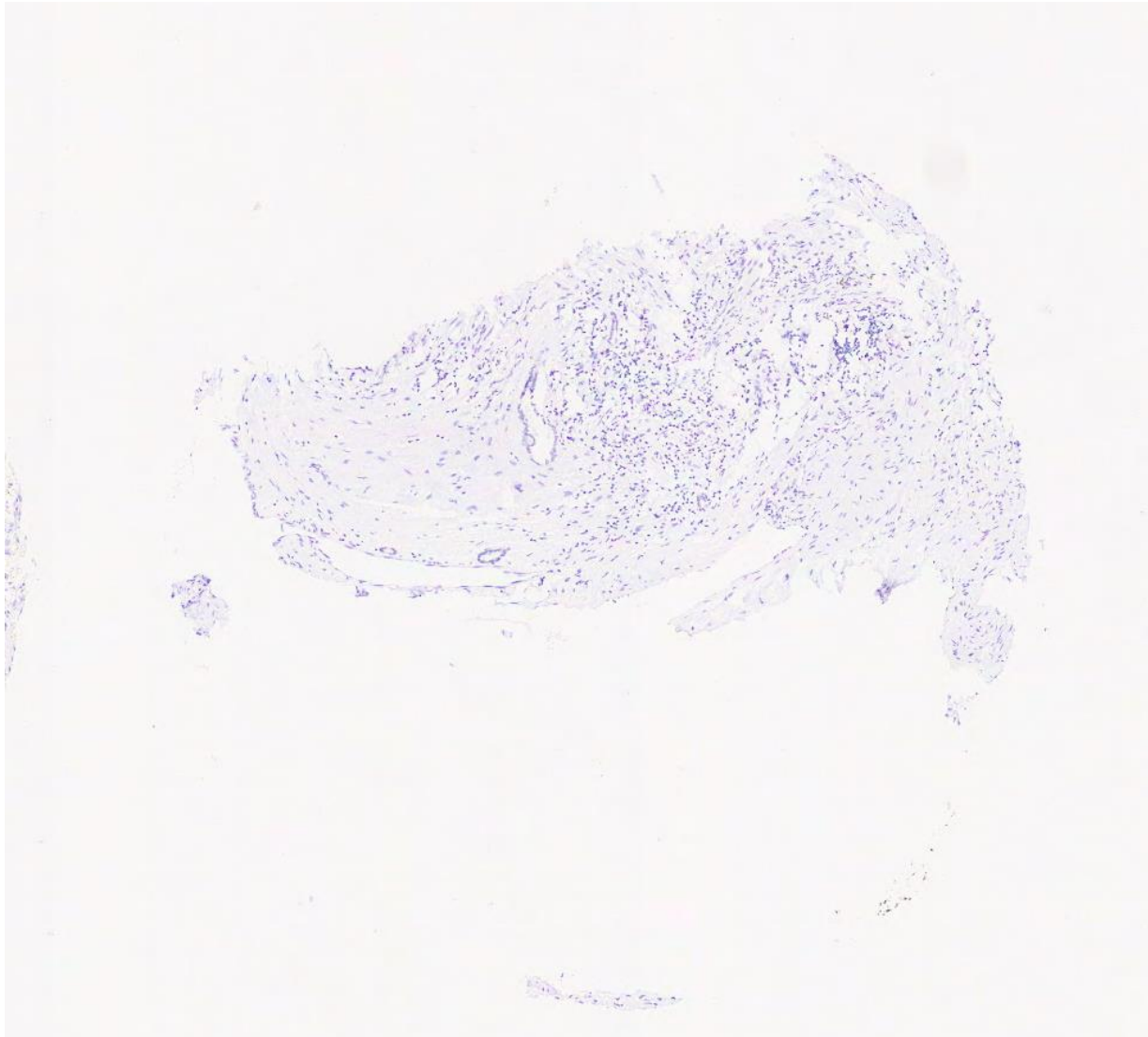

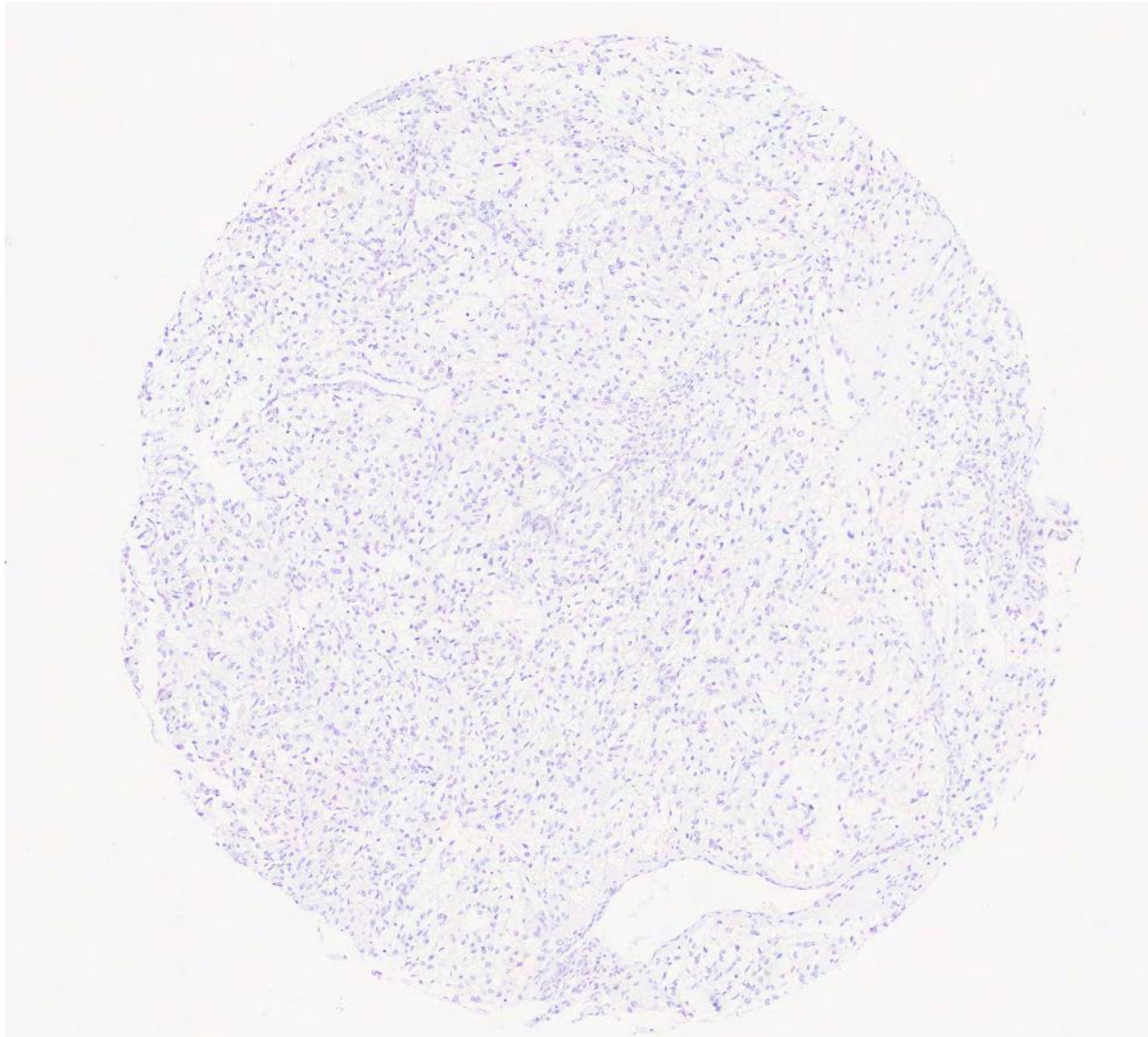

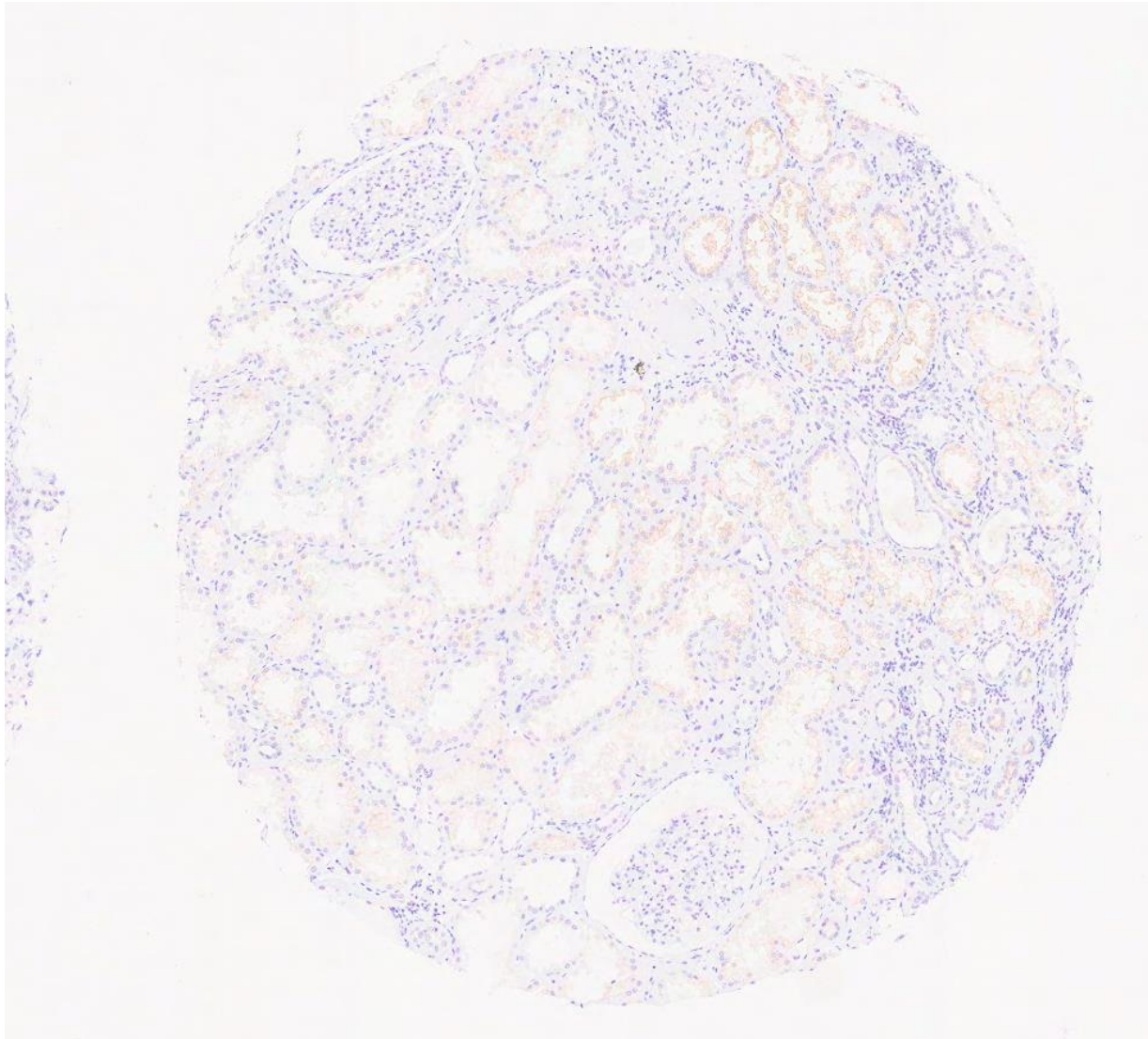

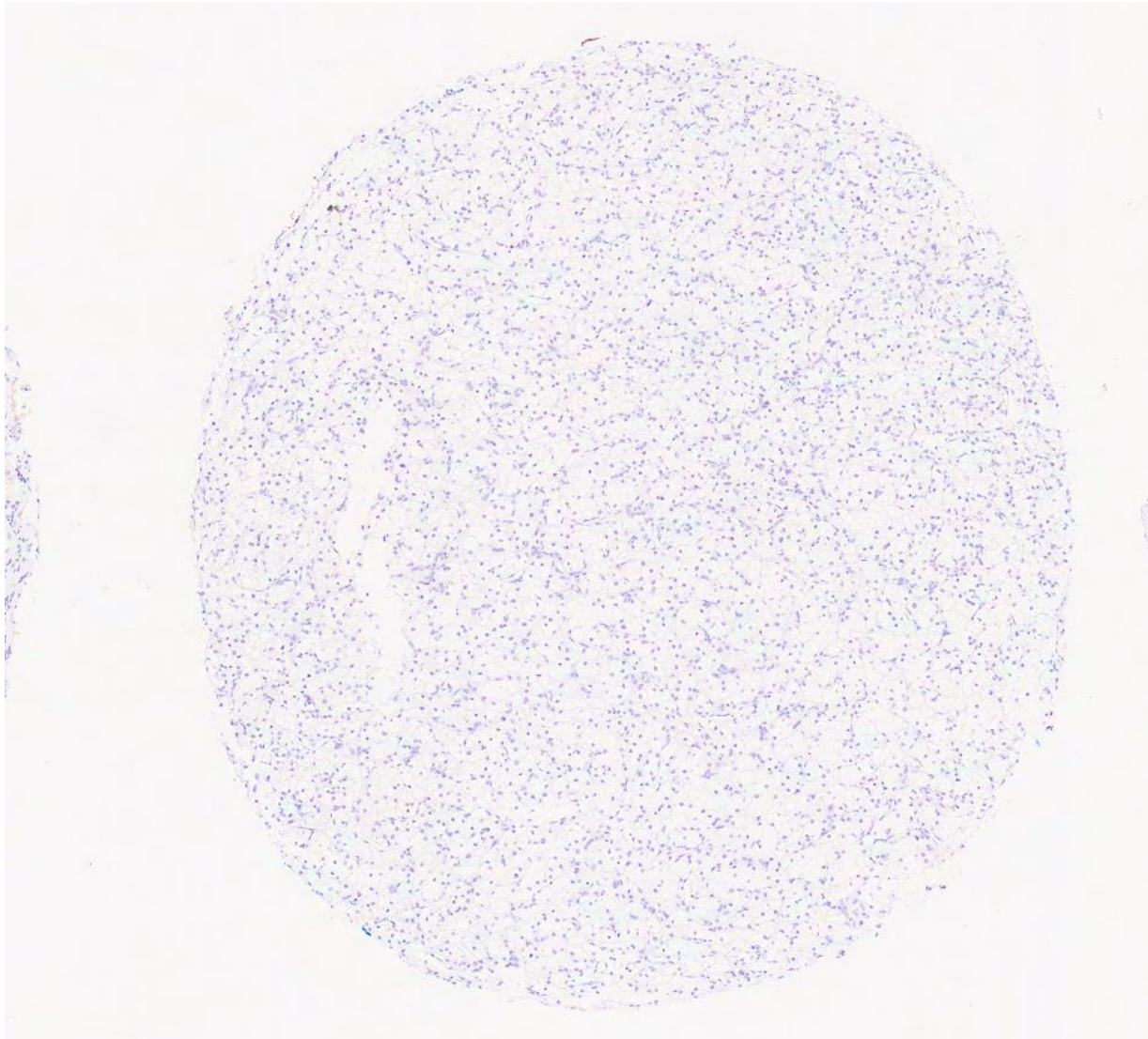

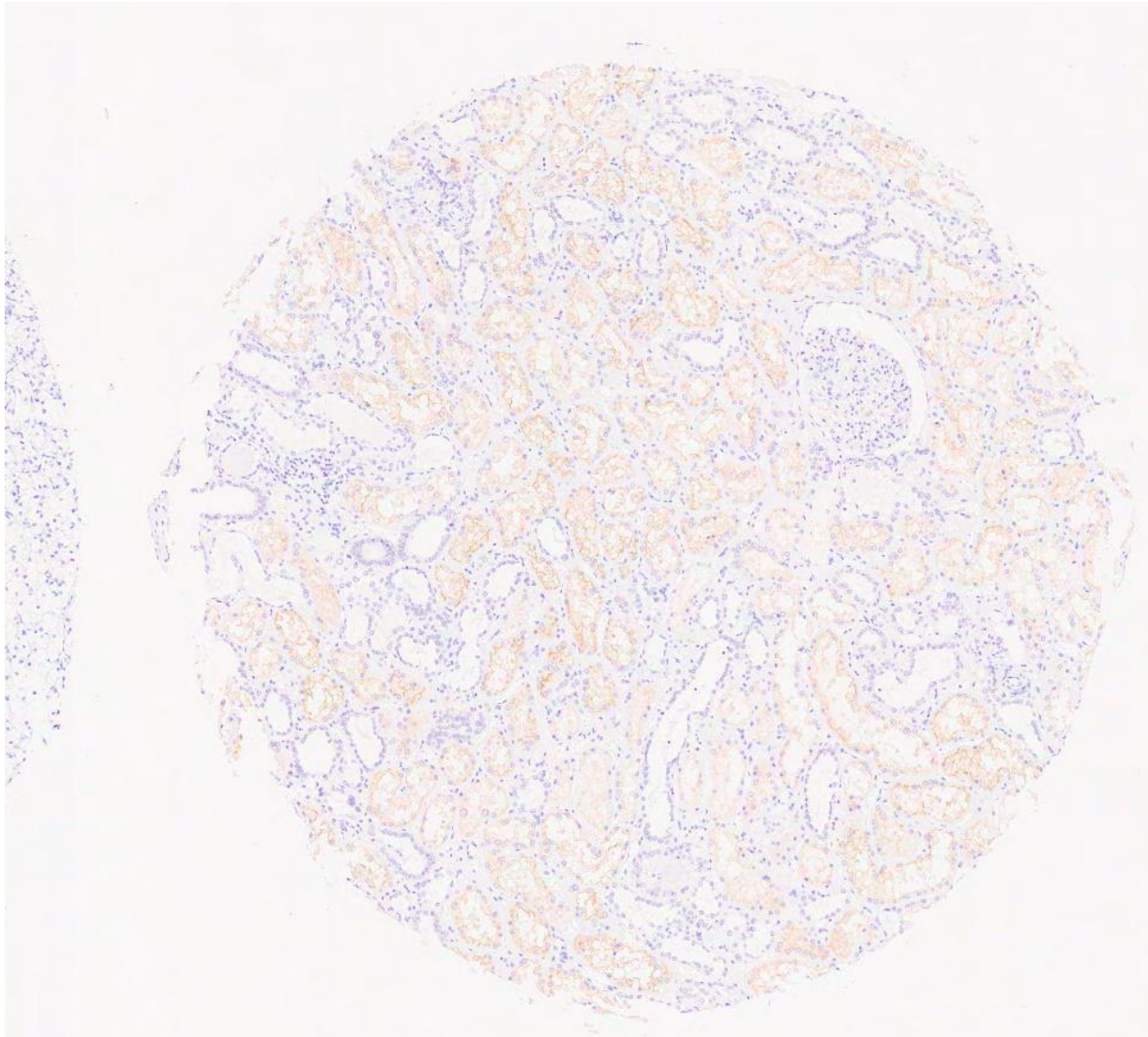

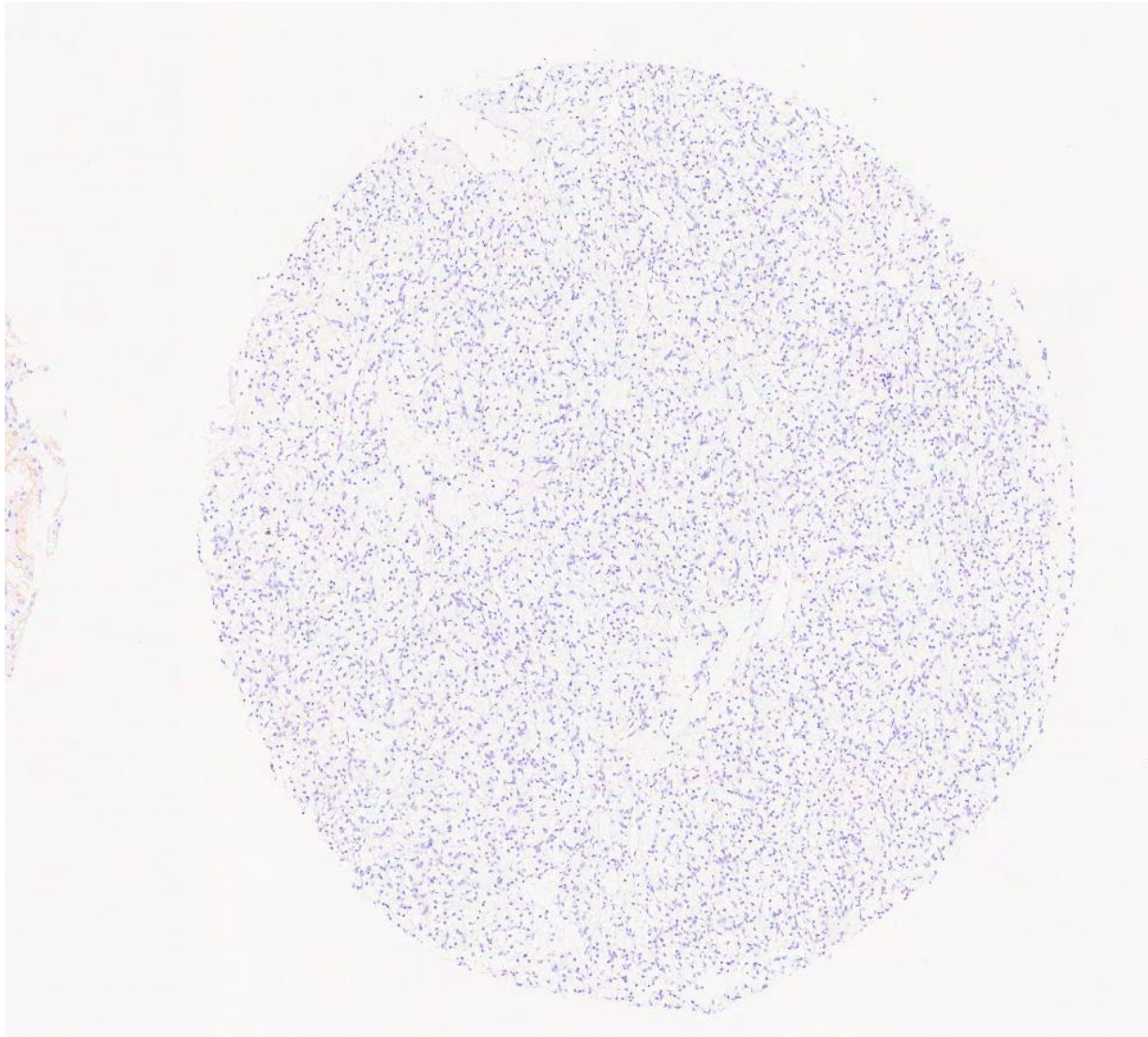

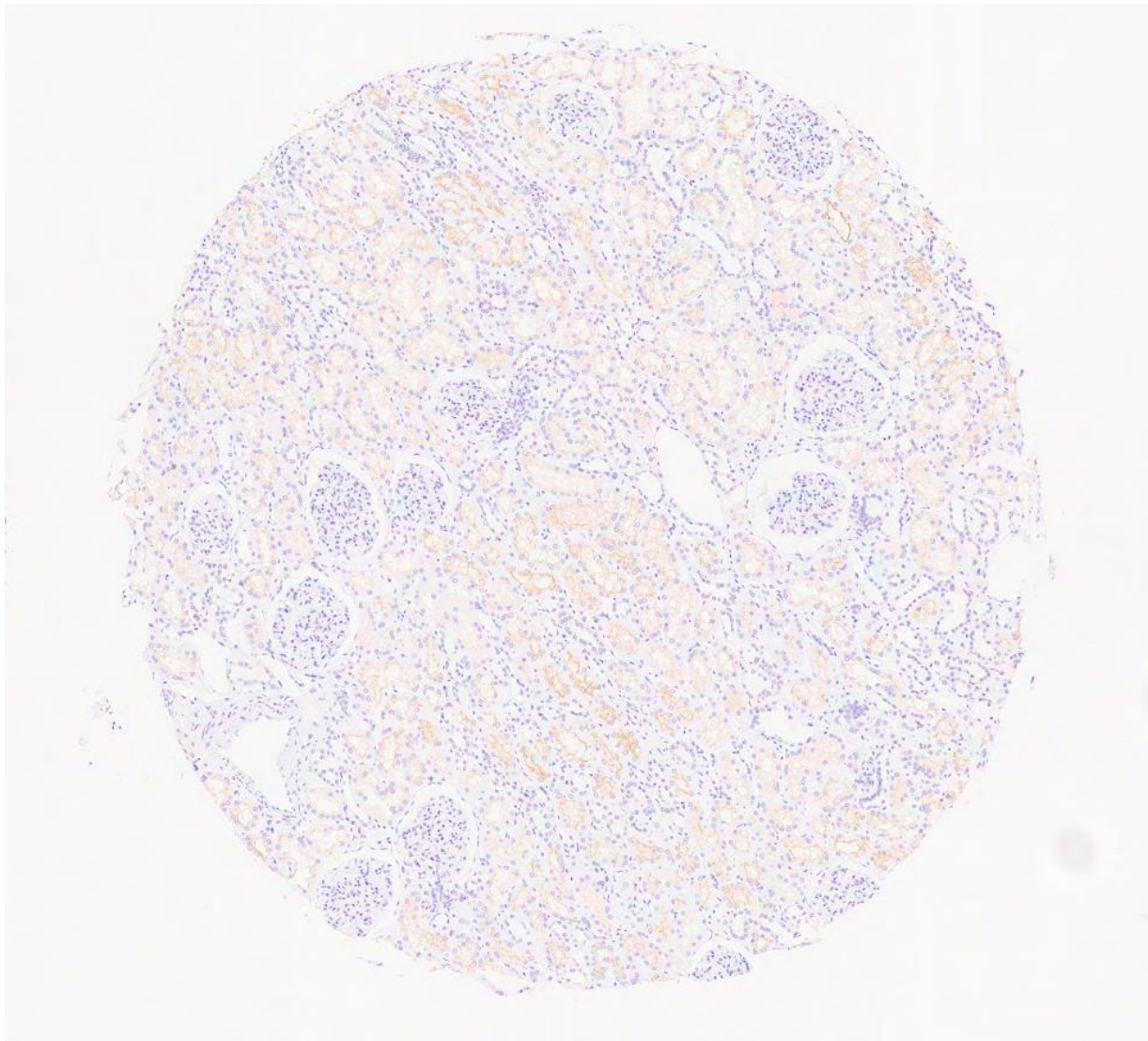

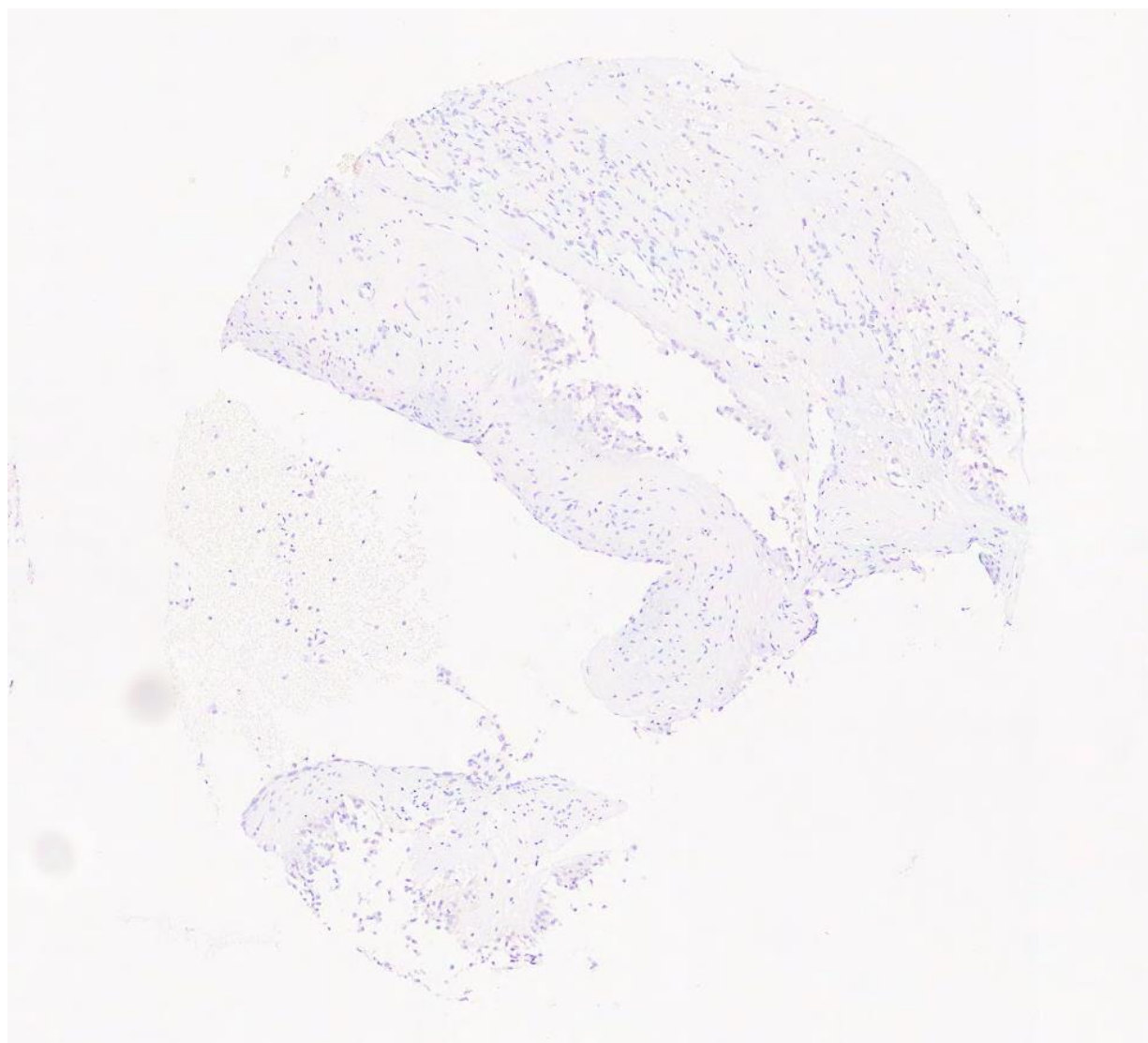

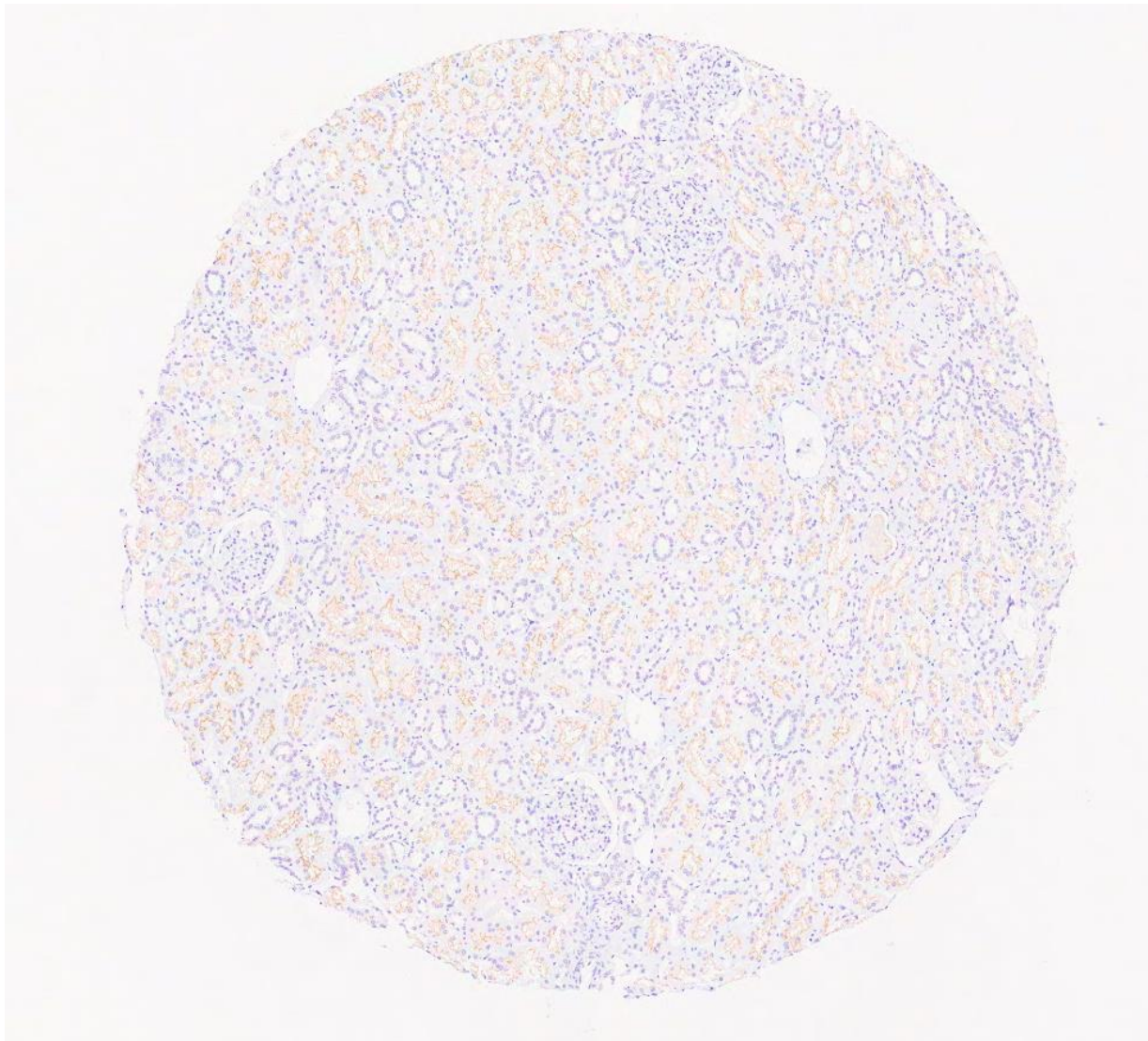

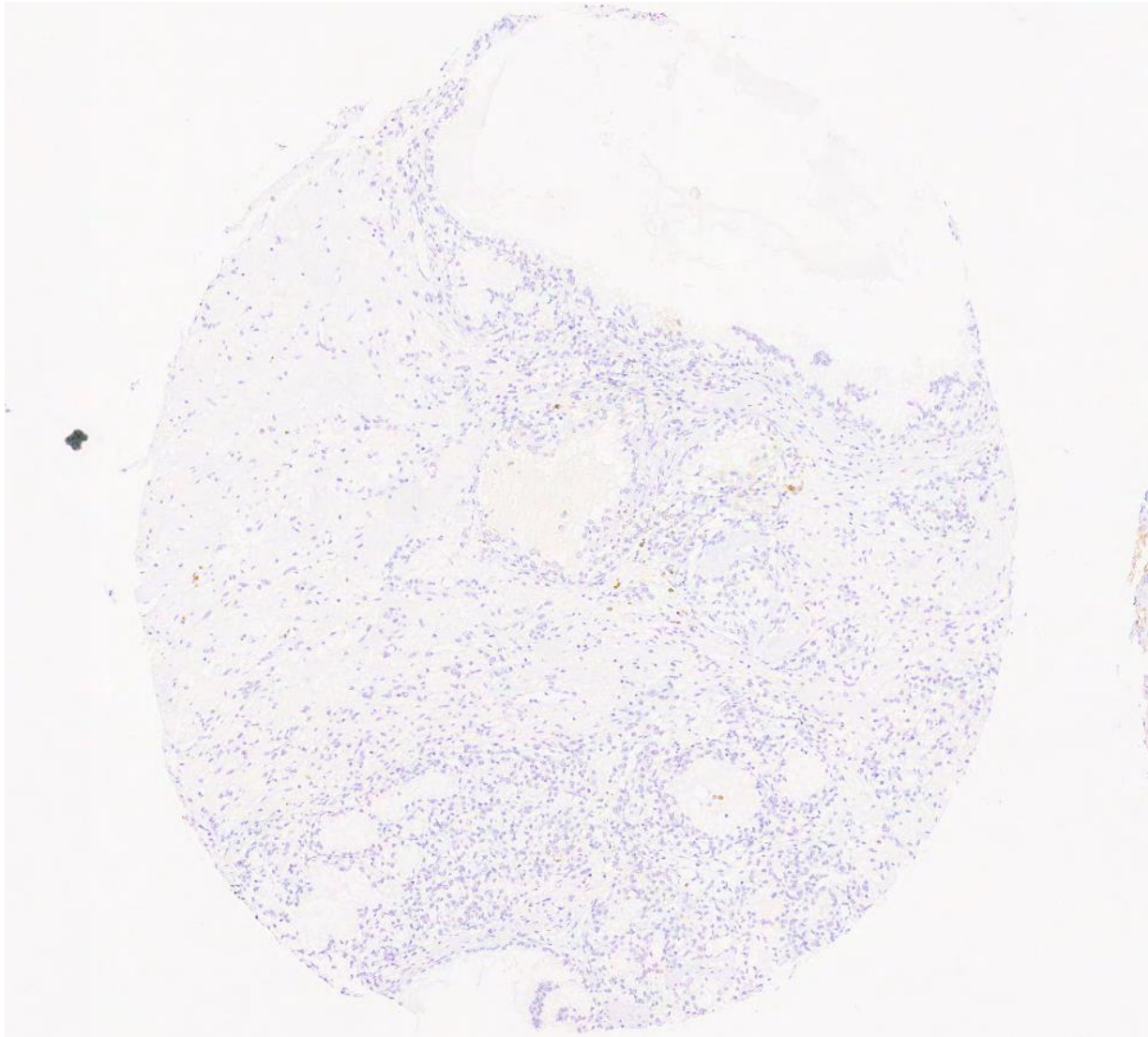

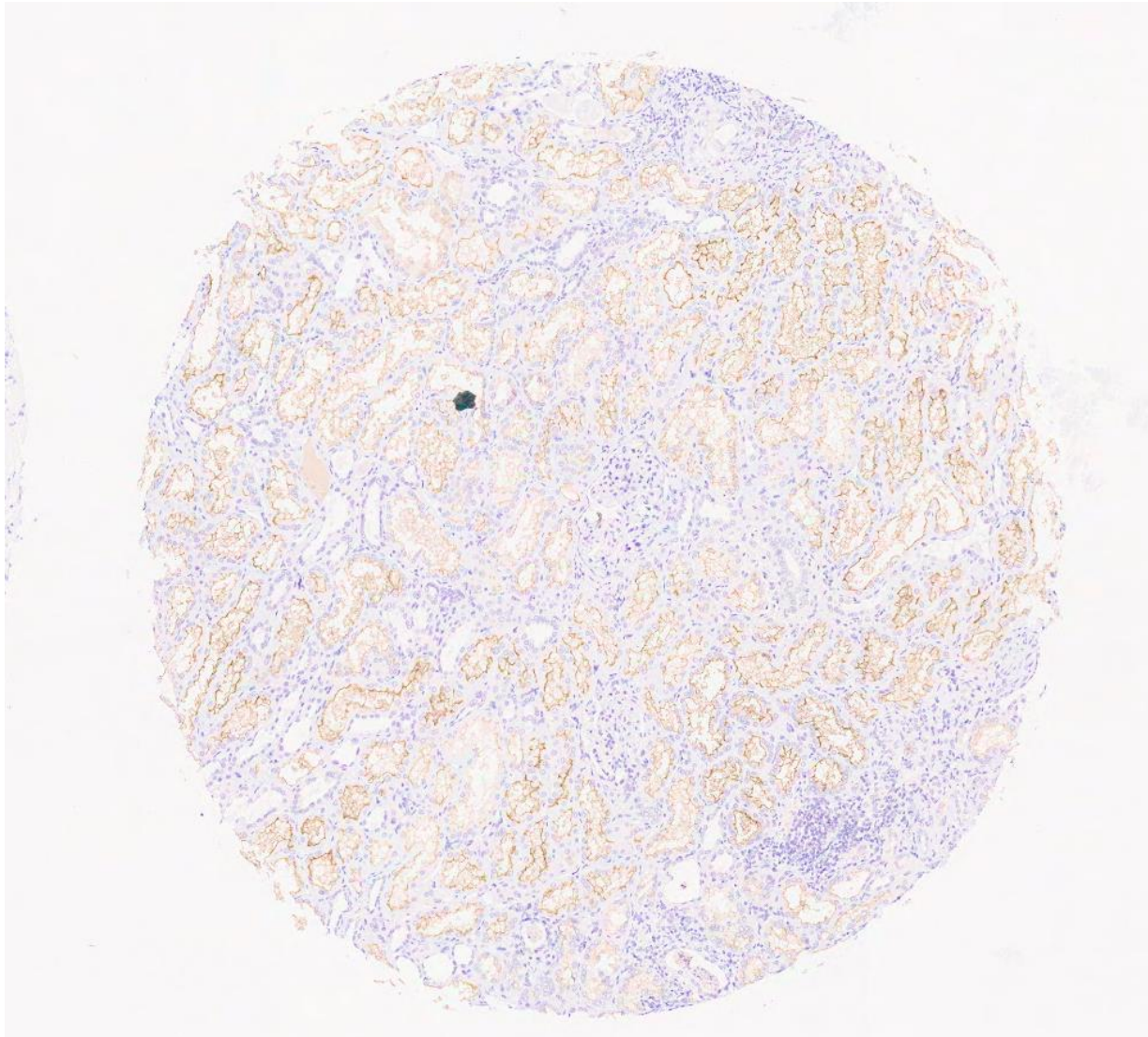

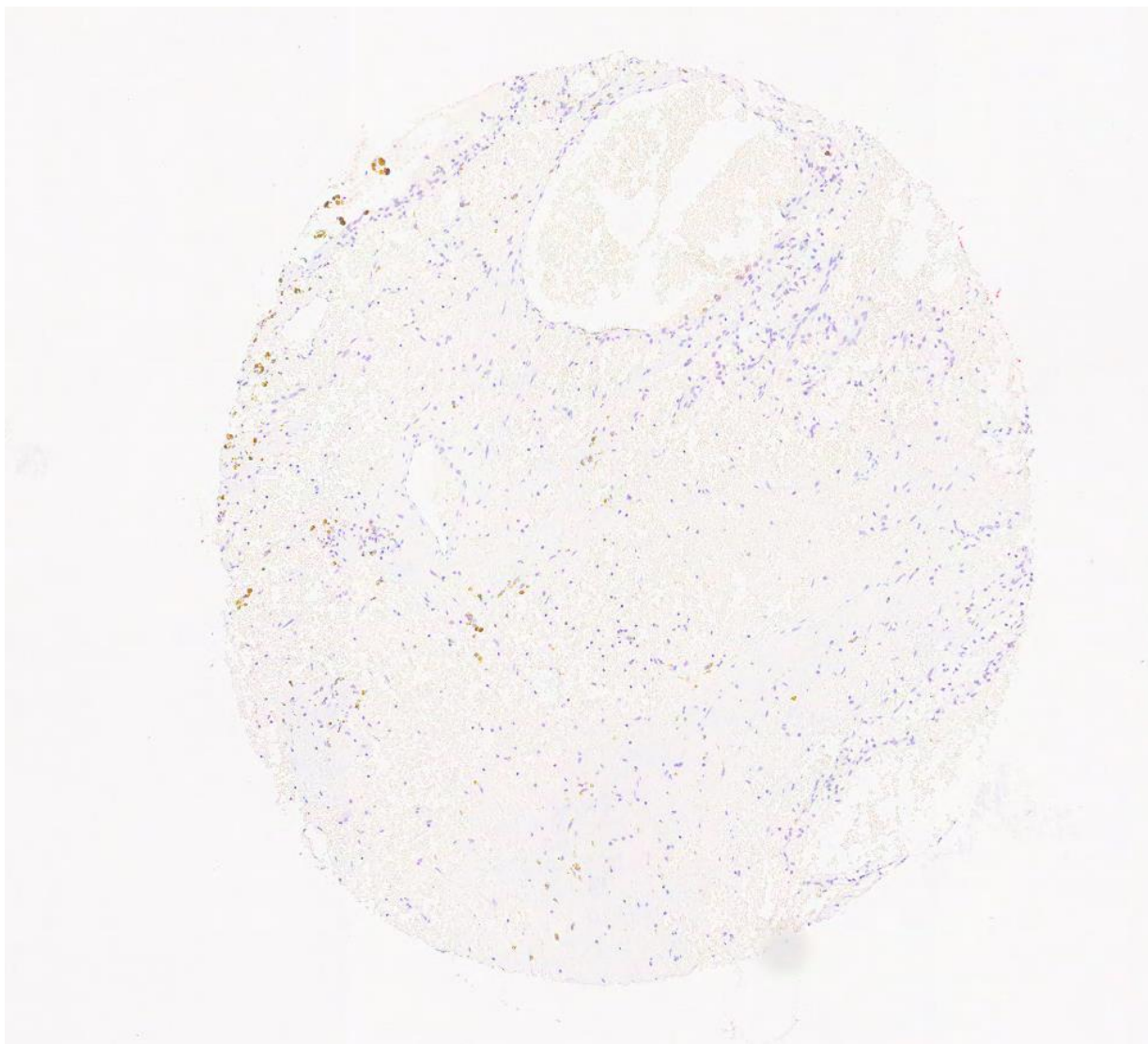

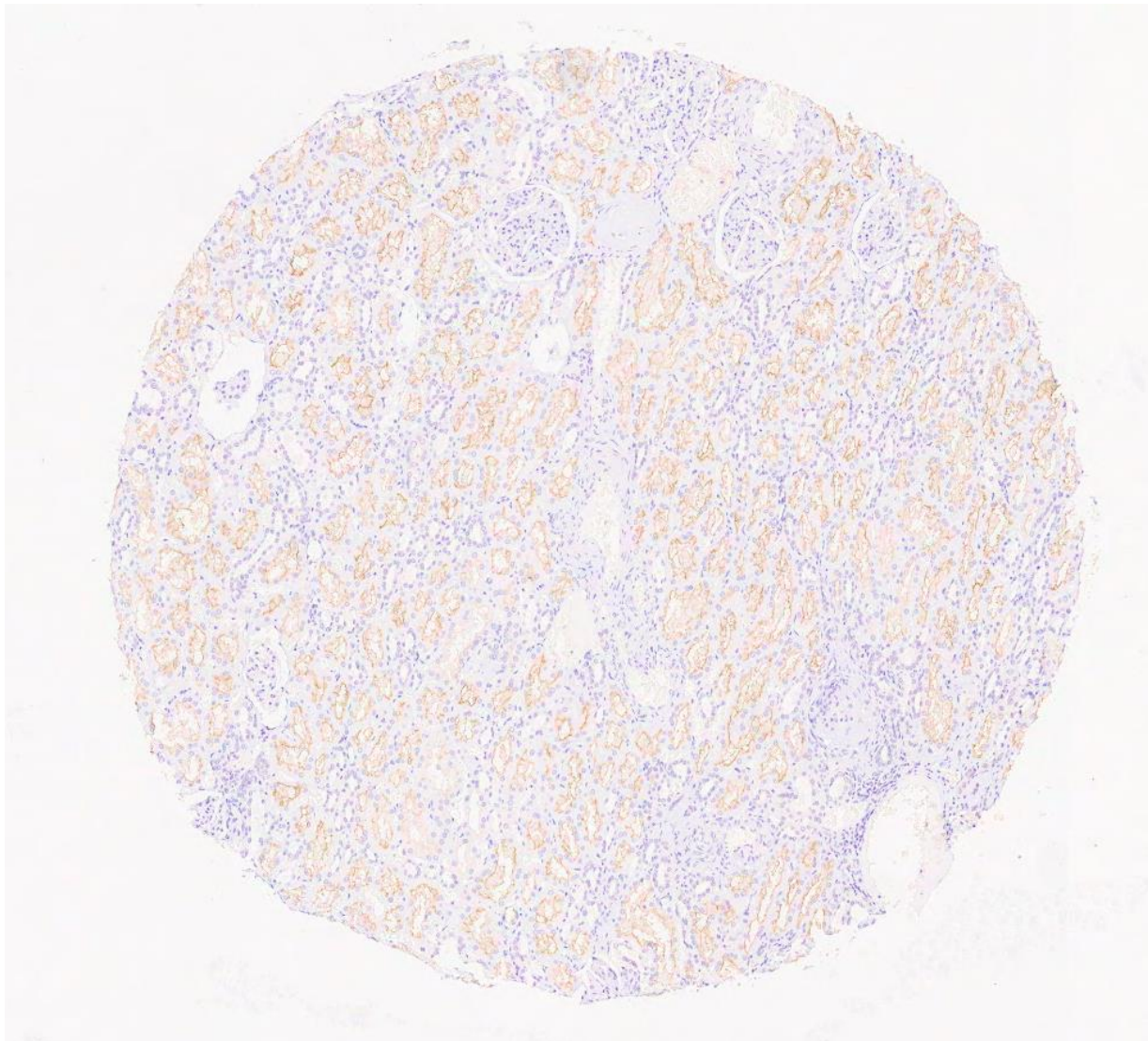

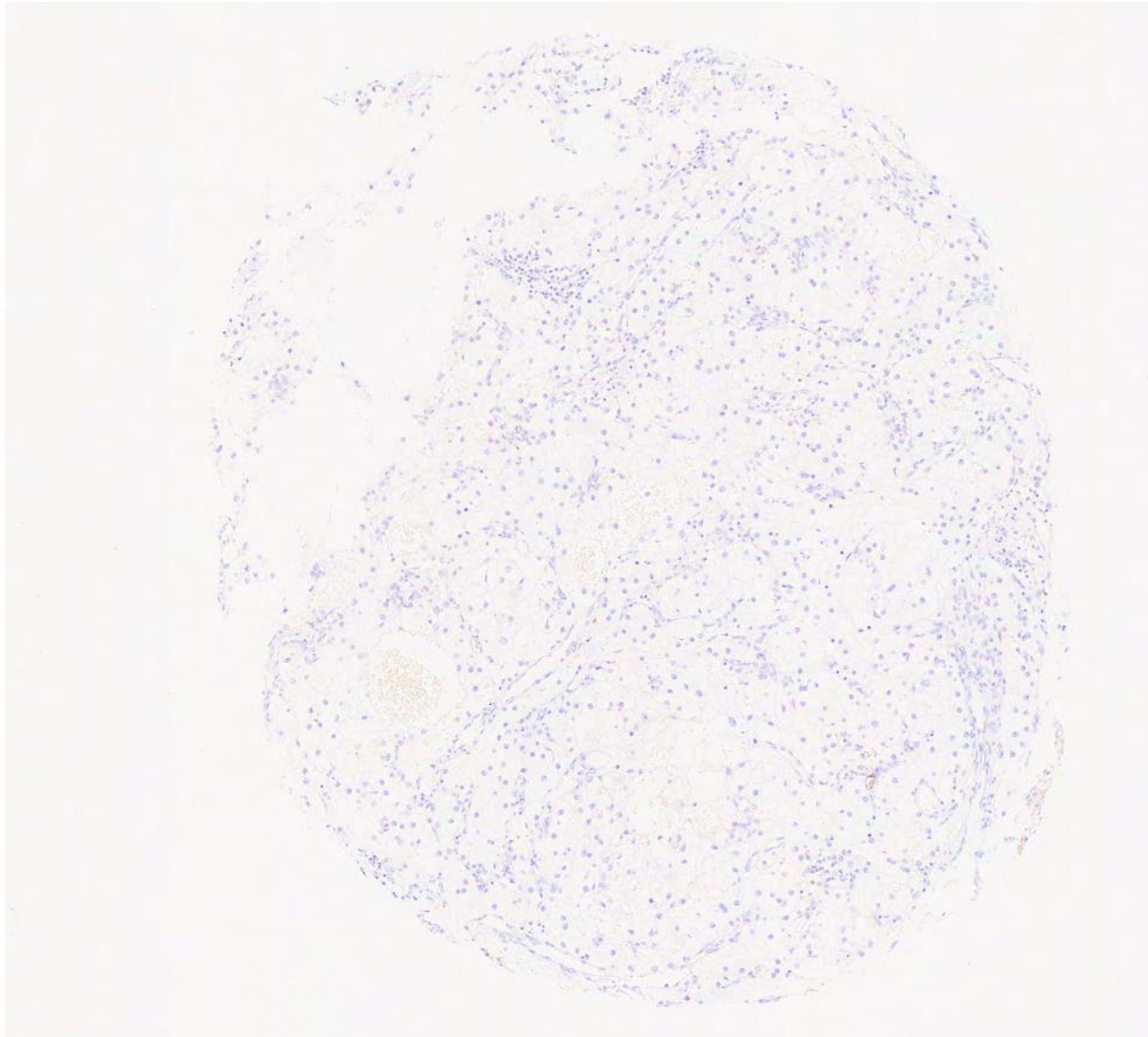

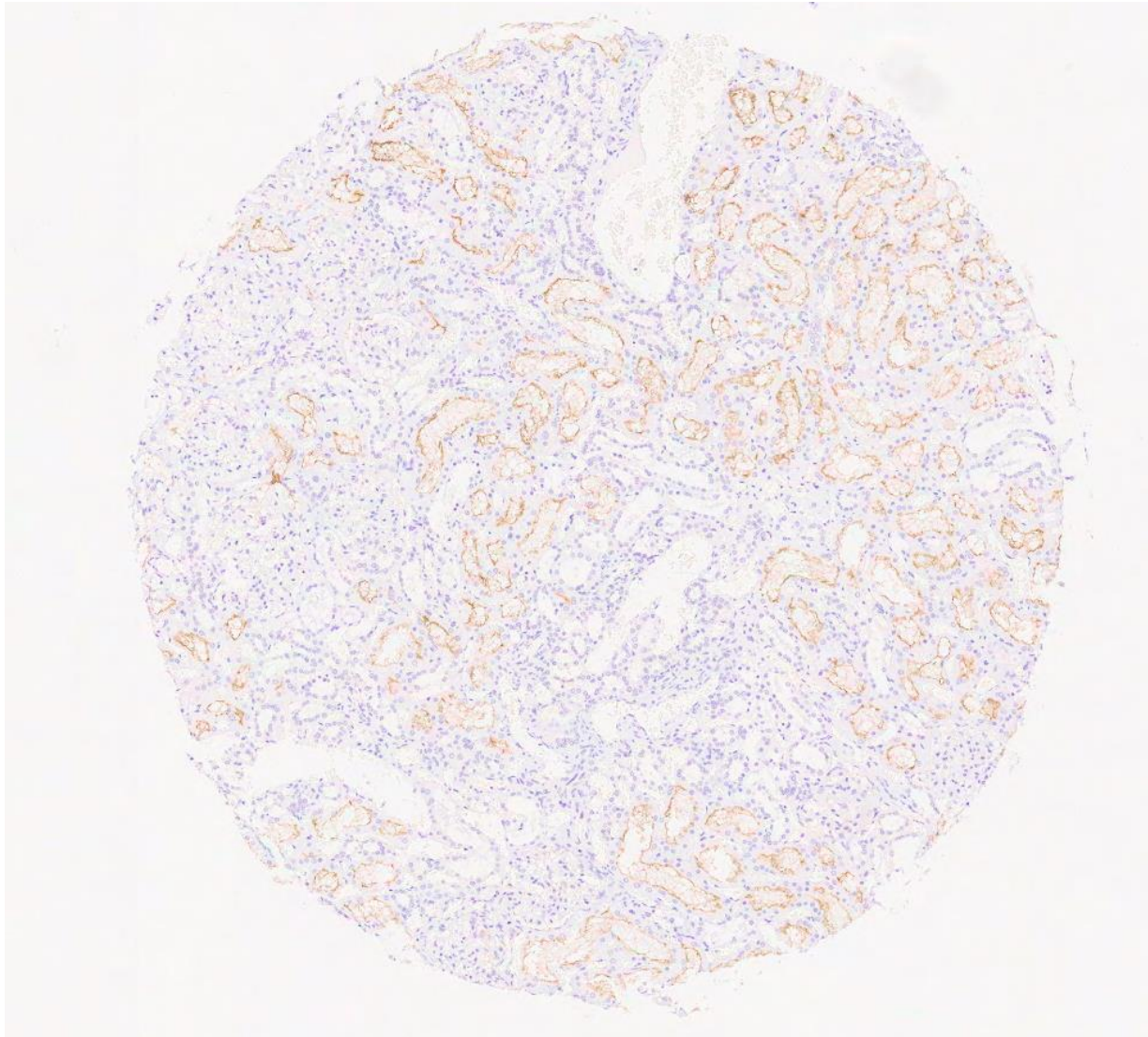

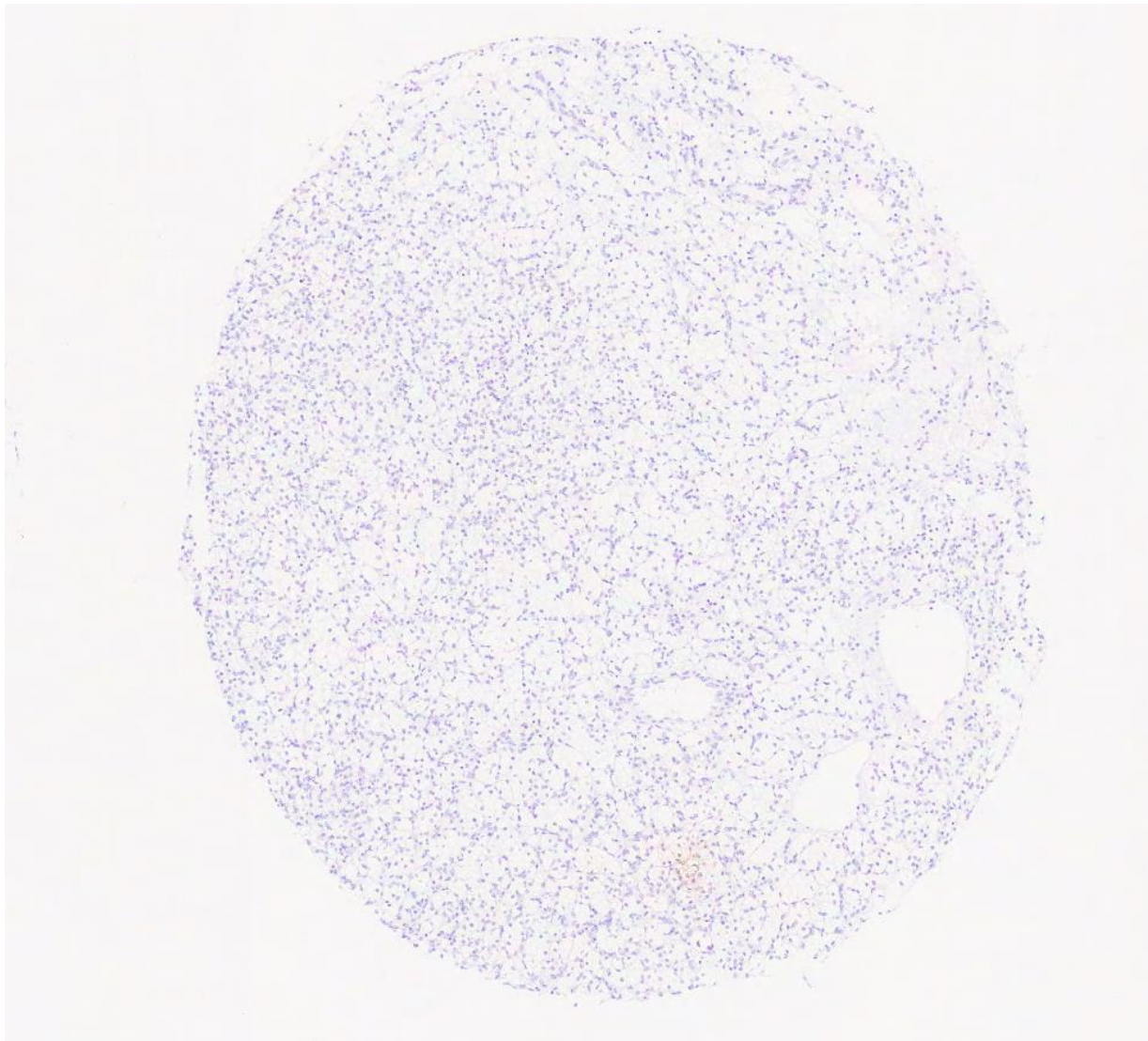

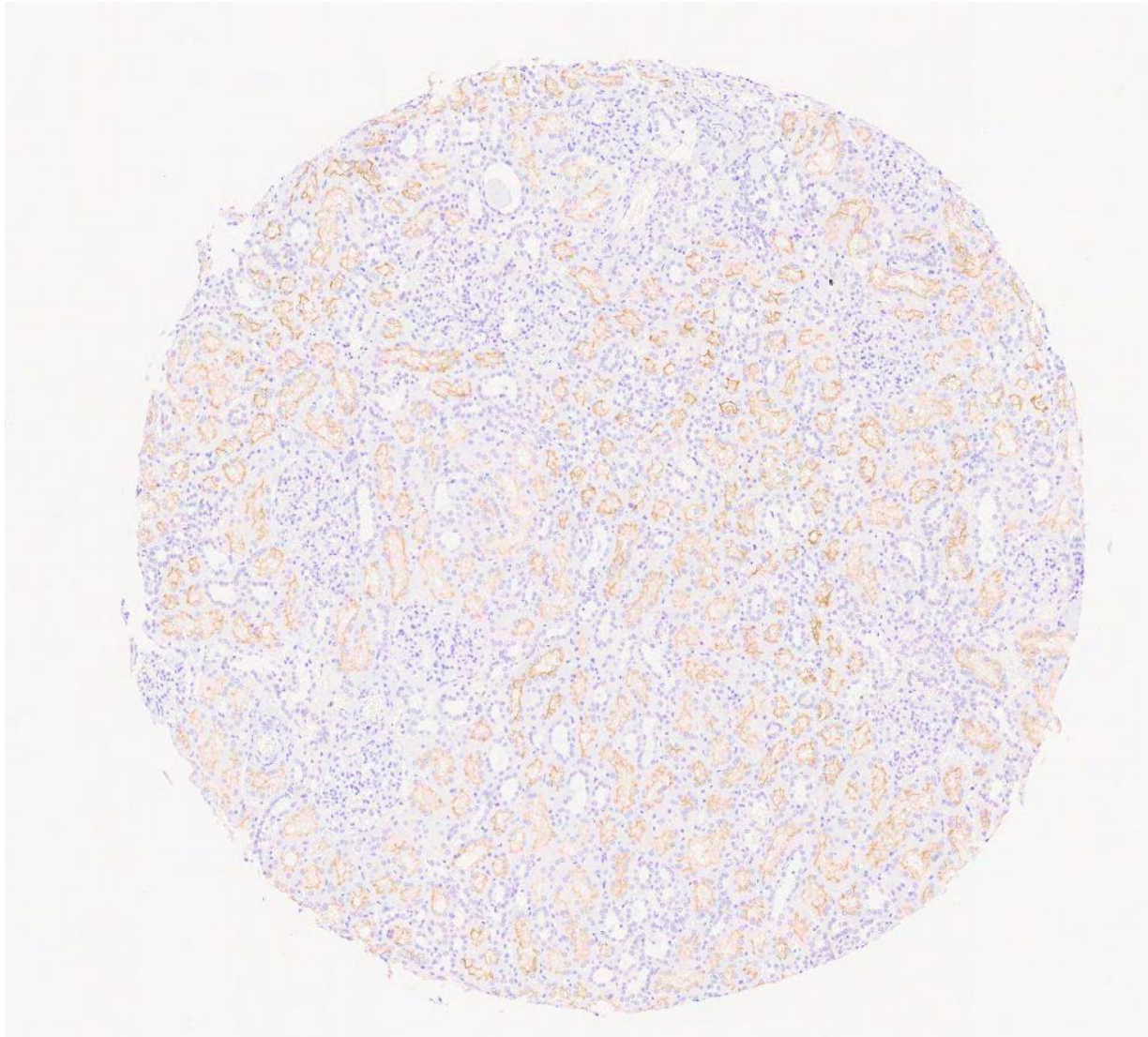

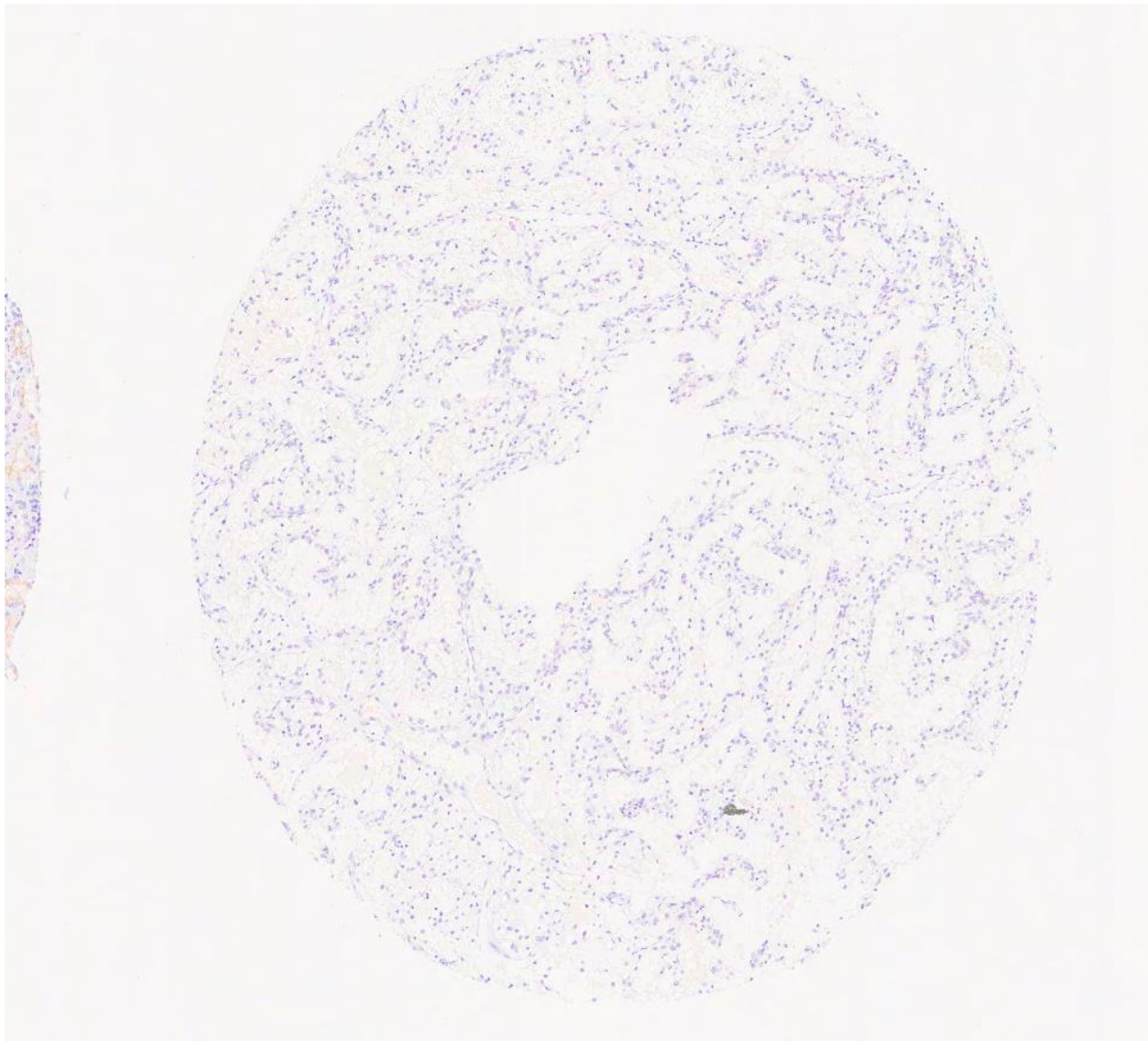

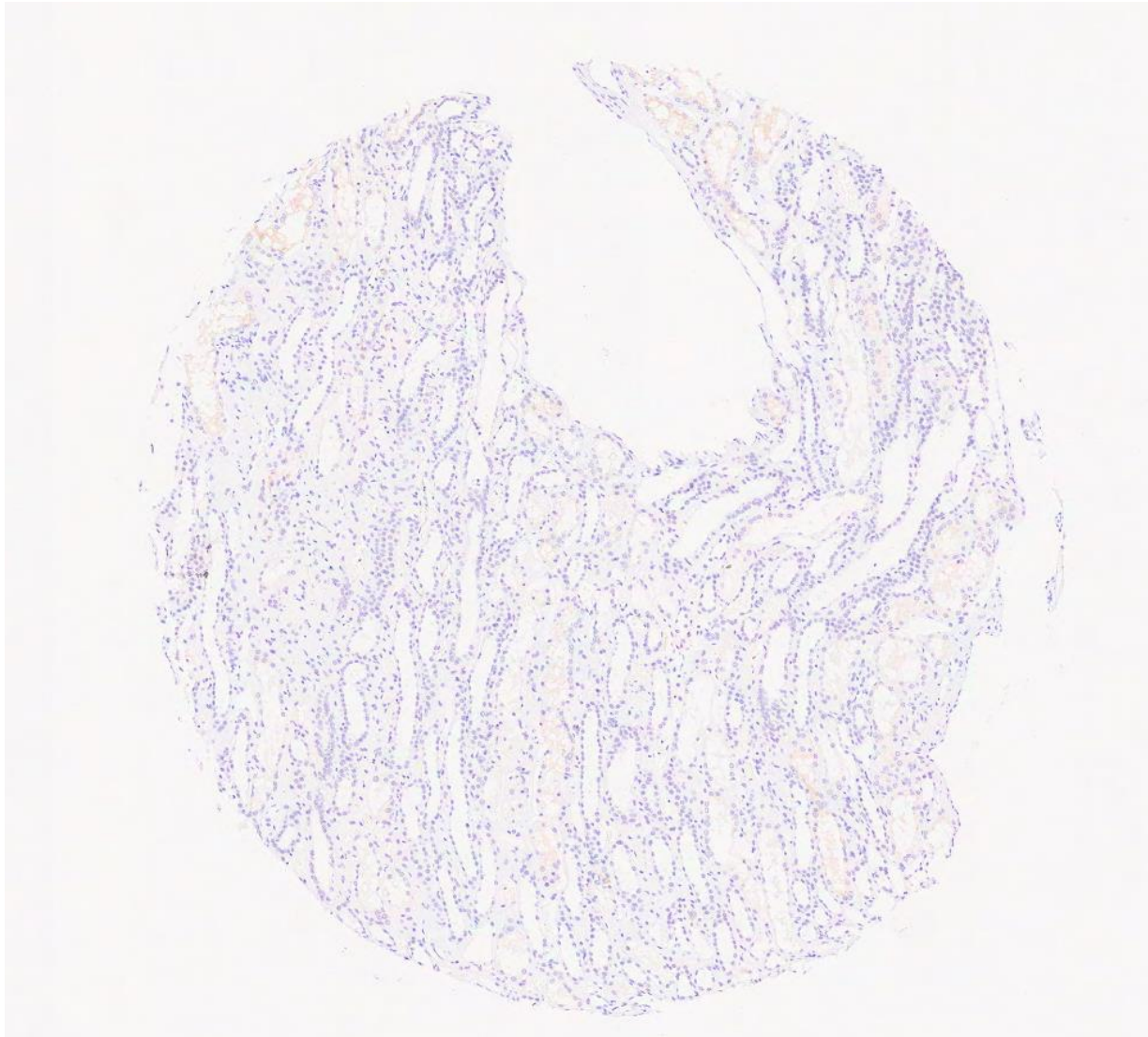

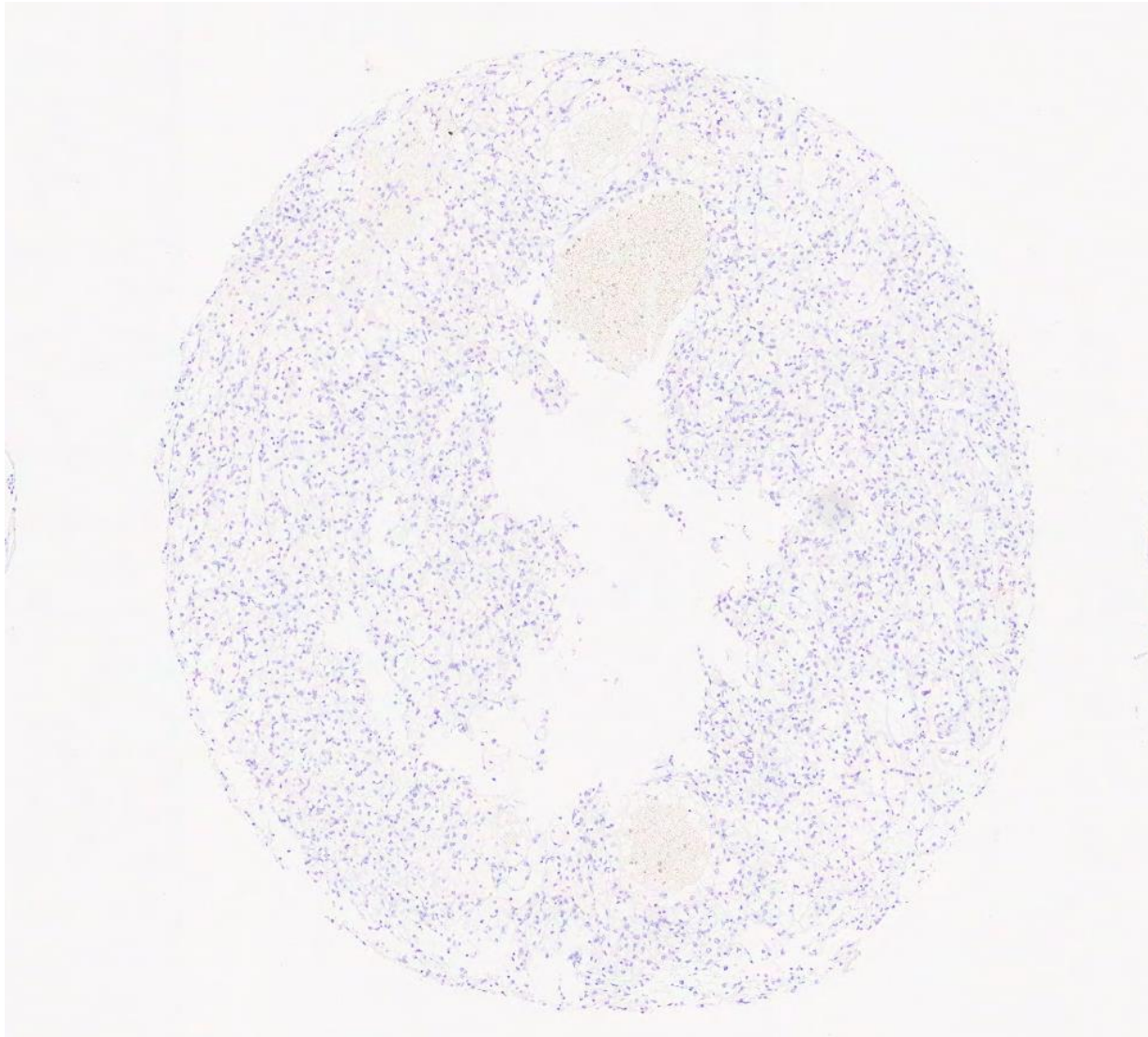

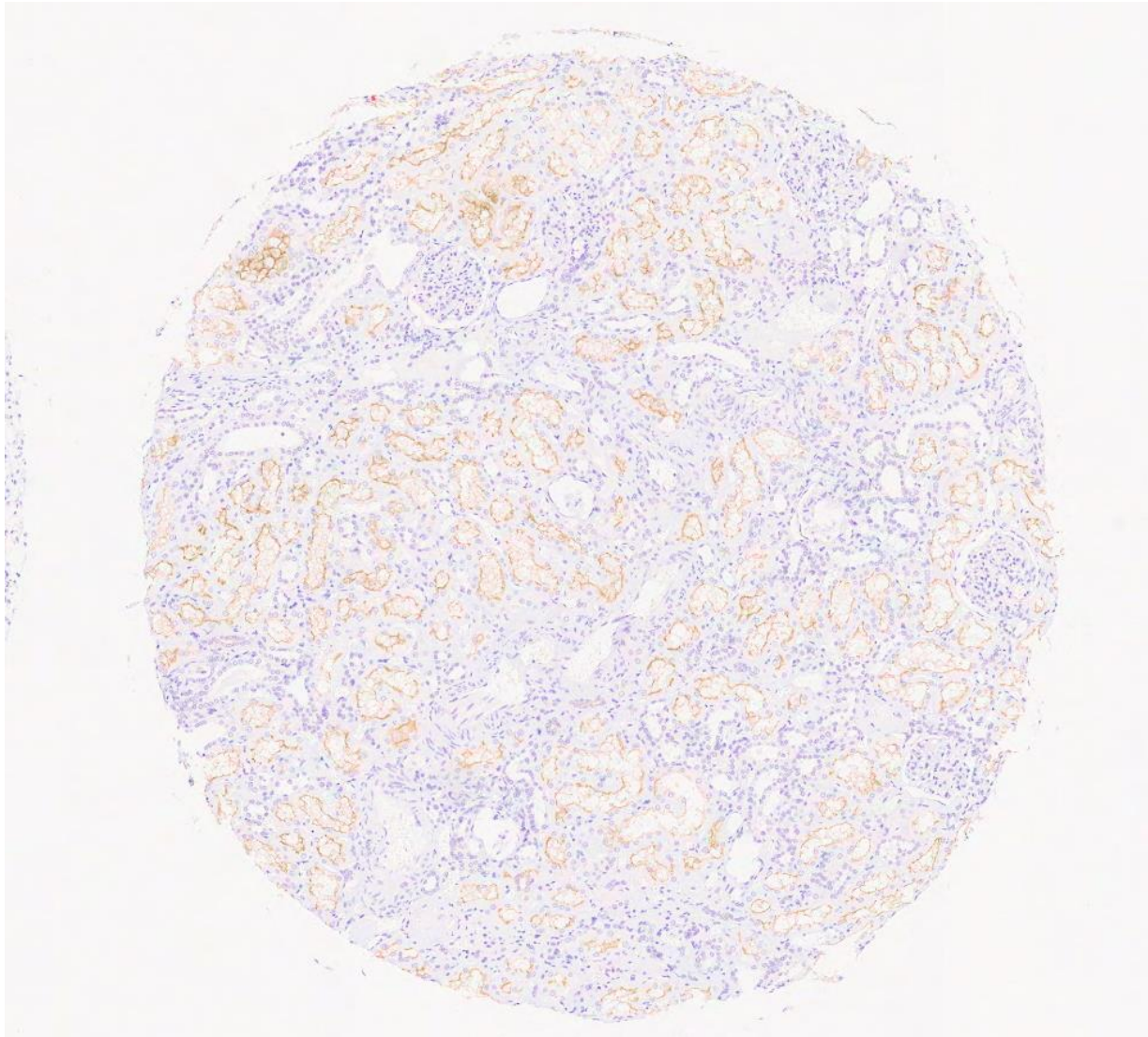

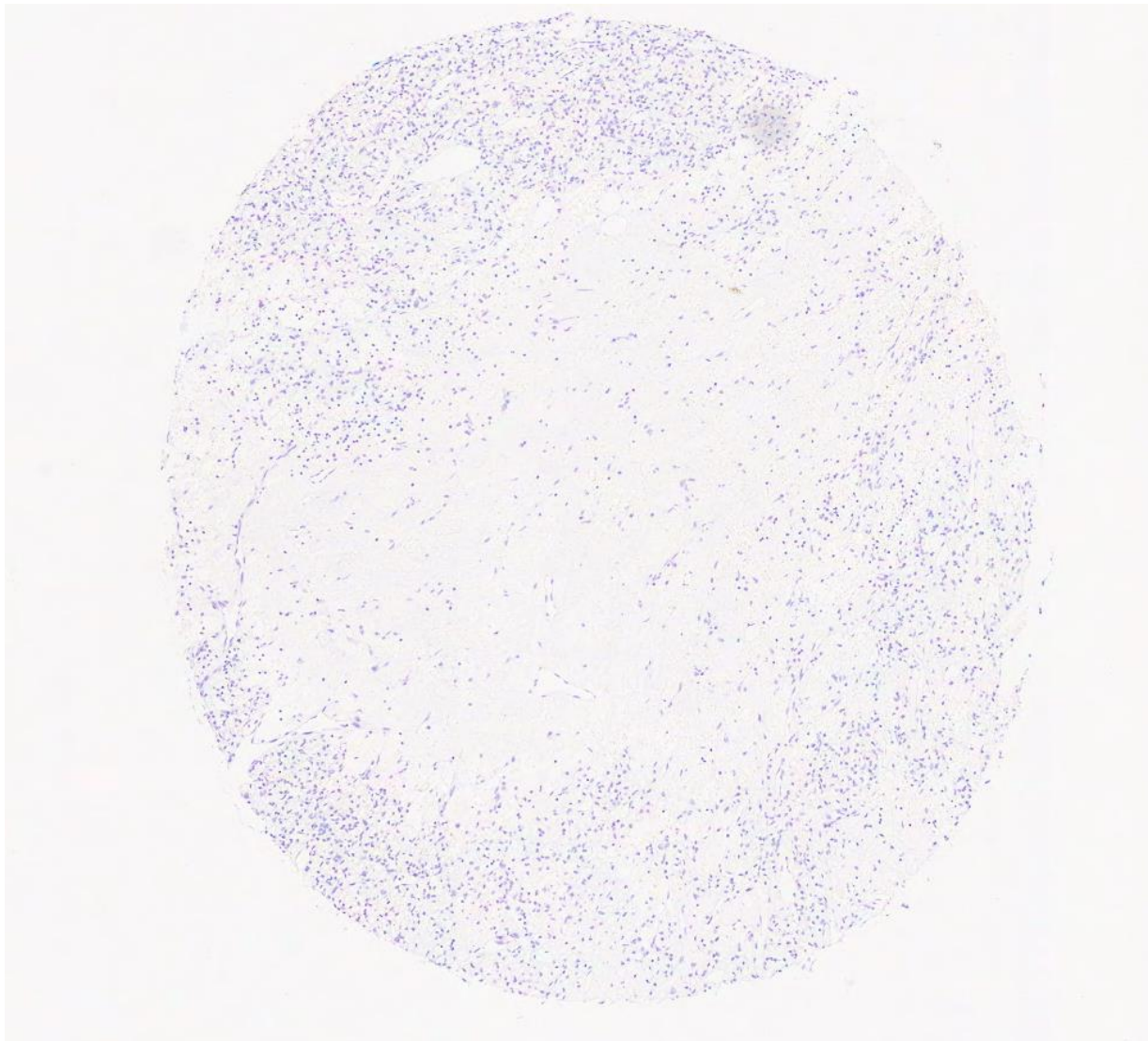

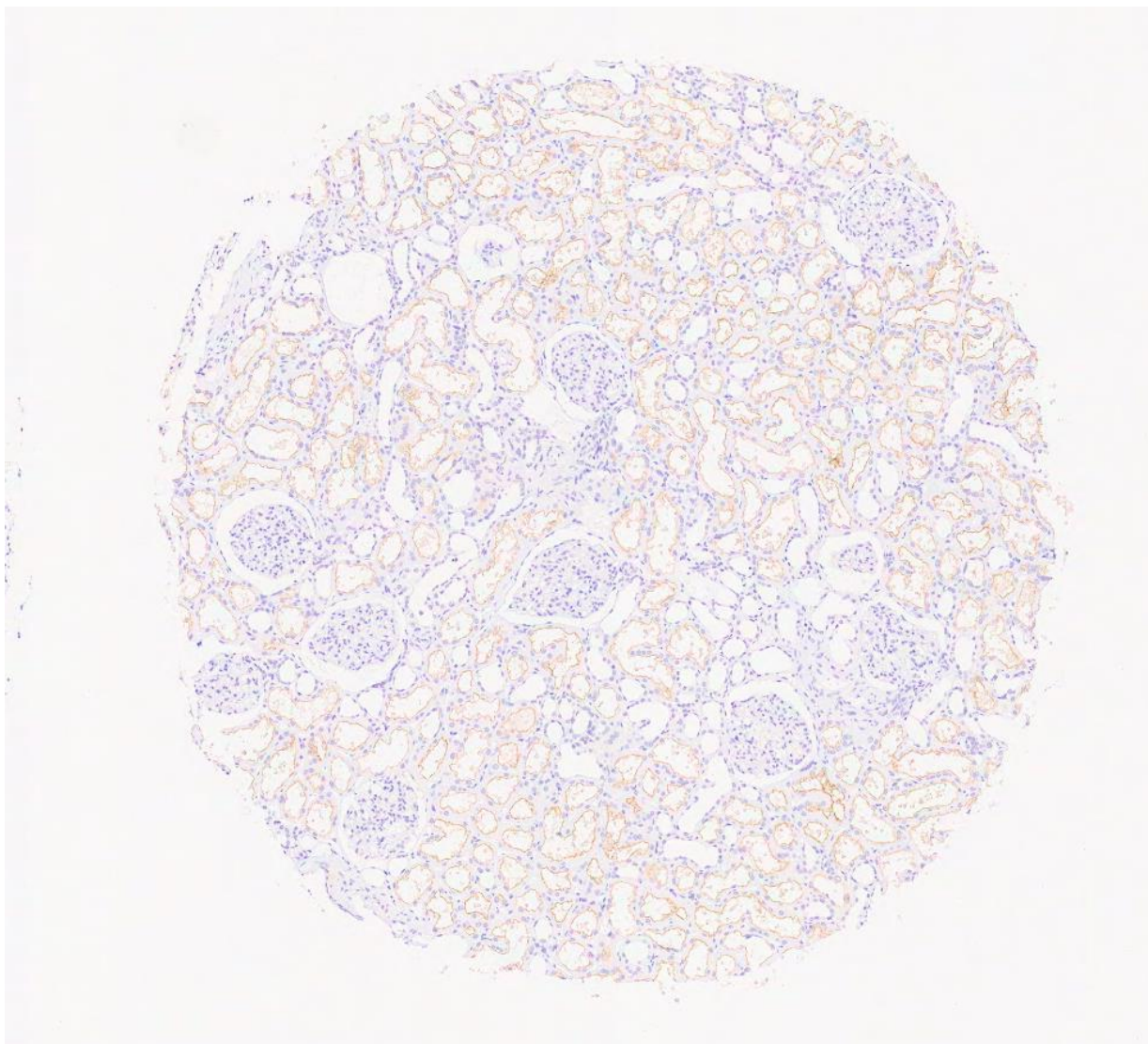

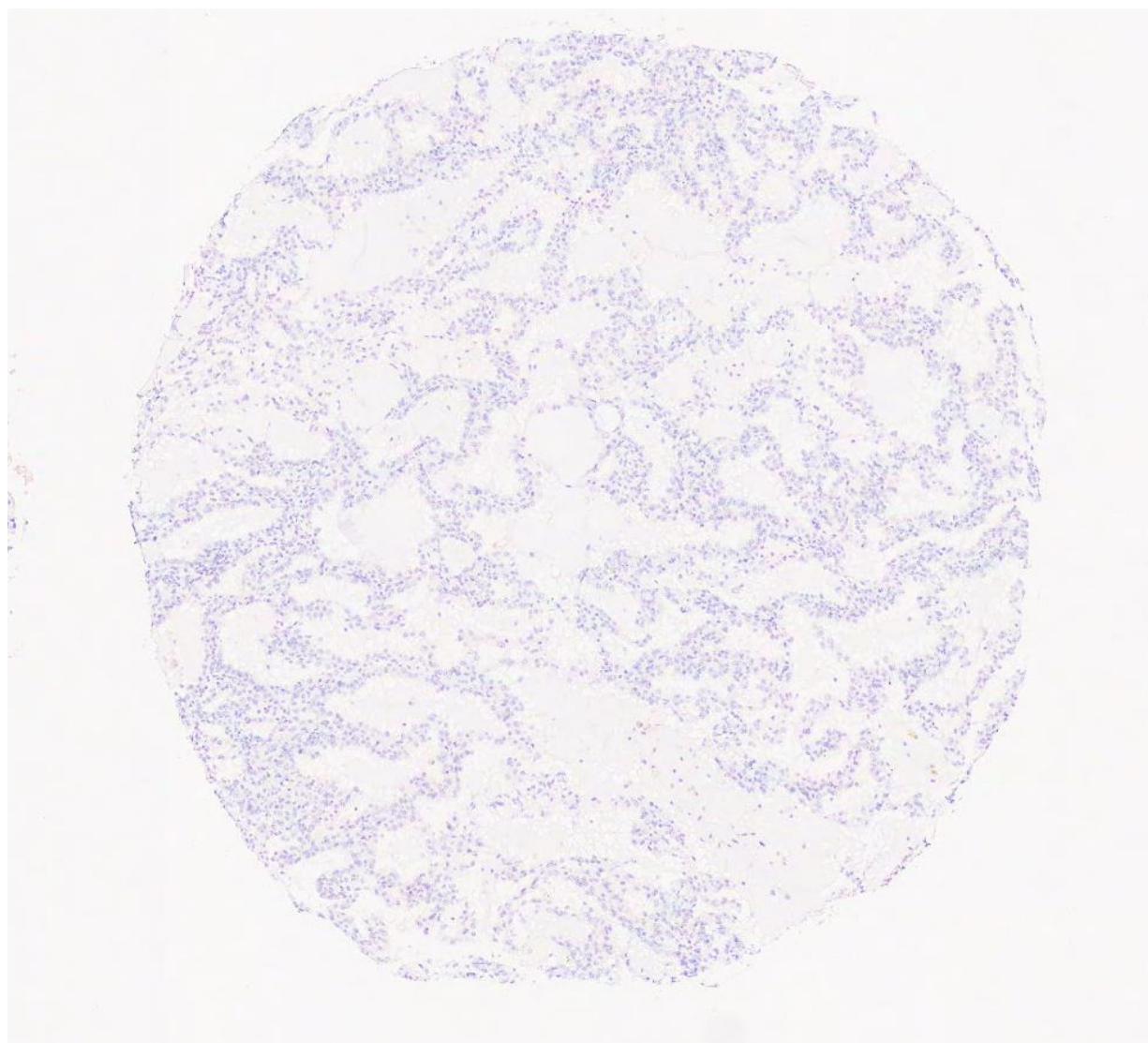

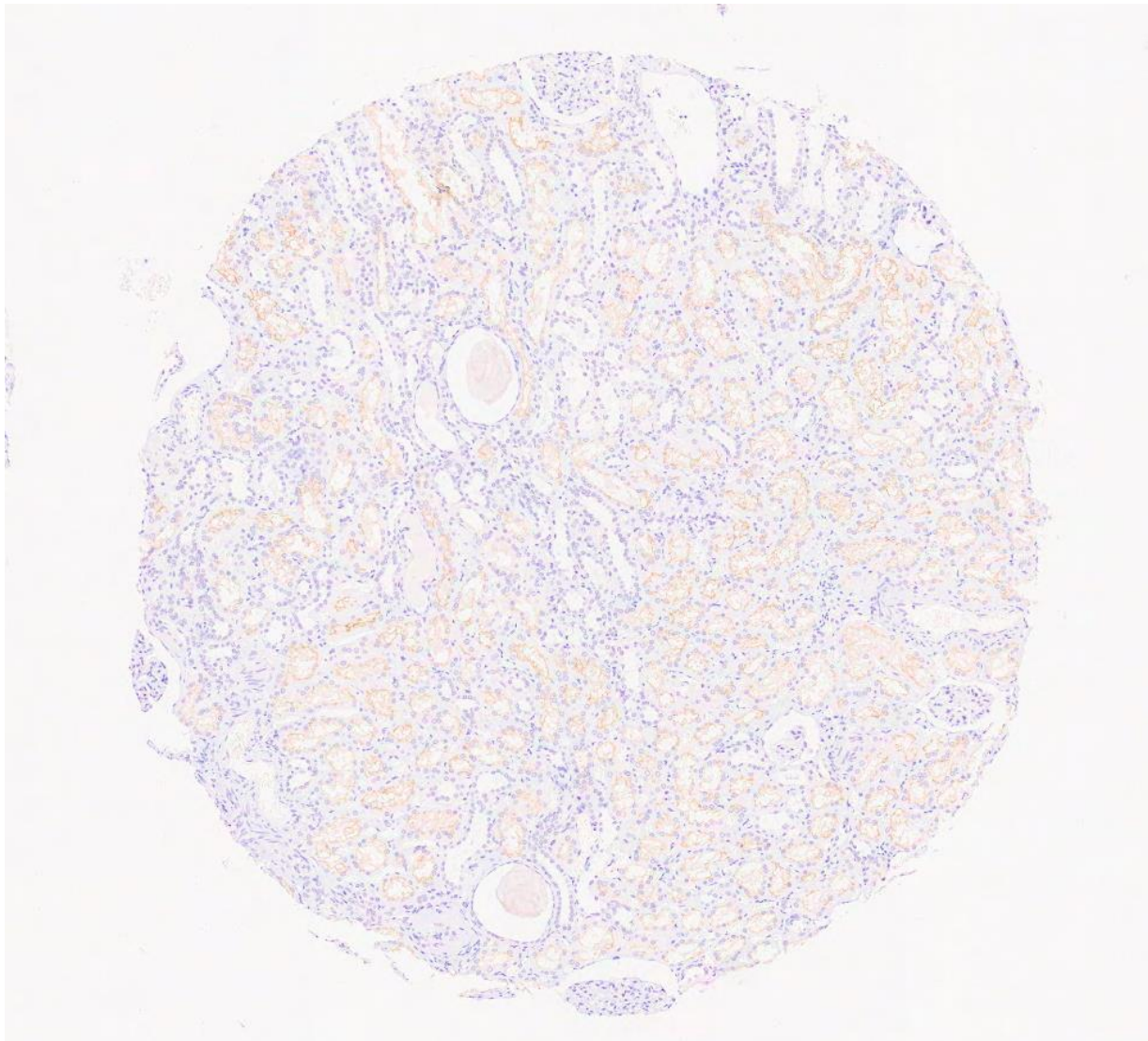

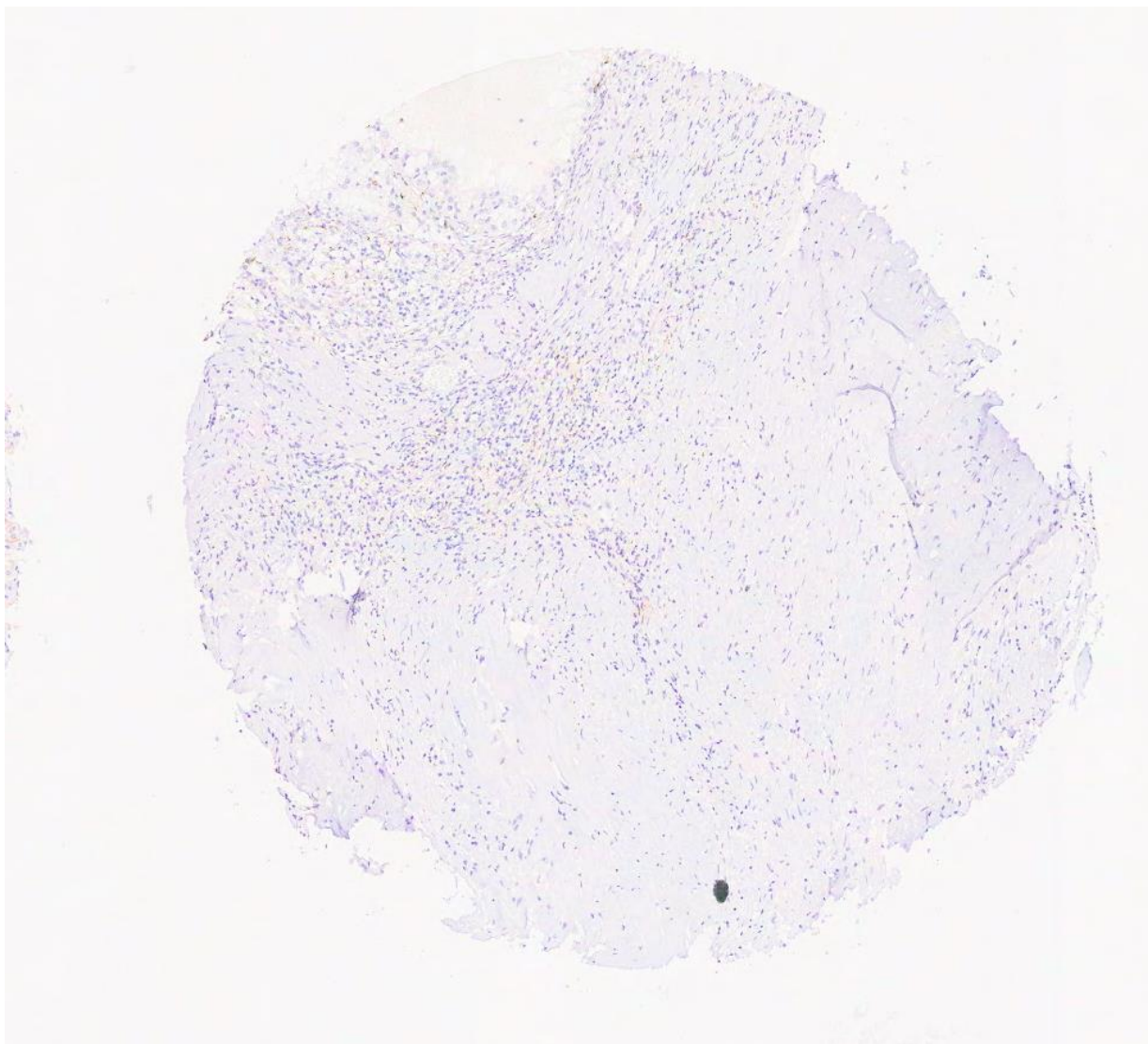

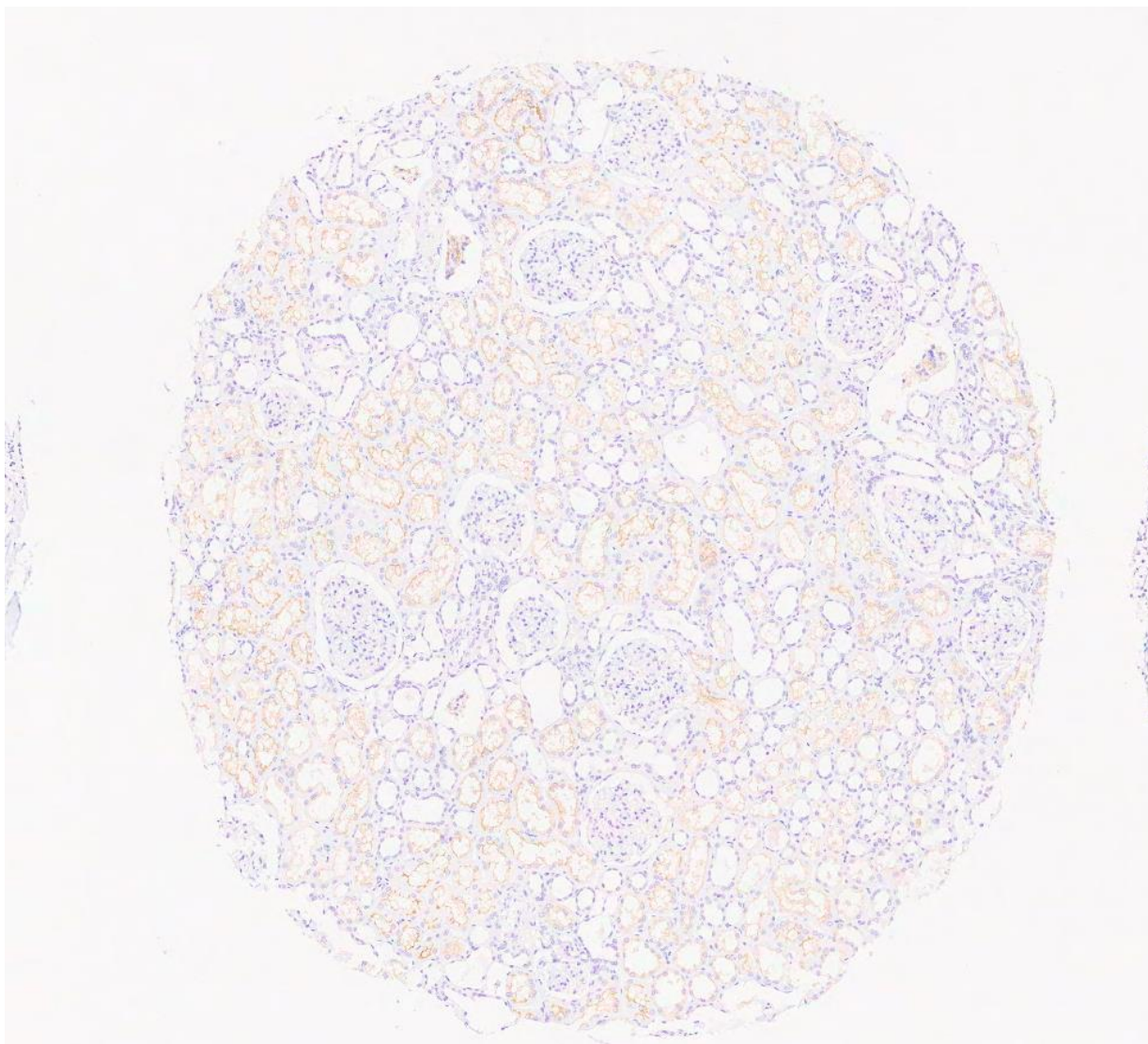

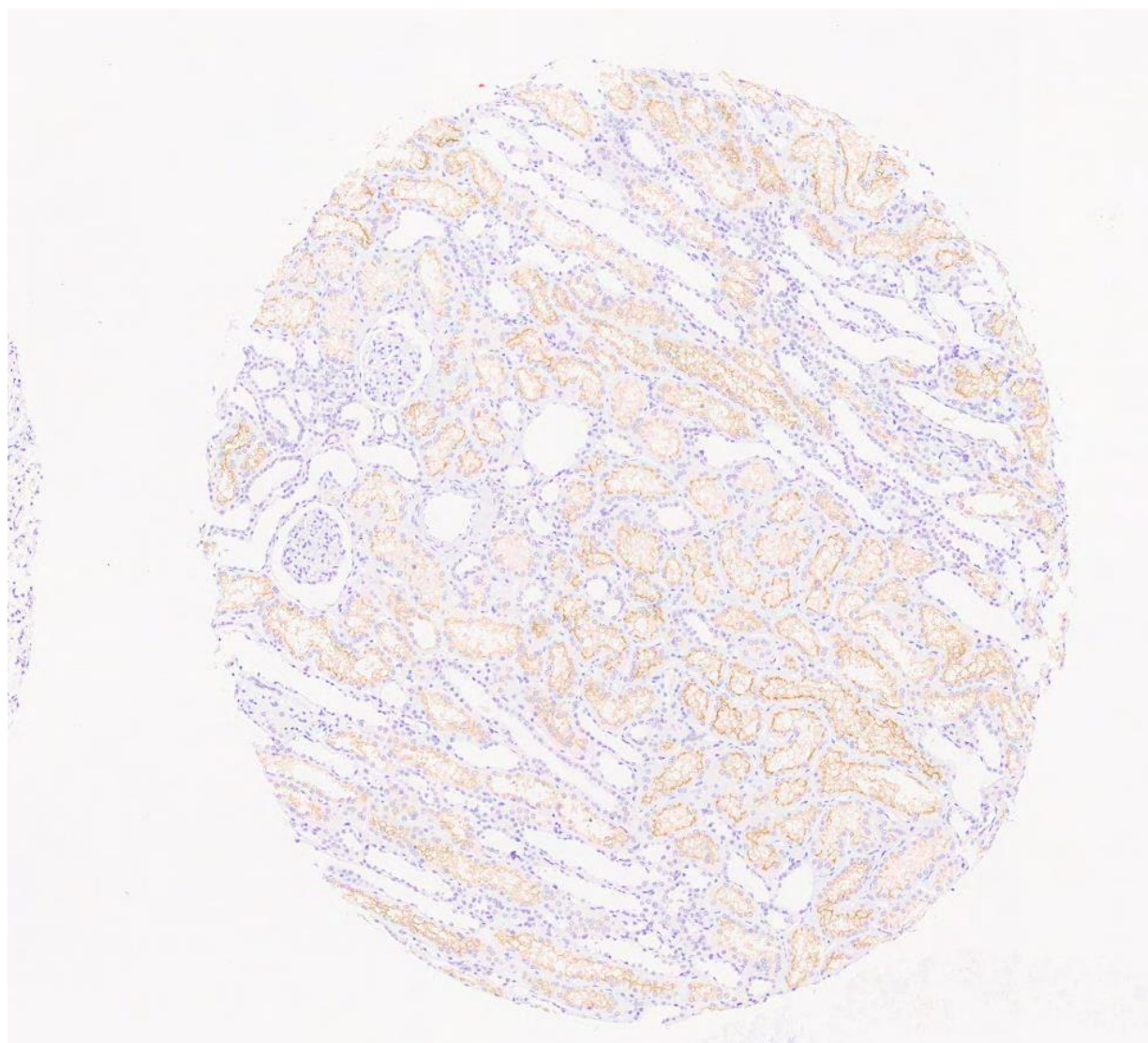

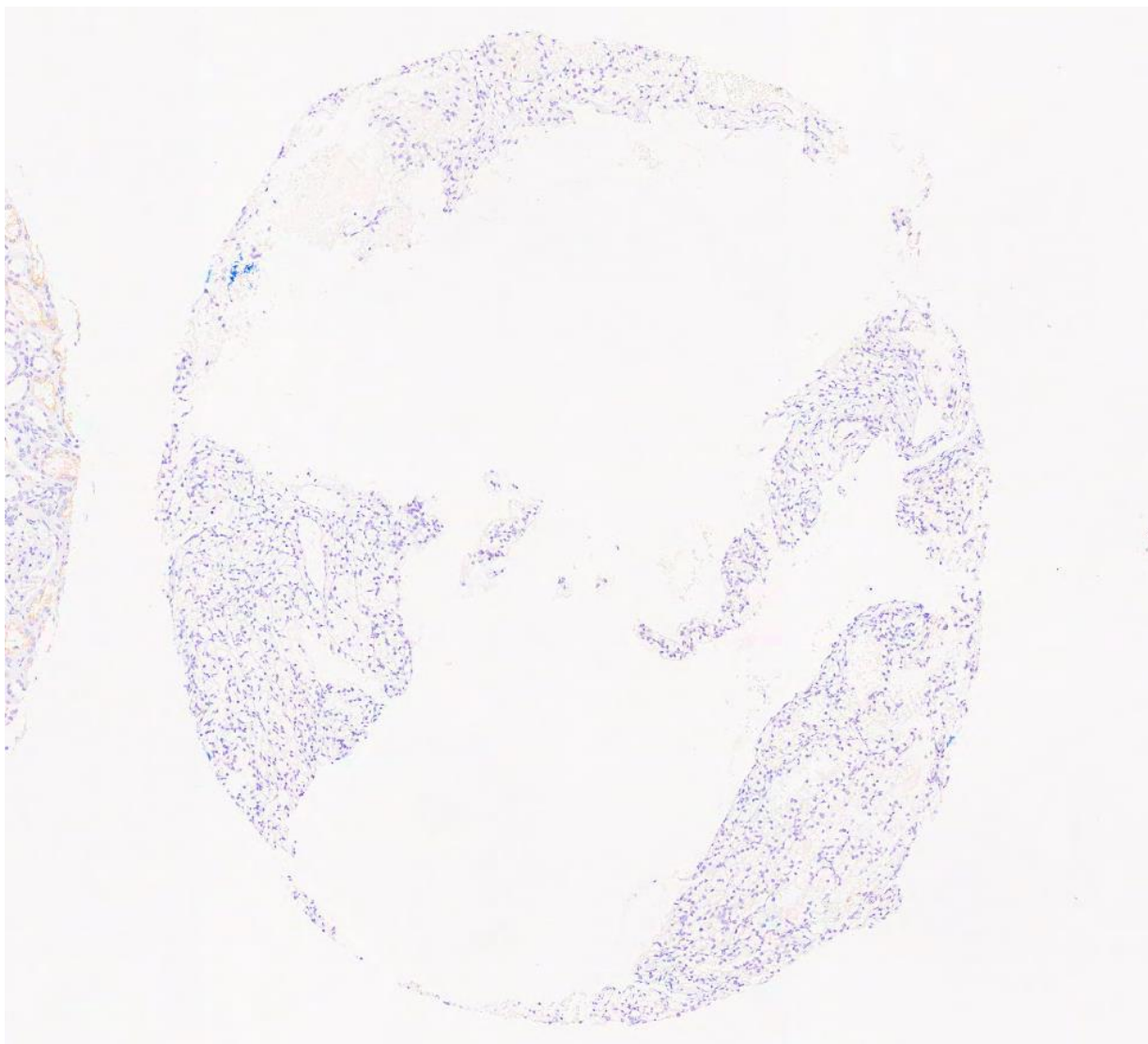

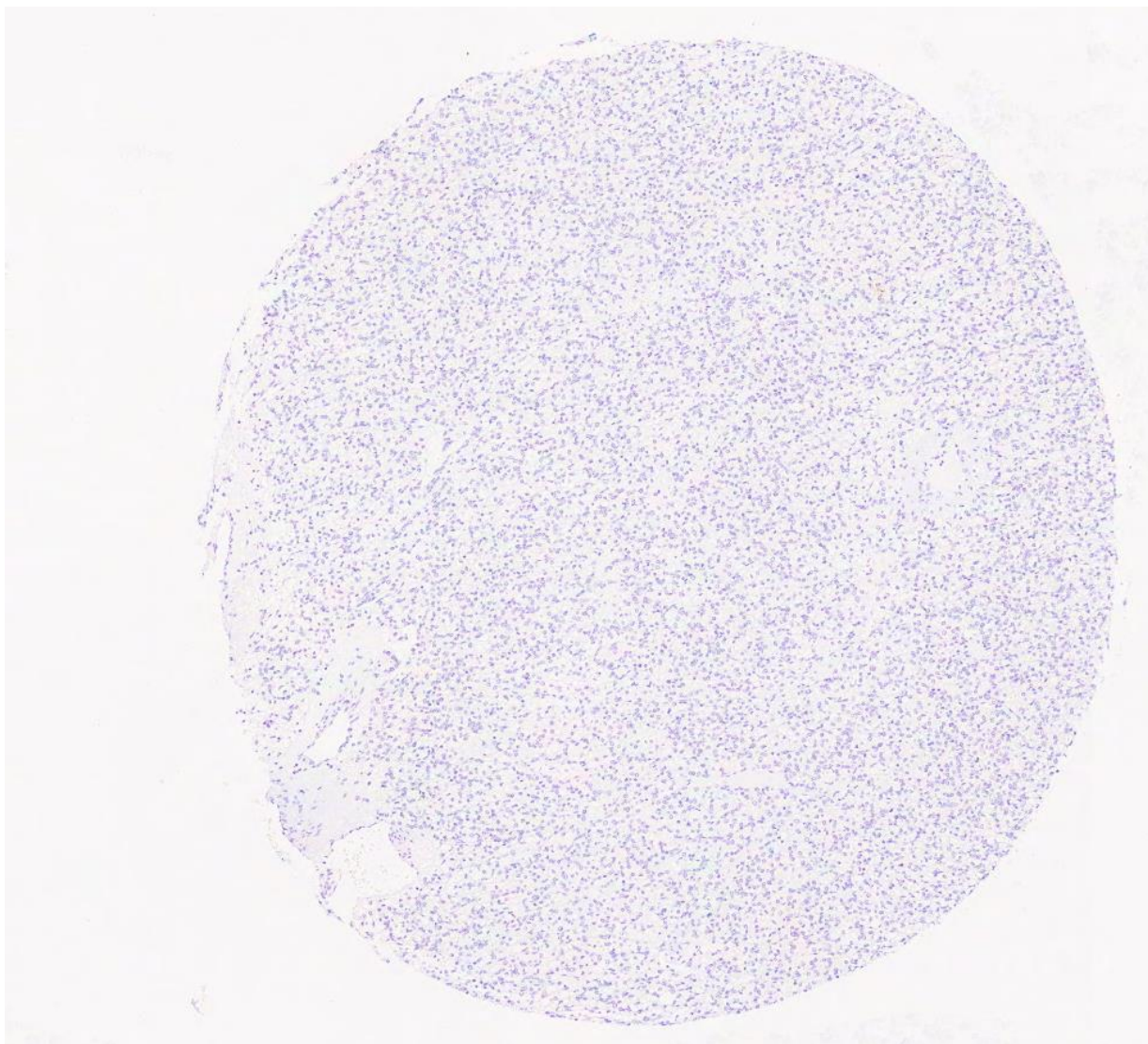

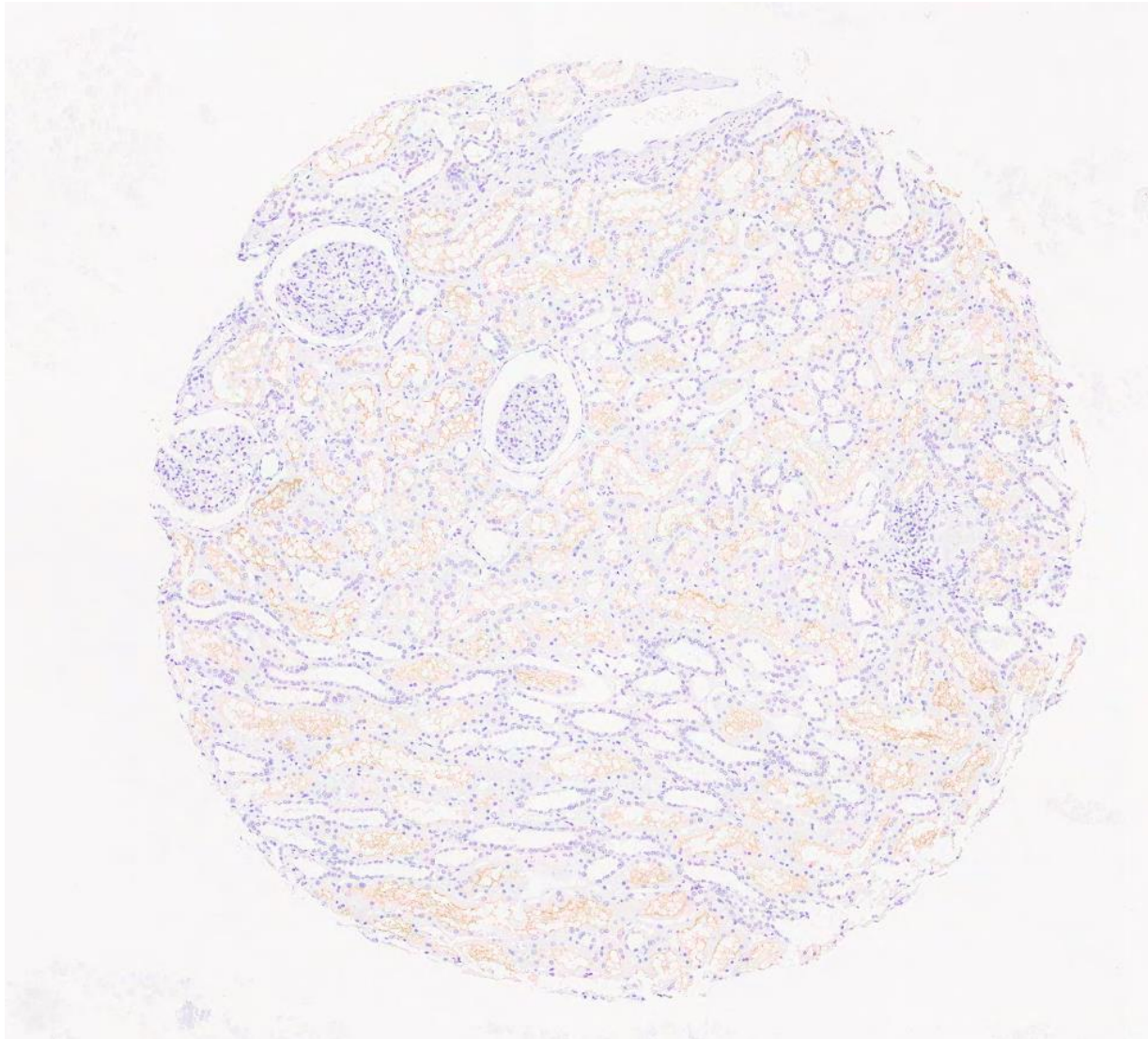

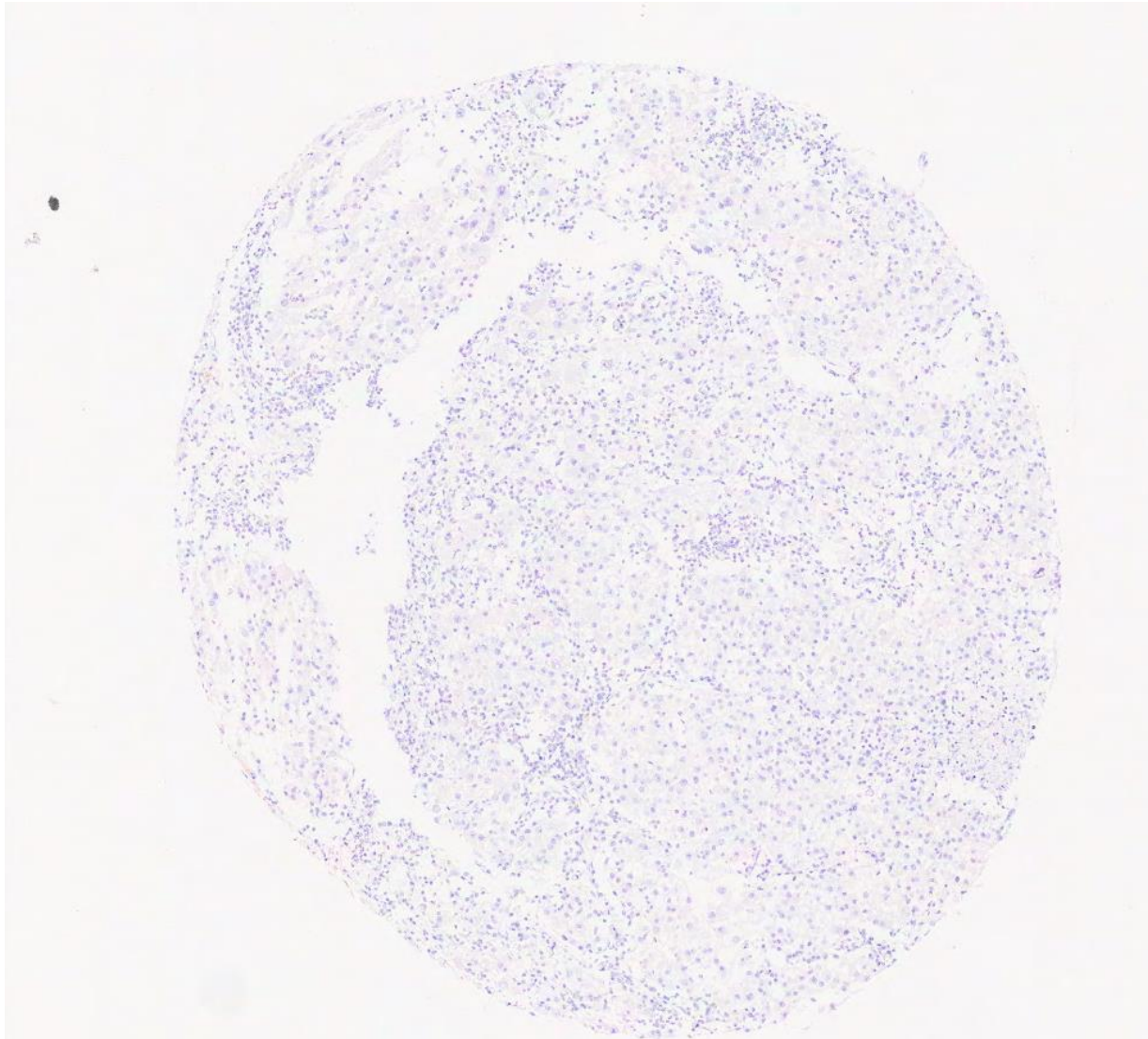

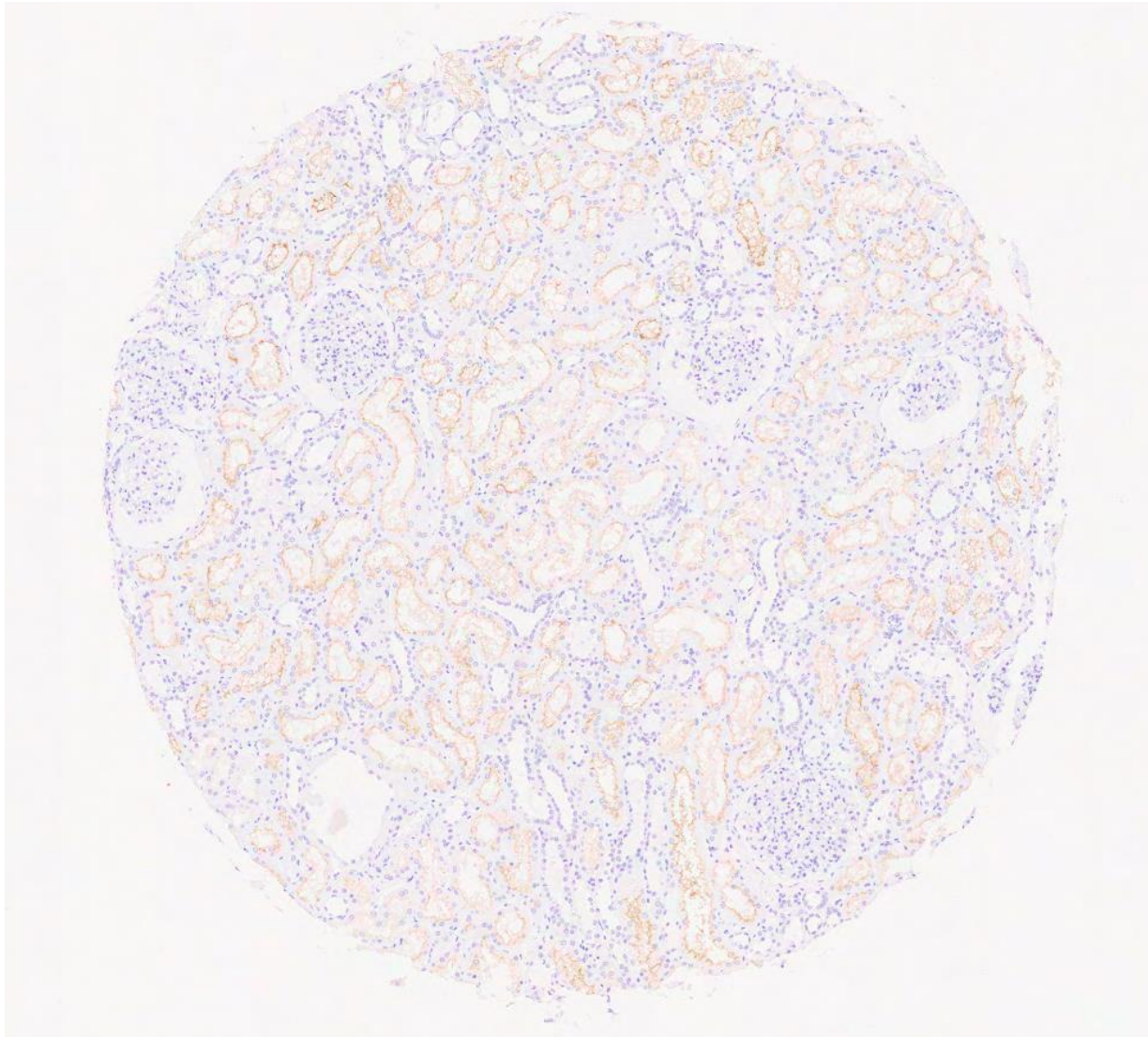

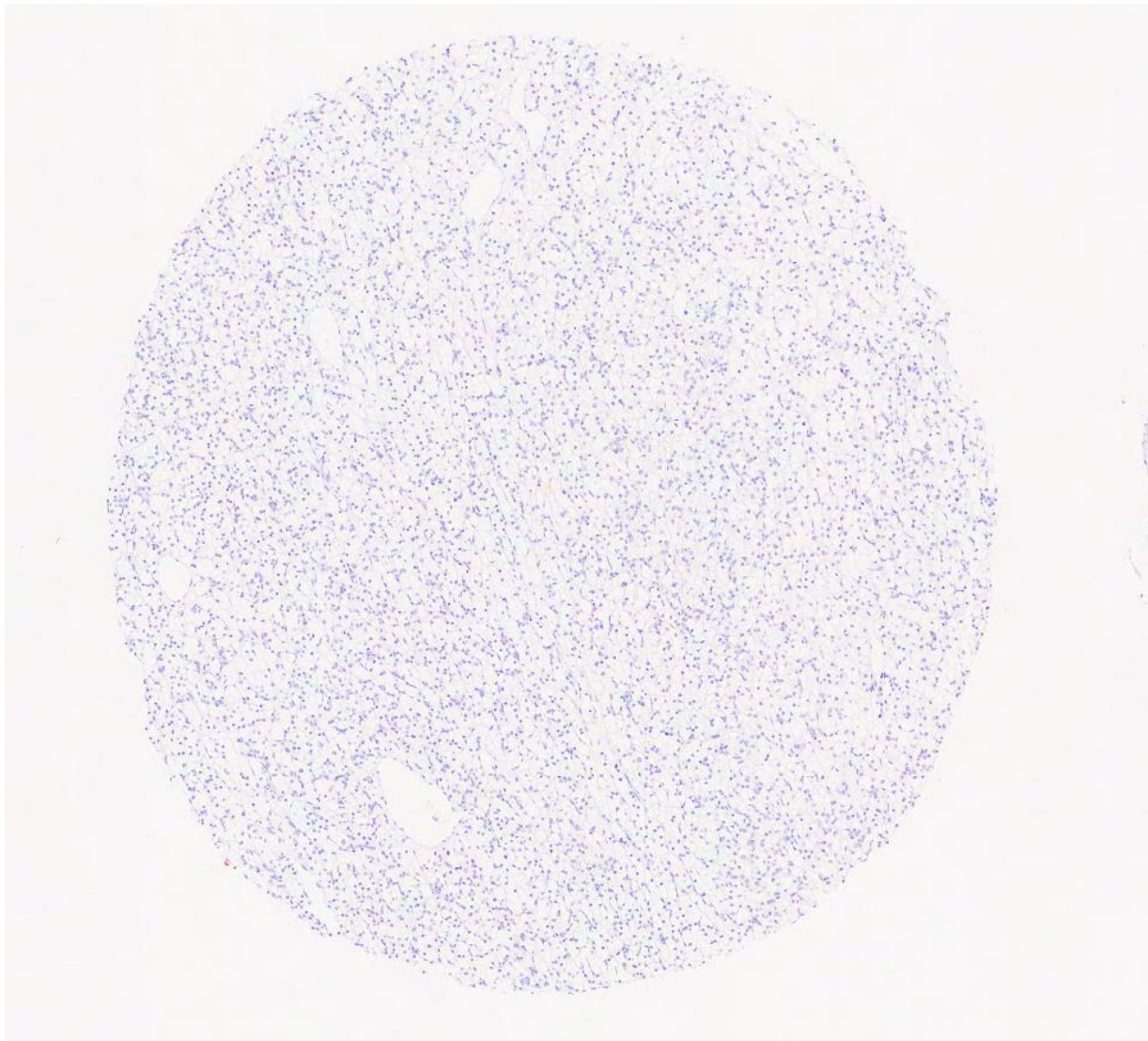

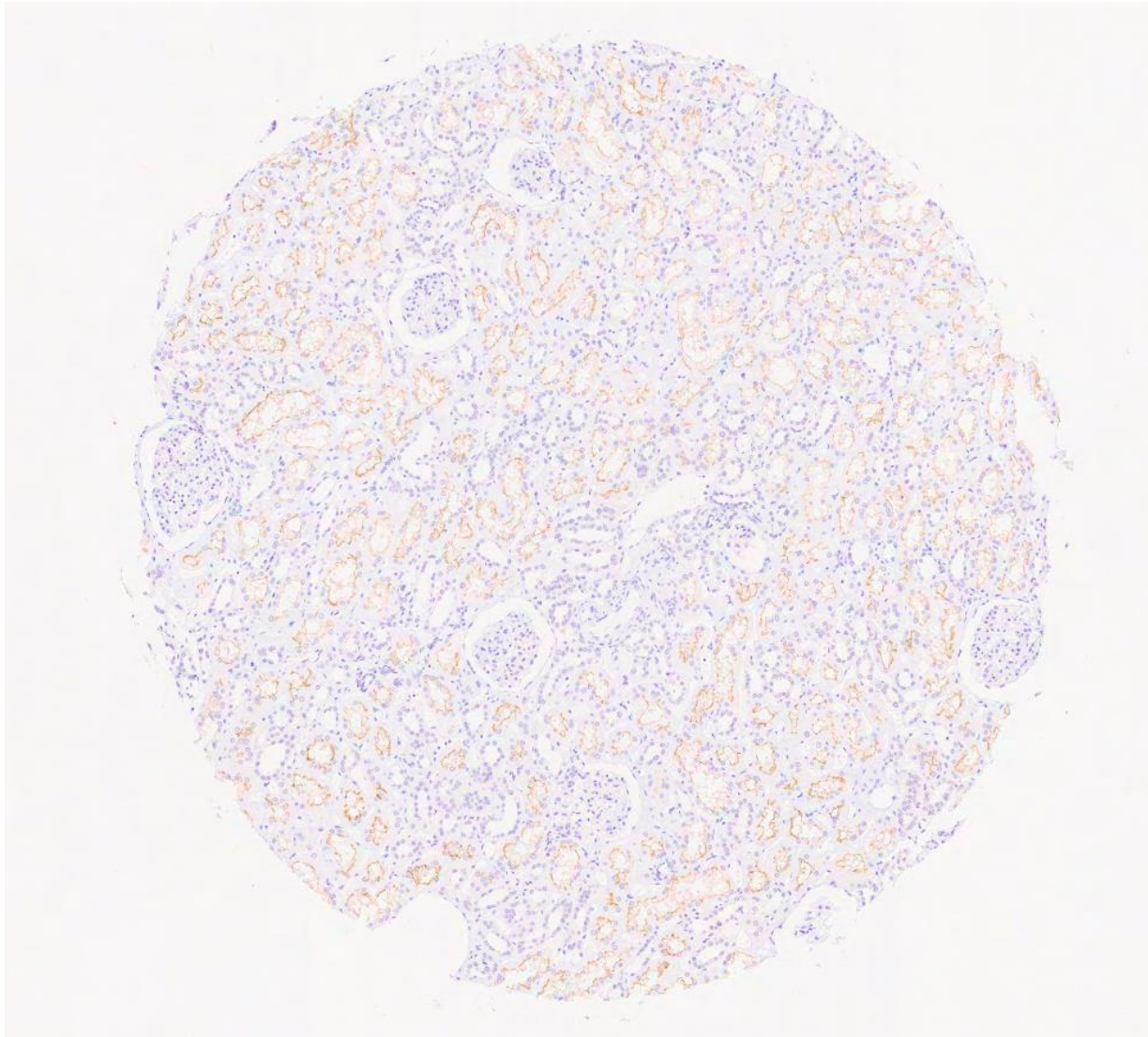

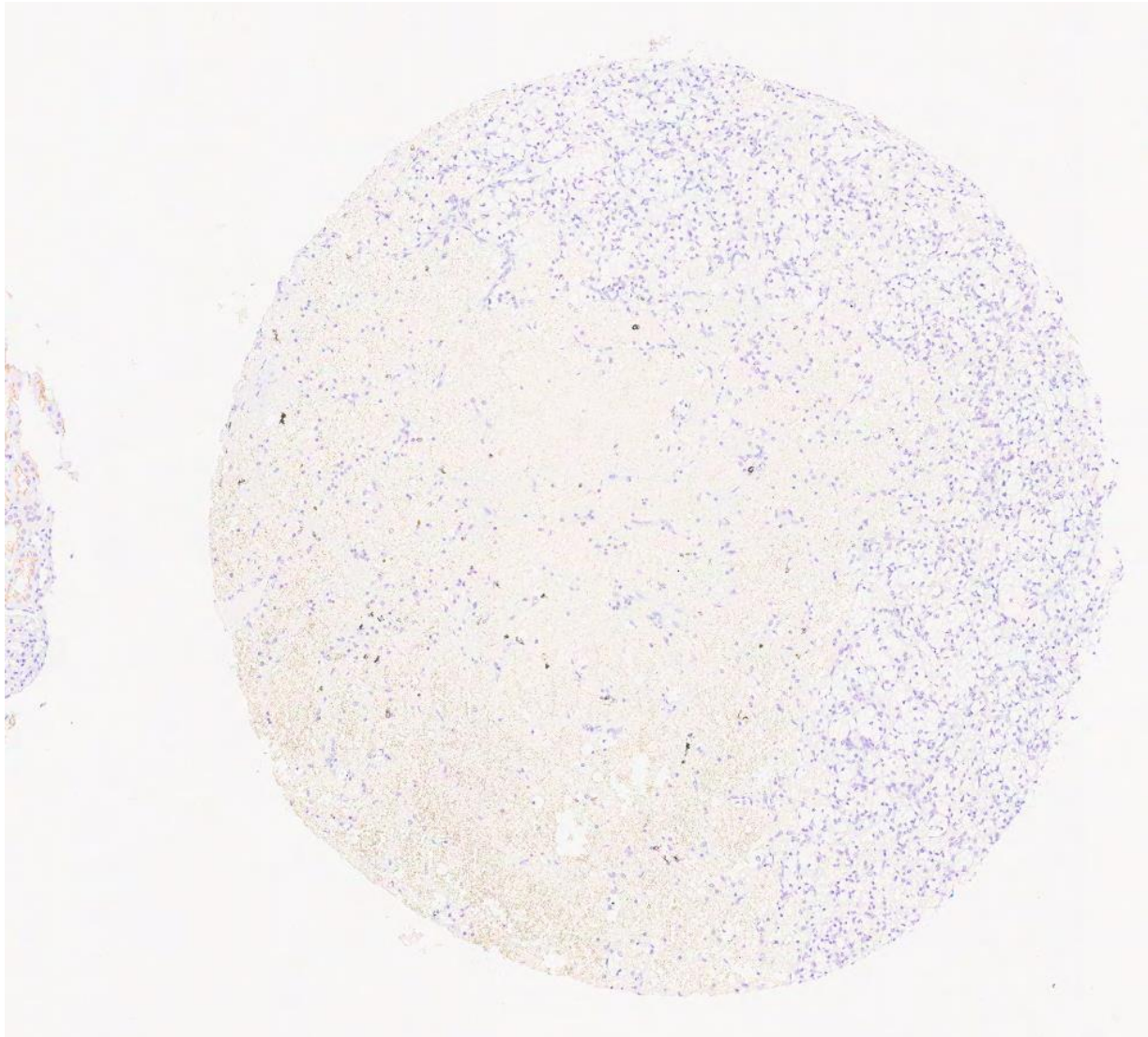

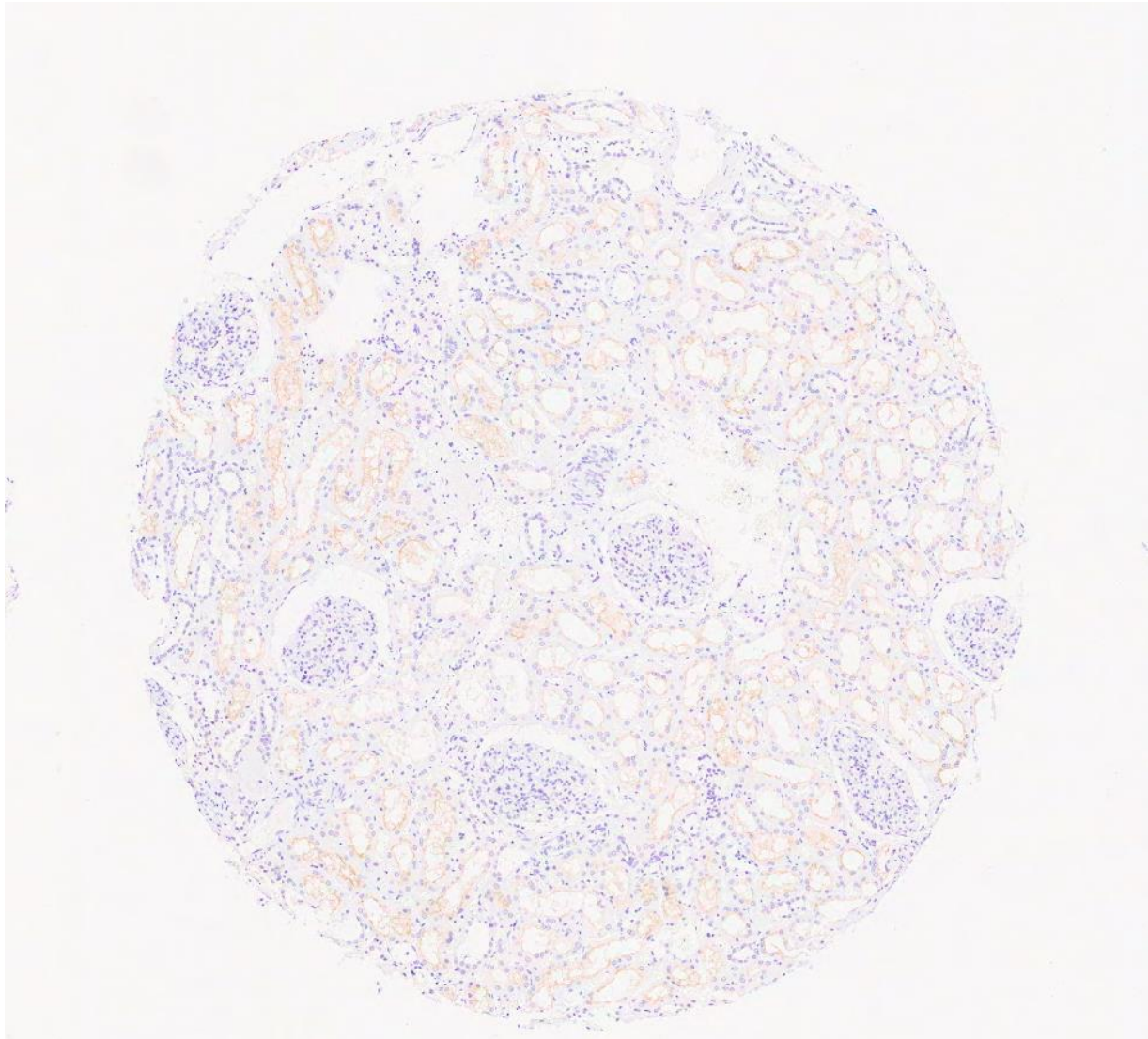

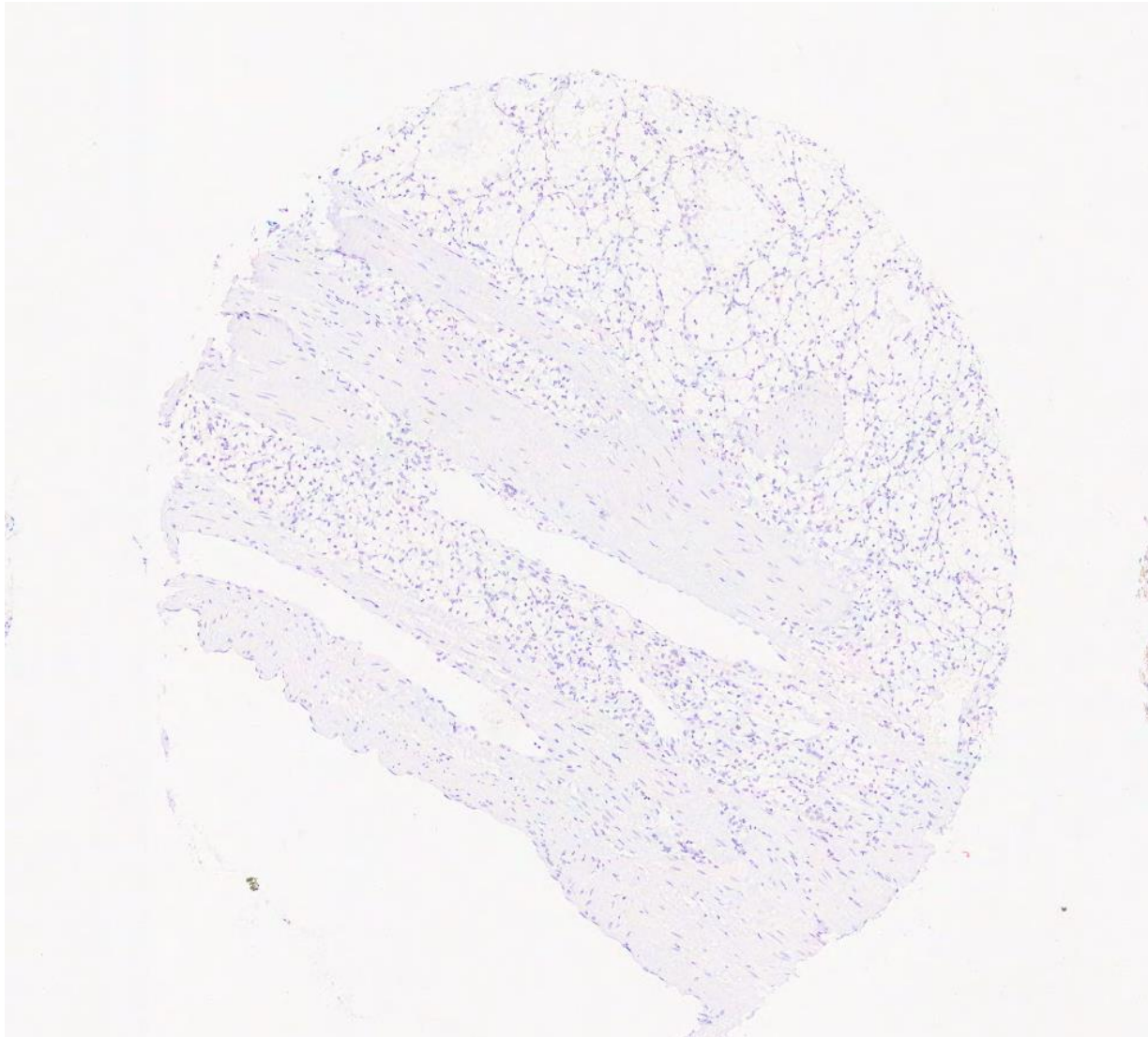

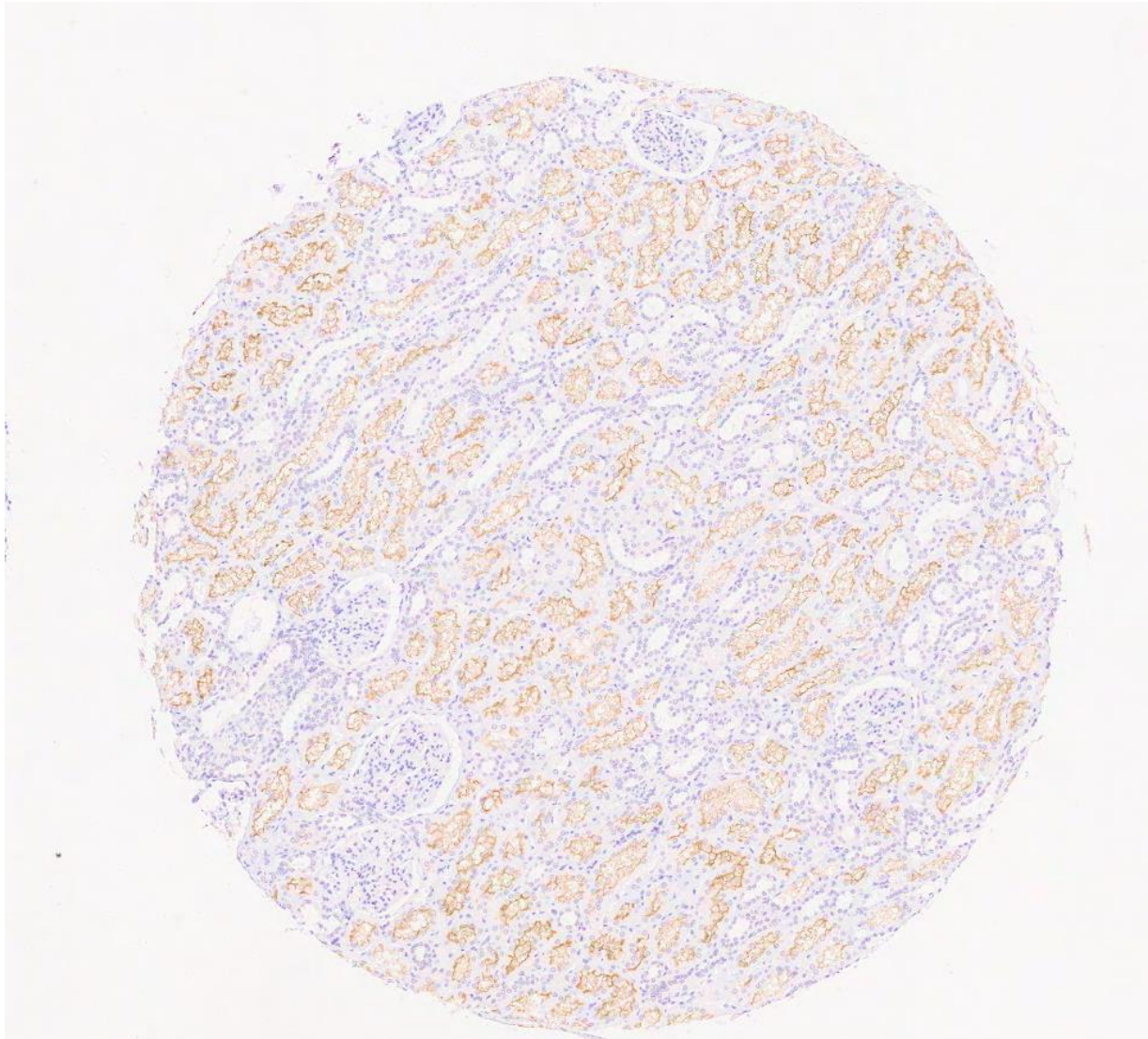

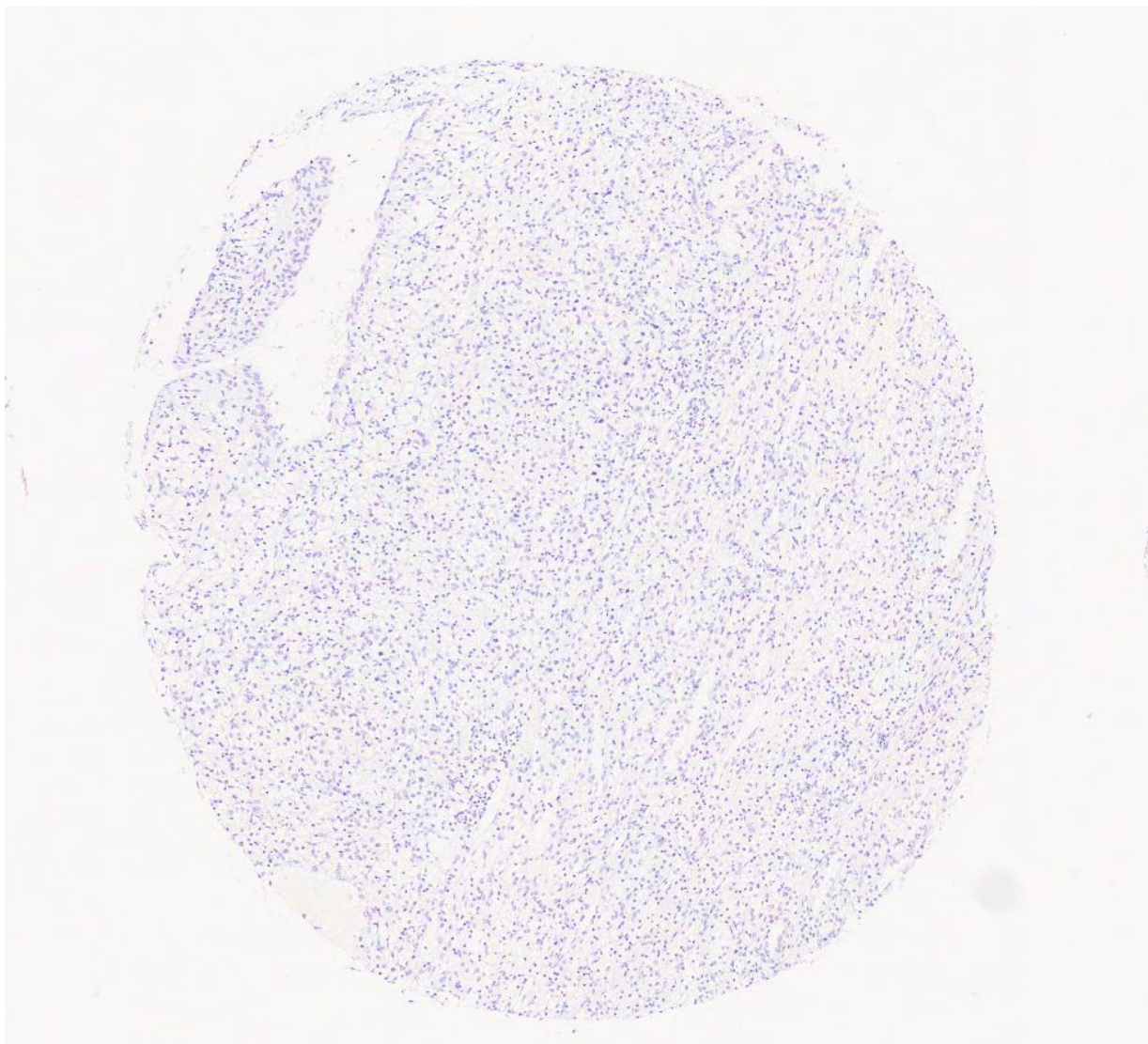

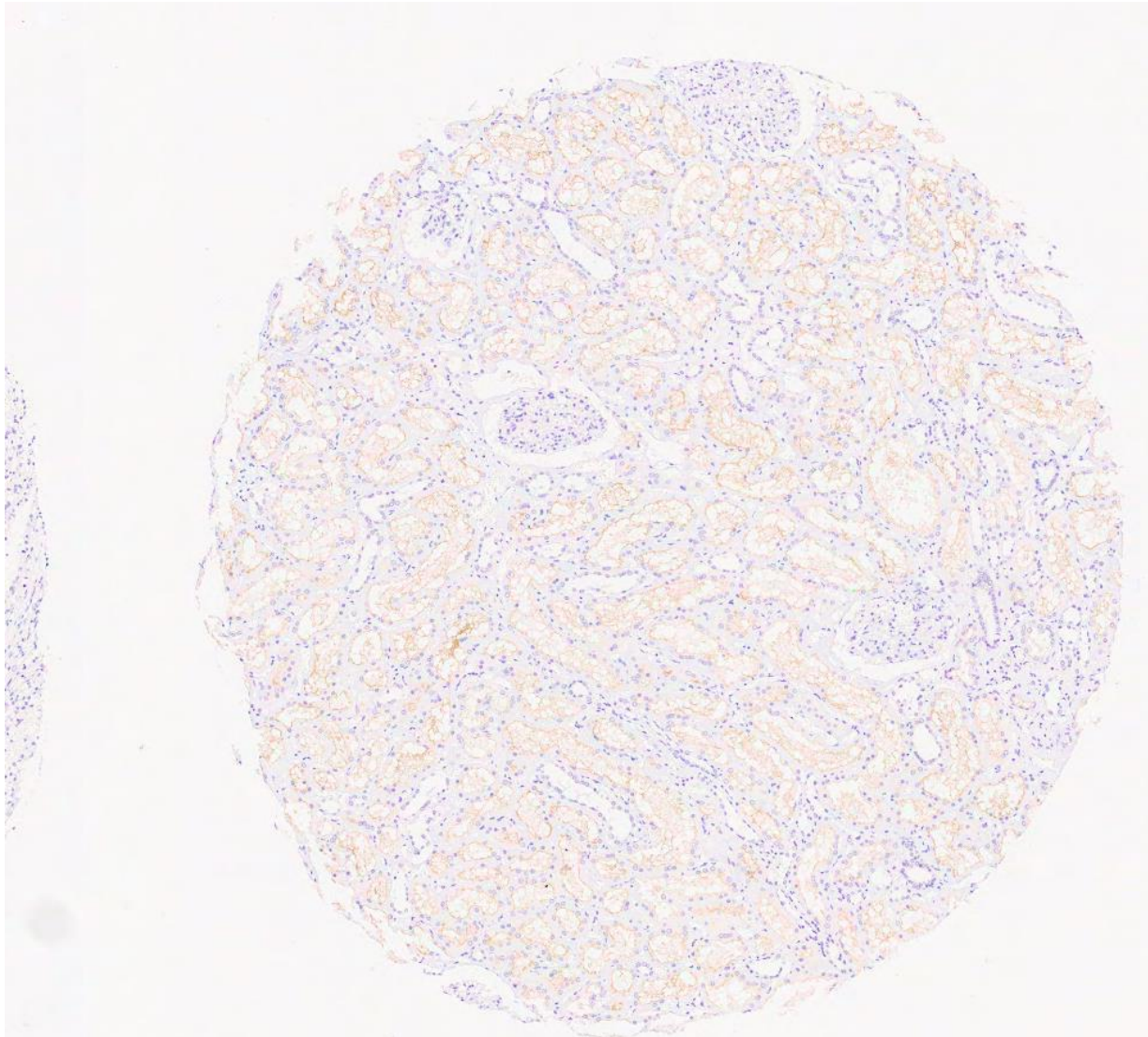

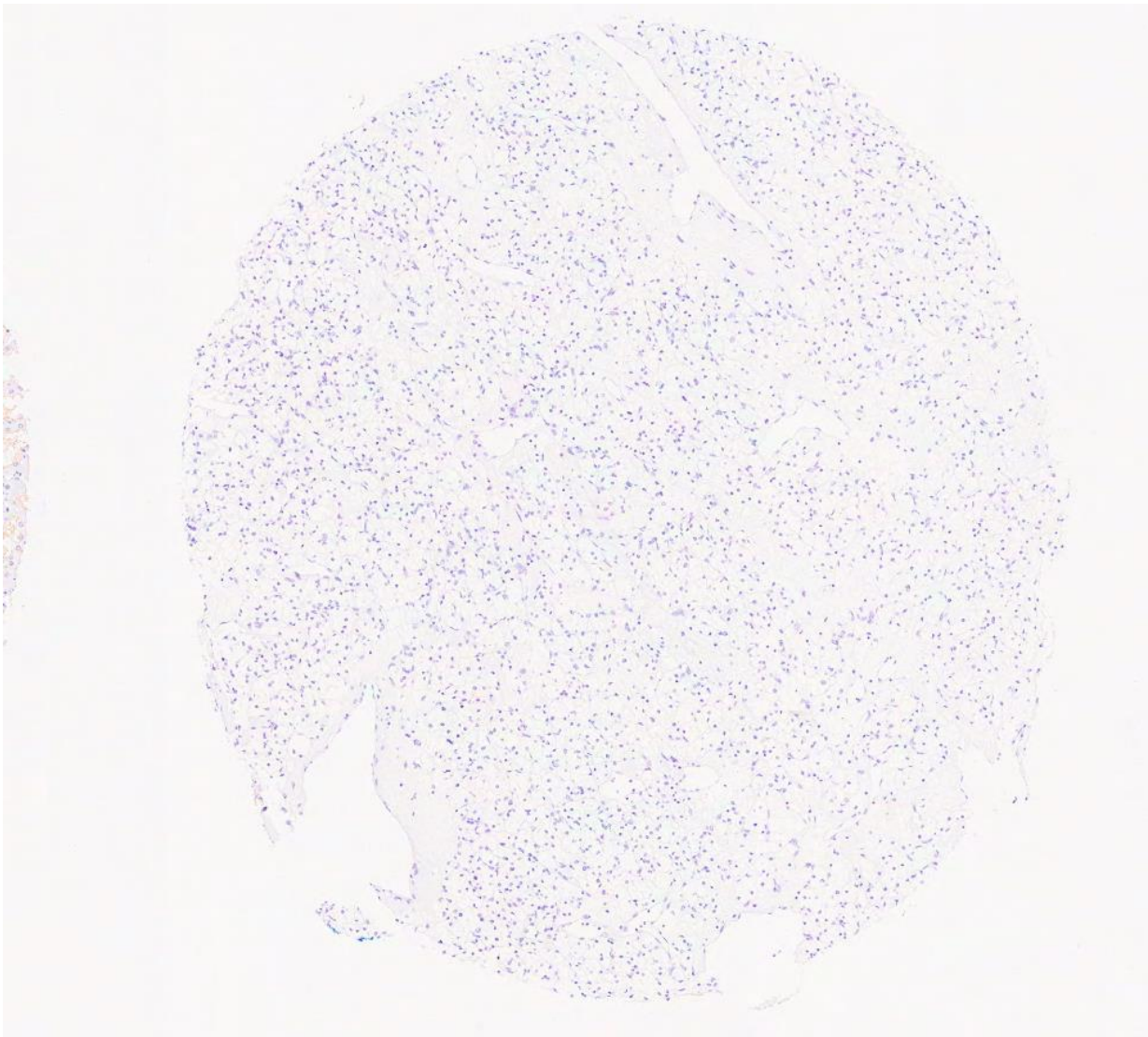

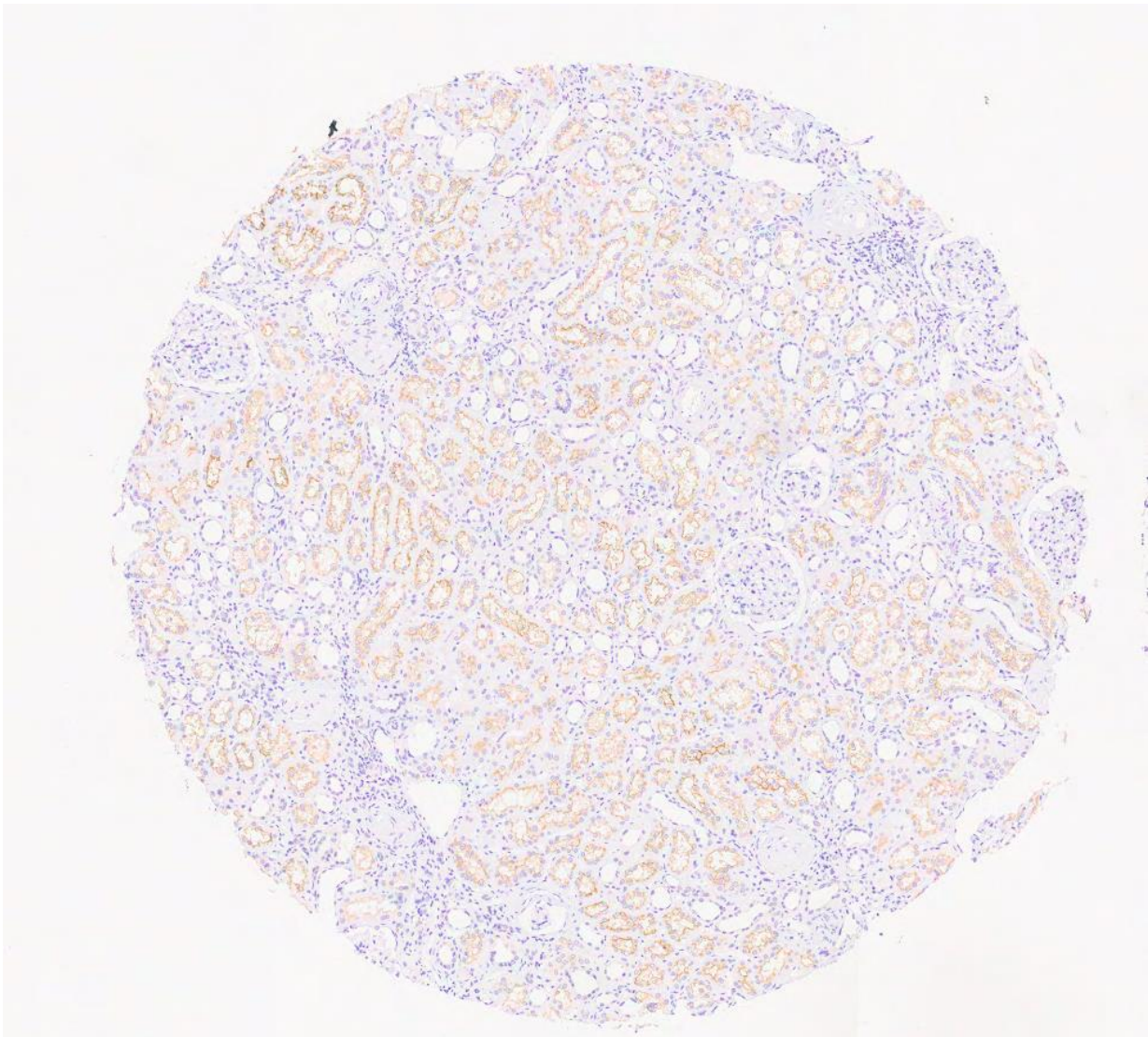

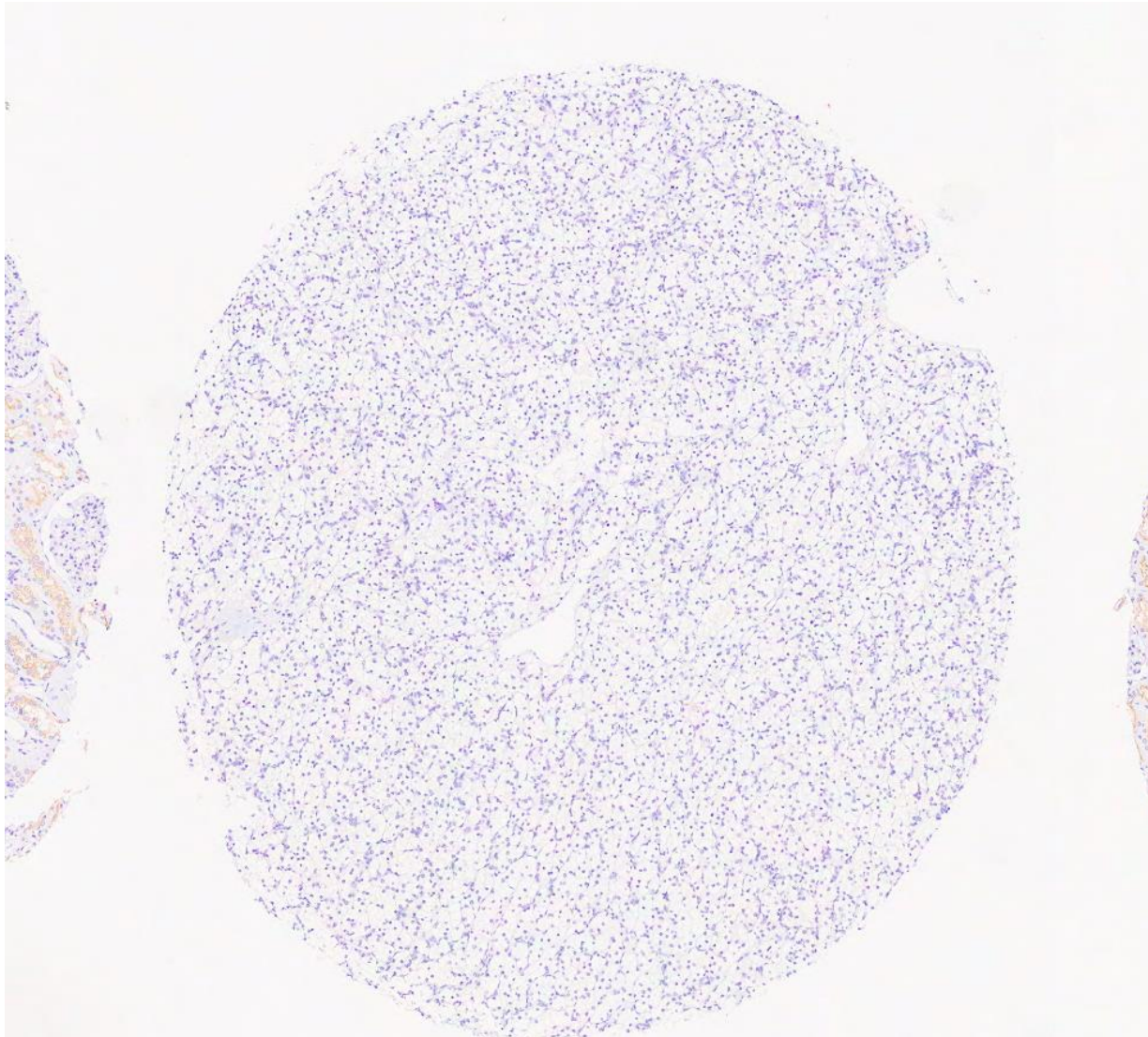

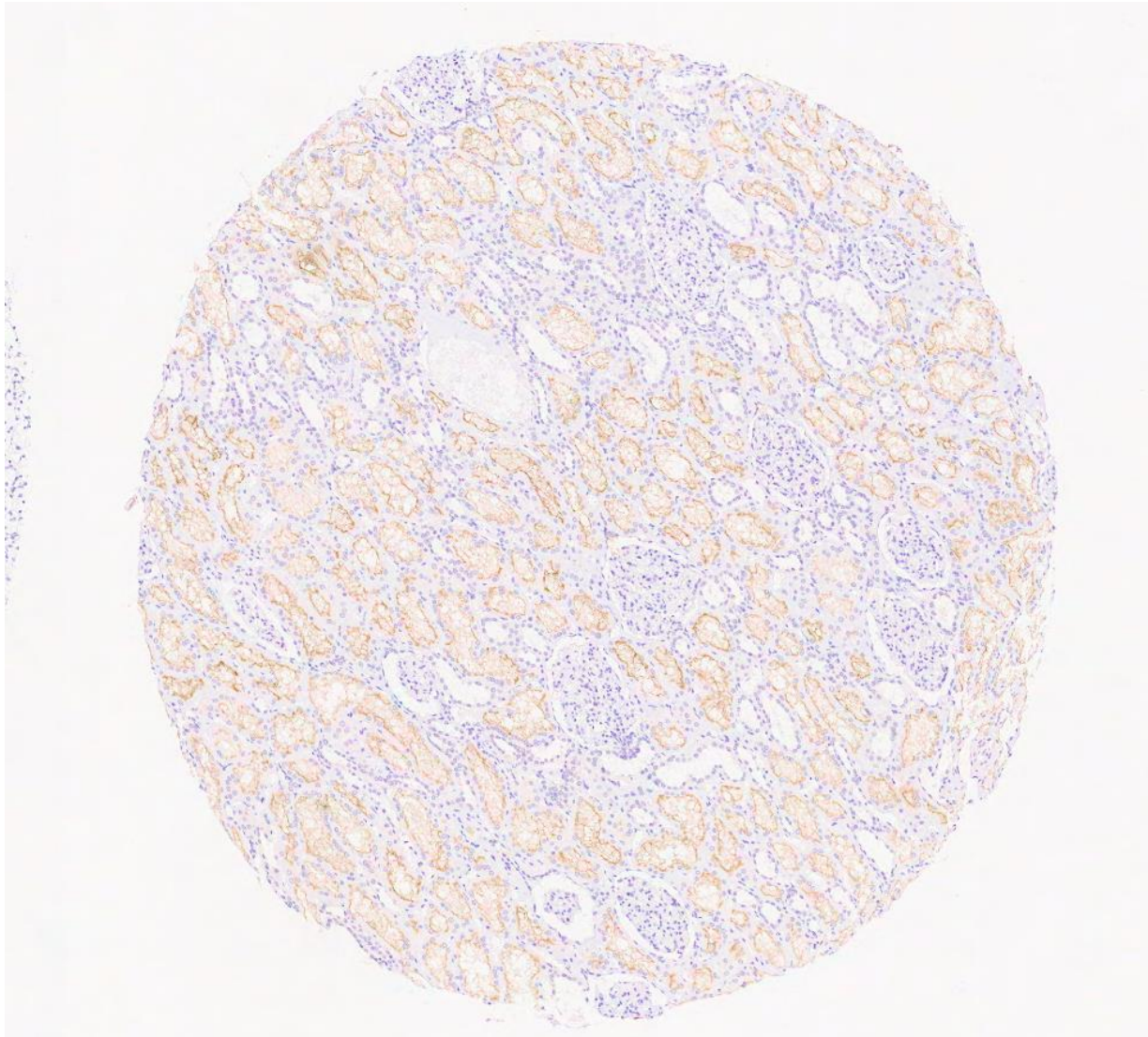

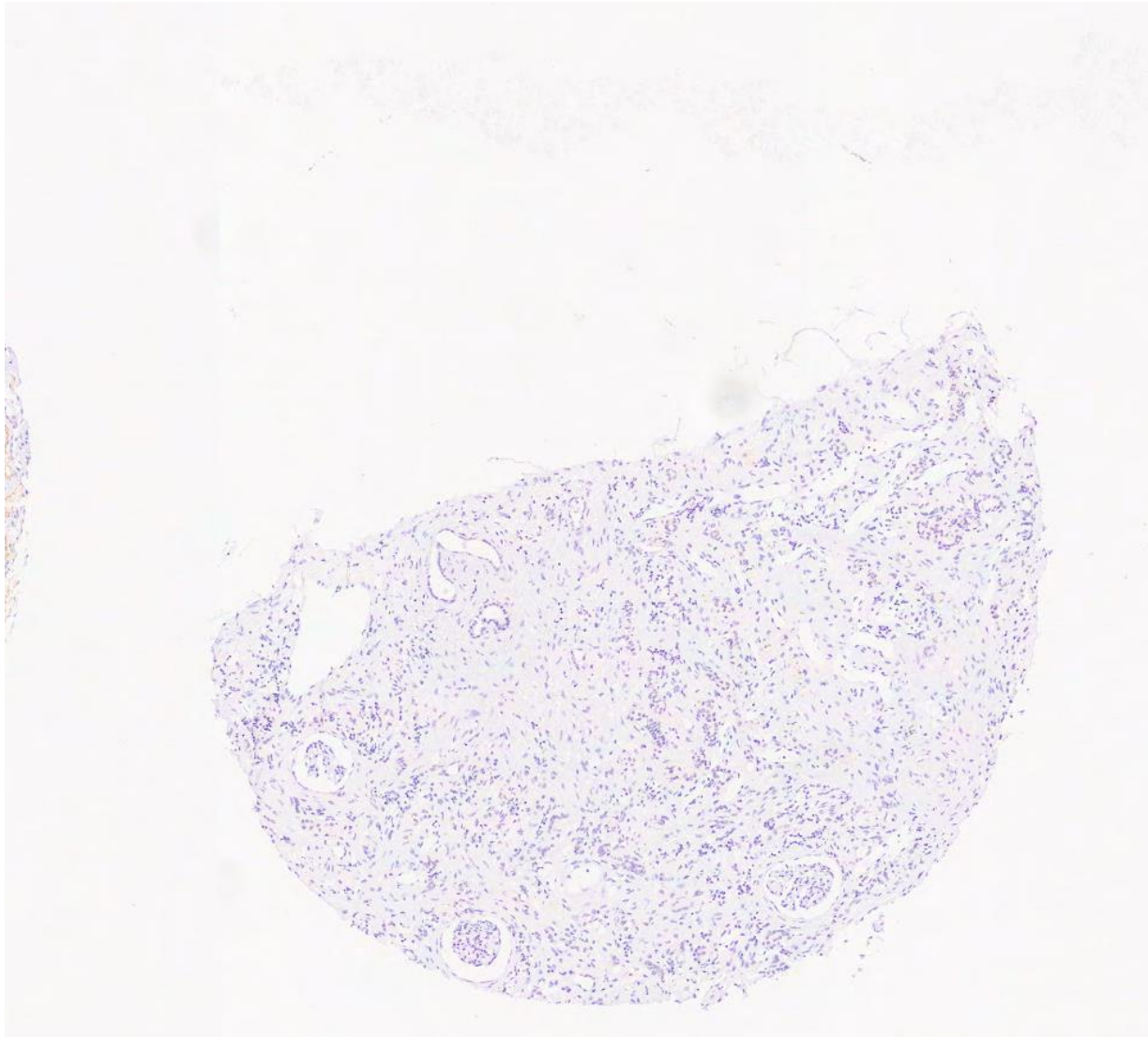

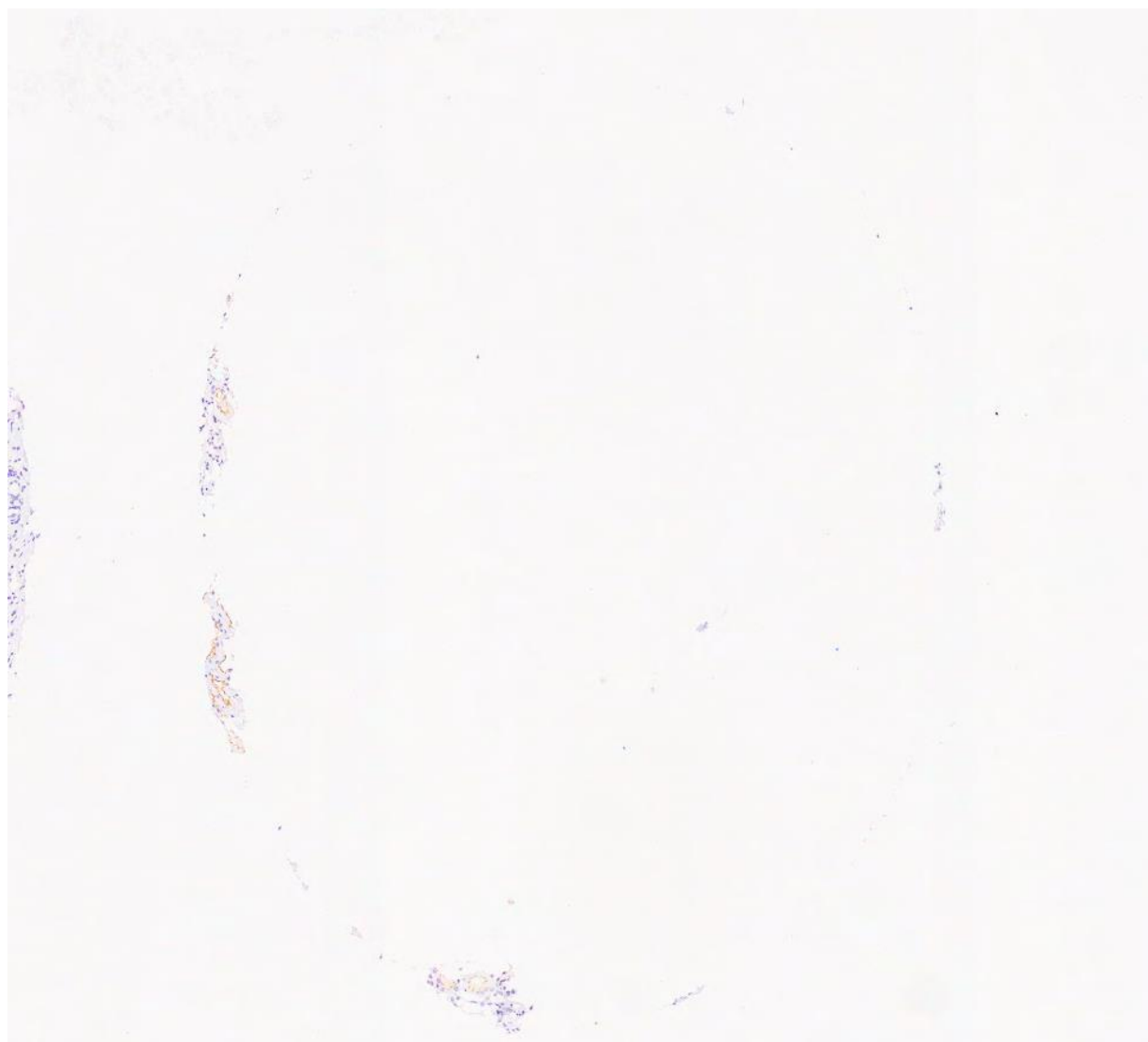

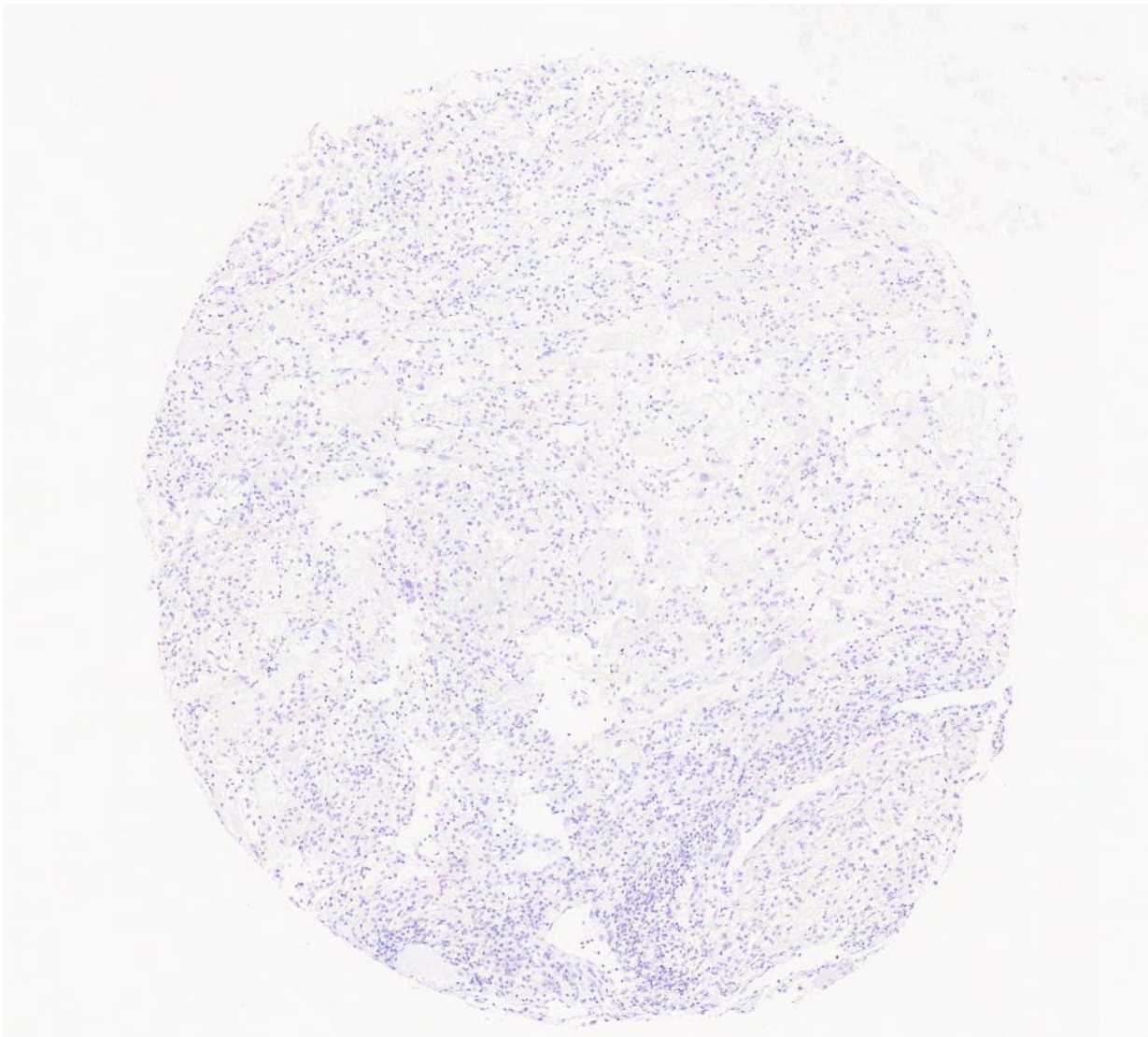

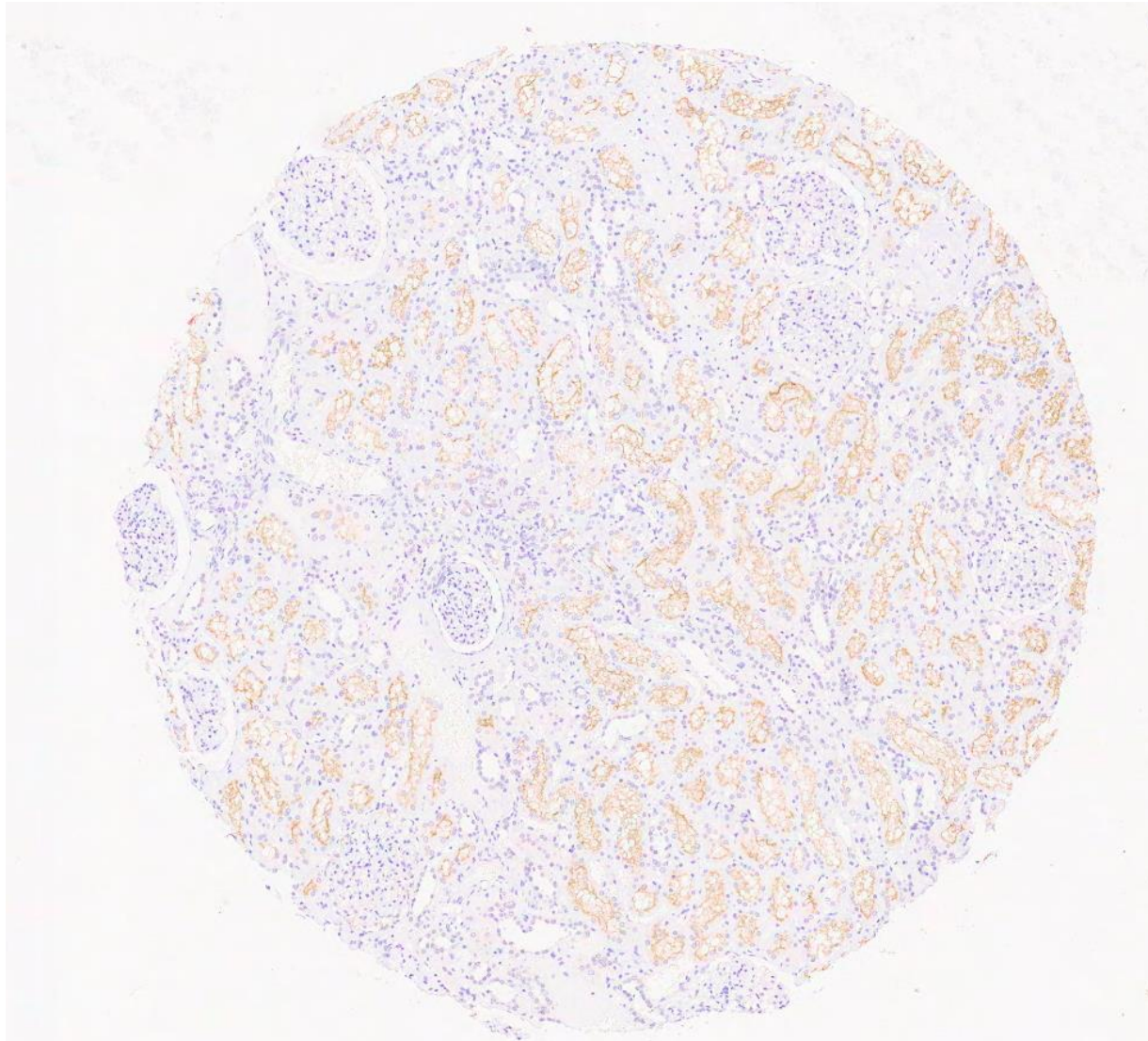

Supplement: Supplementary file 3 [file DataSheet_1.pdf]
