# Supplementary material for: A comprehensive investigation discovered the novel methyltransferase METTL24 as one presumably prognostic gene for kidney renal clear cell carcinoma potentially modulating tumor immune microenvironment
Source: Front Immunol. 2022 Oct 14;13:926461. doi: 10.3389/fimmu.2022.926461 (PMC9613963; doi:10.3389/fimmu.2022.926461)
Supplement: Supplementary file 4 [file DataSheet_2.pdf]

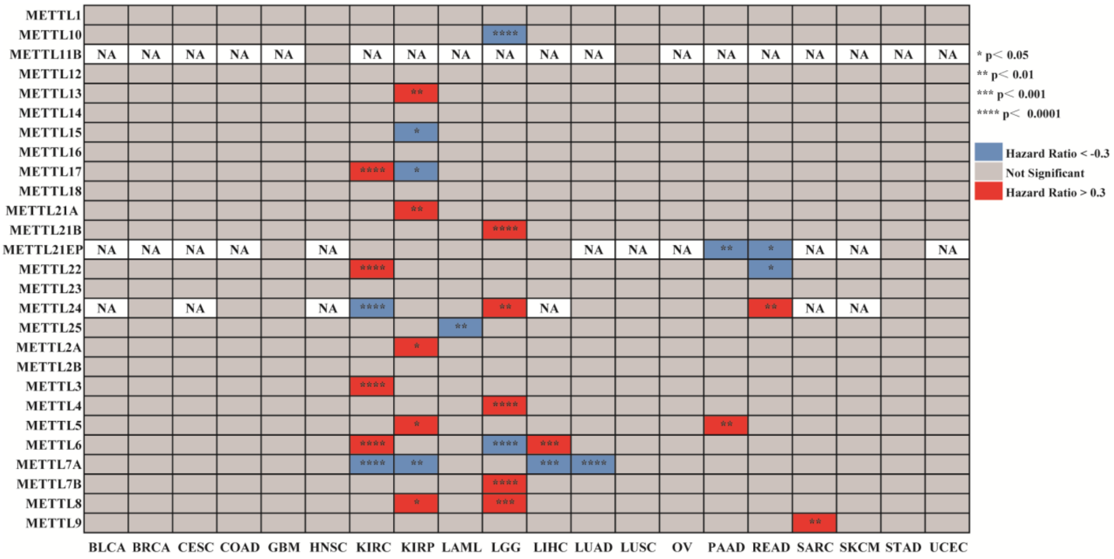

Supplementary Figure S1 The correlation between METTL genes' expression and hazard ratios of multiple cancers.

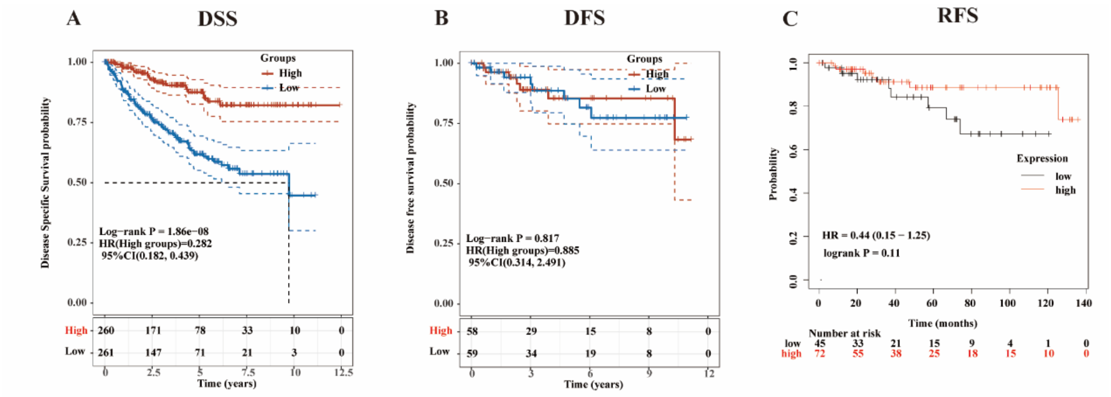

Supplementary Figure S2 The impact of METTL24 expression on disease free survival rate (DSS), disease free survival rate (DFS) and relapse free survival rate (RFS) of renal clear renal cell carcinoma (KIRC) patients. The Kaplan-Meier curve showed the impact of METTL24 expression on the **(A)** DSS**, (B)** DFS, and **(C)** RFS of KIRC patients.

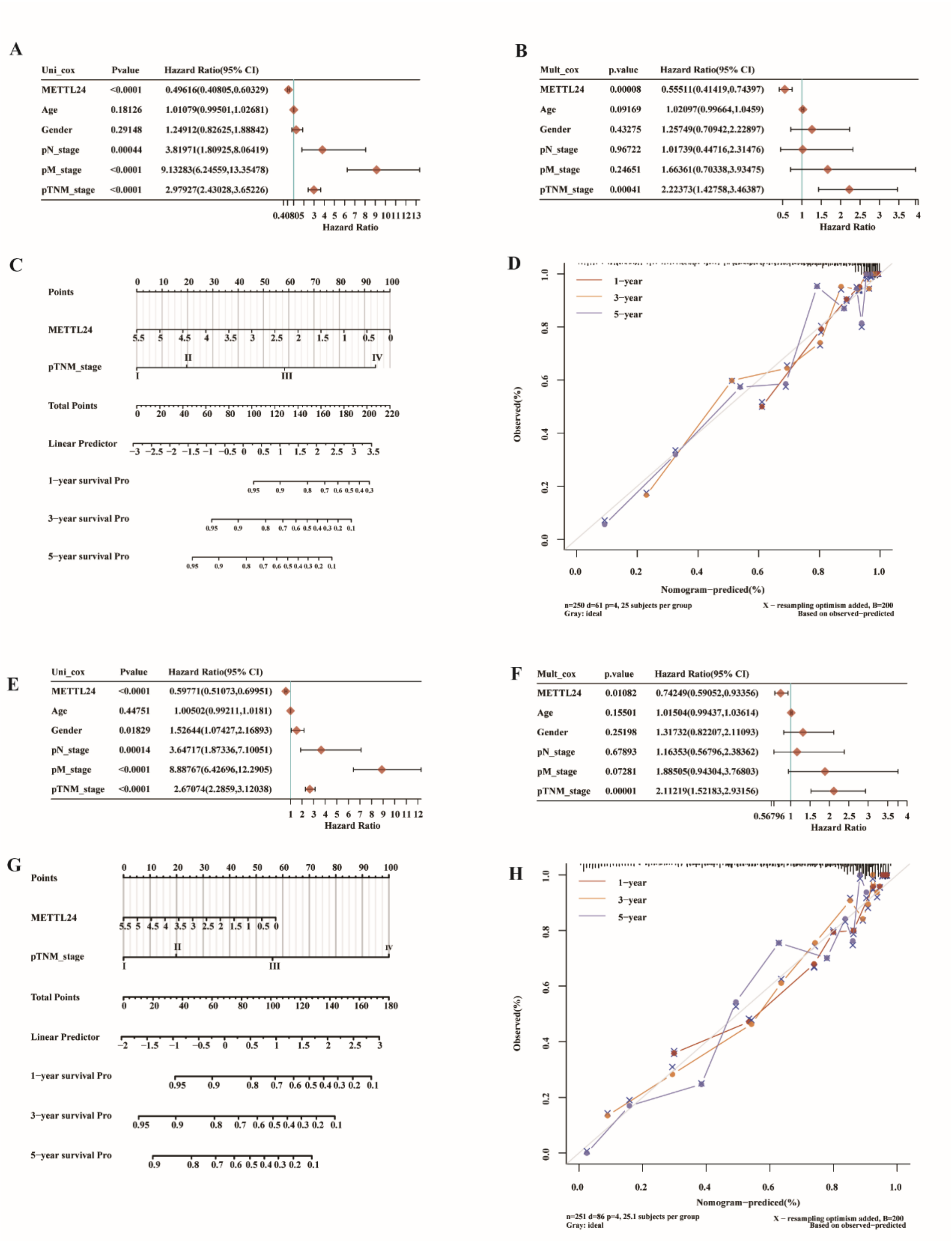

Supplementary Figure S3 METTL24 as one potential independent prognostic gene for KIRC as analyzed using the PFS and DSS data. Based on the PFS data, **(A)** the forest image showed METTL24 as a potential risk factor for KIRC as analyzed using the univariate Cox regression, **(B)** the forest image showed METTL24 as one potential prognostic factor for KIRC as analyzed using the multivariate Cox regression, **(C)** a predictive nomogram based on the METTL24 risk score and other clinicopathological variables predicted the 1-, 3-, and 5-year survival rates of KIRC patients, and **(D)** the Calibration curves indicated the agreement between anticipated and actual survival rates after 1, 3, and 5 year. Based on the DSS data, **(E)** the forest

image showed METTL24 as a potential risk factor for KIRC as analyzed using the univariate Cox regression, **(F)** the forest image showed METTL24 as one potential prognostic factor for KIRC as analyzed using the multivariate Cox regression, **(G)** a predictive nomogram based on the METTL24 risk score and other clinicopathological variables predicted the 1-, 3-, and 5-year survival rates of KIRC patients, and **(H)** the Calibration curves indicated the agreement between anticipated and actual survival rates after 1, 3, and 5 years.

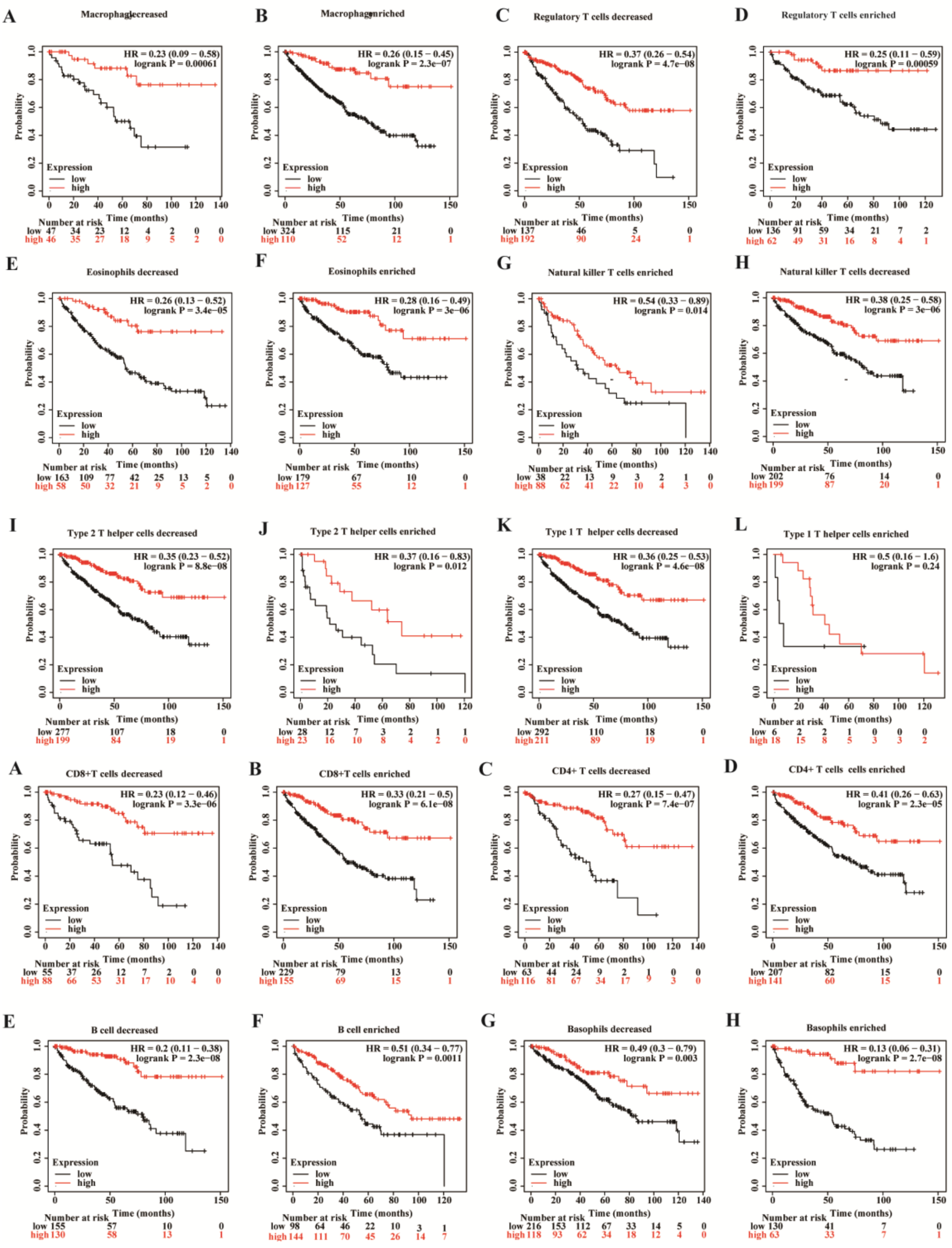

Supplementary Figure S4 The influence of METTL24 expression on the overall survival rate (OS) of KIRC patients with high or low infiltration degrees of immune cells. The impact of METTL24 expression on the OS of KIRC patients with enriched or decreased infiltration ratios of **(A-B)** macrophages, **(C-D)** regulatory T cells, **(E-F)** eosinophils, **(G-H)** natural killer T cells, **(I-J)** type 2 helper cells, **(K-L)** type 1 helper cells. The survival analysis was performed on the Kaplan-Meier plotter database.
