# Supplementary material for: A comprehensive investigation discovered the novel methyltransferase METTL24 as one presumably prognostic gene for kidney renal clear cell carcinoma potentially modulating tumor immune microenvironment
Source: Front Immunol. 2022 Oct 14;13:926461. doi: 10.3389/fimmu.2022.926461 (PMC9613963; doi:10.3389/fimmu.2022.926461)
Supplement: Supplementary file 5 [file DataSheet_3.pdf]

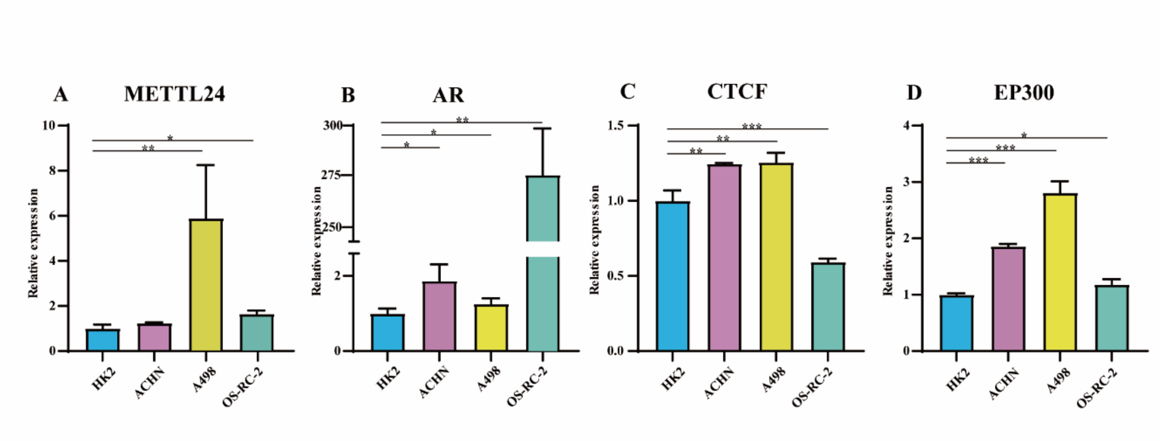

Supplementary Figure S5 RT-qPCR analyses identified the mRNA expression of METTL24 and its potential transcription factors in different cell lines. The mRNA expression levels of METTL24 **(A),** AR **(B),** CTCF **(C),** and EP300**(D)** in human immortalized renal cell lines HK2 and human renal cancer cell lines (ACHN, A498, OS-RC-2**)** were tested by RT-qPCR.
